# Supplementary material for: Comprehensive analysis platform to understand, remedy, and eliminate amyotrophic lateral sclerosis (CAPTURE ALS): Study protocol for a Canadian multicenter, multimodal, longitudinal observational study
Source: PLoS One. 2025 Dec 4;20(12):e0332430. doi: 10.1371/journal.pone.0332430 (PMC12677780; doi:10.1371/journal.pone.0332430)
Supplement: S1 Appendix — (PDF) [file pone.0332430.s001.pdf]

CAPTURE ALS

ID: *CAPT* **Visit:** 1 (*SCREENING*)
Date:

Screen Visit /Onset Information Form

Date performed: ________________

Information collected by: ____________________ ____________________
name *signature*

DEMOGRAPHICS

Age at time of screen visit: ____________

Sex at birth: Male ☐ Female ☐

Gender: Man ☐ Woman ☐ Non-Binary ☐ Prefer not to say ☐

Race*: Black☐ East Asian☐ Indigenous☐ Latin American☐ Middle Eastern☐ South Asian☐ Southeast Asian☐ White☐ Unknown☐ Prefer not to say☐ Other☐ If other, state: ____________

****Black (African, African Canadian, Afro-Caribbean descent); East Asian (Chinese, Japanese, Korean, Taiwanese descent); Indigenous (First Nation, Inuk/Inuit, Métis); Latin American (Hispanic of Latin American descent); Middle Eastern (Arab, Persian, West Asian descent e.g. Afghan, Egyptian, Iranian, Kurdish, Lebanese, Turkish); South Asian (Bangladeshi, Indian, Indo-Caribbean, Pakistani, Sri Lankan); Southeast Asian (Cambodian, Filipino, Indonesian, Thai, Vietnamese); White (European descent)***

Hand preference: Right ☐ Left ☐ Ambidextrous ☐

Occupation: Working ☐ Not working ☐ Prefer not to say☐

Highest level of education: Elementary☐ High school☐ Post-secondary☐

Years of education beginning at Grade 1: ________ years

Preferred language: English ☐ French ☐ Other ☐ If other, please state: ________________

Age participant began learning English/French: __________

If *not* 0, ask participant for self-rated level of understanding of English/French on a scale of 0-10: _________

*If rating is < 7 consider not administering **neurocognitive** tests.*

Is participant able to read English? Yes ☐ No ☐

Birthplace (City, Country): ____________________________

Where was participant primarily raised (City, Country)? _________________________

Does participant communicate by speaking? Yes ☐ No ☐

If no, what is used to communicate? Gestures ☐ Writing ☐ Technology ☐ Other ☐ If other, list: ______________________

Has participant experienced changes in his/her speech in the past 2 years? Yes ☐ No ☐

Has participant ever participated in a clinical trial? Yes ☐ Past ☐ Unknown ☐ Never ☐

Has the participant ever participated in a CALSNIC study? Yes ☐ No ☐

If yes, CALSNIC Participant ID: ________________

CAPTURE ALS

ID: *CAPT* **Visit:** 1 (*SCREENING*)

Date:

ONSET INFORMATION (patients only)

***Symptom onset date (MM-DD-YYYY):**

**When limb weakness, speech/swallowing difficulties, dyspnea, or generalized weakness were first noticed*

Diagnosis date (MM-DD-YYYY):

Site of onset of progressive weakness (select one): Bulbar☐ Truncal☐ *Generalized☐ Limb☐ Respiratory☐

**Choose generalized if both bulbar and limb weakness reported at the same time*

If limb onset, upper or lower? Upper extremity ☐ Lower extremity ☐ Both ☐ Unknown☐

If limb onset, what side? Left ☐ Right ☐ Both ☐ Unknown ☐

First symptom if not weakness? (select one):

Fasciculations ☐ Atrophy ☐ Cramping ☐ Stiffness ☐ Weight loss ☐ Cognitive/Behavioural ☐ Other ☐ N/A ☐

If other, or cognitive/behavioural, please provide details:

Other first symptom onset date (MM-DD-YYYY):

Notes:
