# Supplementary material for: Comprehensive analysis platform to understand, remedy, and eliminate amyotrophic lateral sclerosis (CAPTURE ALS): Study protocol for a Canadian multicenter, multimodal, longitudinal observational study
Source: PLoS One. 2025 Dec 4;20(12):e0332430. doi: 10.1371/journal.pone.0332430 (PMC12677780; doi:10.1371/journal.pone.0332430)
Supplement: S2 Appendix — (PDF) [file pone.0332430.s002.pdf]

CAPTURE ALS

| **ID:** *CAPT* | **Visit: 1 (*SCREENING)*** |
| --- | --- |
| **Date:** | |

# Study Status Form

**Date of Assessment:** ______________________

**Assessment performed by:** ______________________ (*name*) ______________________ (*signature*)

**INITIAL** ☐

| | | | |
| --- | --- | --- | --- |
| **Active** | Yes ☐ | No ☐ | Consent date:<br><br>Main Consent Form Version Date: |
| **Withdrawn** | Yes ☐ | No ☐ | If yes, date: |
| **Lost to Follow-up** | Yes ☐ | No ☐ | If yes, date of last contact (phone, email, or in-person): |
| **Deceased** | Yes ☐ | No ☐ | If yes, date: |

| | | | | |
| --- | --- | --- | --- | --- |
| **Cause of Death:** | Related to ALS ☐ | Not related to ALS ☐ | Unknown ☐ | |
| **Death was due to MAiD** | Yes ☐ | No ☐ | Unknown ☐ | N/A ☐ |
| **List cause of death, if known:** | | | | |

| | | |
| --- | --- | --- |
| **Participant is interested in receiving emails about optional participant engagement activities** | Yes ☐ | No ☐ |
| **Participant wants to be contacted for further studies related to CAPTURE ALS** | Yes ☐ | No ☐ |

**PATIENTS ONLY**

| | | | |
| --- | --- | --- | --- |
| **PEG insertion** | Yes ☐ | No ☐ | If yes, date: |
| **BiPAP start** | Yes ☐ | No ☐ | If yes, date: |
| **PAV >22hrs/day***<br>*Permanently Assisted Ventilation | Yes ☐ | No ☐ | If yes, date: |
| **Tracheostomy** | Yes ☐ | No ☐ | If yes, date: |

**NOTES**
