# Supplementary material for: Comprehensive analysis platform to understand, remedy, and eliminate amyotrophic lateral sclerosis (CAPTURE ALS): Study protocol for a Canadian multicenter, multimodal, longitudinal observational study
Source: PLoS One. 2025 Dec 4;20(12):e0332430. doi: 10.1371/journal.pone.0332430 (PMC12677780; doi:10.1371/journal.pone.0332430)
Supplement: S3 Appendix — (PDF) [file pone.0332430.s003.pdf]

ID: *CAPT*	Visit: 1 (*SCREENING)*
Date:	

Medical Evaluations Form

Date performed: ___________________

Information collected by: ________________________ ________________________
name *signature*

CURRENT MEDICATIONS			INITIAL ☐
**Riluzole**	Yes ☐	No ☐	If yes, start date (YYYY-MM-DD):
**Edaravone**	Yes ☐	No ☐	If yes, start date (YYYY-MM-DD):
**AMX0035**	Yes ☐	No ☐	If yes, start date (YYYY-MM-DD):
**Anticonvulsants**	Yes ☐	No ☐	If yes, list medication and dose:
**Psychiatric medications**	Yes ☐	No ☐	If yes, list medication and dose:
**Benzodiazepines**	Yes ☐	No ☐	If yes, list medication and dose:
**Opiates**	Yes ☐	No ☐	If yes, list medication and dose:
**Baclofen**	Yes ☐	No ☐	If yes, list medication and dose:
**Other medications (and dose if known):**			

Current alcohol consumption (average number of drinks per week):

1 Drink is:
1 Beer (341ml/12 oz; 5% ABV)
1 glass of wine (142 ml/5 oz; 12% ABV)
1 distilled alcohol (43 ml/1.5 oz; 40% ABV)

Smoker: Yes ☐ No ☐ Former ☐

If yes or former:

Number of packages of cigarettes (where one pack contains 20) smoked daily: _____ packs Unknown ☐

Number of years smoking: _____ year(s)

Year of cessation:___________

Recreational drug use (type and approximate quantity per week):

Is participant currently in a clinical trial? Yes ☐ No ☐

If yes, which ones and start date (YYYY-MM-DD):

MEDICAL CONDITIONS / HISTORY INITIAL ☐

Has the participant been hit in the head hard enough to cause a loss of consciousness? Yes ☐ No ☐

If yes,

Did they visit the Emergency Room? Yes ☐ No ☐

Were they hospitalized? Yes ☐ No ☐

Stroke Yes ☐ No ☐

Cognitive/learning issues Yes ☐ No ☐

**Epilepsy**	Yes ☐	No ☐
**Other neurological conditions:**<br>If yes, specify condition:	Yes ☐	No ☐
**Depression***<br>**Defined as formal clinical diagnosis or use of antidepressant medication for the purpose of depression in the present or past.*	Yes ☐	No ☐
**Other psychiatric conditions**<br>If yes, specify condition:	Yes ☐	No ☐
**Malignancy**<br>If yes, specify type, year, and if chemo or radiation were used:	Yes ☐	No ☐
**Diabetes**	Yes ☐	No ☐
**Hypertension**	Yes ☐	No ☐
**Other significant medical conditions**<br>If yes, specify:	Yes ☐	No ☐
**Notes:**		
