# Supplementary material for: Comprehensive analysis platform to understand, remedy, and eliminate amyotrophic lateral sclerosis (CAPTURE ALS): Study protocol for a Canadian multicenter, multimodal, longitudinal observational study
Source: PLoS One. 2025 Dec 4;20(12):e0332430. doi: 10.1371/journal.pone.0332430 (PMC12677780; doi:10.1371/journal.pone.0332430)
Supplement: S4 Appendix — (PDF) [file pone.0332430.s004.pdf]

S4 Appendix. Family History and Genetic Testing Form.

CAPTURE ALS CAPTURE SLA

CAPTURE ALS

Family History and Genetic Testing Form

ID: ***CAPT*** **__ __ __ __ __ __ __ __** **Visit: 2 (month 0)**

Date performed: ________________

Information collected by: ____________________ ____________________

name *signature*

FAMILY HISTORY **INITIAL ☐** **NO CHANGE ☐** **UPDATE ☐**

Are you adopted? Yes ☐ No ☐

Are your parents, cousins, or otherwise biologically related to each other? Yes ☐ No ☐

***Has anyone in your family ever been diagnosed with ALS or FTD?** Yes ☐ No ☐

***Has anyone in your family ever been diagnosed with dementia, psychiatric illness, other motor neuron diseases or other neurological disorders?** Yes ☐ No ☐

**If Yes, complete the following table. [e.g. 3 siblings, 2 affected, diagnosis ALS (sibling 1), diagnosis FTD (sibling 2)]*
**If No to both questions, do not complete the table.*

**Relative**	**Number of Family Members**	**Number of Affected Family Members**	**Diagnosis**
Mother	1	______	☐ Amyotrophic Lateral Sclerosis (ALS)<br>☐ Frontotemporal Dementia (FTD)<br>☐ Dementia (specify)<br>☐ Psychiatric illness (specify)<br>☐ Other Motor Neuron Disease (specify)<br>☐ Other Neurological disorders (specify)
Father	1	______	☐ Amyotrophic Lateral Sclerosis (ALS)<br>☐ Frontotemporal Dementia (FTD)<br>☐ Dementia (specify)<br>☐ Psychiatric illness (specify)<br>☐ Other Motor Neuron Disease (specify)<br>☐ Other Neurological disorders (specify)
Siblings	______	______	☐ Amyotrophic Lateral Sclerosis (ALS)<br>☐ Frontotemporal Dementia (FTD)<br>☐ Dementia (specify)<br>☐ Psychiatric illness (specify)<br>☐ Other Motor Neuron Disease (specify)<br>☐ Other Neurological disorders (specify)
Half Siblings	______	______	☐ Amyotrophic Lateral Sclerosis (ALS)<br>☐ Frontotemporal Dementia (FTD)<br>☐ Dementia (specify)<br>☐ Psychiatric illness (specify)<br>☐ Other Motor Neuron Disease (specify)<br>☐ Other Neurological disorders (specify)
Maternal Grandmother	1	______	☐ Amyotrophic Lateral Sclerosis (ALS)<br>☐ Frontotemporal Dementia (FTD)<br>☐ Dementia (specify)<br>☐ Psychiatric illness (specify)<br>☐ Other Motor Neuron Disease (specify)<br>☐ Other Neurological disorders (specify)

Maternal Grandfather	1	______	☐ Amyotrophic Lateral Sclerosis (ALS)<br>☐ Frontotemporal Dementia (FTD)<br>☐ Dementia (specify)<br>☐ Psychiatric illness (specify)<br>☐ Other Motor Neuron Disease (specify)<br>☐ Other Neurological disorders (specify)
Paternal Grandmother	1	______	☐ Amyotrophic Lateral Sclerosis (ALS)<br>☐ Frontotemporal Dementia (FTD)<br>☐ Dementia (specify)<br>☐ Psychiatric illness (specify)<br>☐ Other Motor Neuron Disease (specify)<br>☐ Other Neurological disorders (specify)
Paternal Grandfather	1	______	☐ Amyotrophic Lateral Sclerosis (ALS)<br>☐ Frontotemporal Dementia (FTD)<br>☐ Dementia (specify)<br>☐ Psychiatric illness (specify)<br>☐ Other Motor Neuron Disease (specify)<br>☐ Other Neurological disorders (specify)
Maternal Aunt	______	______	☐ Amyotrophic Lateral Sclerosis (ALS)<br>☐ Frontotemporal Dementia (FTD)<br>☐ Dementia (specify)<br>☐ Psychiatric illness (specify)<br>☐ Other Motor Neuron Disease (specify)<br>☐ Other Neurological disorders (specify)
Maternal Uncle	______	______	☐ Amyotrophic Lateral Sclerosis (ALS)<br>☐ Frontotemporal Dementia (FTD)<br>☐ Dementia (specify)<br>☐ Psychiatric illness (specify)<br>☐ Other Motor Neuron Disease (specify)<br>☐ Other Neurological disorders (specify)
Paternal Aunt	______	______	☐ Amyotrophic Lateral Sclerosis (ALS)<br>☐ Frontotemporal Dementia (FTD)<br>☐ Dementia (specify)<br>☐ Psychiatric illness (specify)<br>☐ Other Motor Neuron Disease (specify)<br>☐ Other Neurological disorders (specify)
Paternal Uncle	______	______	☐ Amyotrophic Lateral Sclerosis (ALS)<br>☐ Frontotemporal Dementia (FTD)<br>☐ Dementia (specify)<br>☐ Psychiatric illness (specify)<br>☐ Other Motor Neuron Disease (specify)<br>☐ Other Neurological disorders (specify)
Maternal Cousin	______	______	☐ Amyotrophic Lateral Sclerosis (ALS)<br>☐ Frontotemporal Dementia (FTD)<br>☐ Dementia (specify)<br>☐ Psychiatric illness (specify)<br>☐ Other Motor Neuron Disease (specify)<br>☐ Other Neurological disorders (specify)

Paternal Cousin	______	______	☐ Amyotrophic Lateral Sclerosis (ALS)<br>☐ Frontotemporal Dementia (FTD)<br>☐ Dementia (specify)<br>☐ Psychiatric illness (specify)<br>☐ Other Motor Neuron Disease (specify)<br>☐ Other Neurological disorders (specify)
Children	______	______	☐ Amyotrophic Lateral Sclerosis (ALS)<br>☐ Frontotemporal Dementia (FTD)<br>☐ Dementia (specify)<br>☐ Psychiatric illness (specify)<br>☐ Other Motor Neuron Disease (specify)<br>☐ Other Neurological disorders (specify)
Other, specify:	______	______	☐ Amyotrophic Lateral Sclerosis (ALS)<br>☐ Frontotemporal Dementia (FTD)<br>☐ Dementia (specify)<br>☐ Psychiatric illness (specify)<br>☐ Other Motor Neuron Disease (specify)<br>☐ Other Neurological disorders (specify)

Notes:

GENETIC TESTING OF PARTICIPANT (from medical records) **DONE ☐** **NOT DONE ☐**

Date of test (MM-DD-YYYY):

Was a mutation found? Yes ☐ No ☐ Unknown ☐

Which gene(s) were involved?

☐ ANG	☐ ANXA11	☐ APP	☐ ARHGEH28	☐ ATXN2
☐ C9orf72	☐ CFAP410	☐ CHCHD10	☐ CHMP2B	☐ DAO
☐ DCTN1	☐ ERBB4	☐ FIG4	☐ FUS	☐ GRN
☐ HNRNPA1	☐ HNRNPA2B1	☐ ITM2B	☐ KIF5A	☐ MAPT
☐ MOBP	☐ NEFH	☐ NEK1	☐ OPTN	☐ PFN1
☐ PSEN1	☐ PSEN2	☐ SETX	☐ SOD1	☐ SQSTM1
☐ TAF15	☐ TARDBP	☐ TBK1	☐ TREM2	☐ TUBA4A
☐ UBQLN2	☐ UNC13A	☐ VAPB	☐ VCP	☐ Other

If other, please state: ____________________________________

Mutation identified or repeat length:

Laboratory name:

Notes:
