# Supplementary material for: Comprehensive analysis platform to understand, remedy, and eliminate amyotrophic lateral sclerosis (CAPTURE ALS): Study protocol for a Canadian multicenter, multimodal, longitudinal observational study
Source: PLoS One. 2025 Dec 4;20(12):e0332430. doi: 10.1371/journal.pone.0332430 (PMC12677780; doi:10.1371/journal.pone.0332430)
Supplement: S5 Appendix — (PDF) [file pone.0332430.s005.pdf]

CAPTURE ALS

ALS FUNCTIONAL RATING SCALE - REVISED (ALSFRS-R) ADMINISTRATION

SOP02EN01

Issue Date: 22-Feb-2022
Version: 1.0

Prepared by:

Claire Magnussen — 22 Feb 2022
Claire Magnussen — Date
Program Manager

Approved by:

22 Feb 2022
Sanjay Kalra MD — Date
CAPTURE ALS National PI

1. SCOPE AND APPLICABILITY

This SOP describes the process for administering the ALS Functional Rating Scale - Revised (ALSFRS-R) to patients participating in CAPTURE ALS. This assessment will not be performed for healthy controls. This SOP may be used at any of the CAPTURE ALS collection sites.

2. SUMMARY OF METHOD

The ALSFRS-R is used to assess change in functional status, across four domains of function and activities of daily living. The scale evaluates function at evaluation compared to prior to onset of ALS symptoms. The scale is administered by a trained evaluator; however, the response is subjective, provided by the patient. The assessment will be administered directly on the source document, and the number beside the most accurate response will be circled. The total score will be tabulated and entered into the database.

3. DEFINITIONS

ALSFRS-R	Amyotrophic Lateral Sclerosis Functional Rating Scale - Revised
BiPAP	Bilevel Positive Airway Pressure
CPAP	Continuous Positive Airway Pressure
MRI	Magnetic Resonance Imaging
NIV	Non-Invasive Ventilation

4. PERSONNEL QUALIFICATIONS/RESPONSIBILITIES

This SOP concerns all personnel (eg, coordinators, investigators) who will be administering the ALSFRS-R.

5. PROCEDURE

5.1 General Procedures

- When an MRI is performed on the same patient, the ALSFRS-R will be performed as close to the MRI scan date as possible. Only ALSFRS-R scores +/- 1 month from the date of MRI examination should be used.
- The preferred method of administration is in person.
- It should be documented if the scale is administered over the telephone. If the patient is unable to respond due to significant bulbar impairment, or if the patient has a significant communication barrier, a caregiver may relay the questions and responses; however the response should be the patient’s response.
- As a general rule, “help” can mean help from a person, device or appliance.
- Cues to help determine the score for each category are provided in *italics*, below.
- It is important for the evaluator to use the probing questions and cues to get the most accurate response from the patient as possible.
- Every time the ALSFRS-R is administered, remind the patient to compare his/her current function with their function **prior to any symptoms of ALS.**

5.2 ALSFRS-R Items

1. Speech

Ask "How are you doing with your speech?" Ask if the patient notes a change in speech. The patient is to compare his/her current function with function prior to any symptoms of ALS.

- □ **4 – Normal speech processes.** *Speech is as it was prior to onset of illness.*
- □ **3 – Detectable speech disturbances**. *Some detectable alteration in speech noticed by patient or carer not attributable to an obvious cause (eg. new dentures).*
- □ **2 – Intelligible with repeating.** *>25% of the time, repeating is necessary for comprehension. Ask who requires repeating (strangers, or those close with the patient as well?). Query about repetition in-person vs. on the telephone.*
- □ **1 – Speech combined with non-vocal communication**. *Writing, use of speech synthesizers, communication via phone/tablet, or similar methods are needed to supplement speech.*
- □ **0 – Loss of useful speech.** *Impossible for the person to communicate verbally.*

2. Salivation

Ask "How are you doing with your saliva?" Rate current status versus prior to ALS onset regardless of whether patient is taking medication for salivation.

- □ **4 – Normal.** *No excess saliva, patient reports normal or dry mouth. If patient starts taking medication for sialorrhea, their response to this item may return from a lower score to "4".*
- □ **3 – Slight but definite excess of saliva in mouth; may have nighttime drooling**. *There is an excess, but there is usually no need to mop up the saliva with a tissue*
- □ **2 – Moderately excessive saliva; may have minimal drooling.** *A tissue needs to be used*.
- □ **1 – Marked excess of saliva with some drooling.** *Likely to be drooling and a tissue is often, but not always used.*
- □ **0 – Marked drooling; *requires constant tissue or handkerchief.*** *or suction.*

3. Swallowing

Ask "How are you doing with your swallowing?"

- □ **4 – Normal eating habits.** *No change from before symptom onset, able to eat any food in typical mouthful sizes or drink liquid without difficulty.*
- □ **3 – Early eating problems – occasional choking.** *Occasionally food will stick or causes coughing or choking. Food may need to be cut up small but is not mashed or liquidized.*

- ☐ ***2* – Dietary consistency changes.** *Food needs to be mashed or liquified, drinks may need thickener, some foods avoided. Presence of choking is not required to score "2".*
- ☐ **1 – Needs supplemental tube feeding.** *% use of feeding tube/caloric intake not specified. Be consistent in your own rating.*
- ☐ **0 – NPO (exclusively parenteral or enteral feeding**).

4. Handwriting

Ask "Are you able to hold a pen?" If yes, then ask "How are you doing with your writing? And explore whether words are legible. Only score the dominant hand and only score for use of a standard pen of normal size. If circumstances allow, you may give the patient pen and paper, and ask them to write something (we don't often write anymore on a daily basis). If this is not possible, or if the patient has not written other words except their name or signature recently and therefore cannot answer the question further, score 1.

- ☐ **4 – Normal.**
- ☐ **3 – Slow or sloppy; all words are legible.** *Using a normal pen there is a change in writing, person may need to use large pen grips or other writing aids. It is important to explain to the patient that you are querying them on their writing under ideal circumstances (with a solid surface, and the right height/angle, etc.).*
- ☐ **2 – Not all words are legible.** *Some words, but not others, are legible. Ignore ability to write name or sign legibly.*
- ☐ **1 – No words are legible, but can still grip pen.** *If patient can only write their name or sign legibly, but other writing is illegible.*
- ☐ **0 – Unable to grip pen.**

5a. Cutting food and handling utensils (patients without gastrostomy)

If someone has a gastrostomy, but it is not the primary method of caloric intake (<50%), treat as without gastrostomy. Ask "How are you doing with cutting food and handling cutlery?"

- ☐ **4 – Normal.** *No change compared with before symptom onset and no change in the type of utensil used.*
- ☐ **3 – Somewhat slow and clumsy, but no help needed.** *Some difficulty either cutting food or holding utensils, but able to do this independently. To help distinguish between "2" and "3", ask the patient about ability to cut a tough steak, or perform meal preparation (repeated cutting/chopping).*
- ☐ **2 – Can cut most foods, although clumsy and slow; some help needed.** *Occasionally assistance is needed (<50%), but patient is independent for the task otherwise. Use of large handled, or adapted, cutlery to achieve the task should be scored as "2".*
- ☐ **1 – Food must be cut by someone, but can still feed slowly.** *Assistance is required >50% of the time for cutting, but not for feeding.*

- □ **0 – Needs to be fed.** *Assistance is needed for any aspect of the task to be achieved. If someone decides not to cut food or feed themselves but otherwise might be able to, score 0.*

5b. Cutting food and handling utensils (patients with gastrostomy)

If someone has a gastrostomy and it is the primary method of caloric intake (>50% nutrition), treat as with gastrostomy. Ask "How are you doing with handling the gastrostomy fastenings and fixtures?"

- □ **4 – Normal.** *No difficulty at all with any manipulations.*
- □ **3 – Clumsy but able to perform all manipulations independently.**
- □ **2 – Some help needed with closures and fasteners.**
- □ **1 – Provides minimal assistance to caregiver.**
- □ **0 – Unable to perform any aspect of task.**

6. Dressing and Hygiene

Ask "How are you doing with dressing and hygiene?" If someone decides not to dress or bathe themselves but would otherwise be able to, score 0.

- □ **4 – Normal function.** *No change compared with before symptom onset.*
- □ **3 – Independent and complete self-care with effort or decreased efficiency.** *Patient is slower than before but remains independent, and does not use any assistance from either another person or a device (eg. button hook).*
- □ **2 – Intermittent assistance or substitute methods.** *Some help is needed either from a caregiver or by use of devices such as button hooks or self-tying laces, but the patient is otherwise independent. If they have changed the clothing they normally wear (eg. zipped clothing instead of buttons), score as substitute method. If they have changed the method they use (eg. sitting down to put on socks), score as substitute method.*
- □ **1 – Needs attendant for self-care.** *All aspects of the task require assistance, but the patient is able to assist the caregiver for much of it.*
- □ **0 – Total dependence.** *Completely unable to carry out any aspect of the task and cannot significantly help the caregiver.*

7. Turning in Bed and Adjusting Bed Clothes

Ask "How are you doing with turning in bed and adjusting the bed sheets?"

- □ **4 – Normal.**
- □ **3 – Somewhat slow and clumsy, but no help needed.** *Can perform both activities independently, but there may be difficulty with either or both activities*
- □ **2 – Can turn alone or adjust sheets, but with great difficulty.** *Patient can perform <u>at least one</u> of the activities independently, but there is great*

difficulty. Distinguishing between “2” and “3” can be challenging – a score of “2” is a true struggle for the patient.

- □ **1 – Can initiate, but not turn or adjust sheets alone**. *The process of turning is begun in some way by the person, but someone else needs to provide the assistance required to complete the task. If one task can be completed independently, score 2. If both tasks require assistance to complete, score 1.*
- □ **0 – Helpless.** *Initiation of turning is impossible.*

8. Walking

Ask “How are you doing with your walking?”

- □ **4 – Normal.** *No change from walking ability before symptom onset*
- □ **3 – Early ambulation difficulties.** *Some difficulty walking (eg. slow, tripping, or imbalance), but no assistance is routinely needed from someone else, or a physical aid (eg. ankle-foot orthosis, walking stick, frame).*
- □ **2 – Walks with assistance.** *Assistance is required from a physical aid (ankle-foot arthosis, cane, etc.) or caregiver.*
- □ **1 – Non-ambulatory functional movement.** *Patient cannot walk, but can help with transfers by weight bearing.*
- □ **0 – No purposeful leg movement.** *If the patient still has foot control, but cannot bear weight, score as “0”.*

9. Climbing Stairs

Ask “How are you doing with climbing stairs?” Only rate ability for walking up stairs, not down. If patient is unsure (eg. because they do not have any stairs), can provide example of going up 3-4 stairs to enter a store/restaurant/etc. If someone decides they do not want to climb stairs but would seem otherwise able, score 0. This item can change most rapidly, and distinguishing between “2” and “3” is challenging. Consistency in probing questions and evaluation is critical.

- □ **4 – Normal.** *No change from ability before symptom onset.*
- □ **3 – Slow.** *Some slowing but the patient does not rest between steps and does not feel unsteady.*
- □ **2 – Mild unsteadiness or fatigue.** *Patient needs to rest between steps, or feels unsteady, but does not need rail.*
- □ **1 – Needs assistance.** *Patient needs assistance including handrail or caregiver. If patient uses the handrail, important to ask if they used the handrail consistently prior to symptoms of ALS.*
- □ **0 – Cannot do.**

10. Dyspnea

First, query the patient to confirm if they have any pre-existing respiratory conditions (eg. asthma, seasonal allergies affecting respiratory function, chronic obstructive pulmonary

disease). If yes, provide the patient with another reminder that they are comparing this function with prior to their symptoms of ALS.

Ask "How are you doing with your breathing?" Score the patient regardless of the apparent cause of breathlessness. If the patient is using some form of assisted ventilation, the patient should rate this symptom when they are not using it. "Walking" means walking at a comfortable speed on the flat. Some evaluators find it helpful to start probing from the bottom of the scale (eg. do you experience any breathlessness when sitting, watching TV?), and moving up the scale.

- □ **4 – None.**
- □ **3 – Occurs when walking.**
- □ **2 – Occurs with one or more of the following: eating, bathing, dressing**. *Can also probe about speaking for >10 min in conversation, as well as potential breathlessness when emotional.*
- □ **1 – Occurs at rest, difficulty breathing when either sitting or lying.**
- □ **0 – Significant difficulty, considering using mechanical respiratory support.**

11. Orthopnea

If patient's medical history notes use of non-invasive ventilation (NIV) prior to onset of ALS, for a diagnosed condition such as sleep apnea, then query the patient on whether any of their NIV machine settings have been altered since diagnosis of ALS. This could indicate that respiratory function has changed. If patient was diagnosed with sleep apnea and NIV was initiated close to onset of ALS symptoms, consult with neurologist, and if available, respirologist, to determine if it is possible that it was misdiagnosis of ALS-related orthopnea.

Ask "How is your breathing when sleeping?" Score based on difficulty regardless of the apparent underlying cause (eg, needing to sleep sitting up because of excessive saliva scores "1"). Query about use of type of bed, and treat a hospital style bed in which the back can be raised independently as if pillows were in place of the raised section.

- □ **4 – None.**
- □ **3 – Some difficulty sleeping at night due to shortness of breath, does not routinely use more than two pillows.** *No difficulty falling asleep, but they do not use more than two pillows, score 3.*
- □ **2 – Needs extra pillows in order to sleep (more than two).** *More than two pillows are needed (or bed back raised up at least 45 degrees). Query if they have always used more than 2 pillows.*
- □ **1 – Can only sleep sitting up.** *Patient sleeps sitting up in bed or in a chair.*
- □ **0 – Unable to sleep without mechanical assistance.** *NIV used most or all of the night. If NIV is used for an hour or so only, score as if not used.*

12. Respiratory Insufficiency

Ask "Do you use non-invasive ventilation (NIV)?" Regard BiPAP as any form of NIV including BiPaP or CPAP. If NIV (eg. CPAP) was used prior to ALS onset, for another

diagnosed condition (eg. sleep apnea), and there have been no changes to the machine's settings, score 4.

- □ **4 – None.**
- □ **3 – Intermittent use of BiPaP.**
- □ **2 – Continuous use of BiPaP during the night**.
- □ **1 – Continuous use of BiPaP during the night and day.**
- □ **0 – Invasive ventilation by intubation or tracheostomy.**

6. DATA/RECORDS MANAGEMENT

The ALSFRS-R will be administered directly on the source document, and the number beside the most accurate response will be circled.

7. REFERENCES

This SOP is adapted from the ENCALS SOP. Note the key differences between the two are on the scoring of Items 3, 10, 11, and 12.

ENCALS SOP for ALSFRS-R administration V1.2, 10 May 2015
https://www.encals.eu/wp-content/uploads/2016/09/ENCALS-SOP-for-ALSFRS-R-v1.2.pdf

8. APPENDICES

ALSFRS-R Form (EN, Version 2, 03-Jan-2022)
ALSFRS-R Form (FR, Version 2, 03-Jan-2022)
