# Supplementary material for: Comprehensive analysis platform to understand, remedy, and eliminate amyotrophic lateral sclerosis (CAPTURE ALS): Study protocol for a Canadian multicenter, multimodal, longitudinal observational study
Source: PLoS One. 2025 Dec 4;20(12):e0332430. doi: 10.1371/journal.pone.0332430 (PMC12677780; doi:10.1371/journal.pone.0332430)
Supplement: S6 Appendix — (PDF) [file pone.0332430.s006.pdf]

**ID:** *CAPT*	**Visit: 2 (month 0)**
**Date:**	

Vital Capacity

**Assessment performed by:**	______________________ *name*		______________________ *signature*	
☐ **Forced Vital Capacity**<br>☐ **Slow Vital Capacity**<br>☐ **Not Done**				
**Date of test (MM-DD-YYYY):**				
**Results obtained from:**	☐ medical records		☐ protocol specific assessment	
**Position for vital capacity:**	supine ☐	upright ☐	not reported ☐	
**Best trial (L):**				
**Best trial (% predicted):**				
**Height:**	___________ cm / inch *(circle unit)*			
**Weight:**	___________ kg / lbs *(circle unit)*			
**Breathing conditions:**	None ☐	Asthma ☐	Possible Asthma ☐	COPD ☐
	Pneumonia ☐	Other ☐		
	If other, state:			
**FVC reliability:**	Reliable ☐	Unreliable ☐	Not reported ☐	
**Notes:**				
