# Supplementary material for: Comprehensive analysis platform to understand, remedy, and eliminate amyotrophic lateral sclerosis (CAPTURE ALS): Study protocol for a Canadian multicenter, multimodal, longitudinal observational study
Source: PLoS One. 2025 Dec 4;20(12):e0332430. doi: 10.1371/journal.pone.0332430 (PMC12677780; doi:10.1371/journal.pone.0332430)
Supplement: S7 Appendix — (PDF) [file pone.0332430.s007.pdf]

CAPTURE ALS

ID: *CAPT*	Visit: 2 (month 0)
Date:	

Neurological Evaluations Form (Visit Month-0)

TAPPING	NOT DONE ☐
**Date performed:**	
**Assessments performed by:** ______________ *name* ______________ *signature*	

	Trial #	Right	Left
Fingers	1		
	2		
Feet	1		
	2		

ENTER "0" IF CAN'T DO BECAUSE OF PROGRESSION OF ALS.

ENTER "UNABLE" IF CAN'T DO BECAUSE OF WEAKNESS DUE TO ANY OTHER CONFOUNDING ILLNESS/INJURY.

SACCADES / ANTISACCADES	NOT DONE ☐
**Date performed:**	
**Assessments performed by:** ______________ *name* ______________ *signature*	
**# Correct Saccades (/8):**	
**# Correct Antisaccades (/8):**	

ID: *CAPT*	Visit: 2 (month 0)
Date:	

Neurological Exam (Physician to Complete)

Date performed: ___________________

Neurological Exam performed by: ________________________ (*name*) ________________________ (*signature*)

FACE				NOT DONE ☐
**Fasciculations**	Yes		No	(Circle one)
	**Right**		**Left**	
**Weakness**	Yes No		Yes No	(Circle one)
**TONGUE**				**NOT DONE ☐**
**Fasciculations**	Yes		No	(Circle one)
**Atrophy**	Yes		No	(Circle one)
**Weakness**	Yes		No	(Circle one)
**Movements**	Normal	Slow	No movement	(Circle one)
**DYSARTHIA**				**NOT DONE ☐**
**Dysarthria**	______			0 = None<br>1 = Mild<br>2 = Moderate<br>3 = Severe (unintelligible or anarthric)
**FASCICULATIONS**				**NOT DONE ☐**
**Trunk**	Yes		No	(Circle one)
	**Right**		**Left**	
**Arm**	Yes No		Yes No	(Circle one)
**Leg**	Yes No		Yes No	(Circle one)
**ATROPHY**				**NOT DONE ☐**
	**Right**		**Left**	(Circle one)
**Arm**	Yes No		Yes No	(Circle one)
**Leg**	Yes No		Yes No	(Circle one)

ID: *CAPT*	Visit: 2 (month 0)
Date:	

SPASTICITY		NOT DONE ☐	Modified Ashworth Scale
**Upper Extremities**	**Right**	**Left**	0 = No increase in tone<br>1 = Slight increase: catch and release OR minimal resistance at end of ROM<br>1+ = Slight increase: catch and release followed by minimal resistance throughout the remainder (<½) of ROM<br>2 = Moderate increase in tone, but passive movement easy<br>3 = Marked increase in tone, passive movement difficult<br>4 = Rigid and immobile
(Forearm Supination / Pronation)	____	____	
**Lower Extremities**	**Right**	**Left**	
(Knee Flexion)	____	____	

POWER		NOT DONE ☐
**Neck Extension**	____	0 = No contraction<br>1 = Flicker or trace of contraction<br>2 = Active movement without gravity<br>3 = Active movement against gravity<br>4- = Active movement against minimal resistance<br>4 = Active movement against moderate resistance<br>4+ = Active movement against strong resistance<br>5 = Normal power
**Neck Flexion**	____	

	Right	Left		Right	eft
**Infraspinatus**	____	____	**Iliopsoas**	____	_ _
**Deltoids**	____	_ _	**Hip Abductors**	____	_ _
**Biceps**	____	_ _	**Quadriceps**	____	_ _
**Triceps**	____	_ _	**Hamstrings**	____	_ _
**Wrist Extensors**	____	_ _	**Ankle Dorsiflexors**	____	_ _
**Wrist Flexors**	____	_ _	**Ankle Plantarflexors**	____	_ _
**EDC**	____	_ _	**EHL**	____	_ _
**FDI**	____	_ _			
**ADM**	____	_ _			
**APB**	____	_ _			

MUSCLE STRETCH REFLEXES			NOT DONE ☐
**Jaw Jerk**	____		0 = Absent<br>1 = Present<br>2 = Clonus
	**Right**	**Left**	
**Biceps**	______	______	0 = Absent<br>1 = Decreased, or seen only with reinforcement<br>2 = Normal<br>3 = Brisk, may have spread<br>4 = Brisk with clonus
**Brachioradialis**	______	______	
**Triceps**	______	______	
**Quadriceps**	______	______	
**Triceps Surae**	______	______	

OTHER REFLEXES			NOT DONE ☐
	Right	Left	
**Hoffman's**	______	______	0 = Absent<br>1 = Present<br>5 = Not done
**Superficial abdominal**	______	_____	
**Babinski**	______	_____	

PSEUDOBULBAR AFFECT			NOT DONE ☐
	Yes	No	(Circle one)

CEREBELLAR DYSFUNCTION			NOT DONE ☐
	**Right**	**Left**	
**Arm (finger – nose ataxia)**	Yes No Unable	Yes No Unable	(Circle one)
**Leg (heel – shin ataxia)**	Yes No Unable	Yes No Unable	(Circle one)

Unable = too weak for reliable assessment

GAIT	NOT DONE ☐
☐ Normal<br>☐ Abnormal<br>☐ Abnormal-Spastic<br>☐ Abnormal-Ataxic<br>☐ Unable to walk without aids	(Select one)

ID: *CAPT*	Visit: 2 (month 0)
Date:	

Sites of ALS Involvement - Based on Clinical Exam NOT DONE ☐

Clinical Region	UMN		LMN	
Bulbar	Yes<br>No<br>Not done	☐<br>☐<br>☐	Yes<br>No<br>Not done	☐<br>☐<br>☐
Cervical	Yes<br>No<br>Not done	☐<br>☐<br>☐	Yes<br>No<br>Not done	☐<br>☐<br>☐
Thoracic	Yes<br>No<br>Not done	☐<br>☐<br>☐	Yes<br>No<br>Not done	☐<br>☐<br>☐
Lumbar	Yes<br>No<br>Not done	☐<br>☐<br>☐	Yes<br>No<br>Not done	☐<br>☐<br>☐

Sites of ALS Involvement – Based on EMG

Was there an EMG done before this visit? No ☐ Yes ☐

Date of EMG: _______________

Clinical Region	EMG evidence of active denervation	
Bulbar	Yes<br>No<br>Not done	☐<br>☐<br>☐
Cervical	Yes<br>No<br>Not done	☐<br>☐<br>☐
Thoracic	Yes<br>No<br>Not done	☐<br>☐<br>☐
Lumbar	Yes<br>No<br>Not done	☐<br>☐<br>☐

DIAGNOSIS

ALS ☐ ALS-FTD ☐ FTD ☐ PLS ☐ PMA ☐ MSA ☐

HSP ☐ Kennedy's ☐ Spinocerebellar Ataxia ☐ Other ☐

If other, specify:

EL ESCORIAL CRITERIA [If diagnosis is ALS or ALS-FTD]

Suspected ☐ Possible ☐ Probable ☐ Probable lab supported ☐ Definite ☐ N/A ☐

Notes:

Neurological Exam performed by: ______________________________

Physician's name

____

Signature

____

Date
