# Supplementary material for: Comprehensive analysis platform to understand, remedy, and eliminate amyotrophic lateral sclerosis (CAPTURE ALS): Study protocol for a Canadian multicenter, multimodal, longitudinal observational study
Source: PLoS One. 2025 Dec 4;20(12):e0332430. doi: 10.1371/journal.pone.0332430 (PMC12677780; doi:10.1371/journal.pone.0332430)
Supplement: S8 Appendix — (PDF) [file pone.0332430.s008.pdf]

CAPTURE ALS

NEUROCOGNITVE TESTING
and
PATIENT REPORTED OUTCOME MEASURES

SOP03EN01

Issue Date: 08-March-2022
Version: 1.0

Prepared by:

Claire Magnussen<br>Program Manager	8 March 2022<br>Date

Approved by:

Sanjay Kalra MD<br>CAPTURE ALS National PI	8 March 2022<br>Date

TABLE OF CONTENTS

1. SCOPE AND APPLICABILITY 6
2. SUMMARY OF METHOD 6
3. DEFINITIONS 6
4. PERSONNEL QUALIFICATIONS/RESPONSIBILITIES 6
5. EQUIPMENT AND SUPPLIES 6
6. PROCEDURE 7
6.1. General Procedures 7
6.1.1. Prior to each visit 7
6.1.2. During testing 7
6.1.3. After testing 7
6.2 Order of Assessments 8
6.3 Schedule of Alternate Forms 9
6.4. Montreal Cognitive Assessment (MoCA) 9
6.5. Edinburgh Cognitive and Behavioural ALS Screen (ECAS) 10
6.6. ECAS Behavior Screen – Caregiver Interview 10
6.7. Semantic Fluency + Abrahams Correction 10
6.8. Boston Naming Test-II (BNT-II) 11
6.9. Hopkins Verbal Learning Test – Revised (HVLT-R) 11
6.10. Hopkins Verbal Learning Test – Revised: Delayed Recall Trial 11
6.11. Social Norms Questionnaire 12
6.12. Hospital Anxiety and Depression Scale (HADS) 12
6.13. Center for Neurologic Study – Lability Scale (CNS-LS) 12
6.14. Frontal Systems Behaviour Scale (FrSBe) Self-Rating Form 12
6.15. Frontal Systems Rating Behavior Scale (FrSBe) Family-Rating Form 13
6.16. Stroop Test (Victoria version) 13
6.17. Judgment of Line Orientation (JLO) 13
6.18. Amyotrophic Lateral Sclerosis Assessment Questionnaire Short Form (ALSAQ-5) 13
6.19. World Health Organization Quality of Life-BREF Scale (WHOQOL-BREF) 14
7. DATA AND RECORDS MANAGEMENT 14
8. REFERENCES 15

9. APPENDICES 15
Appendix 9.1 Montreal Cognitive Assessment (MoCA) 16
Appendix 9.1.1: MoCA [ENGLISH] 17
Appendix 9.1.2: MoCA Administration/Scoring Guidelines [ENGLISH] 18
Appendix 9.1.3: MoCA [FRENCH] 23
Appendix 9.1.4: MoCA modifications CLOCK 24
Appendix 9.1.5: MoCA modifications CUBE 25
Appendix 9.2: Edinburgh Cognitive and Behavioral ALS Screen (ECAS) 26
Appendix 9.2.1: ECAS A [ENGLISH] 27
Appendix 9.2.2: ECAS A [FRENCH] 37
Appendix 9.2.3: ECAS B [ENGLISH] 47
Appendix 9.2.4: ECAS B [FRENCH] 57
Appendix 9.2.5: ECAS C [ENGLISH] 67
Appendix 9.2.6: ECAS C [FRENCH] 77
Appendix 9.2.7: ECAS Guidance A, B, C [ENGLISH] 87
Appendix 9.2.8: ECAS Guidance A, B, C [FRENCH] 99
Appendix 9.3: Sematic Fluency and Abrahams Correction 129
Appendix 9.3.1: Semantic Fluency [ENGLISH] 130
Appendix 9.3.2: Semantic Fluency [FRENCH] 131
Appendix 9.3.3: Abrahams Correction [ENGLISH] 132
Appendix 9.3.4: Abrahams Correction [FRENCH] 133
Appendix 9.4: Boston Naming Test-II (BNT-II) 134
Appendix 9.4.1: BNT-II Short & Standard Forms [ENGLISH] 135
Appendix 9.4.2: BNT-II Short & Standard Form [FRENCH] 143
Appendix 9.4.3: BNT-II Stimulus picture example 146
Appendix 9.5: Hopkins Verbal Learning Test – Revised (HVLT-R) 147
Appendix 9.5.1: HVLT-R (Form 1) [ENGLISH] 148
Appendix 9.5.2: HVLT-R (Form 2) [ENGLISH] 150
Appendix 9.5.3: HVLT-R (Form 1) [FRENCH] 152
Appendix 9.5.4: HVLT-R (Form 2) [FRENCH] 157
Appendix 9.6: Social Norms Questionnaire 162
Appendix 9.6.1: Social Norms Questionnaire [ENGLISH] 163

Appendix 9.6.2: Social Norms Questionnaire [FRENCH] ... 164
Appendix 9.6.3: Social Norms Questionnaire Scoring Instructions [ENGLISH] ... 165
Appendix 9.6.4: Social Norms Questionnaire Scoring Instructions [FRENCH] ... 166
Appendix 9.7: Hospital Anxiety and Depression Scale (HADS) ... 167
Appendix 9.7.1: HADS [ENGLISH] ... 168
Appendix 9.7.2: HADS [FRENCH] ... 169
Appendix 9.8: Center for Neurological Study-Lability Scale (CNS-LS) ... 170
Appendix 9.8.1: CNS-LS [ENGLISH] ... 171
Appendix 9.8.2: CNS-LS [FRENCH] ... 172
Appendix 9.9: Frontal Systems Behaviour Scale (FrSBe) ... 173
Appendix 9.9.1: FrSBe (Self-Rating form) [ENGLISH] ... 174
Appendix 9.9.2: FrSBe (Self-Rating form) [FRENCH] ... 178
Appendix 9.9.3: FrSBe (Family-Rating form) [ENGLISH] ... 180
Appendix 9.9.4: FrSBe (Family-Rating form) [FRENCH] ... 184
Appendix 9.10: Stroop Test (Victoria version) ... 186
Appendix 9.10.1: Stroop Test (Victoria version) [ENGLISH] ... 187
Appendix 9.10.2: Stroop Test (Victoria version) [FRENCH] ... 188
Appendix 9.10.3: Stroop Test (Victoria version) Manual [ENGLISH] ... 189
Appendix 9.10.4: Stroop Test (Victoria version) stimulus cards [ENGLISH] ... 194
Appendix 9.10.5: Stroop Test (Victoria version) stimulus cards [FRENCH] ... 195
Appendix 9.10.6: Stroop Test (Victoria version) pointing modification ... 197
Appendix 9.11: Judgment of Line Orientation Form (JLO) ... 198
Appendix 9.11.1: JLO example [ENGLISH] ... 199
Appendix 9.11.2: JLO Form V&H [ENGLISH] ... 200
Appendix 9.11.3: JLO instructions [ENGLISH] ... 201
Appendix 9.11.4: JLO instructions [FRENCH] ... 202
Appendix 9.12: Amyotrophic Lateral Sclerosis Assessment Questionnaire Short Form (ALSAQ-5) ... 203
Appendix 9.12.1: ALSAQ-5 [ENGLISH] ... 204
Appendix 9.12.2: ALSAQ-5 [FRENCH] ... 206
Appendix 9.13: World Health Organization Quality of Life-BREF Scale (WHOQOL-BREF) ... 208
Appendix 9.13.1: WHOQOL-BREF [ENGLISH] ... 209

Appendix 9.13.2: WHOQOL-BREF [FRENCH] 213

Appendix 9.14: Neurocognitive Evaluations Form 216

Appendix 9.14.1: Neurocognitive Evaluations Form (SCREENING) [ENGLISH] 217

Appendix 9.14.2: Neurocognitive Evaluations Form (Month 0 and 8) [ENGLISH] 218

Appendix 9.14.3: Neurocognitive Evaluations Form (Month 4 and 12) [ENGLISH] 220

Appendix 9.14.4: Neurocognitive Evaluations Form (Caregiver) (Month 0 and 8) [ENGLISH] 221

Appendix 9.14.5: Neurocognitive Evaluations Form (Caregiver) (Month 4 and 12) [ENGLISH] 222

1. SCOPE AND APPLICABILITY

This SOP describes the processes for administering the neurocognitive tests and patient reported outcome measures (PROMs) in CAPTURE ALS. This SOP may be used at any of the CAPTURE ALS collection sites.

2. SUMMARY OF METHOD

Following signed informed consent, patient and healthy control participants in CAPTURE ALS will complete various written and oral neurocognitive tests to evaluate their cognitive abilities over time and PROMs to evaluate perceptions of their health status. Consented caregivers will complete questionnaires that ask about the behaviour of the patient participant that they care for or know well. The tests are administered or provided by the study coordinator, and the scores are entered into the database.

3. DEFINITIONS

ALSAQ-5	Amyotrophic Lateral Sclerosis Assessment Questionnaire-5
BNT-II	Boston Naming Test-II
CNS-LS	Center for Neurologic Study Lability Scale
ECAS	Edinburgh Cognitive and Behavioural ALS Screen
FrSBe	Frontal Systems Behaviour Scale
HVLT-R	Hopkins Verbal Learning Test-Revised
HADS	Hospital Anxiety and Depression Scale
JLO	Judgement of Line Orientation
MoCA	Montreal Cognitive Assessment
PROMs	Patient-Reported Outcome Measures
REB	Research Ethics Board
SNQ	Social Norms Questionnaire
SOP	Standard Operating Procedure
WHOQOL-BREF	World Health Organization Quality-of-Life

4. PERSONNEL QUALIFICATIONS/RESPONSIBILITIES

This SOP concerns all personnel (e.g. coordinators, investigators) who will be administering the neurocognitive tests to CAPTURE ALS participants. Training must be documented on the study training log.

5. EQUIPMENT AND SUPPLIES

- printed forms and instructions for the various tests (see **9. Appendices**).
- pen / pencils
- stopwatch
- notepad
- calculator

6. PROCEDURE

6.1. General Procedures

6.1.1. Prior to each visit

- Determine if participants' and caregivers' preferred language is English or French and ensure that all relevant forms are available in their preferred language.
- Use only forms that have received local REB approval.
- Each site will be given a HARD-COPY stimulus book for BNT-II, Stroop Test, and JLO. Each English participant will be provided a HARD-COPY BNT-II record booklet. All other forms are to be printed by the coordinator.
- Prepare an addressed & stamped envelope in case it is necessary to send self-administered evaluations home with participants.
- Encourage patient participants to attend with caregivers.
- Remind participants to bring appropriate reading lenses to complete all tests.
- If a patient participant has significant dysarthria/anarthria, bring a notepad for written responses, and if other technology is used to communicate, it should be brought to the study visit.

6.1.2. During testing

- Write the participant's CAPTURE ALS ID number, visit number and date on all original neurocognitive tests (do not complete other demographics).
- Ensure scripts within tests are followed.
- Self-corrections may be allowed for certain tests. See administration guidelines for each questionnaire to determine what is permitted.
- Self-administered tests (e.g. Social Norms Questionnaire, CNS-LS, FrSBe Self-Rating form, FrSBe Family-Rating form, ALSAQ-5, WHOQOL-BREF) can be sent home with participants to reduce visit time. Instruct participants to complete within a week and mail back as soon as possible. Follow up if not received within a month of visit.
- For patient participants with dysarthria/anarthria, tests can be completed by writing responses either on the test booklet or on a separate sheet of paper. If the participant chooses to type responses on a device such as an iPad or Boogie board, the research assistant will have to copy the provided responses from the device screen onto the *Neurocognitive Evaluation Form.*
- For patient participants with severe dysarthria AND severe hand weakness, an electronic device for typing will be helpful and other tests can be done by pointing to letters/numbers written on paper. Contact the Program Manager prior to the first study visit for more details on modified administration of each test.

6.1.3. After testing

- Use the provided hard copy of each assessment during administration, carefully score each assessment on the hard copy, and TRANSFER the required scores into the corresponding section of the *Neurocognitive Evaluation Form*.
- Indicate which form version was used (if applicable), and the date of administration.

6.2 Order of Assessments

If a sedative will be taken to facilitate the MRI, neurocognitive tests and PROMs should be administered *prior* to the sedative being taken.

It is recommended that the assessments be performed in the following order:

Participants

1. Montreal Cognitive Assessment
2. Edinburgh Cognitive and Behavioural ALS Screen (Pages 1-8 of ECAS)
3. Semantic Fluency + Abrahams Correction
4. Boston Naming Test-II
5. Hopkins Verbal Learning Test (Trials 1-3)
6. Social Norms Questionnaire (self-administered)
7. Hospital Anxiety and Depression Scale (self-administered)
8. Center for Neurologic Study Lability Scale (self-administered)
9. FrSBe Self-Rating Form (self-administered, PATIENTS only)
10. Stroop Test Victoria Version
11. Hopkins Verbal Learning Test Delayed Recall (Trial 4)
12. Judgement of Line Orientation, if required

The self-administered PROMs [ALSAQ-5 (PATIENTS only), WHOQOL-BREF] may be performed at any point during the visit.

Caregivers

1. ECAS Behaviour Screen and Psychosis Screen (Pages 9-10 of ECAS)
2. FrSBe Family Rating Form (self-administered)

6.3 Schedule of Alternate Forms

There are alternate versions for several of the tests (MoCA, ECAS, Semantic Fluency, BNT-II, HVLT-R, JLO). Forms/versions will alternate between visits in order to minimize practice effects.

**Patient Participants**					
**Assessments**	**Visit 1 Screen**	**Visit 2 Month 0**	**Visit 3 Month 4**	**Visit 4 Month 8**	**Visit 5 Month 12**
**MoCA**	V8.1				
**ECAS**		Version A	Version B	Version C	Version A
**Semantic Fluency**		Animals	Fruit	Vegetables	Animals
**BNT-II**		Short		Long	
**HVLT-R**		Form 1		Form 2	
**JLO**		Form V		Form V	

**Control Participants**					
**Assessments**	**Visit 1 Screen**	**Visit 2 Month 0**	N/A	**Visit 3 Month 8**	N/A
**MoCA**	V8.1				
**ECAS**		Version A		Version C	
**Semantic Fluency**		Animals		Vegetables	
**BNT-II**		Short		Long	
**HVLT-R**		Form 1		Form 2	
**JLO**		Form V		Form H	

6.4. Montreal Cognitive Assessment (MoCA)

1. The MoCA, Version 8.1, will be administered to patient and healthy control participants at the screening visit [1].
2. Use the provided form and follow directions in the separate administration and scoring guidelines (see **Appendix 9.1**).
3. For patient participants who are unable to draw the cube or clock due to hand weakness, use the provided modification drawings as a multiple choice.
   a. Score 1 point for choosing the correct cube.
   b. Score 3 points for choosing the correct clock (score 2 points for clock with correct numbers only, and 1 for incorrect clock).
   c. If modifications drawings were used, check the "yes" box on the *Neurocognitive Evaluation form.*
4. Note, an additional point is to be added to the total score for participants with ≤12 years of education.

6.5. Edinburgh Cognitive and Behavioural ALS Screen (ECAS)

1. The ECAS will be administered to patient participants at MONTH 0, 4, 8, and 12 with Versions A, B, C & A administered at each visit, respectively [2-4].
2. The ECAS will be administered to healthy control participants at MONTH 0 (version A) and 8 (version C).
3. Use the provided form and follow directions in the separate administration and scoring guidelines (see **Appendix 9.2**).

6.6. ECAS Behavior Screen – Caregiver Interview

1. This is a SELF-ADMINISTERED assessment.
2. Consented caregivers should be given the “Behavioral Screen - Caregiver Interview” and “ALS Psychosis Screen” (last 2 pages of the ECAS) to complete at each visit.
3. Use the provided form and point out instructions to the caregiver located at the top of the form (see **Appendix 9.2).**
4. This test can be sent to the consented caregiver to be completed within a week of the study visit and mailed back to the site study coordinator.
5. For each behavioral category, total the number of “Yes” responses. A score of 0 indicates that all responses are “No”.

6.7. Semantic Fluency + Abrahams Correction

1. Semantic Fluency + Abrahams Correction will be administered to patient participants at MONTH 0, 4, 8, and 12 [5].
   a. ‘Animals’ will be done at MONTH 0.
   b. ‘Fruit’ will be done at MONTH 4.
   c. ‘Vegetables’ will be done at MONTH 8.
   d. ‘Animals’ will be done at MONTH 12.
2. Semantic Fluency + Abrahams Correction will be administered to healthy control participants at MONTH 0 and 8.
   a. ‘Animals’ will be done at MONTH 0.
   b. ‘Vegetables’ will be done at MONTH 8.
3. Use the provided form and follow administration and scoring instructions at the top of the form (see **Appendix 9.3**).
4. Semantic Fluency can be SPOKEN (1 minute is allotted) or WRITTEN (2 minutes allotted).
5. Following Semantic Fluency testing, perform Abrahams Correction on the provided form.
6. Rewriting the participant’s words on the Abrahams Correction form may be necessary to ensure legibility.
   a. Note: if the Abrahams Correction form was not used, make sure to record the **TIME to read/copy aloud** on the Semantic Fluency form.

6.8. Boston Naming Test-II (BNT-II)

1. The BNT-II will be administered to patient and healthy control participants at MONTH 0 and 8 [6].
    a. Short Form (15 pictures) will be done at MONTH 0.
    b. Long Form (60 pictures) will be done at MONTH 8.
2. Use the provided HARD-COPY stimulus picture book (**1 picture book/site**) and ***record booklets*** (use the provided form for French responses, see **Appendix 9.4**).
    a. SHORT FORM & STANDARD FORM are included in each [English] record booklet. Use the same record booklet for both MONTH 0 and 8 test administrations **(*1 record booklet/participant*).**
    b. For French administrations, use the provided form instead of the *record booklet*.
3. Answers will only be scored as CORRECT or INCORRECT. Do not use phonemic cues, semantic cues, or multiple-choice options, and do not time the latency of responses.
4. Do not tell the participants if their answers are correct or incorrect, as pictures from the Short form will be repeated on the Long form.

6.9. Hopkins Verbal Learning Test – Revised (HVLT-R)

1. The HVLT-R will be administered to patient and healthy control participants at MONTH 0 and 8. Trials 1-3 will be performed first [7].
    a. Form 1 will be administered at MONTH 0.
    b. Form 2 will be administered at MONTH 8.
2. Use the provided form and follow administration and scoring instructions at the top of the form (see **Appendix 9.5**).
3. There must be a delay of 20-25 minutes between the learning and the delayed recall trials of the HVLT-R. During this time, please do not perform other verbal memory-related assessments (e.g. BNT-II, ECAS, Semantic Fluency). However, this time can be used to perform other assessments if desired (e.g. Finger/Toe tapping, CNS-LS, Social Norms Questionnaire, FrSBe, FVC, ALSFRS-R, ALSAQ-5, WHOQOL-BREF).
4. Do not inform participants that there will be a delayed recall portion of this test.

6.10. Hopkins Verbal Learning Test – Revised: Delayed Recall Trial

1. Perform this assessment 20-25 minutes following the HVLT-R learning trial.
2. This is the Trial 4 on the HVLT forms as well as the Delayed Recognition section.

6.11. Social Norms Questionnaire

1. This is a SELF-ADMINISTERED assessment.
2. It will be given to patient and healthy control participants at MONTH 0 and 8.
3. Use the provided form and point out instructions to participants located at the top of the form. Follow directions in the separate scoring guide (see **Appendix 9.6**).
4. This test can be sent home with participants to be completed within a week of the study visit and mailed back to the site study coordinator.

6.12. Hospital Anxiety and Depression Scale (HADS)

1. This is a SELF-ADMINISTERED assessment [8].
2. It will be given to patient and healthy control participants at MONTH 0 and 8.
3. Use the provided form and point out instructions to participants located at the top of the form (see **Appendix 9.7**).
4. There will be separate scores for Depression and Anxiety.

6.13. Center for Neurologic Study – Lability Scale (CNS-LS)

1. This is a SELF-ADMINISTERED assessment [9].
2. It will be given to patient and healthy control participants at MONTH 0 and 8.
3. Use the provided form and point out instructions to participants located at the fop of the form (see **Appendix 9.8**).
4. Point out to the participants that the answer they choose should describe the way they have been feeling *during the past week*. The numeric answer should be written in the space provided.
5. This test can be sent home with participants to be completed within a week of the study visit and mailed back to the site study coordinator.

6.14. Frontal Systems Behaviour Scale (FrSBe) Self-Rating Form

1. This is a SELF-ADMINISTERED assessment [10].
2. It will be given to patient participants ONLY, at MONTH 0 and 8.
3. Use the provided form and point out instructions to participants located at the top of the form (see **Appendix 9.9**).
4. Point out to the patient participants that they will need to score each phrase *before illness and injury AND at the present time***.** Also, point out the scoring scale at the top of each page.
5. This test can be sent home with patient participants to be completed within a week of the study visit and mailed back to the site study coordinator.
6. Transfer the circled scores into the corresponding shaded boxes on the score sheet, Subtotal each column and then calculate the total score.

6.15. Frontal Systems Rating Behavior Scale (FrSBe) Family-Rating Form

1. This is a SELF-ADMINISTERED questionnaire.
2. It will be given to consented caregivers to complete at MONTH 0 and 8.
3. Use the provided form and point out instructions to caregivers located at the top of the form (see **Appendix 9.9**).
4. Point out to the caregivers that they will need to score each phrase *before illness and injury AND at the present time.* Also, point out the scoring scale at the top of each page.
5. This test can be sent home with consented caregiver to be completed within a week of the study visit and mailed back to the site study coordinator.
6. Transfer the circled scores into the corresponding shaded boxes on the score sheet, Subtotal each column and then calculate the total score.

6.16. Stroop Test (Victoria version)

1. The Stroop Test will be administered to patient and healthy control participants at MONTH 0 and 8 [11].
2. Use the provided HARD-COPY stimulus cards (1 **stimulus cards / site**) and forms, and follow directions in the separate administration and scoring guidelines (see **Appendix 9.10**).
3. Patient participants with dysarthria/anarthria will use the provided color block pointing sheet to tap the colors rather than saying them. Note the use of color block pointing modification on the *Neurocognitive Evaluation Form.*

6.17. Judgment of Line Orientation (JLO)

1. The JLO test should be done ONLY if the ECAS Visuospatial score is less than or equal to 10 [12].
2. It will be administered to patient and healthy control participants, if necessary, at MONTH 0 and 8. Alternate versions of the form will be used at each visit, respectively (Form V, Form H).
3. Use the provided form and HARD-COPY stimulus book **(1 stimulus book/site)**, follow directions in the separate administration and scoring guidelines (see **Appendix 9.11**).

6.18. Amyotrophic Lateral Sclerosis Assessment Questionnaire Short Form (ALSAQ-5)

1. This is a SELF-ADMINISTERED PROM [13].
2. It will be given to patient participants ONLY, at MONTH 0, 4, 8, and 12.
3. Use the provided form and point out instructions to participants located at the top of the form (see **Appendix 9.12**).
4. This test can be sent home with participants to be completed within a week of the study visit and mailed back to the site study coordinator.

6.19. World Health Organization Quality of Life-BREF Scale (WHOQOL-BREF)

1. This is a SELF-ADMINISTERED PROM [14].
2. It will be given to patient participants at MONTH 0, 4, 8 and 12 and to healthy control participants at MONTH 0 and 8.
3. Use the provided form and point out instructions to participants located at the top of the form (see **Appendix 9.13**).
4. This test can be sent home with participants to be completed within a week of the study visit and mailed back to the site study coordinator.

7. DATA AND RECORDS MANAGEMENT

Please record scores for each assessment in the *Neurocognitive Evaluation Form* for each visit (see **Appendix 9.14**).

8. REFERENCES

1. Nasreddine, Z.S., et al., *The Montreal Cognitive Assessment, MoCA: a brief screening tool for mild cognitive impairment.* J Am Geriatr Soc, 2005. **53**(4): p. 695-9.
2. Abrahams, S., et al., *Screening for cognition and behaviour changes in ALS.* Amyotroph Lateral Scler Frontotemporal Degener, 2014. **15**(1-2): p. 9-14.
3. Crockford, C.J., et al., *ECAS A-B-C: alternate forms of the Edinburgh Cognitive and Behavioural ALS Screen.* Amyotroph Lateral Scler Frontotemporal Degener, 2018. **19**(1-2): p. 57-64.
4. Niven, E., et al., *Validation of the Edinburgh Cognitive and Behavioural Amyotrophic Lateral Sclerosis Screen (ECAS): A cognitive tool for motor disorders.* Amyotroph Lateral Scler Frontotemporal Degener, 2015. **16**(3-4): p. 172-9.
5. Benton, A.L., *Differential behavioural effects in frontal lobe disease.* Neuropsychologia, 1968. **6**(1): p. 53-60.
6. Kaplan, E.G.H.W.S.S.O.L.-V.A.v., *Boston naming test*. 2001, Austin,TX: Pro-ed.
7. Kreutzer, J.S.C.B.D.J., *Encyclopedia of clinical neuropsychology.* 2011: p. 1274-1274.
8. Zigmond, A.S. and R.P. Snaith, *The hospital anxiety and depression scale.* Acta Psychiatr Scand, 1983. **67**(6): p. 361-70.
9. Moore, S.R., et al., *A self report measure of affective lability.* J Neurol Neurosurg Psychiatry, 1997. **63**(1): p. 89-93.
10. Grace, J. and P.F. Malloy, *Frontal Systems Behavior Scale: Professional manual*. 2001, Psychological Assessment Resources, Inc.: Lutz, Florida.
11. Regard, M., *Cognitive rigidity and flexibility: a neuropsychological study.* 1981, University of Victoria.
12. Benton, A.L., *Judgment of Line Orientation*. 1983, Lutz, Florida: Psychological Assessment Resources.
13. Golab-Janowska M, H.K.S.J., *Usefulness of the ALSAQ-5 scale in evaluation of quality of life in amyotrophic lateral sclerosis.* Neurol. Neurochir. Pol. Neurologia i Neurochirurgia Polska, 2010. **44**(6): p. 560-566.
14. Young, C.A.M.R.A.-C.A.B.G.C.S.D.D.J.E.J.H.C.O.H.T.M.C.J.M.T.P.A., *Measuring quality of life in ALS/MND: validation of the WHOQOL-BREF.* Amyotroph Lateral Scler Frontotemporal Degener Amyotrophic lateral sclerosis & frontotemporal degeneration, 2020: p. 1-9.

9. APPENDICES

Appendix 9.1 Montreal Cognitive Assessment (MoCA)

Appendix 9.1.1: MoCA [ENGLISH]
Appendix 9.1.2: MoCA Administration/Scoring Guidelines [ENGLISH]
Appendix 9.1.3: MoCA [FRENCH]
Appendix 9.1.4: MoCA modifications CLOCK
Appendix 9.1.5: MoCA modifications CUBE

Appendix 9.1.1: MoCA [ENGLISH]

Found in [MoCA (EN, V8.1, 28Jun2017) - CAPTURE ALS]

MONTREAL COGNITIVE ASSESSMENT (MOCA®)
Version 8.1 English

Name:
Education: Date of birth:
Sex: DATE:

		POINTS
**VISUOSPATIAL / EXECUTIVE** 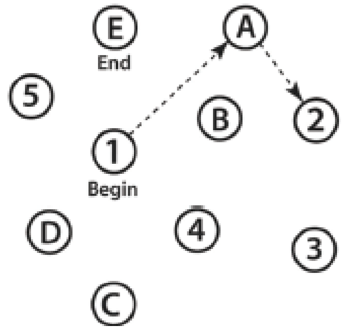 [ ] 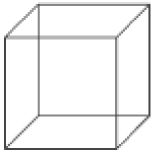 Copy cube [ ]	Draw CLOCK (Ten past eleven) (3 points) [ ] Contour [ ] Numbers [ ] Hands	__/5
**NAMING** 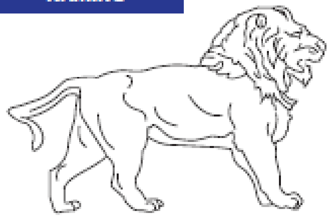 [ ] 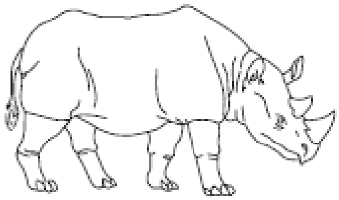 [ ] 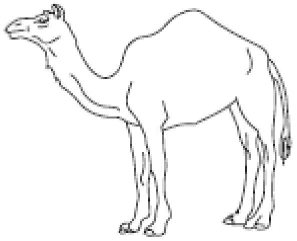 [ ]		__/3

**MEMORY** Read list of words, subject must repeat them. Do 2 trials, even if 1st trial is successful. Do a recall after 5 minutes.		FACE	VELVET	CHURCH	DAISY	RED	NO POINTS
	1ST TRIAL						
	2ND TRIAL						

		POINTS
**ATTENTION** Read list of digits (1 digit/ sec.).	Subject has to repeat them in the forward order. [ ] 2 1 8 5 4<br>Subject has to repeat them in the backward order. [ ] 7 4 2	__/2
Read list of letters. The subject must tap with his hand at each letter A. No points if ≥ 2 errors	[ ] F B A C M N A A J K L B A F A K D E A A A J A M O F A A B	__/1
Serial 7 subtraction starting at 100.	[ ] 93 [ ] 86 [ ] 79 [ ] 72 [ ] 65<br>4 or 5 correct subtractions: **3 pts**, 2 or 3 correct: **2 pts**, 1 correct: **1 pt**, 0 correct: **0**	__/3
**LANGUAGE** Repeat:	I only know that John is the one to help today. [ ]<br>The cat always hid under the couch when dogs were in the room. [ ]	__/2
Fluency: Name maximum number of words in one minute that begin with the letter F.	[ ] _____ (N≥11 words)	__/1
**ABSTRACTION** Similarity between e.g. banana - orange = fruit	[ ] train - bicycle [ ] watch - ruler	__/2

**DELAYED RECALL**	(MIS)	Has to recall words WITH NO CUE	FACE	VELVET	CHURCH	DAISY	RED	Points for UNCUED recall only	__/5
			[ ]	[ ]	[ ]	[ ]	[ ]		
Memory Index Score (MIS)	X3								
	X2	Category cue						MIS = ____/15	
	X1	Multiple choice cue							

**ORIENTATION**	[ ] Date [ ] Month [ ] Year [ ] Day [ ] Place [ ] City	__/6

© Z. Nasreddine MD www.mocatest.org

MIS: /15
(Normal ≥ 26/30)
Add 1 point if ≤ 12 yr edu

TOTAL __/30

Administered by: ______________________

Training and Certification are required to ensure accuracy

Appendix 9.1.2: MoCA Administration/Scoring Guidelines [ENGLISH]
Found in [MoCA (EN, V8.1, 28Jun2017) - CAPTURE ALS]

Montreal Cognitive Assessment (MoCA)
Version 8.1

Administration and Scoring Instructions

The Montreal Cognitive Assessment (MoCA) was designed as a rapid screening instrument for mild cognitive dysfunction. It assesses different cognitive domains: attention and concentration, executive functions, memory, language, visuoconstructional skills, conceptual thinking, calculations, and orientation. The MoCA may be administered by anyone who understands and follows the instructions, however, only a health professional with expertise in the cognitive field may interpret the results. Time to administer the MoCA is approximately 10 minutes. The total possible score is 30 points; a score of 26 or above is considered normal.

All instructions may be repeated once.

1. Alternating Trail Making:

Administration: The examiner instructs the subject: *"Please draw a line going from a number to a letter in ascending order. Begin here* [point to (1)] *and draw a line from 1 then to A then to 2 and so on. End here* [point to (E)]."

Scoring: One point is allocated if the subject successfully draws the following pattern: 1- A- 2- B- 3- C- 4- D- 5- E, without drawing any lines that cross. Any error that is not immediately self-corrected (meaning corrected before moving on to the Cube task) earns a score of 0. A point is not allocated if the subject draws a line to connect the end (E) to the beginning (1).

2. Visuoconstructional Skills (Cube):

Administration: The examiner gives the following instructions, pointing to the cube: *"Copy this drawing as accurately as you can."*

Scoring: One point is allocated for a correctly executed drawing.
- Drawing must be three-dimensional.
- All lines are drawn.
- All lines meet with little or no space.
- No line is added.
- Lines are relatively parallel and their length is similar (rectangular prisms are accepted).
- The cube's orientation in space must be preserved.

A point is not assigned if any of the above criteria is not met.

3. Visuoconstructional Skills (Clock):

Administration: The examiner must ensure that the subject does not look at his/her watch while performing the task and that no clocks are in sight. The examiner indicates the appropriate space and gives the following instructions: *"Draw a clock. Put in all the numbers and set the time to 10 past 11."*

Scoring: One point is allocated for each of the following three criteria:

© Z. Nasreddine MD

• Contour (1 pt.): the clock contour must be drawn (either a circle or a square). Only minor distortions are acceptable (e.g., slight imperfection on closing the circle). If the numbers are arranged in a circular manner but the contour is not drawn the contour is scored as incorrect.
• Numbers (1 pt.): all clock numbers must be present with no additional numbers. Numbers must be in the correct order, upright and placed in the approximate quadrants on the clock face. Roman numerals are acceptable. The numbers must be arranged in a circular manner (even if the contour is a square). All numbers must either be placed inside or outside the clock contour. If the subject places some numbers inside the clock contour and some outside the clock contour, (s)he does not receive a point for Numbers.
• Hands (1 pt.): there must be two hands jointly indicating the correct time. The hour hand must be clearly shorter than the minute hand. Hands must be centered within the clock face with their junction close to the clock center.

4. Naming:

Administration: Beginning on the left, the examiner points to each figure and says: *"Tell me the name of this animal."*

Scoring: One point is given for each of the following responses: (1) lion (2) rhinoceros or rhino (3) camel or dromedary.

5. Memory:

Administration: The examiner reads a list of five words at a rate of one per second, giving the following instructions: *"This is a memory test. I am going to read a list of words that you will have to remember now and later on. Listen carefully. When I am through, tell me as many words as you can remember. It doesn't matter in what order you say them."* The examiner marks a check in the allocated space for each word the subject produces on this first trial. The examiner may not correct the subject if (s)he recalls a deformed word or a word that sounds like the target word. When the subject indicates that (s)he has finished (has recalled all words), or can recall no more words, the examiner reads the list a second time with the following instructions: *"I am going to read the same list for a second time. Try to remember and tell me as many words as you can, including words you said the first time."* The examiner puts a check in the allocated space for each word the subject recalls on the second trial. At the end of the second trial, the examiner informs the subject that (s)he will be asked to recall these words again by saying: *"I will ask you to recall those words again at the end of the test."*

Scoring: No points are given for Trials One and Two.

6. Attention:

Forward Digit Span: Administration: The examiner gives the following instructions: "*I am going to say some numbers and when I am through, repeat them to me exactly as I said them.*" The examiner reads the five number sequence at a rate of one digit per second.

Backward Digit Span: Administration: The examiner gives the following instructions: "*Now I am going to say some more numbers, but when I am through you must repeat*

© Z. Nasreddine MD

them to me in the backward order." The examiner reads the three number sequence at a rate of one digit per second. If the subject repeats the sequence in the forward order, the examiner may not ask the subject to repeat the sequence in backward order at this point.

Scoring: One point is allocated for each sequence correctly repeated (N.B.: the correct response for the backward trial is 2-4-7).

Vigilance: Administration: The examiner reads the list of letters at a rate of one per second, after giving the following instructions: "*I am going to read a sequence of letters. Every time I say the letter A, tap your hand once. If I say a different letter, do not tap your hand.*"

Scoring: One point is allocated if there is zero to one error (an error is a tap on a wrong letter or a failure to tap on letter A).

Serial 7s: Administration: The examiner gives the following instructions: *"Now, I will ask you to count by subtracting 7 from 100, and then, keep subtracting 7 from your answer until I tell you to stop."* The subject must perform a mental calculation, therefore, (s)he may not use his/her fingers nor a pencil and paper to execute the task. The examiner may not repeat the subject's answers. If the subject asks what her/his last given answer was or what number (s)he must subtract from his/her answer, the examiner responds by repeating the instructions if not already done so.

Scoring: This item is scored out of 3 points. Give no (0) points for no correct subtractions, 1 point for one correct subtraction, 2 points for two or three correct subtractions, and 3 points if the subject successfully makes four or five correct subtractions. Each subtraction is evaluated independently; that is, if the subject responds with an incorrect number but continues to correctly subtract 7 from it, each correct subtraction is counted. For example, a subject may respond "92 – 85 – 78 – 71 – 64" where the "92" is incorrect, but all subsequent numbers are subtracted correctly. This is one error and the task would be given a score of 3.

7. **Sentence repetition:**

Administration: The examiner gives the following instructions: *"I am going to read you a sentence. Repeat it after me, exactly as I say it* [pause]: ***I only know that John is the one to help today."*** Following the response, say: *"Now I am going to read you another sentence. Repeat it after me, exactly as I say it* [pause]: ***The cat always hid under the couch when dogs were in the room."***

Scoring: One point is allocated for each sentence correctly repeated. Repetitions must be exact. Be alert for omissions (e.g., omitting "only"), substitutions/additions (e.g., substituting "only" for "always"), grammar errors/altering plurals (e.g. "hides" for "hid"), etc.

8. **Verbal fluency:**

Administration: The examiner gives the following instructions: *"Now, I want you to tell me as many words as you can think of that begin with the letter F. I will tell you to stop after one minute. Proper nouns, numbers, and different forms of a verb are not permitted. Are you ready?* [Pause] [Time for 60 sec.] *Stop."* If the subject names two consecutive

© Z. Nasreddine MD

words that begin with another letter of the alphabet, the examiner repeats the target letter if the instructions have not yet been repeated.

Scoring: One point is allocated if the subject generates 11 words or more in 60 seconds. The examiner records the subject's responses in the margins or on the back of the test sheet.

9. Abstraction:

Administration: The examiner asks the subject to explain what each pair of words has in common, starting with the example: *"I will give you two words and I would like you to tell me to what category they belong to* [pause]: *an orange and a banana.*" If the subject responds correctly the examiner replies: *''Yes, both items are part of the category Fruits.''* If the subject answers in a concrete manner, the examiner gives one additional prompt: *"Tell me another category* to *which these items belong to."* If the subject does not give the appropriate response *(fruits)*, the examiner says: *"Yes, and they also both belong to the category Fruits."* No additional instructions or clarifications are given. After the practice trial, the examiner says: *"Now, a train and a bicycle."* Following the response, the examiner administers the second trial by saying: *"Now, a ruler and a watch."* A prompt (one for the entire abstraction section) may be given if none was used during the example.

Scoring: Only the last two pairs are scored. One point is given for each pair correctly answered. The following responses are acceptable:
- train-bicycle = means of transportation, means of travelling, you take trips in both
- ruler-watch = measuring instruments, used to measure

The following responses are **not** acceptable:
- train-bicycle = they have wheels
- ruler-watch = they have numbers

10. Delayed recall:

Administration: The examiner gives the following instructions: *"I read some words to you earlier, which I asked you to remember. Tell me as many of those words as you can remember."* The examiner makes a check mark (√) for each of the words correctly recalled spontaneously without any cues, in the allocated space.

Scoring: **One point is allocated for each word recalled freely without any cues.**

Memory index score (MIS):

Administration: Following the delayed free recall trial, the examiner provides a category (semantic) cue for each word the subject was unable to recall. Example: *''I will give you some hints to see if it helps you remember the words, the first word was a body part.''* If the subject is unable to recall the word with the category cue, the examiner provides him/her with a multiple choice cue. Example: *"Which of the following words do you think it was, NOSE, FACE, or HAND?"* All non-recalled words are prompted in this manner. The examiner identifies the words the subject was able to recall with the help of a cue (category or multiple-choice) by placing a check mark (√) in the appropriate space. The cues for each word are presented below:

© Z. Nasreddine MD

Target Word	Category Cue	Multiple Choice
FACE	body part	nose, face, hand (shoulder, leg)
VELVET	type of fabric	denim, velvet, cotton (nylon, silk)
CHURCH	type of building	church, school, hospital (library, store)
DAISY	type of flower	rose, daisy, tulip (lily, daffodil)
RED	color	red, blue, green (yellow, purple)

* The words in parentheses are to be used if the subject mentions one or two of the multiple choice responses during the category cuing.

Scoring: To determine the MIS (which is a sub-score), the examiner attributes points according to the type of recall (see table below). The use of cues provides clinical information on the nature of the memory deficits. For memory deficits due to retrieval failures, performance can be improved with a cue. For memory deficits due to encoding failures, performance does not improve with a cue.

**MIS scoring**				**Total**
Number of words recalled spontaneously	…	multiplied by	3	…
Number of words recalled with a category cue	…	multiplied by	2	…
Number of words recalled with a multiple choice cue	…	multiplied by	1	…
	**Total MIS** (add all points)			**---/15**

11. Orientation:

Administration: The examiner gives the following instructions: "*Tell me today's date.*" If the subject does not give a complete answer, the examiner prompts accordingly by saying: *"Tell me the [year, month, exact date, and day of the week]."* Then the examiner says: *"Now, tell me the name of this place, and which city it is in."*

Scoring: One point is allocated for each item correctly answered. The date and place (name of hospital, clinic, office) must be exact. No points are allocated if the subject makes an error of one day for the day and date.

TOTAL SCORE: Sum all subscores listed on the right-hand side. Add one point for subject who has 12 years or fewer of formal education, for a possible maximum of 30 points. A final total score of 26 and above is considered normal.

Please refer to the MoCA website at www.mocatest.org for more information on the MoCA.

© Z. Nasreddine MD

Appendix 9.1.3: MoCA [FRENCH]

Found in [MoCA (FR, V8.1, May2017) - CAPTURE ALS]

MONTREAL COGNITIVE ASSESSMENT (MOCA®)

Version 8.1 Français

Nom :
Scolarité :   Date de naissance :
Sexe :   DATE :

	POINTS
**VISUOSPATIAL / EXÉCUTIF**	

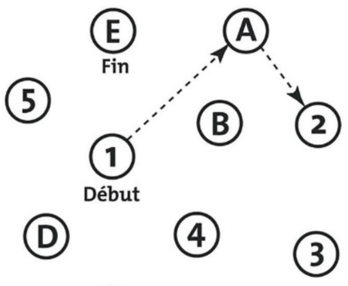

[ ]

Copier le cube

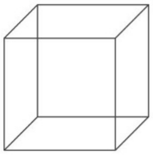

[ ]

Dessiner une HORLOGE (Onze heures et dix minutes) (3 points)

[ ] Contour   [ ] Chiffres   [ ] Aiguilles

__/5

DÉNOMINATION

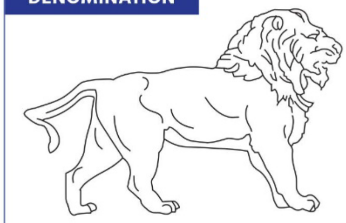

[ ]

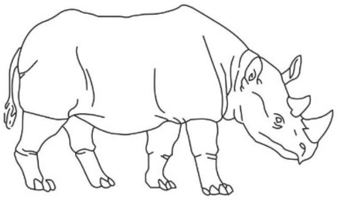

[ ]

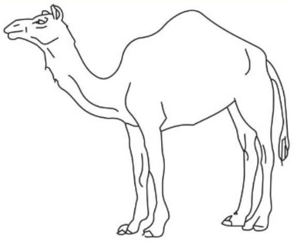

[ ]

__/3

MÉMOIRE

Lire la liste de mots, le sujet doit la répéter. Faire 2 essais même si le 1er essai est réussi. Faire un rappel après 5 minutes.

	VISAGE	VELOURS	ÉGLISE	MARGUERITE	ROUGE
1ER ESSAI					
2E ESSAI					

PAS DE POINT

ATTENTION

Lire la série de chiffres (1 chiffre / sec.).   Le sujet doit la répéter dans le même ordre.   [ ] 2 1 8 5 4

Le sujet doit la répéter à l'envers.   [ ] 7 4 2

__/2

Lire la série de lettres. Le sujet doit taper de la main à chaque lettre A. Pas de points si ≥ 2 erreurs.

[ ]   F B A C M N A A J K L B A F A K D E A A A J A M O F A A B

__/1

Soustraire série de 7 à partir de 100.   [ ] 93   [ ] 86   [ ] 79   [ ] 72   [ ] 65

4 ou 5 soustractions correctes : **3 pts**,   2 ou 3 correctes : **2 pts**,   1 correcte : **1 pt**,   0 correcte : **0 pt**

__/3

LANGAGE

Répéter : Le colibri a déposé ses œufs sur le sable.   [ ]

L'argument de l'avocat les a convaincus.   [ ]

__/2

Fluidité du langage. Nommer un maximum de mots commençant par la lettre « F » en 1 min.   [ ] _____ (N ≥ 11 mots)

__/1

ABSTRACTION

Similitude entre ex : banane - orange = fruit   [ ] train - bicyclette   [ ] montre - règle

__/2

RAPPEL

	(MIS)	Doit se souvenir des mots SANS INDICE	VISAGE	VELOURS	ÉGLISE	MARGUERITE	ROUGE	Points pour rappel SANS INDICE seulement
	X3		[ ]	[ ]	[ ]	[ ]	[ ]	
Memory Index Score (MIS)	X2	Indice de catégorie						MIS = ____ /15
	X1	Indice choix multiples						

__/5

ORIENTATION

[ ] Date   [ ] Mois   [ ] Année   [ ] Jour   [ ] Endroit   [ ] Ville

__/6

© Z. Nasreddine MD

www.mocatest.org

MIS: /15

(Normal ≥ 26/30)

Ajouter 1 point si scolarité ≤ 12 ans

TOTAL __/30

Administré par : ______________________________

Entraînement et certification requis pour assurer la précision.

Appendix 9.1.4: MoCA modifications CLOCK

Found in [MoCA modifications CLOCK - CAPTURE ALS]

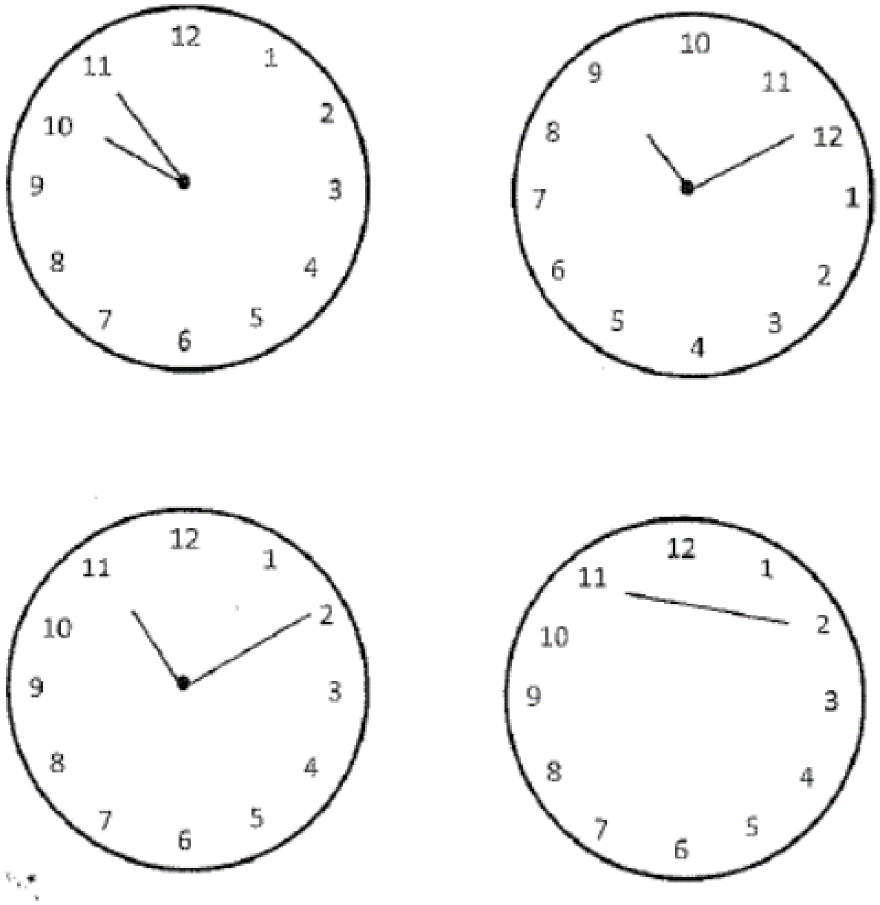

Appendix 9.1.5: MoCA modifications CUBE

Found in [MoCA modifications CUBE - CAPTURE ALS]

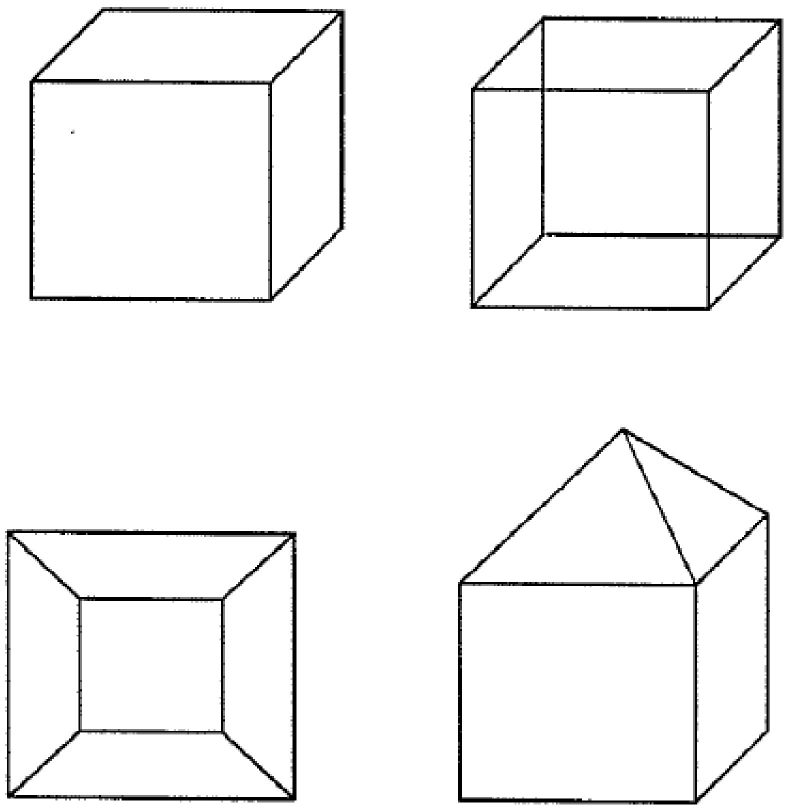

Appendix 9.2: Edinburgh Cognitive and Behavioral ALS Screen (ECAS)

Appendix 9.2.1: ECAS A [ENGLISH]
Appendix 9.2.2: ECAS A [FRENCH]
Appendix 9.2.3: ECAS B [ENGLISH]
Appendix 9.2.4: ECAS B [FRENCH]
Appendix 9.2.5: ECAS C [ENGLISH]
Appendix 9.2.6: ECAS C [FRENCH]
Appendix 9.2.7: ECAS Guidance A, B, C [ENGLISH]
Appendix 9.2.8: ECAS Guidance A, B, C [FRENCH]

Appendix 9.2.1: ECAS A [ENGLISH]
Found in [ECAS A (EN, V1.1, 2020) - CAPTURE ALS]

EDINBURGH COGNITIVE AND BEHAVIORAL ALS SCREEN – UNIVERSITY OF PENNSYLVANIA (ECAS-PENN)

North American English Version Form A, Version 1.1 Revised (2020)

Developed by Sharon Abrahams and Thomas H. Bak, University of Edinburgh
Adapted for use in North America by Katya Rascovsky, Corey McMillan, and Murray Grossman, University of Pennsylvania, in collaboration with Michael Benatar on behalf of the Clinical Research in ALS and Related Disorders for Therapeutic Development (CReATe) Consortium.

Date of testing: ....
Occupation: ....
....
Handedness: ....
Years of education: ....

Name/ID: ....
Date of Birth: ....
Highest Completed Degree:…………………………..
Premorbid language difficulties ………………………….

LANGUAGE - Naming

➲ Ask: Say or write down the names of these pictures:

Score
0-8
☐

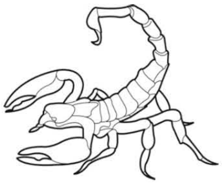
.... ☐

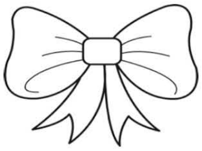
.... ☐

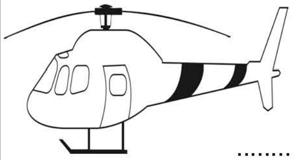
.... ☐

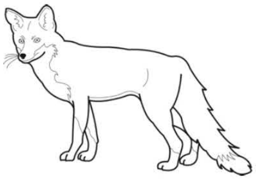
.... ☐

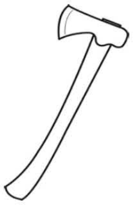
.... ☐

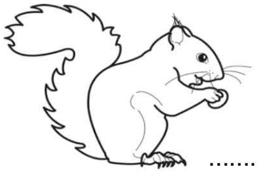
.... ☐

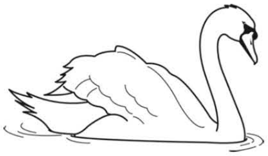
.... ☐

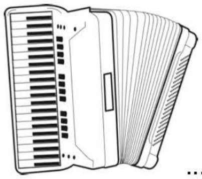
.... ☐

LANGUAGE - Comprehension

➲ Ask: Point or say the one which is:

1. Something you can fly in ....
2. Something with webbed feet ....
3. An animal that climbs trees ....
4. Something used for chopping ....
5. A means of transportation................................
6. Something with a sharp edge ....
7. Something with a sting ....
8. Something with a diet of nuts and seeds ....

Score
0-8
☐

MEMORY – Immediate recall

➲ Say: 'I am going to read you a short story. Please listen carefully. When I am finished, say or write as much as you can remember'. Score according to the Administration and Guidance Notes.

Last Sunday, the annual park cleanup took place in Marigold Woods. Forty two people joined in to remove old bicycles and shopping carts. Mr. Robert Webber from the woodland project told local reporters that he was very impressed and especially proud of the 17 children who came along.

Score 0-10

Also use this score to calculate % retained (bottom p.7)

LANGUAGE - Spelling

➲ Say: 'Spell, either by speaking or writing, the following words.' If the person is using assistive technology, ask them to turn off any predictive text facility. Give one point on "coathanger", "lawnmower", "skateboard", and "screwdriver" if the person inserts a "space" when spelling the word.

1. Envelope ....
2. Skateboard ....
3. Constructing ....
4. Partner ....
5. Biscuit ....
6. Lawnmower ....
7. Deliver ....
8. Recorded ....
9. Coathanger ....
10. Orchestra ....
11. Screwdriver ....
12. Brought ....

Score 0-12

FLUENCY - Letter S ☐ Oral ☐ Written

➲ Say: 'I am going to give you a letter of the alphabet and I would like you to say or write as many different words as you can beginning with that letter, but not names of people or places, or numbers.'
- If speaking, say 'You will have **one** minute. The letter is S.'
- If writing, say: 'You will have **two** minutes. The letter is S.'

No. of correct words =

Time to read/ copy aloud =

Vfi =

➲ Next the person copies/reads these words aloud.

- If speaking, say: 'read aloud these words as fast as possible. Before you do this, check that you can read them. I will time you. Ready? Begin.'
- If writing, say: 'copy these words as fast as possible. I will time you. Ready? Begin.'

Verbal Fluency Index (Vfi) calculation:

If spoken:
Vfi = (60seconds – no. of seconds to read aloud words) / No. of correct words generated

If written:
Vfi = (120seconds – no. of seconds to copy words) / No. of correct words generated

VFI conversion to score table

SPOKEN VFI	WRITTEN VFI	**Score**
≥ 12.00	≥ 20.00	**0**
10.00 to <12.00	16.50 to < 20.00	**2**
8.00 to < 10.00	13.00 to < 16.50	**4**
6.00 to < 8.00	9.50 to < 13.00	**6**
4.00 to < 6.00	6.00 to < 9.50	**8**
2.00 to < 4.00	2.50 to < 6.00	**10**
< 2.00	< 2.50	**12**

Score 0-12

EXECUTIVE – Reverse Digit Span

➲ Say: 'I am going to say some numbers and I would like you to say them back to me in reverse order. For example, if I say '2 3 4', you should say '4 3 2'. Let's practice. If I say '7 1 9', what would you say?' Stop when person gets both trials of a line wrong. Score total number of trials correct. After each pair of trials remind the participant 'Now the number of items will increase'.

Score 0-12

Trial		Check	Trial		Check
1	2 6		2	5 8	
3	9 3 5		4	4 1 6	
5	7 2 8 4		6	9 5 7 3	
7	6 9 4 2 1		8	8 3 2 5 6	
9	8 1 3 5 7 9		10	3 6 2 7 3 4	
11	1 6 9 3 5 8 6		12	2 3 6 8 4 9 2	

EXECUTIVE – Alternation

➲ Say: 'I want you to alternate between numbers and letters, starting with 1-A, then 2-B, 3-C, and so on. Please continue from there, alternating between numbers and letters, in order, without skipping any until I tell you to stop. Let's begin together: 1-A, 2-B, 3-C…'

Score 0-12

Trial		Check	Trial		Check	Trial		Check	Trial		Check
1	4-D		2	5-E		3	6-F		4	7-G	
5	8-H		6	9-I		7	10-J		8	11-K	
9	12-L		10	13-M		11	14-N		12	15-O	

FLUENCY - Letter T ☐ Oral ☐ Written

➲ Say: 'I am going to give you a letter of the alphabet and I would like you to say or write as many different words as you can beginning with that letter, but not names of people or places, or numbers. This time the word must only be **four letters** long. No more or less than four letters'
- If speaking, say 'You will have **one** minute. The letter is T.'
- If writing, say: 'You will have **two** minutes. The letter is T.'

No. of correct words =

Time to read/ copy aloud =

Vfi =

➲ Next the person copies/reads these words aloud.

- If speaking, say: 'read aloud these words as fast as possible. Before you do this, check that you can read them. I will time you. Ready? Begin.'
- If writing, say: 'copy these words as fast as possible. I will time you. Ready? Begin.'

Verbal Fluency Index (Vfi) calculation:

If spoken:
Vfi = 60seconds – no. of seconds to read aloud words / No. of correct words generated

If written:
Vfi = 120seconds – no. of seconds to copy words / No. of correct words generated

VFI conversion to score table

SPOKEN VFI	WRITTEN VFI	Score
≥ 20.00	≥ 27.25	**0**
16.75 to < 20.00	23.00 to < 27.25	**2**
13.50 to < 16.75	18.75 to < 23.00	**4**
10.25 to < 13.50	14.50 to < 18.75	**6**
7.00 to < 10.25	10.25 to < 14.50	**8**
3.75 to < 7.00	6.00 to < 10.25	**10**
< 3.75	< 6.00	**12**

Score 0-12

VISUOSPATIAL – Dot Counting

➲ Say: 'I would like you to count how many dots are in each box, but without pointing to them.'

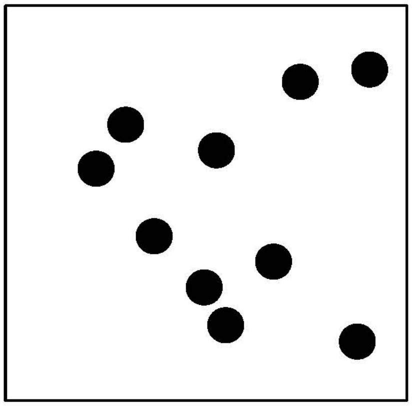 ☐

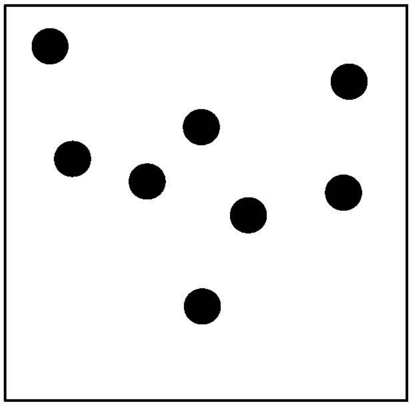 ☐

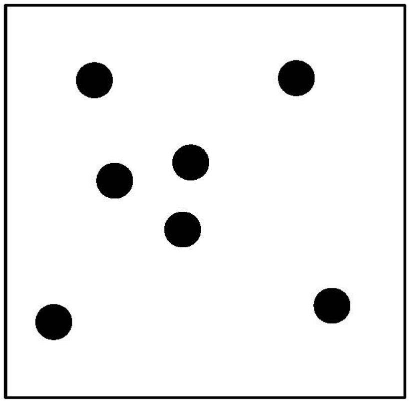 ☐

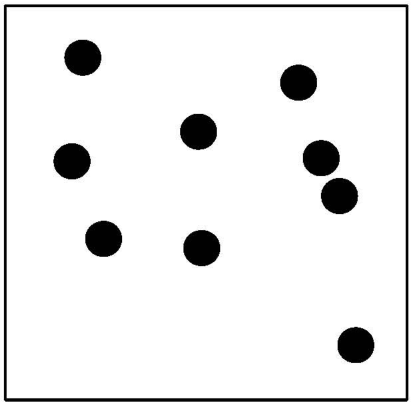 ☐

Score 0-4 ☐

VISUOSPATIAL – Cube Counting

➲ Say: 'How many cubes are in each structure, including the ones you may not be able to see?'

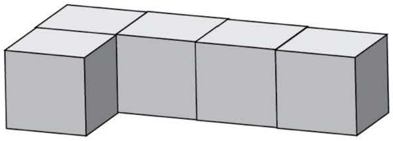 ☐

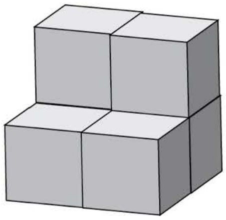 ☐

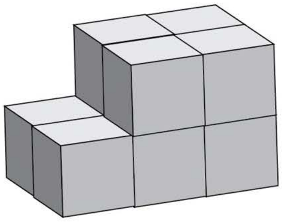 ☐

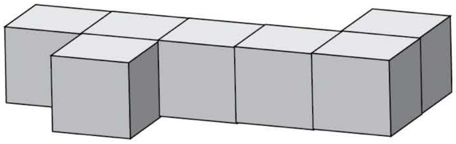 ☐

Score 0-4 ☐

VISUOSPATIAL – Number Location

➲ Say: 'Which number corresponds to the position of the dot?'

Score 0-4

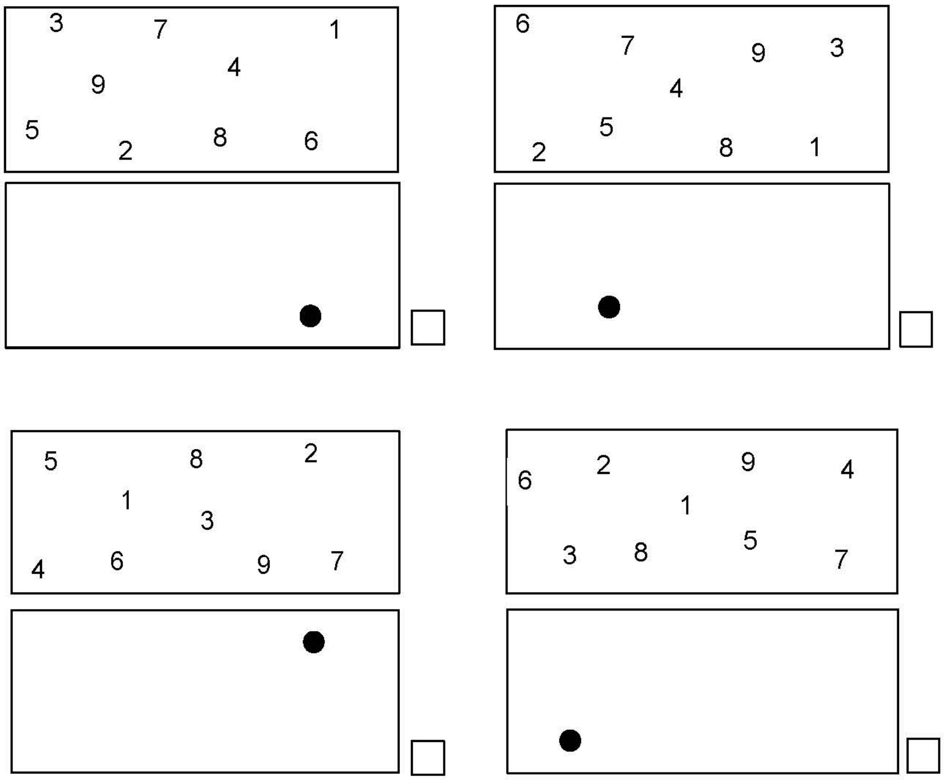

EXECUTIVE – Sentence Completion

➲ Say: 'Listen carefully to these sentences and as soon as I have finished reading them, please tell me, or write, a word that finishes the sentence as quickly as possible. For example, '*She was so tired that she went straight to…bed*'. Do not score.

1. He called the restaurant to reserve a ....
2. When she got up in the morning, the sun was....................

➲ Say: 'Now I'd like you to do that again, but this time the word you give should not make sense whatsoever in the context of the sentence. It must not be related to the word that actually completes the sentence. For example, '*John cut his hand with the sharp…orange*'. If the person does not respond within 20 seconds, move onto the next question.

1. The mailman knocked on the ....
2. He brought his umbrella with him in case of ....
3. Sally spread her toast with butter and ....
4. John went to the barbers to get his hair ....
5. She dived into the swimming ....
6. They all went to the local café for something to ....

Give 2 points for different word, 1 for related word (e.g. associated or opposite meaning) or 0 for exact word.

Score
0-12

SOCIAL COGNITION – Part A

➲ Say: 'You are going to see some pictures, one in each corner of a box. You have to choose **which picture you like best.** Either point to or say which picture you like best. Please respond as quickly as possible.' Circle participant's choice.

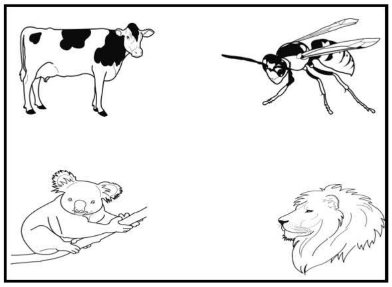

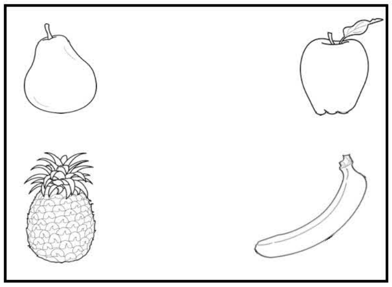

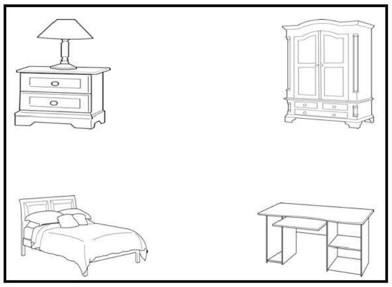

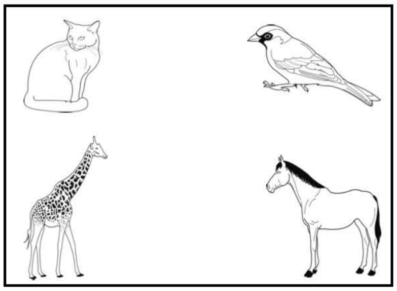

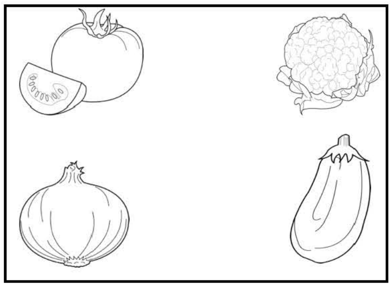

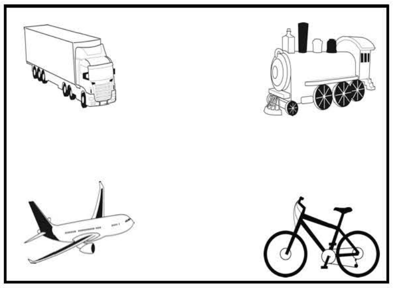

SOCIAL COGNITION – Part B

➲ Say: 'You are going to see some pictures, one in each corner of a box. You have to choose **which picture the face likes best.** Either point to or say which picture **the face likes best**. Please respond as quickly as possible.' Circle participant's choice. Correct items = 2 points, error = 1 point, egocentric error = 0 points.

Score 0-12

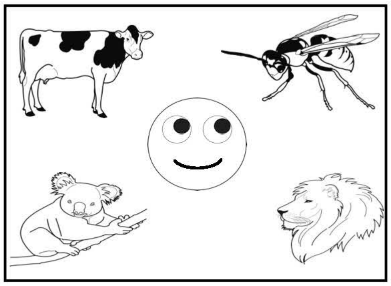

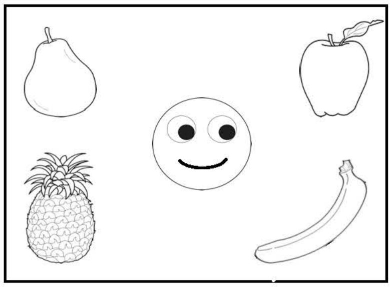

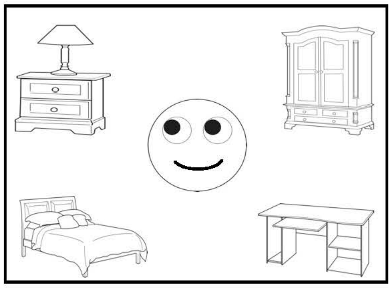

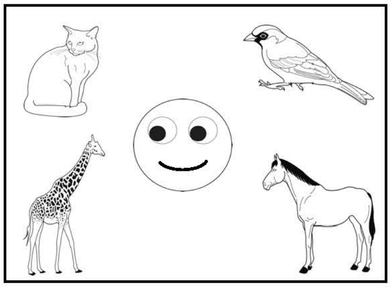

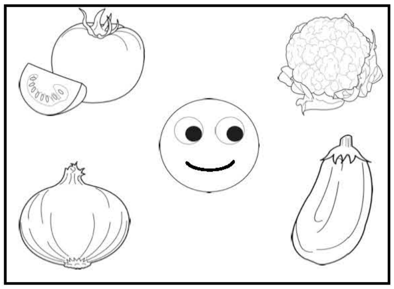

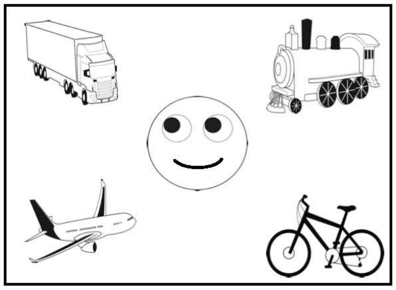

MEMORY – Delayed Recall

Scoring procedure for retention: obtain delayed recall performance (page 8) and, together with immediate recall score (page 2), determine percentage retained. Convert percentage retained to Score using table below. If delayed recall = 0, score = 0.

Delayed recall to percentage retained calculation

(Delayed recall score [page 8]) / (Immediate recall score [page 2[) *x 100 = % retained*

(...................................) / (...................................) *x 100 = ...% retained*

Percentage retained to converted score table

Percentage retained	**Converted score**	Percentage retained	**Converted score**
1-10%	**1**	51-60%	**6**
11-20%	**2**	61-70%	**7**
21-30%	**3**	71-80%	**8**
31-40%	**4**	81-90%	**9**
41-50%	**5**	91-100+ %	**10**

➲ Say: 'At the beginning of this interview, I read you a short story. Tell me as much as you can remember from that story'. Score according to the Administration and Guidance Notes.

Last Sunday, the annual park cleanup took place in Marigold Woods. Forty two people joined in to remove old bicycles and shopping carts. Mr. Robert Webber from the woodland project told local reporters that he was very impressed and especially proud of the 17 children who came along.

Delayed recall raw score (0-10) ☐

Converted retention score (0-10) ☐

MEMORY – Delayed Recognition

If all items recalled, skip and score 4. Otherwise ask questions below.

Say: 'Let's see if you can remember anything more about that story. I will ask you some questions, please tell me if they are true or false'.

Circle responses (true or false) and score 1 point for each item recognized in this section. Use table below to calculate score.

Score 0-4 ☐

Was the story about an event that occurred last Saturday?	T	**F**	1
Was the event the park cleanup?	**T**	F	1
Did this take place in Marigold Woods?	**T**	F	1
Did they remove old soda cans and candy wrappers?	T	**F**	1
Was the man in the story called Mr. Webber?	**T**	F	1
Was his first name 'Thomas'?	T	**F**	1
Was he from the local government?	T	**F**	1
Was he especially proud of the children for coming along?	**T**	F	1

Yes = True (T)
No = False (F)

Recognition to recognition score table

Number of correct answers	**Converted Score**
0-4	**0**
5	**1**
6	**2**
7	**3**
8	**4**

SCORES

**Language**	Naming, Comprehension, Spelling	/28
**Verbal Fluency**	Fluency Letter S, Fluency Letter T	/24
**Executive**	Reverse Digit Span, Alternation, Sentence Completion, Social Cognition	/48
	**ALS-SPECIFIC:**	**/100**
Memory	*Immediate recall, Delayed retention, Delayed recognition*	*/24*
Visuospatial	*Dot Counting, Cube Counting, Number Location*	*/12*
	ALS NON-SPECIFIC:	***/36***
	**ECAS TOTAL SCORE:**	**/136**

S. Abrahams & T.H. Bak
Dot counting is reproduced with kind permission from J. R. Hodges

EDINBURGH COGNITIVE AND BEHAVIORAL ALS SCREEN – UNIVERSITY OF PENNSYLVANIA (ECAS-PENN)
American English Form A, Version 1 (2018)

BEHAVIOR SCREEN – Caregiver Interview

➲ Please ask the caregiver about the following possible behaviors. Symptoms should have occurred repeatedly and not just on one instance, and may have occurred prior to the development of any motor signs. Tick 'Yes', 'No' or 'Don't Know'. If 'Yes', please provide a brief written description. Give one point for every 'Yes' response (maximum = 10).

**A**	**Behavioral disinhibition**				BEHAVIOR
1	Socially inappropriate behavior, e.g.<br>*inappropriate behavior with strangers*<br>*criminal behavior*	Y	N	DK	
2	Loss of manners or decorum, e.g.<br>*crude or sexually explicit remarks, jokes or opinions that may be offensive to others*<br>*lack of response to social cues*	Y	N	DK	
3	Impulsive, rash or careless actions, e.g.<br>*new onset gambling, or buying or selling property without regard for consequences*<br>*giving out personal information inappropriately, e.g. credit card numbers*	Y	N	DK	
**B**	**Apathy or inertia**				
4	Loss of interest, drive or motivation, e.g.<br>*passivity and lack of spontaneity*<br>*needs prompting to initiate or continue routine activities*	Y	N	DK	
**C**	**Loss of sympathy or empathy**				
5	Diminished response to other people's needs and feelings<br>*Positive rating on this feature should be based on specific examples that reflect a lack of understanding or indifference to other people's feelings, e.g*<br>*hurtful comments*<br>*disregard for others' pain or distress*	Y	N	DK	
6	Diminished social interest, interrelatedness, personal warmth or general closeness in social engagement, e.g.<br>*coldness*<br>*lack of eye contact*	Y	N	DK	
**D**	**Perseverative, stereotyped, compulsive or ritualistic behavior**				
7	Simple repetitive movements, e.g.<br>*tapping, clapping*<br>*scratching, picking skin or clothing*<br>*repeating words*	Y	N	DK	
8	Complex, compulsive or ritualistic behaviors, e.g.<br>*counting, cleaning rituals, checking*<br>*collecting, hoarding*	Y	N	DK	

E	Hyperorality and altered food preferences			
9	Altered food preferences, e.g.<br>*food fads*<br>*carbohydrate craving (particularly sweets)*	Y	N	DK
10	Binge eating or hyperorality, e.g.,<br>*cramming or continuing to eat despite satiety*<br>*oral exploration or consumption of inedible objects*	Y	N	DK
**SCORE**				
		**TOTAL**		*/10*

**SYMPTOMS**	
➲ Please check box if at least one of the symptoms was present in each of the following categories.	
**A. Behavioral disinhibition**	
**B. Apathy or inertia**	
**C. Loss of sympathy or empathy**	
**D. Perseverative, stereotyped, compulsive or ritualistic behaviour**	
**E. Hyperorality and altered food preferences**	

ALS Psychosis Screen

➲ Please ask the caregiver about the following possible symptoms. Check 'Yes', 'No' or 'Don't Know'. If 'Yes', please provide a brief written description. Give one point for every 'Yes' response (maximum = 3).

1	Has strange and/or bizarre beliefs and behaviors	Y	N	DK
2	Hears or sees things that are not there, and/or feels the presence of someone who is not there	Y	N	DK
3	Is overly suspicious, and/or feels persecuted	Y	N	DK
**SCORE**				
		**TOTAL**		*/3*

**ONSET AND DURATION OF SYMPTOMS**		
➲ Please check or complete box to indicate response.		
**1. Do these symptoms represent a CHANGE from the patient's previous behavior?**	Y	N
If yes, did the changes occur:		
a. BEFORE the onset of the disease?	Y	N
b. at the same time as other symptoms?	Y	N
c. AFTER the onset of the disease?	Y	N
**2. Do they still persist?**	Y	N
**3. If not, how long did they last?**		

Appendix 9.2.2: ECAS A [FRENCH]
Found in [ECAS A (FR, 15May2020) - CAPTURE ALS]

ECHELLE COGNITIVE ET COMPORTEMENTALE D'EDIMBOURG DE DÉPISTAGE DE LA SLA
ECAS Formulaire A (Version Français Canada 2020)
Developed by S. Abrahams and T.H. Bak, University of Edinburgh
Adapted for use in North America by K. Rascovsky and M. Grossman, University of Pennsylvania

Date d'essai: ……………………………………………………………
Occupation: ……………………………………………………………
……………………………………………………………………………
Main Dominante:………………………………………………………
Années d'éducation:……………………………………………………

Nom/ID: ……………………………………………………
Date de naissance: ……………………………………
Éducation: Secondaire = 11; CEGEP = 13
Baccalauréat = 16; Maitrise = 18; Doctorat = 20
Difficulté de langue?: ……………………………….

LANGAGE - Appellation

➲ Demandez: Veuillez dire ou écrire le nom des images suivantes:

Score 0-8 ☐

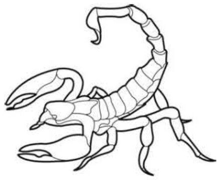
………………………… ☐

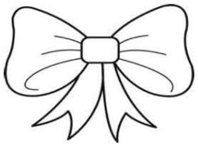
………………………… ☐

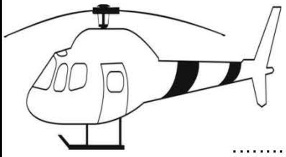
………………………… ☐

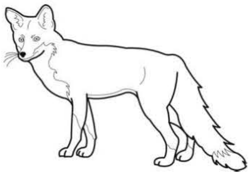
………………………… ☐

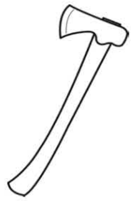
………………………… ☐

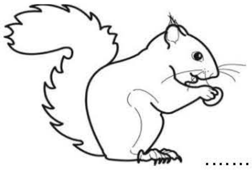
………………………… ☐

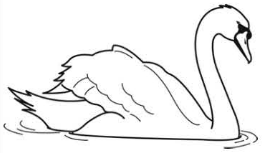
………………………… ☐

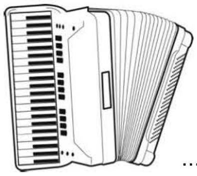
………………………… ☐

LANGAGE - Compréhension

➲ Demandez: pointez:

Score 0-8 ☐

1. Quelque chose dans laquelle vous pouvez [illegible]
2. Quelque chose avec des pattes palmées………………………
3. Un animal qui grimpe aux [illegible]
4. Quelque chose qu'on utilise pour découper……………………
5. Un moyen de [illegible]
6. Quelque chose avec une lame……………………………………
7. Quelque chose avec un dard…………………………………
8. Quelque chose qui mange des noix et des [illegible]

MÉMOIRE – Rappel immédiat

Score 0-10

➲ Dites: 'Je vais vous lire une histoire courte. Veuillez l'écouter attentivement. Lorsque j'ai fini, dites ou écrivez autant que vous le pouvez sur l'histoire.' Accordez 1 point pour chaque section soulignée (entière ou partielle) rappelée.

__Dimanche__ dernier, le __nettoyage annuel du parc__ a eu lieu dans __le bois Marigold__. __Quarante-deux__ personnes ont participé pour enlever les vieux __vélos et paniers d'épicerie__. __M. Robert Webber__ du __projet Woodland__ a déclaré aux journalistes locaux qu'il a été très __impressionné et particulièrement fier__ des __17 enfants__ qui sont venus.

Utiliser également ce score pour calculer le % de rétention plus tard

LANGAGE – Orthographe

Score 0-12

➲ Dites: 'Veuillez épeler à haute voix sur papier les mots suivants.' Si la personne utilise une technologie d'assistance, demandez-lui d'éteindre toute fonction prédictive.

1. Enveloppe..
2. Planche à roulettes…………………………………
3. Construction ....
4. Partenaire...
5. Biscuit..
6. Tondeuse à gazon..
7. Livrer………......................................
8. Enregistré...
9. Cintre..
10. Orchestre...
11. Tournevis ....
12. Apporté ....

FLUENCE VERBALE – Lettre S

☐ Oral ☐ Écrit

➲ Dites: 'Je vais vous donner une lettre de l'alphabet et j'aimerais que vous disiez ou écriviez autant de mots que vous le pouvez qui commencent par cette lettre. Les noms propres ou les chiffres ne sont pas acceptés.'
- Si parlé, dites: 'Vous avez une minute. La lettre est S.'
- Si écrit, dites: 'Vous avez deux minutes. La lettre est S.'

No. de mots corrects =

Temps pour lire/ copier =

Vfi =

➲ Ensuite, la personne copie ou lit les mots à haute voix.

- Si parlé, dites: 'Lisez ces mots à haute voix aussi vite que possible. Avant de faire cela, vérifiez que vous pouvez les lire. Je vais vous chronométrer. Prêt? Commencez.'
- Si écrit, dites: 'Copiez ces mots aussi vite que possible. Je vais vous chronométrer. Prêt? Commencez.'

Calcul "Verbal Fluency Index (Vfi)":

Si oral:
Vfi = 60 secondes – no. de secondes pour lire les mots à voix haute / No. de mots corrects générés

Si écrit:
Vfi = 120secondes – no. de secondes pour copier les mots / No. de mots corrects générés

Conversion VFI en tableau de score

ORAL VFI	ECRIT VFI	Score
≥ 12.00	≥ 20.00	0
10.00 to <12.00	16.50 to < 20.00	2
8.00 to < 10.00	13.00 to < 16.50	4
6.00 to < 8.00		
4.00 to < 6.00	9.50 to < 13.00	6
2.00 to < 4.00	6.00 to < 9.50	8
< 2.00	2.50 to < 6.00	10

Score 0-12

EXÉCUTIF – Empan de chiffres inversé

➲ Dites: 'Je vais vous donner une liste de chiffres et j'aimerais que vous me les répétiez dans l'ordre inversé. Par exemple, si je dis 2-3-4, vous devriez dire 4-3-2. Essayons avec 7-1-9, que me diriez-vous?' Arrêtez quand la personne se trompe aux deux essais de la même ligne. Notez le nombre total d'essais corrects.

Essai		Vérifier	Essai		Vérifier
1	2 6		2	5 8	
3	9 3 5		4	4 1 6	
5	7 2 8 4		6	9 5 7 3	
7	6 9 4 2 1		8	8 3 2 5 6	
9	8 1 3 5 7 9		10	3 6 2 7 3 4	
11	1 6 9 3 5 8 6		12	2 3 6 8 4 9 2	

Score 0-12 ☐

Longeur ☐

EXÉCUTIF – Alternance

➲ Dites: 'J'aimerais que vous alterniez entre chiffres et lettres, en commençant par 1A, puis 2B, 3C, et ainsi de suite. Veuillez alterner entre chiffres et lettres, dans l'ordre, sans en sauter, jusqu'à ce que je vous dise d'arrêter. Commençons ensemble : 1A, 2B, 3C… '

Essai		Vérifier	Essai		Vérifier	Essai		Vérifier	Essai		Vérifier
1	4-D		2	5-E		3	6-F		4	7-G	
5	8-H		6	9-I		7	10-J		8	11-K	
9	12-L		10	13-M		11	14-N		12	15-O	

Score 0-12 ☐

FLUENCE VERBALE – Lettre T

☐ Oral ☐ Écrit

➲ Dites: 'Je vais vous donner une lettre de l'alphabet et j'aimerais que vous disiez ou écriviez autant de mots que vous le pouvez qui commencent par cette lettre. Les noms propres ou les chiffres ne sont pas acceptés. Maintenant, le mot doit être long de quatre lettres. Ni plus, ni moins, que quatre lettres.
- Si oral, dites: 'Vous avez une minute. La lettre est T.'
- Si écrit, dites: 'Vous avez deux minutes. La lettre est T.'

No. de mots corrects =

Temps pour lire/ copier =

Vfi =

➲ Ensuite, la personne copie ou lit les mots à haute voix.

- Si parlé, dites: 'Lisez ces mots à haute voix aussi vite que possible. Avant de faire cela, vérifiez que vous pouvez les lire. Je vais vous chronométrer. Prêt? Commencez.'
- Si écrit, dites: 'Copiez ces mots aussi vite que possible. Je vais vous chronométrer. Prêt? Commencez.'

Calcul "Verbal Fluency Index (Vfi)":

Si oral:
Vfi = <u>60 secondes – no. de secondes pour lire les mots a voix haute</u>
No. de mots corrects générés

Si écrit:
Vfi = <u>120secondes – no. de secondes pour copier les mots</u>
No. de mots corrects générés

Conversion VFI en tableau de score

ORAL VFI	ÉCRIT VFI	Score
≥ 20.00	≥ 27.25	0
16.75 to < 20.00	23.00 to < 27.25	2
13.50 to < 16.75	18.75 to < 23.00	4
10.25 to < 13.50	14.50 to < 18.75	6
7.00 to < 10.25	10.25 to < 14.50	8
3.75 to < 7.00	6.00 to < 10.25	10
< 3.75	< 6.00	12

Score 0-12 ☐

VISUOSPATIAL – Compter les points

➲ Dites: ‘J’aimerais que vous comptiez le nombre de points dans chaque boîte, mais sans les pointer.’

Score
0-4

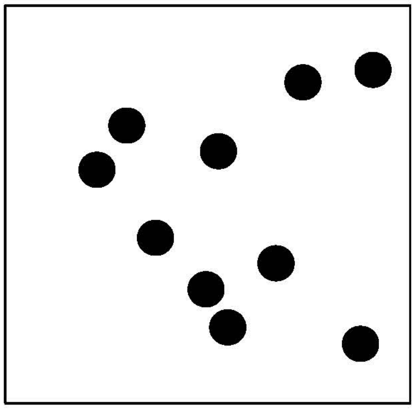

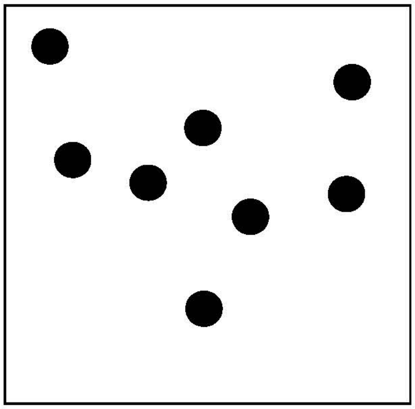

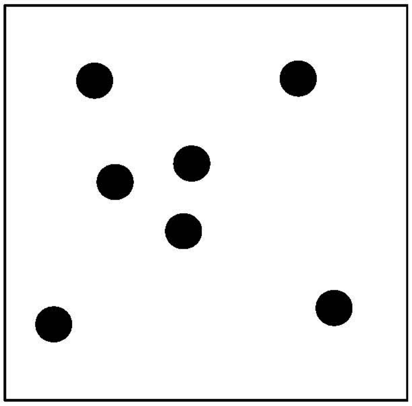

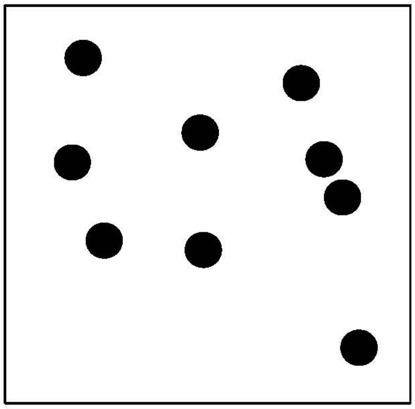

VISUOSPATIAL – Comptage de cubes

➲ Dites: ‘Combien de cubes y a-t-il dans chaque structure, incluant ceux que vous n’êtes pas capable de voir?’

Score
0-4

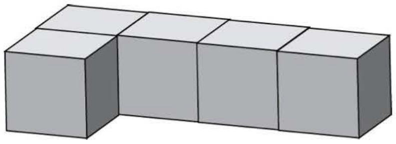

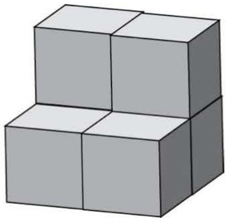

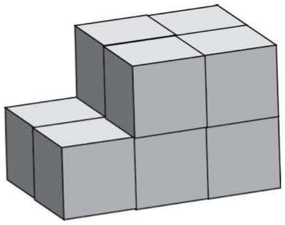

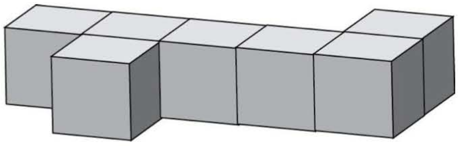

VISUOSPATIAL – Localisation de chiffres

➲ Dites: 'Quel chiffre correspond à la position du point?'

Score 0-4

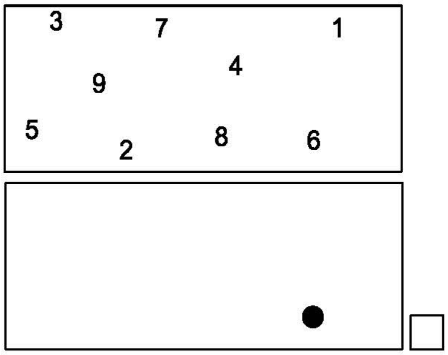

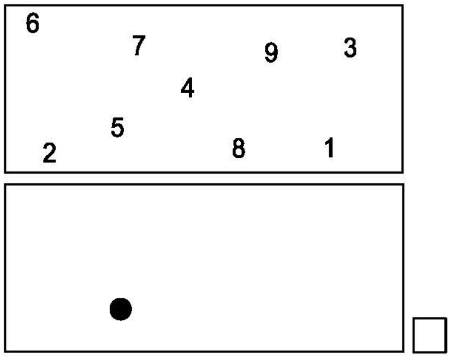

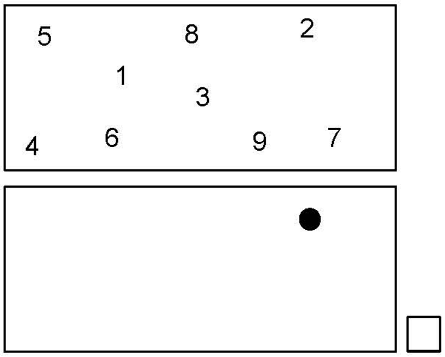

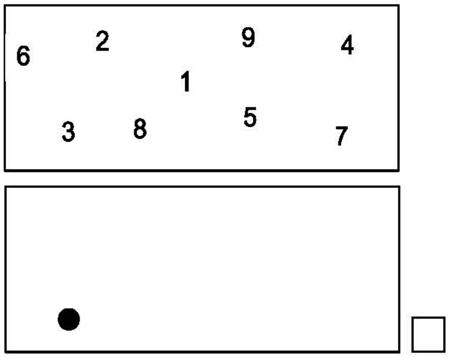

EXÉCUTIF – Complétion de phrase

➲ Dites: 'Écoutez attentivement ces phrases. Aussitôt que j'aurai fini de les lire, veuillez me dire, ou écrire, un mot qui finit la phrase aussi vite que possible. Par exemple: '*elle était si fatiguée qu'elle est allée directement au…lit*'. Ne pas donner de score.

1. Il a appelé le restaurant pour réserver une ....
2. Lorsqu'elle s'est réveillée le matin, le soleil était........................

➲ Dites: 'Maintenant j'aimerais que l'on recommence, mais cette fois-ci j'aimerais que le mot que vous donnerez ne fasse aucun sens dans le contexte de la phrase. Il ne doit pas être relié au mot qui complète correctement la phrase. Par exemple, `*John s'est coupé la main avec…une orange*'. Si la personne ne répond pas dans les 20 secondes, passez à la question suivante.

1. Le facteur a frappé à la ……………………...............................

2. Il a apporté son parapluie avec lui en cas de ....

3. Sally prépare son toast avec du beurre et ....

4. John est allé chez le barbier pour se faire .... les cheveux

5. Elle a plongé dans la ....

6. Ils sont tous allés au café local pour .... quelque chose

Donnez un score de 2 pour un mot different, 1 pour un mot différent mais relié (associé ou sens opposè) ou 0 pour le mot exacte.

Score
0-12

COGNITION SOCIALE – Partie A

➲ Dites: 'Vous allez voir quelques photos, une dans chaque coin d'une boîte. Vous devez choisir quelle photo vous préférez. Vous pouvez pointer ou dire quelle image vous préfèrez. Veuillez répondre aussi vite que possible.' Encerclez le choix du participant.

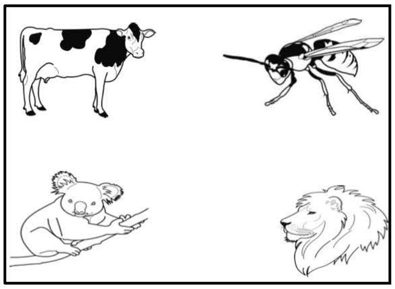

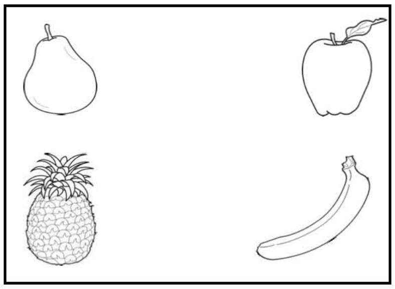

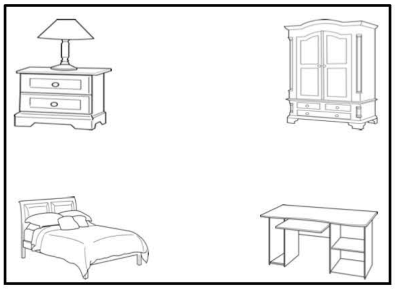

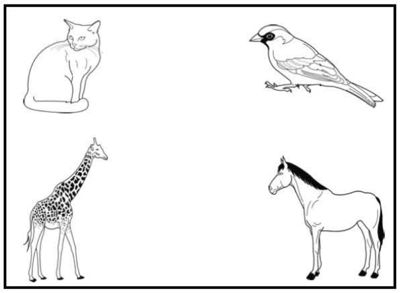

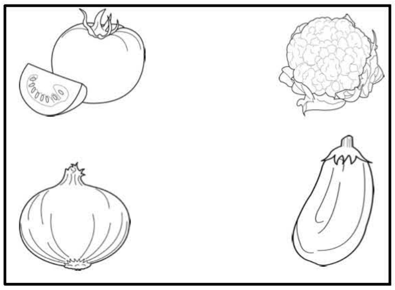

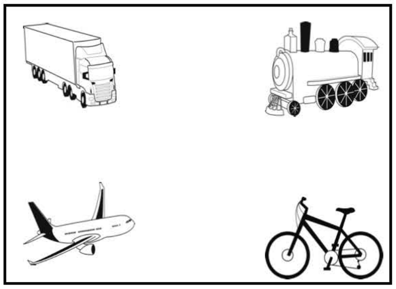

COGNITION SOCIALE – Partie B

➲ Dites: 'Vous allez voir quelques photos, une dans chaque coin d'une boîte. Vous devez choisir quelle photo le visage préfère. Vous pouvez pointer ou dire quelle image il préfère. Veuillez répondre aussi vite que possible.' Encercler le choix du participant. Items corrects = 2 points, erreur = 1 point, erreur égocentrique = 0 points.

Score 0-12

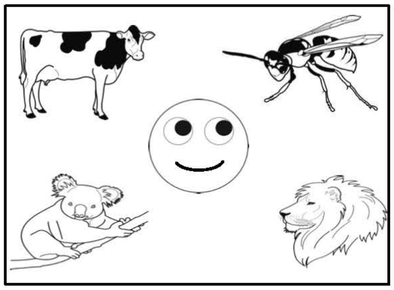

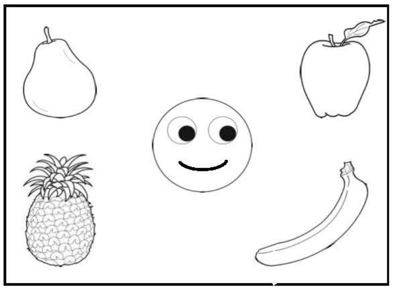

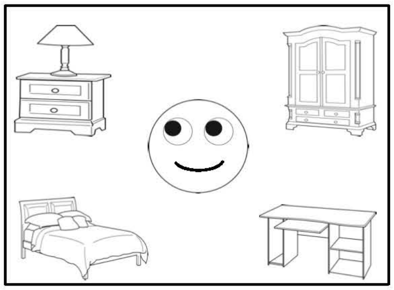

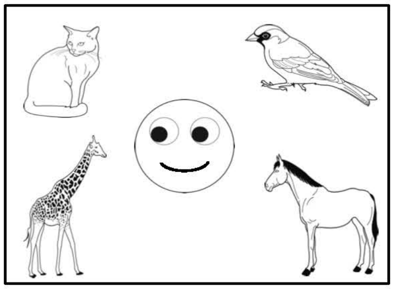

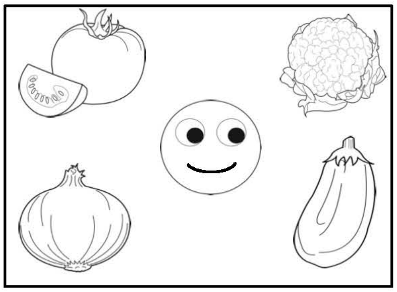

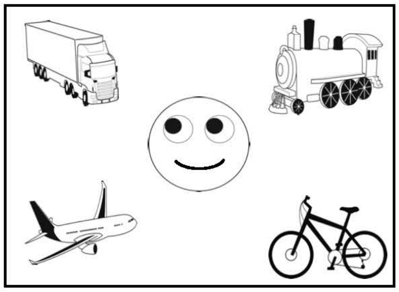

MÉMOIRE – Rappel retardé

Procédure de notation pour la rétention: obtenir un score de rappel différé (page suivante) et, avec le score de rappel immédiat, déterminer le pourcentage retenu. Convertir le pourcentage retenu en score de rétention. Si score = 0, score converti = 0.

Calcul du pourcentage de rappel retardé retenu

$$\frac{\text{(Score de rappel retardé)}}{\text{(Score de rappel immédiat)}} \times 100 = \%\ \text{retenu}$$

(..................................)
x 100 = ...% retenu
(..................................)

Tableau du pourcentage retenu et du score de rétention

Pourcentage retenu	**Score converti**	Pourcentage retenu	**Score converti**
1-10%	**1**	51-60%	**6**
11-20%	**2**	61-70%	**7**
21-30%	**3**	71-80%	**8**
31-40%	**4**	81-90%	**9**
41-50%	**5**	91-100+ %	**10**

➲ Dites: ‘Au début de cette entrevue, je vous ai lu une histoire courte. Dites-moi tout ce que vous pouvez vous rappeler sur cette histoire’. Accordez 1 point pour chaque section soulignée (entière ou partielle) rappelée.

Dimanche *dernier, le* ***nettoyage annuel du parc*** *a eu lieu dans* ***le bois Marigold***.
Quarante-deux *personnes ont participé à enlever les vieux* ***vélos et paniers d'épicerie***.
M. Robert Webber *du* ***projet Woodland*** *a déclaré aux journalistes locaux qu'il a été très* ***impressionné et particulièrement fier*** *des* ***17 enfants*** *qui sont venus.*

Score brut de rappel retardé (0-10) ☐

Score retenu converti (0-10) ☐

MÉMOIRE – Reconnaissance retardée

Si tous les éléments rappelés, passez et notez 4. Sinon, posez des questions ci-dessous.

Dites: ‘Voyons voir si vous pouvez vous rappeler de plus de détails sur cette histoire. Je vais vous poser quelques questions, répondez-moi par oui ou par non.’

Encerclez les réponses (vrai ou faux) et notez 1 point pour chaque item reconnu dans cette section. Utilisez le tableau ci-dessous pour calculer le score.

Score 0-4 ☐

L'histoire était-elle à propos d'un évènement s'étant déroulé samedi passé?	V	**F**	1
L'évènement était-il le nettoyage du parc?	**V**	F	1
L'histoire s'est-elle passée dans le bois Marigold?	**V**	F	1
Ont-ils enlevé des vieilles canettes de soda et des emballages de bonbons?	V	**F**	1
L'homme de l'histoire s'appelait-il Mr. Webber?	**V**	F	1
Son prénom était-il Thomas?	V	**F**	1
Était-il du conseil local?	V	**F**	1
Était-il particulièrement fier des enfants qui sont venus?	**V**	F	1

Oui = Vrai (V)
Non = Faux (F)

Tableau du score de reconnaissance de la reconnaissance	
No. De bonne réponses	**Score Converti**
0-4	**0**
5	**1**
6	**2**
7	**3**
8	**4**

SCORES

**Langage**	Appellation, Compréhension, Orthographe		/28
**Fluence verbale**	Aisance de langage Lettre S, Aisance de langage Lettre T		/24
**Executif**	Empan de chiffres inversé, Alternance, Complétion de phrase, Cognition sociale		/48
	**SLA-SPECIFIQUE:**		**/100**
Mémoire	*Rappel immédiat, Rappel retardé, Reconnaissance retardée*		*/24*
Visuospatial	*Compter les points, Comptage de cubes, Localisation de chiffres*		*/12*
	SLA NON-SPECIFIQUE:		***/36***
	**SCORE ECAS TOTAL:**		**/136**

S. Abrahams & T.H. Bak
Dot counting is reproduced with kind permission from J. R. Hodges

ECHELLE COGNITIVE ET COMPORTEMENTALE D'EDIMBOURG DE DÉPISTAGE DE LA SLA ECAS (Version Français Canada 2018)

Dépistage de comportement – Entretien avec aidant

➲ Veuillez interroger l'aidant au sujet des comportements possibles suivants. Les symptômes devraient avoir eu lieu de façon répétitive et non une fois seulement, et peuvent avoir eu lieu avant le développement d'un symptôme moteur quelconque. Cochez oui, non, ou ne sait pas. Si oui, décrivez brièvement. Donnez 1 point pour chaque réponse oui (maximum = 10).

**A**	**Désinhibition comportementale**			
1	Comportement socialement inapproprié, e.g.<br>*comportement inapproprié avec des étrangers*<br>*comportement criminel*	O	N	NSP
2	Perte de manières ou de décorum, e.g.<br>*remarques crues ou sexuellement explicites, blagues ou opinions pouvant être offensantes pour d'autres*<br>*manque de réponse face aux signaux sociaux*	O	N	NSP
3	Actions impulsives, imprudentes ou négligentes, e.g.<br>*Commence à faire des jeux de hasard, achète ou vend des propriétés sans considérer les conséquences, partage des informations personnelles de façon inappropriée (numéro de carte de crédit, etc.)*	O	N	NSP
**B**	**Apathie, Inertie**			
4	Perte d'intérêt ou de motivation , e.g.<br>*passivité, manque de spontanéité*<br>*besoin d'être poussé pour initier ou continuer des activités routinières*	O	N	NSP
**C**	**Perte de sympathie ou d'empathie**			
5	Diminution de la réponse aux besoins et sentiments des autres<br>*Un score positif à cette section devrait être basé sur des exemples spécifiques montrant un manque de compréhension ou une indifférence aux sentiments des autres.*<br>*Commentaires blessants*<br>*Ne tiens pas compte de la douleur ou la détresse des autres*	O	N	NSP
6	Diminution de l'intérêt social, de l'interrelation, de la chaleur ou de la proximité dans les interactions sociales, e.g.<br>*froideur*<br>*pas de contact visuel*	O	N	NSP
**D**	**Comportement de persévération, stéréotype, compulsif ou de rituel**			
7	Mouvements simples et répétés, e.g.<br>*tapotements, applaudissements*<br>*grattage, arrachage de peau ou de vêtements*<br>*répétition de mots*	O	N	NSP
8	Comportements complexes, compulsifs ou de rituel, e.g.<br>*Comptage, rituel de nettoyage, vérification*<br>*Collectionner, accumuler des objets*	O	N	NSP
**E**	**Frénésies alimentaires ou hyperoralité et changement de préférences alimentaires**			

COMPORTEMENT

9	Changement de préférences alimentaires, e.g.<br>*manies alimentaires (habitudes changeantes)*<br>*envies de glucides (particulièrement ceux sucrés)*	O	N	NSP	
10	Grignotage compulsif ou hyperoralité, e.g.,<br>*Boulimie ou continuer à manger même après satiété*<br>*exploration orale ou consommation d'objets non-comestibles*	O	N	NSP	
**SCORE**					
				**TOTAL**	*/10*

**SYMPTÔMES**	
➲ Veuillez cochez la case si au moins un des symptômes était présent dans chacune des catégories suivantes.	
**A. Désinhibition comportementale**	
**B. Apathie, Inertie**	
**C. Perte de sympathie ou d'empathie**	
**D. Comportement de persévération, stéréotype, compulsif ou de rituel**	
**E. Hyperoralité et changement de préférences alimentaires**	

Dépistages de psychose SLA

➲ Veuillez interroger l'aidant au sujet des symptômes possibles suivants. Cochez oui, non, ou ne sait pas. Si oui, décrivez brièvement. Donnez 1 point pour chaque réponse oui (maximum de 3).

1	A des comportements ou croyances bizarres	O	N	NSP
2	Entend ou voit des choses qui ne sont pas là, et/ou ressent la présence de quelqu'un qui n'est pas là	O	N	NSP
3	Est excessivement suspicieux, et/ou se sent persécuté	O	N	NSP
**SCORE**				
			**TOTAL**	*/3*

**DÉBUT ET DURÉE DES SYMPTÔMES**		
➲ Veuillez cocher ou compléter la case pour indiquer la réponse.		
**1. Est-ce que ces symptômes représentent un changement par rapport au comportement passé du patient?**	O	NSP
Si oui, les changements ont-ils eu lieu:		
a. AVANT le début de la maladie?	O	NSP
b. Au même moment?	O	NSP
c. APRÈS le début de la maladie?	O	NSP
**2. Ces symptômes persistent-ils?**	O	NSP
**3. Si non, combien de temps ont-ils duré?**		

Appendix 9.2.3: ECAS B [ENGLISH]

Found in [ECAS B (EN, V1.1, 2020) - CAPTURE ALS]

EDINBURGH COGNITIVE AND BEHAVIORAL ALS SCREEN – UNIVERSITY OF PENNSYLVANIA (ECAS-PENN)

North American English Version Form B, Version 1.1 Revised (2020)

Developed by Sharon Abrahams and Thomas H. Bak, University of Edinburgh

Adapted for use in North America by Katya Rascovsky, Corey McMillan, and Murray Grossman, University of Pennsylvania, in collaboration with Michael Benatar on behalf of the Clinical Research in ALS and Related Disorders for Therapeutic Development (CReATe) Consortium.

Date of testing: ....
Occupation: ....
....
Handedness: ....
Years of education: ....

Name/ID: ....
Date of Birth: ....
Highest Completed Degree:..................................
Premorbid language difficulties ....

LANGUAGE - Naming

➲ Ask: Say or write down the names of these pictures:

Score 0-8 ☐

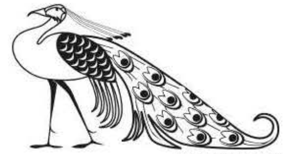
.... ☐

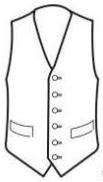
.... ☐

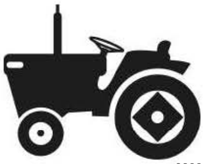
.... ☐

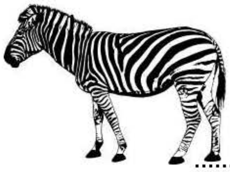
.... ☐

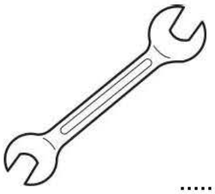
.... ☐

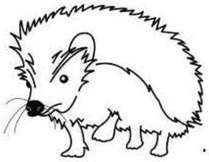
.... ☐

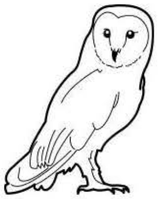
.... ☐

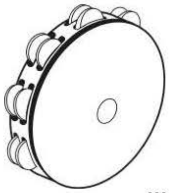
.... ☐

LANGUAGE - Comprehension

➲ Ask: Point or say the one which is:

Score 0-8 ☐

1. Something you wear......................................
2. An animal with colorful feathers...............................
3. Something you play..
4. A means of transportation.......................................
5. Something a plumber might use......................
6. Something that rolls into a ball...................................
7. Something used on a farm..............................
8. Something that lives in the African savannah.............

MEMORY – Immediate recall

➲ Say: 'I am going to read you a short story. Please listen carefully. When I am finished, say or write as much as you can remember'. Score according to the Administration and Guidance Notes.

Three fishing boats helped rescue a whale that swam too close to the shore. The whale was spotted swimming in circles. Alan Williams from the Marine Conservation Society said thirty-two young whales got lost last winter when looking for food.

Score 0-10

☐

Also use this score to calculate % retained (bottom p.7)

LANGUAGE - Spelling

➲ Say: 'Spell, either by speaking or writing, the following words.' If the person is using assistive technology, ask them to turn off any predictive text facility. Give one point on "watermelon", "earthquake", "toothpick", and "sunflower" if the person inserts a "space" when spelling the word.

1. Lecture	....	2. Toothpick	....
3. Gathering	....	4. Pollution	....
5. Receipt	....	6. Sunflower	....
7. Inspire	....	8. Argued	....
9. Watermelon	....	10. Dictionary	....
11. Earthquake	....	12. Thought	....

Score 0-12

☐

FLUENCY - Letter F ☐ Oral ☐ Written

➲ Say: 'I am going to give you a letter of the alphabet and I would like you to say or write as many different words as you can beginning with that letter, but not names of people or places, or numbers.'

- If speaking, say: 'You will have **one** minute. The letter is F.'
- If writing, say 'You will have **two** minutes. The letter is F.'

No. of correct words =

Time to read/ copy aloud =

Vfi =

➲ Next the person copies/reads these words aloud.

- If speaking, say: 'read aloud these words as fast as possible. Before you do this, check that you can read them. I will time you. Ready? Begin.'
- If writing, say: 'copy these words as fast as possible. I will time you. Ready? Begin.'

Verbal Fluency Index (Vfi) calculation:

If spoken:

Vfi = 60seconds – no. of seconds to read aloud words / No. of correct words generated

If written:

Vfi = 120seconds – no. of seconds to copy words / No. of correct words generated

VFI conversion to score table

SPOKEN VFI	WRITTEN VFI	Score
≥ 12.00	≥ 20.00	0
10.00 to < 12.00	16.50 to < 20.00	2
8.00 to < 10.00	13.00 to < 16.50	4
6.00 to < 8.00	9.50 to < 13.00	6
4.00 to < 6.00	6.00 to < 9.50	8
2.00 to < 4.00	2.50 to < 6.00	10
< 2.00	< 2.50	12

Score 0-12

☐

EXECUTIVE – Reverse Digit Span

➲ Say: 'I am going to say some numbers and I would like you to say them back to me in reverse order. For example, if I say '2 3 4', you should say '4 3 2'. Let's practice. If I say '7 1 9', what would you say?' Stop when person gets both trials of a line wrong. Score total number of trials correct. After each pair of trials remind the participant 'Now the number of items will increase'.

Score 0-12

Trial		Check	Trial		Check
**1**	2 8		**2**	4 9	
**3**	5 1 3		**4**	8 2 4	
**5**	1 5 2 7		**6**	6 3 8 1	
**7**	9 4 3 8 6		**8**	2 4 1 9 7	
**9**	1 8 3 5 4 2		**10**	7 3 9 2 6 1	
**11**	4 8 2 6 8 3 1		**12**	5 1 6 4 2 9 3	

EXECUTIVE – Alternation

➲ Say: 'I want you to alternate between numbers and letters, starting with 1-A, then 2-B, 3-C, and so on. Please continue from there, alternating between numbers and letters, in order, without skipping any until I tell you to stop. Let's begin together: 1-A, 2-B, 3-C…'

Score 0-12

Trial		Check	Trial		Check	Trial		Check	Trial		Check
**1**	4-D		**2**	5-E		**3**	6-F		**4**	7-G	
**5**	8-H		**6**	9-I		**7**	10-J		**8**	11-K	
**9**	12-L		**10**	13-M		**11**	14-N		**12**	15-O	

FLUENCY - Letter D ☐ Oral ☐ Written

➲ Say: 'I am going to give you a letter of the alphabet and I would like you to say or write as many different words as you can beginning with that letter, but not names of people or places, or numbers. This time the word must only be **four letters** long. No more or less than four letters'
- If speaking, say 'You will have **one** minute. The letter is D.'
- If writing, say: 'You will have **two** minutes. The letter is D.'

No. of correct words =

Time to read/ copy aloud =

Vfi =

➲ Next the person copies/reads these words aloud.
- If speaking, say: 'read aloud these words as fast as possible. Before you do this, check that you can read them. I will time you. Ready? Begin.'
- If writing, say: 'copy these words as fast as possible. I will time you. Ready? Begin.'

Verbal Fluency Index (Vfi) calculation:

If spoken:
Vfi = 60seconds – no. of seconds to read aloud words / No. of correct words generated

If written:
Vfi = 120seconds – no. of seconds to copy words / No. of correct words generated

VFI conversion to score table

SPOKEN VFI	WRITTEN VFI	**Score**
≥ 20.00	≥ 35.00	**0**
16.75 to < 20.00	28.50 to < 35.00	**2**
13.50 to < 16.75	22.00 to < 28.50	**4**
10.25 to < 13.50	15.50 to < 22.00	**6**
7.00 to < 10.25	9.00 to < 15.50	**8**
3.75 to < 7.00	2.50 to < 9.00	**10**
< 3.75	< 2.50	**12**

Score 0-12

VISUOSPATIAL – Dot Counting

➲ Say: 'I would like you to count how many dots are in each box, but without pointing to them.'

Score 0-4

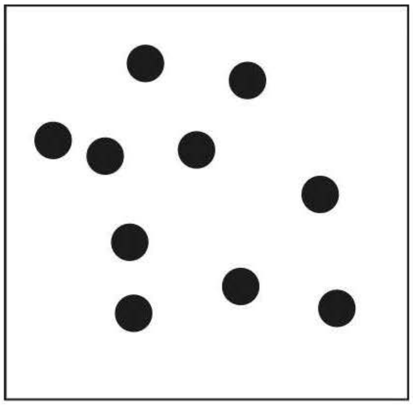

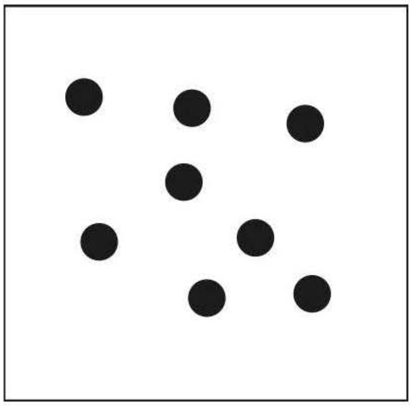

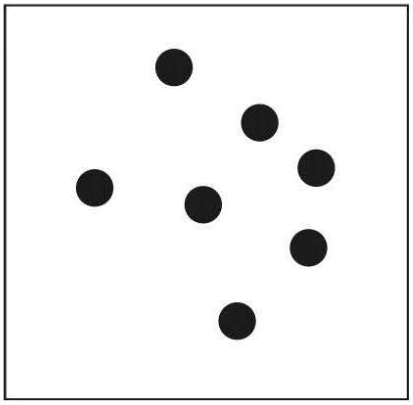

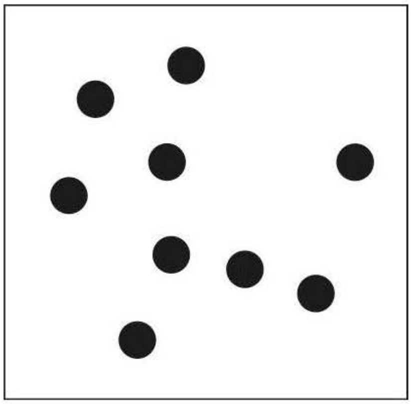

VISUOSPATIAL – Cube Counting

➲ Say: 'How many cubes are in each structure, including the ones you may not be able to see?'

Score 0-4

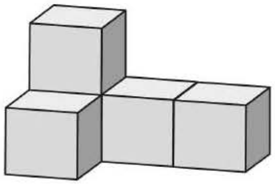

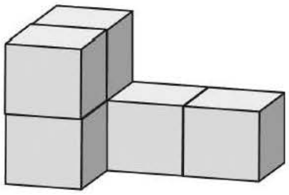

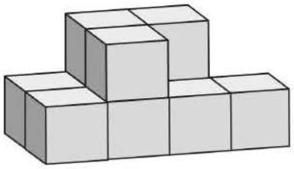

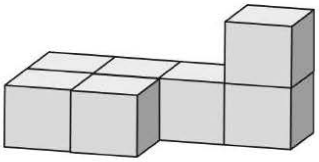

**VISUOSPATIAL – Number Location**	
➲ Say: 'Which number corresponds to the position of the dot?'	Score 0-4

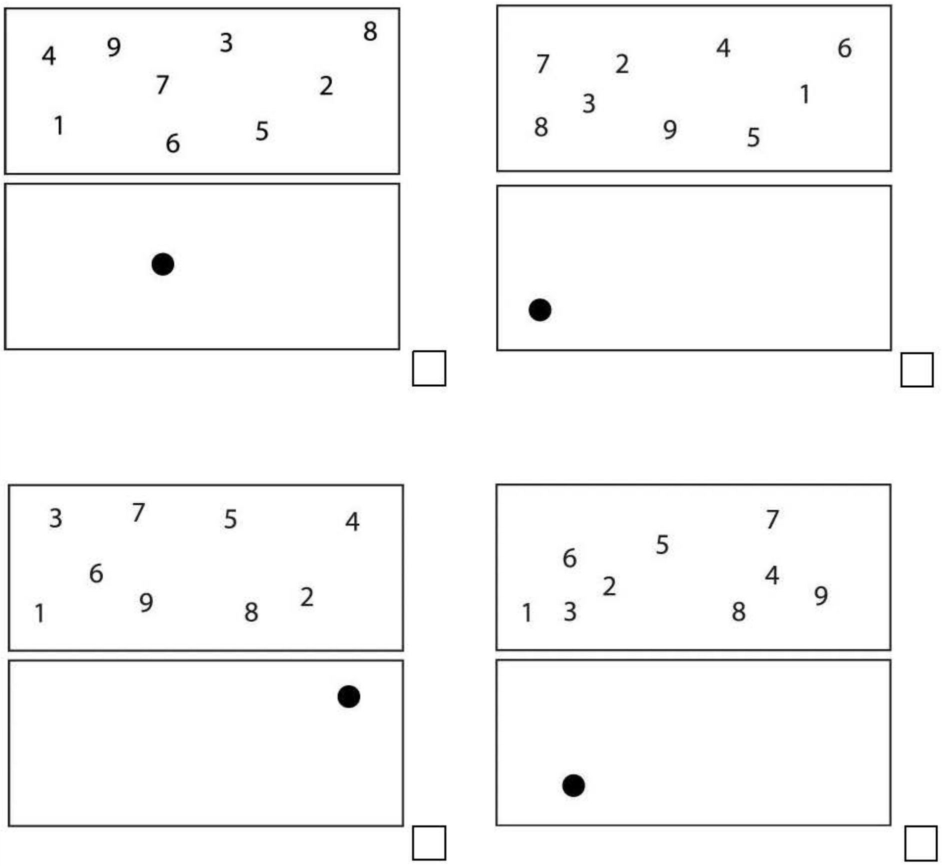

**EXECUTIVE – Sentence Completion**	

➲ Say: 'Listen carefully to these sentences and as soon as I have finished reading them, please tell me, or write, a word that finishes the sentence as quickly as possible. For example, '*She was so tired that she went straight to…bed*'. Do not score.

1. He called up the restaurant to reserve a ....
2. When she got up in the morning, the sun was....................

➲ Say: 'Now I'd like you to do that again, but this time the word you give should not make sense whatsoever in the context of the sentence. It must not be related to the word that actually completes the sentence. For example, '*John cut his hand with the sharp…orange*'. If the person does not respond within 20 seconds, move onto the next question.

	Score 0-12
1. She answered the phone because it was	
2. The joke was so funny, he started to	
3. Daniel unlocked the door with a	
4. The child cut paper with a pair of	
5. After months of practice, Lisa passed her driving	
6. Simon ate his dinner with a knife and	
**Give 2 points for different word, 1 point for related word (e.g. associated or opposite meaning) or 0 points for exact word.**	

SOCIAL COGNITION – Part A

➲ Say: 'You are going to see some pictures, one in each corner of a box. You have to choose **which picture you like best.** Either point to or say which picture you like best. Please respond as quickly as possible.' Circle participant's choice.

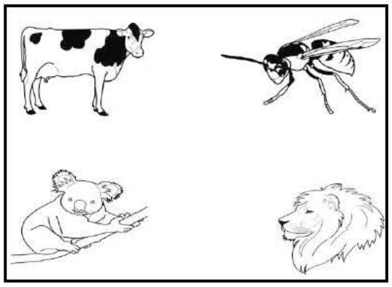

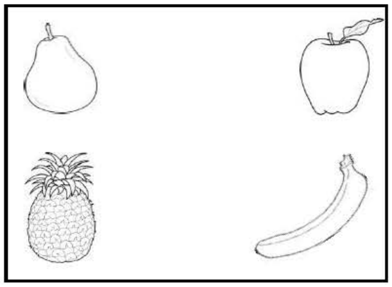

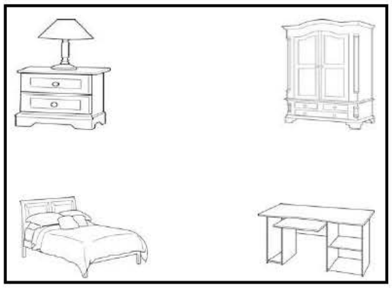

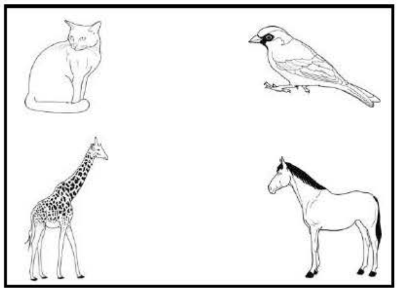

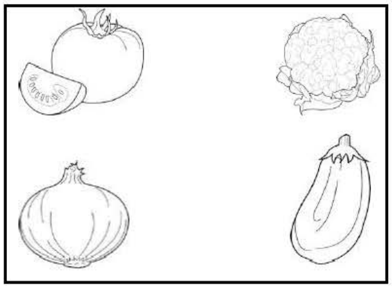

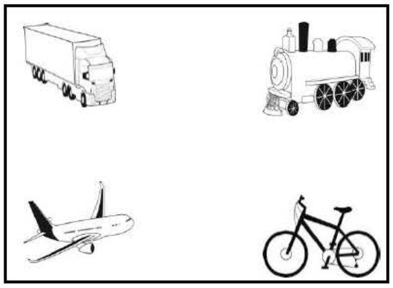

SOCIAL COGNITION – Part B

➲ Say: 'You are going to see some pictures, one in each corner of a box. You have to choose **which picture the face likes best.** Either point to or say which picture **the face likes best**. Please respond as quickly as possible.' Circle participant's choice. Correct items = 2 points, error = 1 point, egocentric error = 0 points.

Score 0-12

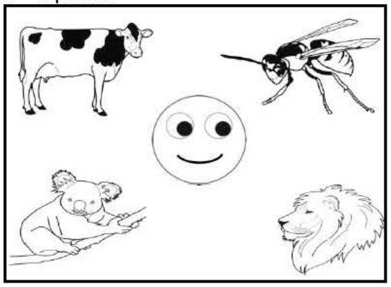

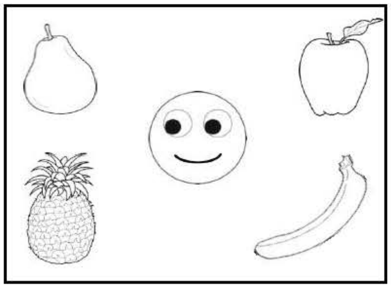

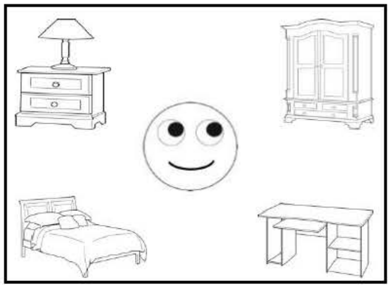

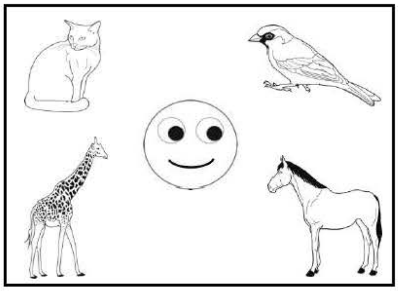

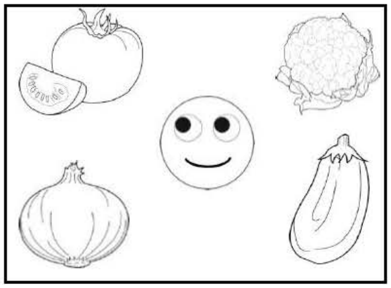

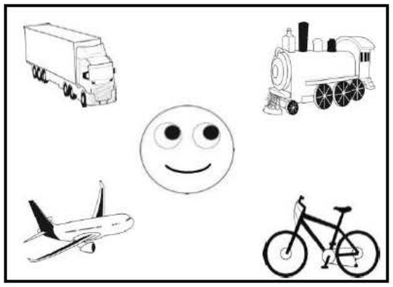

MEMORY – Delayed Recall

Scoring procedure for retention: obtain delayed recall performance (page 8) and, together with immediate recall score (page 2), determine percentage retained. Convert percentage retained to Score using table below. If delayed recall = 0, score = 0.

Delayed recall to percentage retained calculation

(Delayed recall score [page 8]) / (Immediate recall score [page 2[) *x 100 = % retained*

(..................................) / (..................................) *x 100 = ...% retained*

Percentage retained to converted score table

Percentage retained	Score	Percentage retained	Score
1-10%	1	51-60%	**6**
11-20%	2	61-70%	**7**
21-30%	3	71-80%	**8**
31-40%	4	81-90%	**9**
41-50%	5	91-100+ %	**10**

➲ Say: 'At the beginning of this interview, I read you a short story. Tell me as much as you can remember from that story'. Score according to the Administration and Guidance Notes.<br>*Three fishing boats helped rescue a whale that swam too close to the shore. The whale was spotted swimming in circles. Alan Williams from the Marine Conservation Society said thirty-two young whales got lost last winter when looking for food.*	Delayed recall raw score (0-10) ☐<br>**Converted retention score (0-10)** ☐

MEMORY – Delayed Recognition

If all items recalled, skip and score 4. Otherwise ask questions below.

Say: 'Let's see if you can remember anything more about that story. I will ask you some questions, please tell me if they are true or false'.

Circle responses (true or false) and score 1 point for each item recognized in this section. Use table below to calculate score.

Score 0-4 ☐

Were there four fishing boats?	T	**F**	1
Was the story about rescuing dolphins?	T	**F**	1
Did this take place close to the shore?	**T**	F	1
Was the animal swimming in circles?	**T**	F	1
Was the man in the story called Mr. Williams?	**T**	F	1
Was his first name Stephen?	T	**F**	1
Were thirty-two whales lost last Summer?	T	**F**	1
Were the lost whales looking for food?	**T**	F	1

Yes = True (T)
No = False (F)

Recognition to recognition score table

Number of correct answers	**Score**
0-4	**0**
5	**1**
6	**2**
7	**3**
8	**4**

SCORES

**Language**	Naming, Comprehension, Spelling	/28
**Verbal Fluency**	Fluency Letter F, Fluency Letter D	/24
**Executive**	Reverse Digit Span, Alternation, Sentence Completion, Social Cognition	/48
	**ALS-SPECIFIC:**	**/100**
Memory	*Immediate recall, Delayed recall score, Delayed recognition*	*/24*
Visuospatial	*Dot Counting, Cube Counting, Number Location*	*/12*
	ALS NON-SPECIFIC:	***/36***
	**ECAS TOTAL SCORE:**	**/136**

S. Abrahams & T. H. Bak
Dot counting is reproduced with kind permission from J. R. Hodges

EDINBURGH COGNITIVE AND BEHAVIORAL ALS SCREEN – UNIVERSITY OF PENNSYLVANIA (ECAS-PENN)
American English Form A, Version 1 (2018)

BEHAVIOR SCREEN – Caregiver Interview

➲ Please ask the caregiver about the following possible behaviors. Symptoms should have occurred repeatedly and not just on one instance, and may have occurred prior to the development of any motor signs. Tick 'Yes', 'No' or 'Don't Know'. If 'Yes', please provide a brief written description. Give one point for every 'Yes' response (maximum = 10).

**A**	**Behavioral disinhibition**				
1	Socially inappropriate behavior, e.g.<br>*inappropriate behavior with strangers*<br>*criminal behavior*	Y	N	DK	BEHAVIOR
2	Loss of manners or decorum, e.g.<br>*crude or sexually explicit remarks, jokes or opinions that may be offensive to others*<br>*lack of response to social cues*	Y	N	DK	
3	Impulsive, rash or careless actions, e.g.<br>*new onset gambling, or buying or selling property without regard for consequences*<br>*giving out personal information inappropriately, e.g. credit card numbers*	Y	N	DK	
**B**	**Apathy or inertia**				
4	Loss of interest, drive or motivation, e.g.<br>*passivity and lack of spontaneity*<br>*needs prompting to initiate or continue routine activities*	Y	N	DK	
**C**	**Loss of sympathy or empathy**				
5	Diminished response to other people's needs and feelings<br>*Positive rating on this feature should be based on specific examples that reflect a lack of understanding or indifference to other people's feelings, e.g*<br>*hurtful comments*<br>*disregard for others' pain or distress*	Y	N	DK	
6	Diminished social interest, interrelatedness, personal warmth or general closeness in social engagement, e.g.<br>*coldness*<br>*lack of eye contact*	Y	N	DK	
**D**	**Perseverative, stereotyped, compulsive or ritualistic behavior**				
7	Simple repetitive movements, e.g.<br>*tapping, clapping*<br>*scratching, picking skin or clothing*<br>*repeating words*	Y	N	DK	
8	Complex, compulsive or ritualistic behaviors, e.g.<br>*counting, cleaning rituals, checking*<br>*collecting, hoarding*	Y	N	DK	

E	**Hyperorality and altered food preferences**				
9	Altered food preferences, e.g.<br>*food fads*<br>*carbohydrate craving (particularly sweets)*	Y	N	DK	
10	Binge eating or hyperorality, e.g.,<br>*cramming or continuing to eat despite satiety*<br>*oral exploration or consumption of inedible objects*	Y	N	DK	
**SCORE**					
				**TOTAL**	*/10*
**SYMPTOMS**					
	➲ Please check box if at least one of the symptoms was present in each of the following categories.				
**A. Behavioral disinhibition**					
**B. Apathy or inertia**					
**C. Loss of sympathy or empathy**					
**D. Perseverative, stereotyped, compulsive or ritualistic behaviour**					
**E. Hyperorality and altered food preferences**					

**ALS Psychosis Screen**				
	➲ Please ask the caregiver about the following possible symptoms. Check 'Yes', 'No' or 'Don't Know'. If 'Yes', please provide a brief written description. Give one point for every 'Yes' response (maximum = 3).			
1	Has strange and/or bizarre beliefs and behaviors	Y	N	DK
2	Hears or sees things that are not there, and/or feels the presence of someone who is not there	Y	N	DK
3	Is overly suspicious, and/or feels persecuted	Y	N	DK
**SCORE**				
			**TOTAL**	*/3*

**ONSET AND DURATION OF SYMPTOMS**		
➲ Please check or complete box to indicate response.		
**1. Do these symptoms represent a CHANGE from the patient's previous behavior?**	Y	N
If yes, did the changes occur:		
a. BEFORE the onset of the disease?	Y	N
b. at the same time as other symptoms?	Y	N
c. AFTER the onset of the disease?	Y	N
**2. Do they still persist?**	Y	N
**3. If not, how long did they last?**		

Appendix 9.2.4: ECAS B [FRENCH]
Found in [ECAS B (FR, 16May2020) - CAPTURE ALS]

ECHELLE COGNITIVE ET COMPORTEMENTALE D'EDIMBOURG DE DÉPISTAGE DE LA SLA
ECAS Formulaire B (Version Français Canada 2020)
Developed by S. Abrahams and T.H. Bak, University of Edinburgh
Adapted for use in North America by K. Rascovsky and M. Grossman, University of Pennsylvania

Date d'essai: ....
Occupation: ....
....
Main Dominante:...
Années d'éducation:... … … … … … … … … … … …... .

Nom/ID: ....
Date de naissance: ....
Éducation: Secondaire = 11; CEGEP = 13
Baccalauréat = 16; Maitrise = 18; Doctorat = 20
Difficulté de langage?......................................

LANGAGE - Appellation

➲ Demandez: Veuillez dire ou écrire le nom des images suivantes:

Score 0-8 ☐

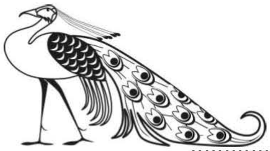
.... ☐

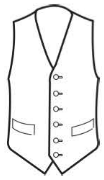
.... ☐

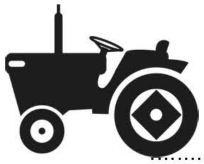
.... ☐

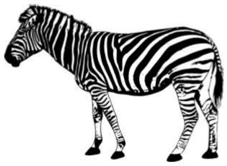
.... ☐

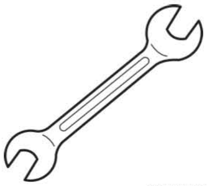
.... ☐

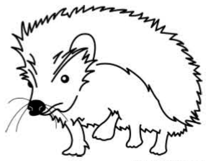
.... ☐

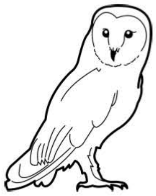
.... ☐

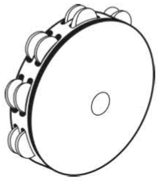
.... ☐

LANGAGE - Compréhension

➲ Demandez: pointez:

Score 0-8 ☐

1. Quelque chose que vous portez …………………......
2. Un animal avec des plumes colorées ………...……........
3. Quelque chose que vous jouez …………………........
4. Un moyen de transport …………………………………….
5. Quelque chose qu'un plombier pourrait utiliser ……..
6. Quelque chose qui se roule en boule …………..……...
7. Quelque chose qu'on utilise dans une ferme ……….
8. Quelque chose qui vit dans la savane africaine …………

MÉMOIRE – Rappel immédiat

Score 0-10 ☐

Utiliser également ce score pour calculer le % de rétention plus tard

➲ Dites: 'Je vais vous lire une histoire courte. Veuillez l'écouter attentivement. Lorsque j'ai fini, dites ou écrivez autant que vous le pouvez sur l'histoire.' Accordez 1 point pour chaque section soulignée (entière ou partielle) rappelée.

*<u>**Trois**</u> <u>**bateaux de pêche**</u> ont <u>**aidé à sauver**</u> une <u>**baleine**</u> qui nageait trop près du <u>**rivage**</u>. La baleine a été aperçue en train de nager en <u>**cercles**</u>. <u>**Alain Williams**</u>, de la <u>**Société de conservation marine**</u> a affirmé que <u>**trente-deux**</u> jeunes baleines se sont perdues <u>**l'hiver passé**</u> en cherchant de la nourriture.*

LANGAGE – Orthographe

➲ Dites: 'Veuillez épeler à haute voix sur papier les mots suivants.' Si la personne utilise une technologie d'assistance, demandez-lui d'éteindre toute fonction prédictive.

Score 0-12 ☐

1. Lecture ....
2. Cure-dents ………………………………………….……
3. Rassemblement...
4. Pollution ....
5. Reçu ....
6. Tournesol ....
7. Inspirer ……...
8. Argumenté ……………….…………………….…......
9. Melon d'eau ....
10. Dictionnaire …..
11. Tremblement de terre ………………………...
12. Pensée …...

FLUENCE VERBALE – Lettre F

☐ Oral ☐ Écrit

➲ Dites: 'Je vais vous donner une lettre de l'alphabet et j'aimerais que vous disiez ou écriviez autant de mots que vous le pouvez qui commencent par cette lettre. Les noms propres ou les chiffres ne sont pas acceptés.'

- Si parlé, dites: 'Vous avez une minute. La lettre est F.'
- Si écrit, dites: 'Vous avez deux minutes. La lettre est F.'

No. de mots corrects =

Temps pour lire/ copier =

Vfi =

➲ Ensuite, la personne copie ou lit les mots à haute voix.

- Si parlé, dites: 'Lisez ces mots à haute voix aussi vite que possible. Avant de faire cela, vérifiez que vous pouvez les lire. Je vais vous chronométrer. Prêt? Commencez.'
- Si écrit, dites: 'Copiez ces mots aussi vite que possible. Je vais vous chronométrer. Prêt? Commencez.'

Calcul "Verbal Fluency Index (Vfi)":

Si oral:
Vfi = <u>60 secondes – no. de secondes pour lire les mots à voix haute</u>
No. de mots corrects générés

Si écrit:
Vfi = <u>120secondes – no. de secondes pour copier les mots</u>
No. de mots corrects générés

Conversion VFI en tableau de score

ORAL VFI	ECRIT VFI	**Score**
≥ 12.00	≥ 20.00	**0**
10.00 to <12.00	16.50 to < 20.00	**2**
8.00 to < 10.00		
6.00 to < 8.00	13.00 to < 16.50	**4**
4.00 to < 6.00	9.50 to < 13.00	**6**
2.00 to < 4.00	6.00 to < 9.50	**8**
< 2.00	2.50 to < 6.00	**10**

Score 0-12 ☐

EXÉCUTIF – Empan de chiffres inversé

➲ Dites: 'Je vais vous donner une liste de chiffres et j'aimerais que vous me les répétiez dans l'ordre inversé. Par exemple, si je dis 2-3-4, vous devriez dire 4-3-2. Essayons avec 7-1-9, que me diriez-vous?' Arrêtez quand la personne se trompe aux deux essais de la même ligne. Notez le nombre total d'essais corrects.

**Essai**		Vérifier	**Essai**		Vérifier
**1**	2 6		**2**	5 8	
**3**	9 3 5		**4**	4 1 6	
**5**	7 2 8 4		**6**	9 5 7 3	
**7**	6 9 4 2 1		**8**	8 3 2 5 6	
**9**	8 1 3 5 7 9		**10**	3 6 2 7 3 4	
**11**	1 6 9 3 5 8 6		**12**	2 3 6 8 4 9 2	

Score 0-12 ☐

Longeur ☐

EXÉCUTIF – Alternance

➲ Dites: 'J'aimerais que vous alterniez entre chiffres et lettres, en commençant par 1A, puis 2B, 3C, et ainsi de suite. Veuillez alterner entre chiffres et lettres, dans l'ordre, sans en sauter, jusqu'à ce que je vous dise d'arrêter. Commençons ensemble : 1A, 2B, 3C...'

**Essai**		Vérifier	**Essai**		Vérifier	**Essai**		Vérifier	**Essai**		Vérifier
**1**	4-D		**2**	5-E		**3**	6-F		**4**	7-G	
**5**	8-H		**6**	9-I		**7**	10-J		**8**	11-K	
**9**	12-L		**10**	13-M		**11**	14-N		**12**	15-O	

Score 0-12 ☐

FLUENCE VERBALE – Lettre D ☐ Oral ☐ Écrit

➲ Dites: 'Je vais vous donner une lettre de l'alphabet et j'aimerais que vous disiez ou écriviez autant de mots que vous le pouvez qui commencent par cette lettre. Les noms propres ou les chiffres ne sont pas acceptés. Maintenant, le mot doit être long de quatre lettres. Ni plus, ni moins, que quatre lettres.

- Si oral, dites: 'Vous avez une minute. La lettre est D.'
- Si écrit, dites: 'Vous avez deux minutes. La lettre est D.'

No. de mots corrects =

Temps pour lire/ copier =

Vfi =

➲ Ensuite, la personne copie ou lit les mots à haute voix.

- Si parlé, dites: 'Lisez ces mots à haute voix aussi vite que possible. Avant de faire cela, vérifiez que vous pouvez les lire. Je vais vous chronométrer. Prêt? Commencez.'
- Si écrit, dites: 'Copiez ces mots aussi vite que possible. Je vais vous chronométrer. Prêt? Commencez.'

Calcul "Verbal Fluency Index (Vfi)":

Si oral:
Vfi = <u>60 secondes – no. de secondes pour lire les mots a voix haute</u>
No. de mots corrects générés

Si écrit:
Vfi = <u>120secondes – no. de secondes pour copier les mots</u>
No. de mots corrects générés

Conversion VFI en tableau de score

ORAL VFI	ÉCRIT VFI	**Score**
≥ 20.00	≥ 27.25	**0**
16.75 to < 20.00	23.00 to < 27.25	**2**
13.50 to < 16.75	18.75 to < 23.00	**4**
10.25 to < 13.50	14.50 to < 18.75	**6**
7.00 to < 10.25	10.25 to < 14.50	**8**
3.75 to < 7.00	6.00 to < 10.25	**10**
< 3.75	< 6.00	**12**

Score 0-12 ☐

VISUOSPATIAL – Compter les points

➲ Dites: ‘J’aimerais que vous comptiez le nombre de points dans chaque boîte, mais sans les pointer.’

Score
0-4

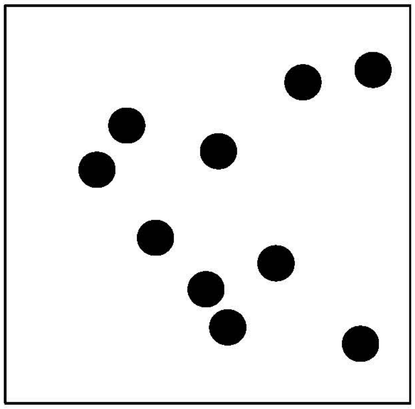

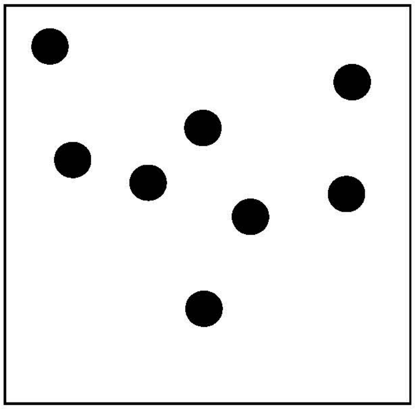

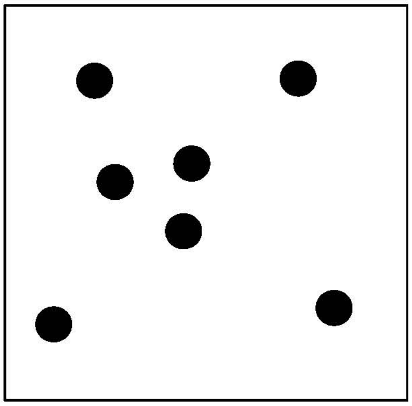

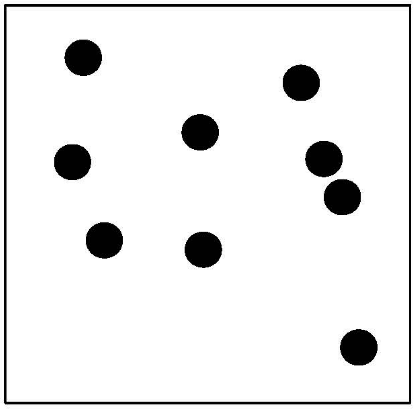

VISUOSPATIAL – Comptage de cubes

➲ Dites: ‘Combien de cubes y a-t-il dans chaque structure, incluant ceux que vous n’êtes pas capable de voir?’

Score
0-4

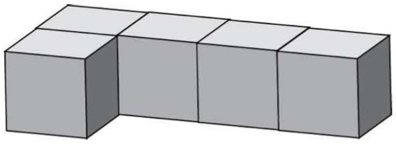

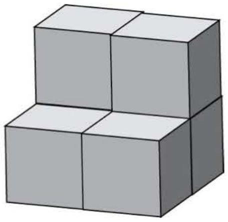

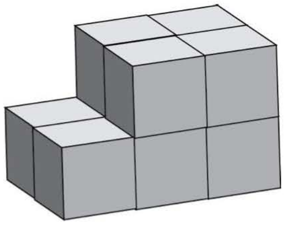

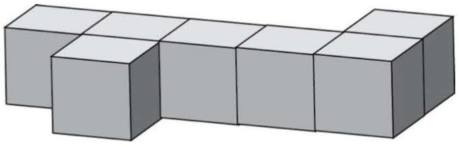

VISUOSPATIAL – Localisation de chiffres

➲ Dites: 'Quel chiffre correspond à la position du point?'

Score 0-4

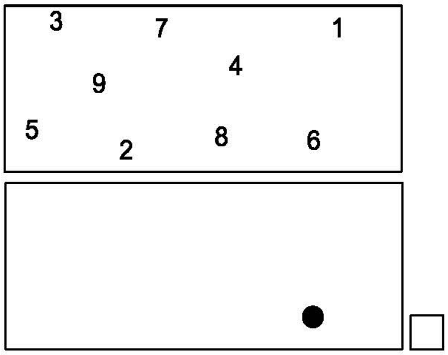

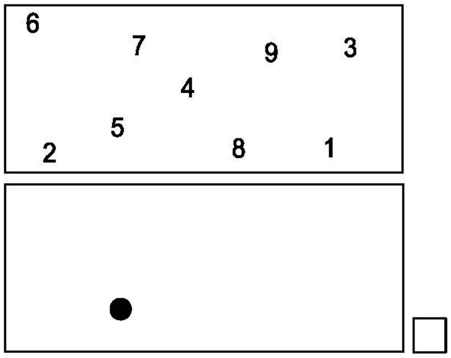

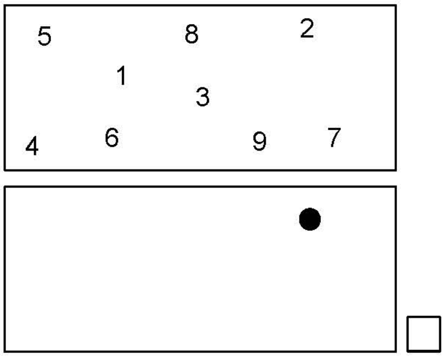

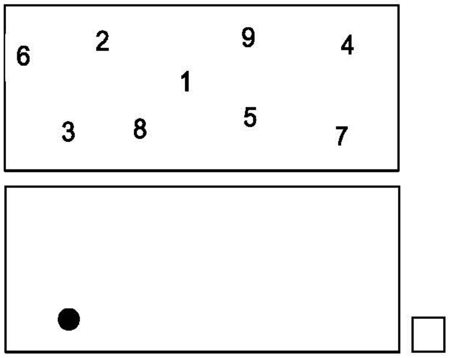

EXÉCUTIF – Complétion de phrase

➲ Dites: 'Écoutez attentivement ces phrases. Aussitôt que j'aurai fini de les lire, veuillez me dire, ou écrire, un mot qui finit la phrase aussi vite que possible. Par exemple: '*elle était si fatiguée qu'elle est allée directement au…lit*'. Ne pas donner de score.

1. Il a appelé le restaurant pour réserver une ....
2. Lorsqu'elle s'est réveillée le matin, le soleil était........................

➲ Dites: 'Maintenant j'aimerais que l'on recommence, mais cette fois-ci j'aimerais que le mot que vous donnerez ne fasse aucun sens dans le contexte de la phrase. Il ne doit pas être relié au mot qui complète correctement la phrase. Par exemple, '*John s'est coupé la main avec…une orange*'. Si la personne ne répond pas dans les 20 secondes, passez à la question suivante.

1. Elle a répondu au téléphone parce qu'il ………………..
2. La blague était si drôle qu'il a commencé à …………………….......................
3. Daniel a déverrouillé la porte avec une ………………...
4. L'enfant a coupé le papier avec une paire de …………………………………….
5. Après plusieurs mois d'entraînement, Lisa a passé son ………………………………..… de conduite
6. Simon a mangé son dîner avec un couteau et …………………………………….

Donnez un score de 2 pour un mot different, 1 pour un mot différent mais relié (associé ou sens opposè) ou 0 pour le mot exacte.

Score
0-12

COGNITION SOCIALE – Partie A

➲ Dites: 'Vous allez voir quelques photos, une dans chaque coin d'une boîte. Vous devez choisir quelle photo vous préférez. Vous pouvez pointer ou dire quelle image vous préfèrez. Veuillez répondre aussi vite que possible.' Encerclez le choix du participant.

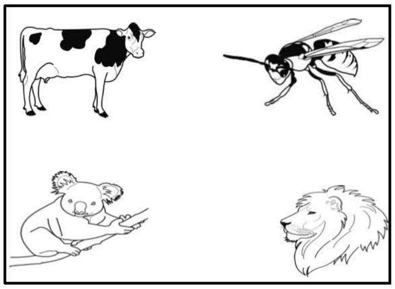
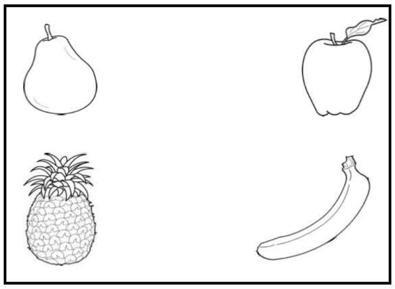
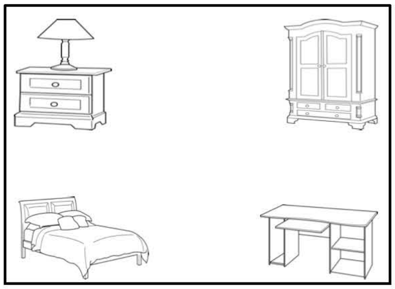
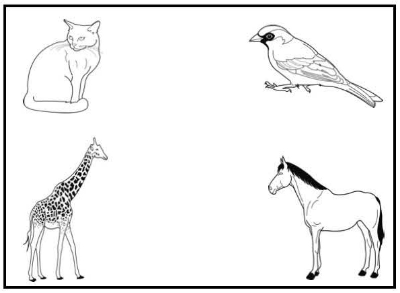
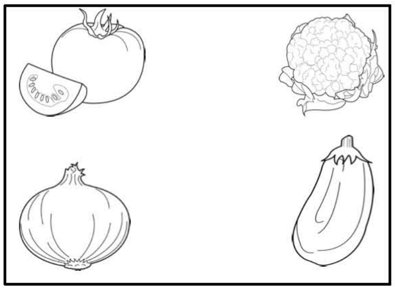
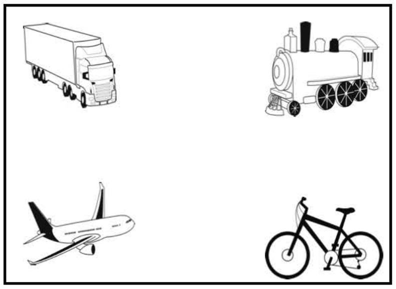

COGNITION SOCIALE – Partie B

➲ Dites: 'Vous allez voir quelques photos, une dans chaque coin d'une boîte. Vous devez choisir quelle photo le visage préfère. Vous pouvez pointer ou dire quelle image il préfère. Veuillez répondre aussi vite que possible.' Encercler le choix du participant. Items corrects = 2 points, erreur = 1 point, erreur égocentrique = 0 points.

Score
0-12

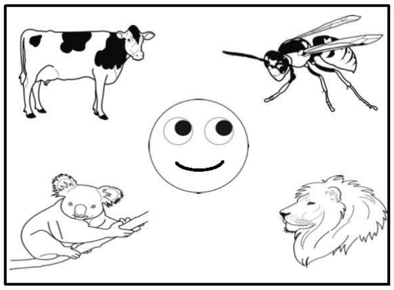

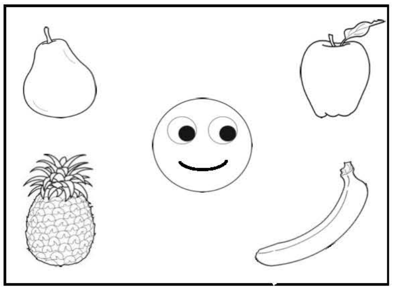

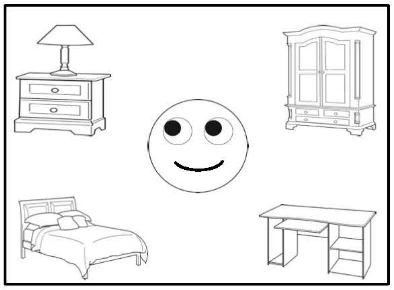

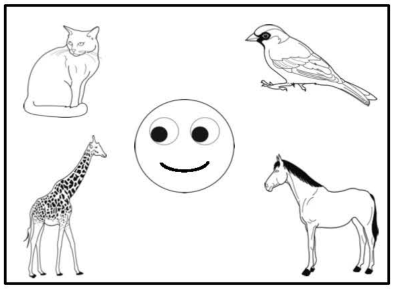

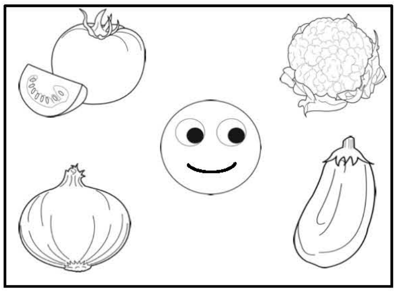

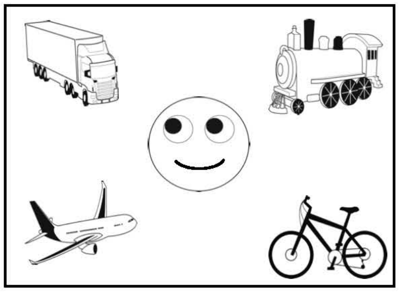

MÉMOIRE – Rappel retardé

Procédure de notation pour la rétention: obtenir un score de rappel différé (page suivante) et, avec le score de rappel immédiat, déterminer le pourcentage retenu. Convertir le pourcentage retenu en score de rétention. Si score = 0, score converti = 0.

Calcul du pourcentage de rappel retardé retenu

(Score de rappel retardé) / (Score de rappel immédiat) *x 100 = % retenu*

(..................................) / (..................................) *x 100 = ...% retenu*

Tableau du pourcentage retenu et du score de rétention

Pourcentage retenu	**Score converti**	Pourcentage retenu	**Score converti**
1-10%	**1**	51-60%	**6**
11-20%	**2**	61-70%	**7**
21-30%	**3**	71-80%	**8**
31-40%	**4**	81-90%	**9**
41-50%	**5**	91-100+ %	**10**

➲ Dites: 'Au début de cette entrevue, je vous ai lu une histoire courte. Dites-moi tout ce que vous pouvez vous rappeler sur cette histoire'. Accordez 1 point pour chaque section soulignée (entière ou partielle) rappelée.<br><br>***Trois*** ***bateaux de pêche*** *ont* ***aidé à sauver*** *une* ***baleine*** *qui nageait trop près du* ***rivage****. La baleine a été aperçue en train de nager en* ***cercles****.* ***Alain Williams****, de la* ***Société de conservation marine*** *a affirmé que* ***trente-deux*** *jeunes baleines se sont perdues* ***l'hiver passé*** *en cherchant de la nourriture.*	Score brut de rappel retardé (0-10) ☐<br><br>**Score retenu converti (0-10)** ☐

MÉMOIRE – Reconnaissance retardée

Si tous les éléments rappelés, passez et notez 4. Sinon, posez des questions ci-dessous.

Dites: 'Voyons voir si vous pouvez vous rappeler de plus de détails sur cette histoire. Je vais vous poser quelques questions, répondez-moi par oui ou par non.'

Encerclez les réponses (vrai ou faux) et notez 1 point pour chaque item reconnu dans cette section. Utilisez le tableau ci-dessous pour calculer le score.

Score 0-4 ☐

Y avait-il quatre bateaux de pêche?	V	**F**	1
L'histoire était-elle à propos du sauvetage de dauphins?	V	**F**	1
Est-ce que cela a eu lieu proche du rivage?	**V**	F	1
L'animal nageait-il en cercle?	**V**	F	1
L'homme dans l'histoire s'appelle-t-il Mr. Williams?	**V**	F	1
Son prénom était-il Stephen?	V	**F**	1
Est-ce que 32 baleines se sont perdues l'été passé?	V	**F**	1
Les baleines perdues cherchaient-elles de la nourriture?	**V**	F	1

Oui = Vrai (V)
Non = Faux (F)

Tableau du score de reconnaissance de la reconnaissance	
No. De bonne réponses	**Score Converti**
0-4	**0**
5	**1**
6	**2**
7	**3**
8	**4**

SCORES

**Langage**	Appellation, Compréhension, Orthographe	/28
**Fluence verbale**	Aisance de langage Lettre F, Aisance de langage Lettre D	/24
**Executif**	Empan de chiffres inversé, Alternance, Complétion de phrase, Cognition sociale	/48
	**SLA-SPECIFIQUE:**	**/100**
Mémoire	*Rappel immédiat, Rappel retardé, Reconnaissance retardée*	*/24*
Visuospatial	*Compter les points, Comptage de cubes, Localisation de chiffres*	*/12*
	SLA NON-SPECIFIQUE:	***/36***
	**SCORE ECAS TOTAL:**	**/136**

S. Abrahams & T.H. Bak
Dot counting is reproduced with kind permission from J. R. Hodges

ECHELLE COGNITIVE ET COMPORTEMENTALE D'EDIMBOURG DE DÉPISTAGE DE LA SLA
ECAS (Version Français Canada 2018)

Dépistage de comportement – Entretien avec aidant

COMPORTEMENT

➲ Veuillez interroger l'aidant au sujet des comportements possibles suivants. Les symptômes devraient avoir eu lieu de façon répétitive et non une fois seulement, et peuvent avoir eu lieu avant le développement d'un symptôme moteur quelconque. Cochez oui, non, ou ne sait pas. Si oui, décrivez brièvement. Donnez 1 point pour chaque réponse oui (maximum = 10).

**A**	**Désinhibition comportementale**			
1	Comportement socialement inapproprié, e.g.<br>*comportement inapproprié avec des étrangers*<br>*comportement criminel*	O	N	NSP
2	Perte de manières ou de décorum, e.g.<br>*remarques crues ou sexuellement explicites, blagues ou opinions pouvant être offensantes pour d'autres*<br>*manque de réponse face aux signaux sociaux*	O	N	NSP
3	Actions impulsives, imprudentes ou négligentes, e.g.<br>*Commence à faire des jeux de hasard, achète ou vend des propriétés sans considérer les conséquences, partage des informations personnelles de façon inappropriée (numéro de carte de crédit, etc.)*	O	N	NSP
**B**	**Apathie, Inertie**			
4	Perte d'intérêt ou de motivation , e.g.<br>*passivité, manque de spontanéité*<br>*besoin d'être poussé pour initier ou continuer des activités routinières*	O	N	NSP
**C**	**Perte de sympathie ou d'empathie**			
5	Diminution de la réponse aux besoins et sentiments des autres<br>*Un score positif à cette section devrait être basé sur des exemples spécifiques montrant un manque de compréhension ou une indifférence aux sentiments des autres.*<br>*Commentaires blessants*<br>*Ne tiens pas compte de la douleur ou la détresse des autres*	O	N	NSP
6	Diminution de l'intérêt social, de l'interrelation, de la chaleur ou de la proximité dans les interactions sociales, e.g.<br>*froideur*<br>*pas de contact visuel*	O	N	NSP
**D**	**Comportement de persévération, stéréotype, compulsif ou de rituel**			
7	Mouvements simples et répétés, e.g.<br>*tapotements, applaudissements*<br>*grattage, arrachage de peau ou de vêtements*<br>*répétition de mots*	O	N	NSP
8	Comportements complexes, compulsifs ou de rituel, e.g.<br>*Comptage, rituel de nettoyage, vérification*<br>*Collectionner, accumuler des objets*	O	N	NSP
**E**	**Frénésies alimentaires ou hyperoralité et changement de préférences alimentaires**			

9	Changement de préférences alimentaires, e.g.<br>*manies alimentaires (habitudes changeantes)*<br>*envies de glucides (particulièrement ceux sucrés)*	O	N	NSP	
10	Grignotage compulsif ou hyperoralité, e.g.,<br>*Boulimie ou continuer à manger même après satiété*<br>*exploration orale ou consommation d'objets non-comestibles*	O	N	NSP	
**SCORE**					
				**TOTAL**	*/10*

**SYMPTÔMES**	
➲ Veuillez cochez la case si au moins un des symptômes était présent dans chacune des catégories suivantes.	
**A. Désinhibition comportementale**	
**B. Apathie, Inertie**	
**C. Perte de sympathie ou d'empathie**	
**D. Comportement de persévération, stéréotype, compulsif ou de rituel**	
**E. Hyperoralité et changement de préférences alimentaires**	

**Dépistages de psychose SLA**				
➲ Veuillez interroger l'aidant au sujet des symptômes possibles suivants. Cochez oui, non, ou ne sait pas. Si oui, décrivez brièvement. Donnez 1 point pour chaque réponse oui (maximum de 3).				
1	A des comportements ou croyances bizarres	O	N	NSP
2	Entend ou voit des choses qui ne sont pas là, et/ou ressent la présence de quelqu'un qui n'est pas là	O	N	NSP
3	Est excessivement suspicieux, et/ou se sent persécuté	O	N	NSP
**SCORE**				
			**TOTAL**	*/3*

**DÉBUT ET DURÉE DES SYMPTÔMES**		
➲ Veuillez cocher ou compléter la case pour indiquer la réponse.		
**1. Est-ce que ces symptômes représentent un changement par rapport au comportement passé du patient?**	O	NSP
Si oui, les changements ont-ils eu lieu:		
a. AVANT le début de la maladie?	O	NSP
b. Au même moment?	O	NSP
c. APRÈS le début de la maladie?	O	NSP
**2. Ces symptômes persistent-ils?**	O	NSP
**3. Si non, combien de temps ont-ils duré?**		

Appendix 9.2.5: ECAS C [ENGLISH]

Found in [ECAS C (EN, V1.1, 2020) - CAPTURE ALS]

EDINBURGH COGNITIVE AND BEHAVIORAL ALS SCREEN – UNIVERSITY OF PENNSYLVANIA (ECAS-PENN)

North American English Version Form C, Version 1.1 Revised (2020)

Developed by Sharon Abrahams and Thomas H. Bak, University of Edinburgh

Adapted for use in North America by Katya Rascovsky, Corey McMillan, and Murray Grossman, University of Pennsylvania, in collaboration with Michael Benatar on behalf of the Clinical Research in ALS and Related Disorders for Therapeutic Development (CReATe) Consortium.

Date of testing: ....

Occupation: ....

....

Handedness: ....

Years of education: ....

Name/ID: ....

Date of Birth: ....

Highest Completed Degree: ....

Premorbid language difficulties ....

LANGUAGE - Naming

➲ Ask: Say or write down the names of these pictures:

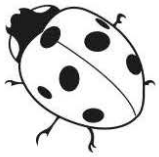
....

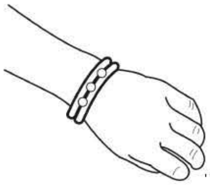
....

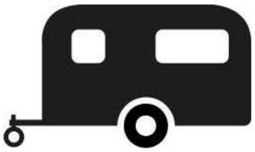
....

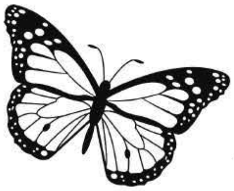
....

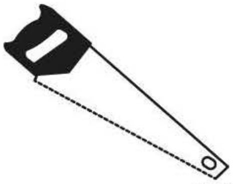
....

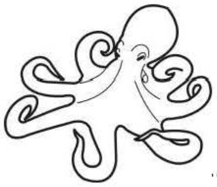
....

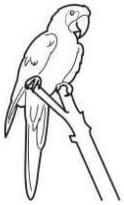
....

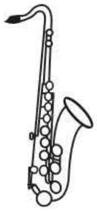
....

Score 0-8

LANGUAGE - Comprehension

➲ Ask: Point or say the one which is:

1. Something that was once a caterpillar ....
2. Something a carpenter uses...................................
3. Something with feathers...................................
4. A place to sleep...................................
5. Something you wear...................................
6. Something you play...................................
7. Something used to cut wood...................................
8. An animal that lives in water...................................

Score 0-8

MEMORY – Immediate recall

➲ Say: 'I am going to read you a short story. Please listen carefully. When I am finished, say or write as much as you can remember'. Score according to the Administration and Guidance Notes.

Helen Blake, from Boston has been awarded the Northern Art Prize for photography. The forty-seven year old started taking photos while hiking. Helen beat seven hundred competitors with her picture of an oak tree in autumn colors.

Score 0-10 ☐

Also use this score to calculate % retained (bottom p.7)

LANGUAGE - Spelling

➲ Say: 'Spell, either by speaking or writing, the following words.' If the person is using assistive technology, ask them to turn off any predictive text facility. Give one point on "headlamp", "wheelbarrow", "babysitter", and "lifeguard" if the person inserts a "space" when spelling the word.

1. Portrait	....	2. Babysitter	....
3. Entertaining	....	4. Reduction	....
5. Aquarium	....	6. Lifeguard	....
7. Borrow	....	8. Suggested	....
9. Headlamp	....	10. Schedule	....
11. Wheelbarrow	....	12. Wrong	....

Score 0-12 ☐

FLUENCY - Letter P ☐ Oral ☐ Written

➲ Say: 'I am going to give you a letter of the alphabet and I would like you to say or write as many different words as you can beginning with that letter, but not names of people or places, or numbers.'

- If speaking, say 'You will have **one** minute. The letter is P.'
- If writing, say: 'You will have **two** minutes. The letter is P.'

No. of correct words =

Time to read/ copy aloud =

Vfi =

➲ Next the person copies/reads these words aloud.

- If speaking, say: 'read aloud these words as fast as possible. Before you do this, check that you can read them. I will time you. Ready? Begin.'
- If writing, say: 'copy these words as fast as possible. I will time you. Ready? Begin.'

Verbal Fluency Index (Vfi) calculation:

If spoken:

$$Vfi = \frac{60seconds - \text{no. of seconds to read aloud words}}{\text{No. of correct words generated}}$$

If written:

$$Vfi = \frac{120seconds - \text{no. of seconds to copy words}}{\text{No. of correct words generated}}$$

VFI conversion to score table

SPOKEN VFI	WRITTEN VFI	**Score**
≥ 12.00	≥ 20.00	**0**
10.00 to < 12.00	16.50 to < 20.00	**2**
8.00 to < 10.00	13.00 to < 16.50	**4**
6.00 to < 8.00	9.50 to < 13.00	**6**
4.00 to < 6.00	6.00 to < 9.50	**8**
2.00 to < 4.00	2.50 to < 6.00	**10**
< 2.00	< 2.50	**12**

Score 0-12 ☐

EXECUTIVE – Reverse Digit Span

➲ Say: 'I am going to say some numbers and I would like you to say them back to me in reverse order. For example, if I say '2 3 4', you should say '4 3 2'. Let's practice. If I say '7 1 9', what would you say?' Stop when person gets both trials of a line wrong. Score total number of trials correct. After each pair of trials remind the participant 'Now the number of items will increase'.

Score 0-12 ☐

Trial		Check	Trial		Check
1	3 1		2	8 4	
3	7 2 5		4	9 6 4	
5	1 8 4 6		6	7 3 6 8	
7	4 9 6 1 3		8	1 8 9 7 5	
9	5 6 1 4 9 2		10	2 7 3 4 8 1	
11	6 2 7 9 1 4 3		12	7 6 8 9 2 3 5	

EXECUTIVE – Alternation

➲ Say: 'I want you to alternate between numbers and letters, starting with 1-A, then 2-B, 3-C, and so on. Please continue from there, alternating between numbers and letters, in order, without skipping any until I tell you to stop. Let's begin together: 1-A, 2-B, 3-C…'

Score 0-12 ☐

Trial		Check	Trial		Check	Trial		Check	Trial		Check
1	4-D		2	5-E		3	6-F		4	7-G	
5	8-H		6	9-I		7	10-J		8	11-K	
9	12-L		10	13-M		11	14-N		12	15-O	

FLUENCY - Letter M ☐ Oral ☐ Written

➲ Say: 'I am going to give you a letter of the alphabet and I would like you to say or write as many different words as you can beginning with that letter, but not names of people or places, or numbers. This time the word must only be **four letters** long. No more or less than four letters'

- If speaking, say: 'You will have **one** minute. The letter is M.'
- If writing, say 'You will have **two** minutes. The letter is M.'

No. of correct words =

Time to read/ copy aloud =

Vfi =

➲ Next the person copies/reads these words aloud.

- If speaking, say: 'read aloud these words as fast as possible. Before you do this, check that you can read them. I will time you. Ready? Begin.'
- If writing, say: 'copy these words as fast as possible. I will time you. Ready? Begin.'

Verbal Fluency Index (Vfi) calculation:

If spoken:

$$Vfi = \frac{60\text{seconds} - \text{no. of seconds to read aloud words}}{\text{No. of correct words generated}}$$

If written:

$$Vfi = \frac{120\text{seconds} - \text{no. of seconds to copy words}}{\text{No. of correct words generated}}$$

VFI conversion to score table

SPOKEN VFI	WRITTEN VFI	**Score**
≥ 20.00	≥ 27.25	**0**
16.75 to < 20.00	23.00 to < 27.25	**2**
13.50 to < 16.75	18.75 to < 23.00	**4**
10.25 to < 13.50	14.50 to < 18.75	**6**
7.00 to < 10.25	10.25 to < 14.50	**8**
3.75 to < 7.00	6.00 to < 10.25	**10**
< 3.75	< 6.00	**12**

Score 0-12 ☐

VISUOSPATIAL – Dot Counting

➲ Say: 'I would like you to count how many dots are in each box, but without pointing to them.'

Score 0-4

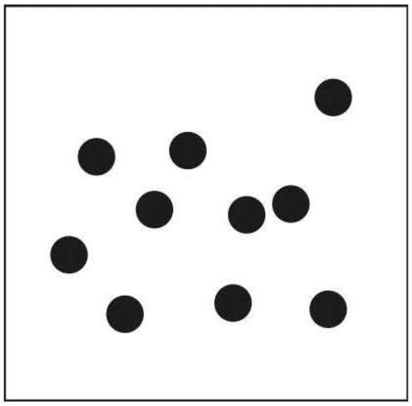

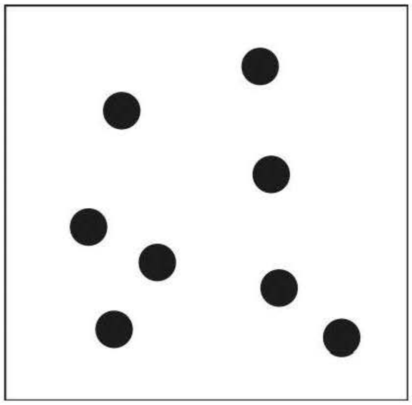

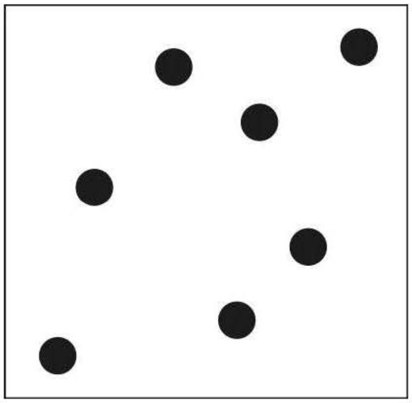

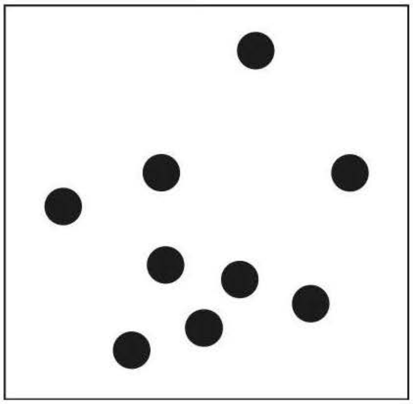

VISUOSPATIAL – Cube Counting

➲ Say: 'How many cubes are in each structure, including the ones you may not be able to see?'

Score 0-4

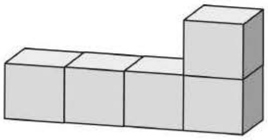

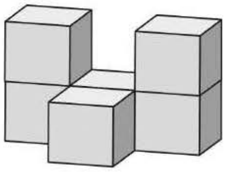

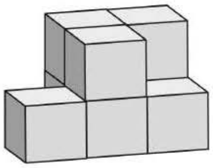

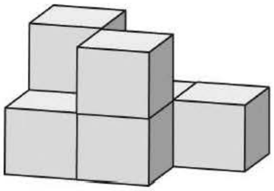

VISUOSPATIAL – Number Location

➲ Say: 'Which number corresponds to the position of the dot?'

Score 0-4

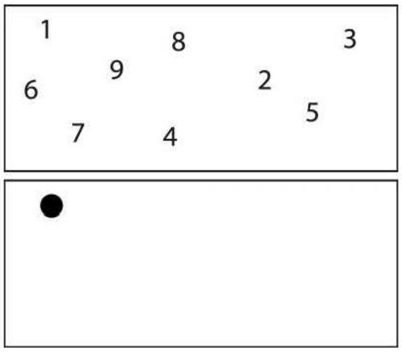

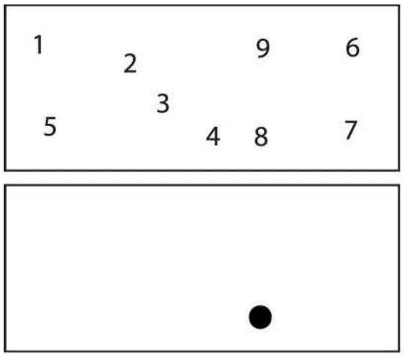

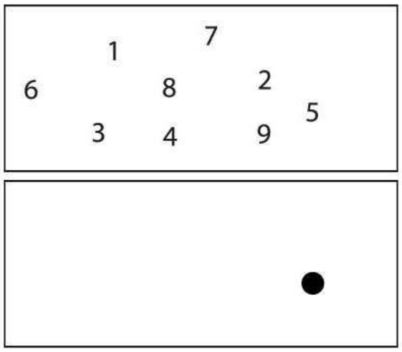

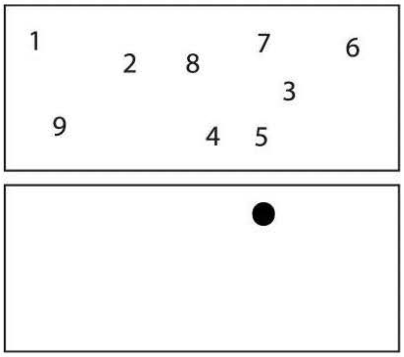

EXECUTIVE – Sentence Completion

➲ Say: 'Listen carefully to these sentences and as soon as I have finished reading them, please tell me, or write, a word that finishes the sentence as quickly as possible. For example, '*She was so tired that she went straight to…bed*'. Do not score.

1. He called up the restaurant to reserve a ....
2. When she got up in the morning, the sun was...................

➲ Say: 'Now I'd like you to do that again, but this time the word you give should not make sense whatsoever in the context of the sentence. It must not be related to the word that actually completes the sentence. For example, '*John cut his hand with the sharp…orange*'. If the person does not respond within 20 seconds, move onto the next question.

1. Lisa went to the library to return some …………………………	Score 0-12
2. After her shower, she dried herself with a …………………………	
3. He put a teabag in his mug and boiled the …………………………	
4. He studied medicine to become a …………………………	
5. The music started and everyone got up to …………………………	
6. John picked up the leash and took his dog for a …………………………	
**Give 2 points for different word, 1 point for related word (e.g. associated or opposite meaning) or 0 points for exact word.**	

SOCIAL COGNITION – Part A

➲ Say: 'You are going to see some pictures, one in each corner of a box. You have to choose **which picture you like best.** Either point to or say which picture you like best. Please respond as quickly as possible.' Circle participant's choice.

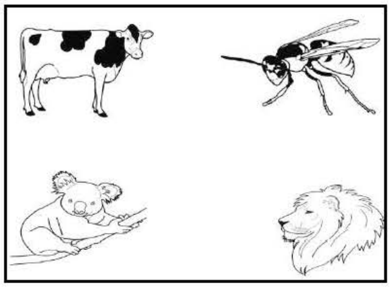

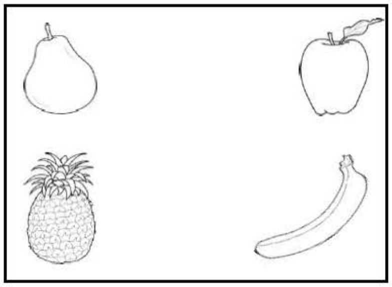

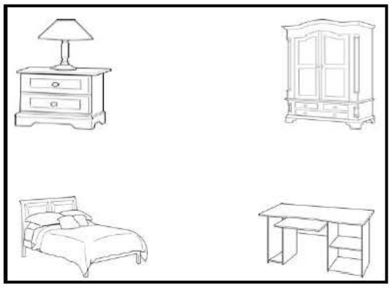

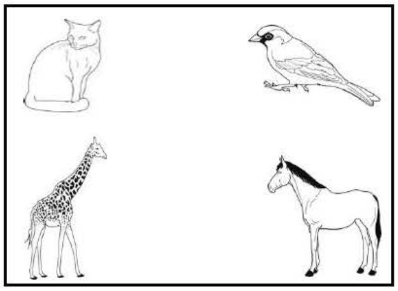

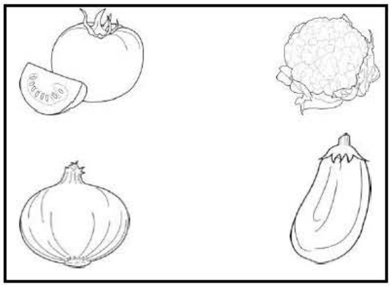

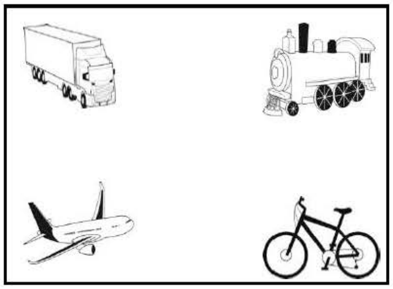

SOCIAL COGNITION – Part B

➲ Say: 'You are going to see some pictures, one in each corner of a box. You have to choose **which picture the face likes best.** Either point to or say which picture **the face likes best**. Please respond as quickly as possible.' Circle participant's choice. Correct items = 2 points, error = 1 point, egocentric error = 0 points.

Score 0-12

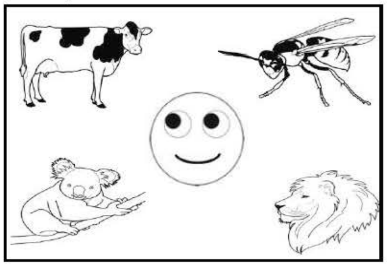

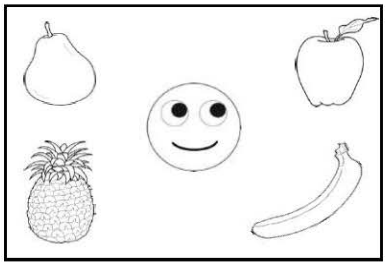

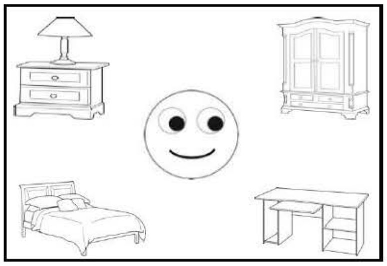

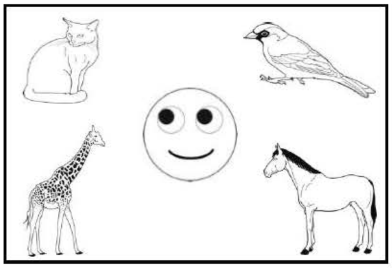

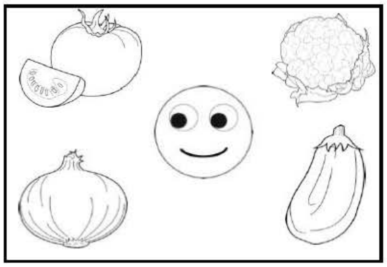

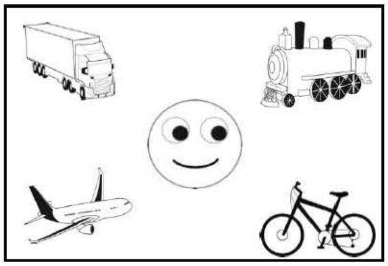

MEMORY – Delayed Recall

Scoring procedure for retention: obtain delayed recall performance (page 8) and, together with immediate recall score (page 2), determine percentage retained. Convert percentage retained to Score using table below. If delayed recall = 0, score = 0.

Delayed recall to percentage retained calculation

(Delayed recall score [page 8]) / (Immediate recall score [page 2[) *x 100 = % retained*

(.................................) / (.................................) *x 100 = ...% retained*

Percentage retained to converted score table

Percentage retained	Score	Percentage retained	Score
1-10%	1	51-60%	**6**
11-20%	**2**	61-70%	**7**
21-30%	**3**	71-80%	**8**
31-40%	**4**	81-90%	**9**
41-50%	**5**	91-100+ %	**10**

➲ Say: 'At the beginning of this interview, I read you a short story. Tell me as much as you can remember from that story'. Score according to the Administration and Guidance Notes.

Helen Blake, from Boston has been awarded the Northern Art Prize for photography. The forty-seven year old started taking photos while hiking. Helen beat seven hundred competitors with her picture of an oak tree in autumn colors.

Delayed recall raw score (0-10) ☐

Converted retention score (0-10) ☐

MEMORY – Delayed Recognition

If all items recalled, skip and score 4. Otherwise ask questions below.

Say: 'Let's see if you can remember anything more about that story. I will ask you some questions, please tell me if they are true or false'.

Circle responses (true or false) and score 1 point for each item recognized in this section. Use table below to calculate score.

Score 0-4 ☐

Was the woman in the story called Helen?	**T**	F	1
Was her second name Smith?	T	**F**	1
Was she from Boston?	**T**	F	1
Did the woman in the story win the Northern Art Prize?	**T**	F	1
Was her prize for painting?	T	**F**	1
Were there nine hundred competitors?	T	**F**	1
Was the woman's picture of an oak tree?	**T**	F	1
Was the woman's picture in winter colors?	T	**F**	1

Yes = True (T)
No = False (F)

Recognition to recognition score table

Number of correct answers	**Score**
0-4	**0**
5	**1**
6	**2**
7	**3**
8	**4**

SCORES

**Language**	Naming, Comprehension, Spelling	/28
**Verbal Fluency**	Fluency Letter P, Fluency Letter M	/24
**Executive**	Reverse Digit Span, Alternation, Sentence Completion, Social Cognition	/48
	**ALS-SPECIFIC:**	**/100**
Memory	*Immediate recall, Delayed recall score, Delayed recognition*	*/24*
Visuospatial	*Dot Counting, Cube Counting, Number Location*	*/12*
	ALS NON-SPECIFIC:	***/36***
	**ECAS TOTAL SCORE:**	**/136**

S. Abrahams & T. H. Bak
Dot counting is reproduced with kind permission from J. R. Hodges

EDINBURGH COGNITIVE AND BEHAVIORAL ALS SCREEN – UNIVERSITY OF PENNSYLVANIA (ECAS-PENN)
American English Form A, Version 1 (2018)

BEHAVIOR SCREEN – Caregiver Interview

➲ Please ask the caregiver about the following possible behaviors. Symptoms should have occurred repeatedly and not just on one instance, and may have occurred prior to the development of any motor signs. Tick 'Yes', 'No' or 'Don't Know'. If 'Yes', please provide a brief written description. Give one point for every 'Yes' response (maximum = 10).

BEHAVIOR

**A**	**Behavioral disinhibition**			
1	Socially inappropriate behavior, e.g.<br>*inappropriate behavior with strangers*<br>*criminal behavior*	Y	N	DK
2	Loss of manners or decorum, e.g.<br>*crude or sexually explicit remarks, jokes or opinions that may be offensive to others*<br>*lack of response to social cues*	Y	N	DK
3	Impulsive, rash or careless actions, e.g.<br>*new onset gambling, or buying or selling property without regard for consequences*<br>*giving out personal information inappropriately, e.g. credit card numbers*	Y	N	DK
**B**	**Apathy or inertia**			
4	Loss of interest, drive or motivation, e.g.<br>*passivity and lack of spontaneity*<br>*needs prompting to initiate or continue routine activities*	Y	N	DK
**C**	**Loss of sympathy or empathy**			
5	Diminished response to other people's needs and feelings<br>*Positive rating on this feature should be based on specific examples that reflect a lack of understanding or indifference to other people's feelings, e.g*<br>*hurtful comments*<br>*disregard for others' pain or distress*	Y	N	DK
6	Diminished social interest, interrelatedness, personal warmth or general closeness in social engagement, e.g.<br>*coldness*<br>*lack of eye contact*	Y	N	DK
**D**	**Perseverative, stereotyped, compulsive or ritualistic behavior**			
7	Simple repetitive movements, e.g.<br>*tapping, clapping*<br>*scratching, picking skin or clothing*<br>*repeating words*	Y	N	DK
8	Complex, compulsive or ritualistic behaviors, e.g.<br>*counting, cleaning rituals, checking*<br>*collecting, hoarding*	Y	N	DK

E	Hyperorality and altered food preferences			
9	Altered food preferences, e.g.<br>*food fads*<br>*carbohydrate craving (particularly sweets)*	Y	N	DK
10	Binge eating or hyperorality, e.g.,<br>*cramming or continuing to eat despite satiety*<br>*oral exploration or consumption of inedible objects*	Y	N	DK
**SCORE**				
	**TOTAL**			*/10*

**SYMPTOMS**	
➲ Please check box if at least one of the symptoms was present in each of the following categories.	
**A. Behavioral disinhibition**	
**B. Apathy or inertia**	
**C. Loss of sympathy or empathy**	
**D. Perseverative, stereotyped, compulsive or ritualistic behaviour**	
**E. Hyperorality and altered food preferences**	

ALS Psychosis Screen

➲ Please ask the caregiver about the following possible symptoms. Check 'Yes', 'No' or 'Don't Know'. If 'Yes', please provide a brief written description. Give one point for every 'Yes' response (maximum = 3).

1	Has strange and/or bizarre beliefs and behaviors	Y	N	DK
2	Hears or sees things that are not there, and/or feels the presence of someone who is not there	Y	N	DK
3	Is overly suspicious, and/or feels persecuted	Y	N	DK
**SCORE**				
	**TOTAL**			*/3*

**ONSET AND DURATION OF SYMPTOMS**		
➲ Please check or complete box to indicate response.		
**1. Do these symptoms represent a CHANGE from the patient's previous behavior?**	Y	N
If yes, did the changes occur:		
a. BEFORE the onset of the disease?	Y	N
b. at the same time as other symptoms?	Y	N
c. AFTER the onset of the disease?	Y	N
**2. Do they still persist?**	Y	N
**3. If not, how long did they last?**		

Appendix 9.2.6: ECAS C [FRENCH]

Found in [ECAS C (FR, 15May2020) - CAPTURE ALS]

ECHELLE COGNITIVE ET COMPORTEMENTALE D'EDIMBOURG DE DÉPISTAGE DE LA SLA

ECAS Formulaire C (Version Français Canada 2020)

Developed by S. Abrahams and T.H. Bak, University of Edinburgh

Adapted for use in North America by K. Rascovsky and M. Grossman, University of Pennsylvania

Date d'essai: ....

Occupation: ....

....

Main Dominante:...

Années d'éducation:... … … … … … … … … … … … … … ....

Nom/ID: ....

Date de naissance: ....

Éducation: Secondaire = 11; CEGEP = 13

Baccalauréat = 16; Maitrise = 18; Doctorat = 20

Difficulté de langage?..

LANGAGE - Appellation

➲ Demandez: Veuillez dire ou écrire le nom des images suivantes:

Score 0-8 ☐

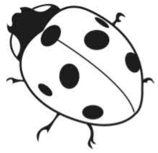

.... ☐

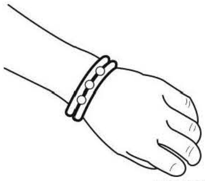

.... ☐

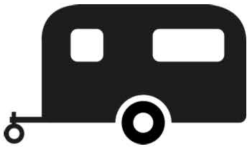

.... ☐

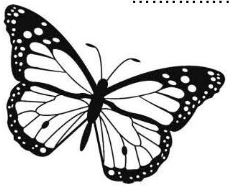

.... ☐

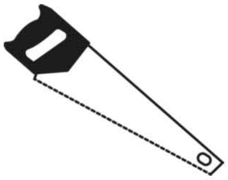

.... ☐

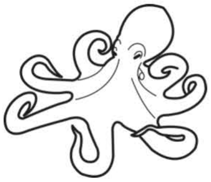

.... ☐

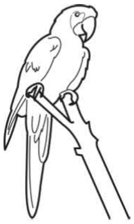

.... ☐

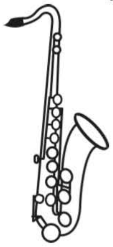

.... ☐

LANGAGE - Compréhension

➲ Demandez: pointez:

Score 0-8 ☐

1. Quelque chose qui était autrefois une chenille
2. Quelque chose qu'un charpentier utilise ……… .
3. Quelque chose avec des plumes ………………
4. Un endroit où dormir ……………………………
5. Quelque chose que vous portez ………………
6. Quelque chose que vous jouez ……………..........
7. Quelque chose que l'on utilise pour couper du bois
8. Un animal qui vit dans l'eau ………………………

MÉMOIRE – Rappel immédiat

Score 0-10 ☐

Utiliser également ce score pour calculer le % de rétention plus tard

➲ Dites: 'Je vais vous lire une histoire courte. Veuillez l'écouter attentivement. Lorsque j'ai fini, dites ou écrivez autant que vous le pouvez sur l'histoire.' Accordez 1 point pour chaque section soulignée (entière ou partielle) rappelée.

*<u>**Hélène Blake**</u>, de <u>**Boston**</u>, a reçu le <u>**Prix**</u> d'art du <u>**Nord**</u> en <u>**photographie**</u>. La dame de <u>**47**</u> ans a commencé à prendre des photographies en faisant de la <u>**randonnée**</u>. Hélène a battu <u>**700**</u> compétiteurs avec sa photo d'un <u>**chêne**</u> aux <u>**couleurs d'automne.**</u>*

LANGAGE – Orthographe

➲ Dites: 'Veuillez épeler à haute voix sur papier les mots suivants.' Si la personne utilise une technologie d'assistance, demandez-lui d'éteindre toute fonction prédictive.

Score 0-12 ☐

1. Portrait ....
2. Baby-sitter……………………………………………
3. Divertissant ....
4. Réduction …………………………...............................
5. Aquarium ………………………………………….
6. Sauveteur...
7. Emprunter …...
8. Suggéré ……………………………………………......
9. Lampe frontale ………………………………...
10. Horaire …..
11. Brouette ……………………………………….
12. Faux ………………………………………………....

FLUENCE VERBALE – Lettre P

☐ Oral ☐ Écrit

➲ Dites: 'Je vais vous donner une lettre de l'alphabet et j'aimerais que vous disiez ou écriviez autant de mots que vous le pouvez qui commencent par cette lettre. Les noms propres ou les chiffres ne sont pas acceptés.'

- Si parlé, dites: 'Vous avez une minute. La lettre est P.'
- Si écrit, dites: 'Vous avez deux minutes. La lettre est P.'

No. de mots corrects =

Temps pour lire/ copier =

Vfi =

➲ Ensuite, la personne copie ou lit les mots à haute voix.

- Si parlé, dites: 'Lisez ces mots à haute voix aussi vite que possible. Avant de faire cela, vérifiez que vous pouvez les lire. Je vais vous chronométrer. Prêt? Commencez.'
- Si écrit, dites: 'Copiez ces mots aussi vite que possible. Je vais vous chronométrer. Prêt? Commencez.'

Calcul "Verbal Fluency Index (Vfi)":

Si oral:

$$\text{Vfi} = \frac{\text{60 secondes – no. de secondes pour lire les mots à voix haute}}{\text{No. de mots corrects générés}}$$

Si écrit:

$$\text{Vfi} = \frac{\text{120secondes – no. de secondes pour copier les mots}}{\text{No. de mots corrects générés}}$$

Conversion VFI en tableau de score

ORAL VFI	ECRIT VFI	Score
≥ 12.00	≥ 20.00	0
10.00 to <12.00	16.50 to < 20.00	2
8.00 to < 10.00	13.00 to < 16.50	4
6.00 to < 8.00		
4.00 to < 6.00	9.50 to < 13.00	6
2.00 to < 4.00	6.00 to < 9.50	8
< 2.00	2.50 to < 6.00	10

Score 0-12 ☐

EXÉCUTIF – Empan de chiffres inversé

➲ Dites: 'Je vais vous donner une liste de chiffres et j'aimerais que vous me les répétiez dans l'ordre inversé. Par exemple, si je dis 2-3-4, vous devriez dire 4-3-2. Essayons avec 7-1-9, que me diriez-vous?' Arrêtez quand la personne se trompe aux deux essais de la même ligne. Notez le nombre total d'essais corrects.

Essai		Vérifier	Essai		Vérifier
1	3 1		2	8 4	
3	7 2 5		4	9 6 4	
5	1 8 4 6		6	7 3 6 8	
7	4 9 6 1 3		8	1 8 9 7 5	
9	5 6 1 4 9 2		10	2 7 3 4 8 1	
11	6 2 7 9 1 4 3		12	7 6 8 9 2 3 5	

Score 0-12

Longeur

EXÉCUTIF – Alternance

➲ Dites: 'J'aimerais que vous alterniez entre chiffres et lettres, en commençant par 1A, puis 2B, 3C, et ainsi de suite. Veuillez alterner entre chiffres et lettres, dans l'ordre, sans en sauter, jusqu'à ce que je vous dise d'arrêter. Commençons ensemble : 1A, 2B, 3C...'

Essai		Vérifier	Essai		Vérifier	Essai		Vérifier	Essai		Vérifier
1	4-D		2	5-E		3	6-F		4	7-G	
5	8-H		6	9-I		7	10-J		8	11-K	
9	12-L		10	13-M		11	14-N		12	15-O	

Score 0-12

FLUENCE VERBALE – Lettre M ☐ Oral ☐ Écrit

➲ Dites: 'Je vais vous donner une lettre de l'alphabet et j'aimerais que vous disiez ou écriviez autant de mots que vous le pouvez qui commencent par cette lettre. Les noms propres ou les chiffres ne sont pas acceptés. Maintenant, le mot doit être long de quatre lettres. Ni plus, ni moins, que quatre lettres.
- Si oral, dites: 'Vous avez une minute. La lettre est M.'
- Si écrit, dites: 'Vous avez deux minutes. La lettre est M.'

No. de mots corrects =

Temps pour lire/ copier =

Vfi =

➲ Ensuite, la personne copie ou lit les mots à haute voix.

- Si parlé, dites: 'Lisez ces mots à haute voix aussi vite que possible. Avant de faire cela, vérifiez que vous pouvez les lire. Je vais vous chronométrer. Prêt? Commencez.'
- Si écrit, dites: 'Copiez ces mots aussi vite que possible. Je vais vous chronométrer. Prêt? Commencez.'

Calcul "Verbal Fluency Index (Vfi)":

Si oral:
Vfi = 60 secondes – no. de secondes pour lire les mots a voix haute / No. de mots corrects générés

Si écrit:
Vfi = 120secondes – no. de secondes pour copier les mots / No. de mots corrects générés

Conversion VFI en tableau de score

ORAL VFI	ÉCRIT VFI	Score
≥ 20.00	≥ 27.25	0
16.75 to < 20.00	23.00 to < 27.25	2
13.50 to < 16.75	18.75 to < 23.00	4
10.25 to < 13.50	14.50 to < 18.75	6
7.00 to < 10.25	10.25 to < 14.50	8
3.75 to < 7.00	6.00 to < 10.25	10
< 3.75	< 6.00	12

Score 0-12

VISUOSPATIAL – Compter les points

➲ Dites: ‘J’aimerais que vous comptiez le nombre de points dans chaque boîte, mais sans les pointer.’

Score
0-4

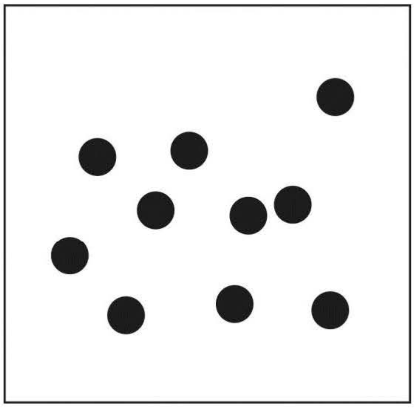

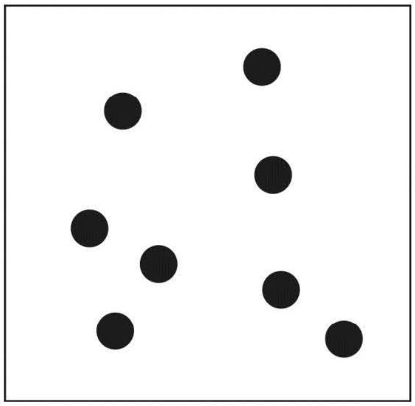

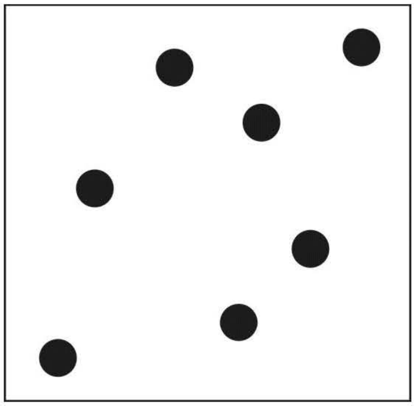

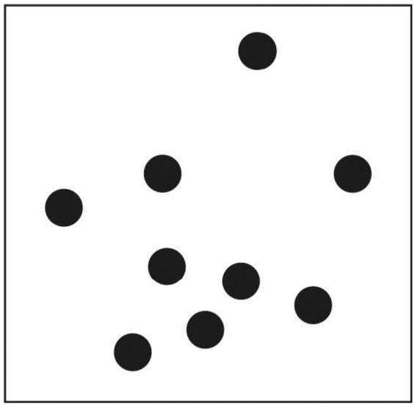

VISUOSPATIAL – Comptage de cubes

➲ Dites: ‘Combien de cubes y a-t-il dans chaque structure, incluant ceux que vous n’êtes pas capable de voir?’

Score
0-4

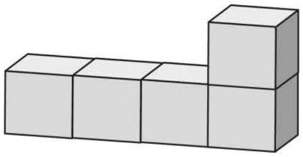

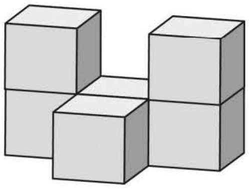

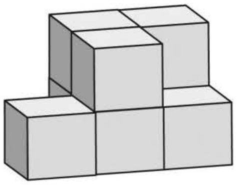

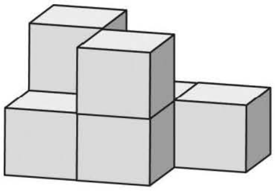

VISUOSPATIAL – Localisation de chiffres

➲ Dites: 'Quel chiffre correspond à la position du point?'

Score 0-4

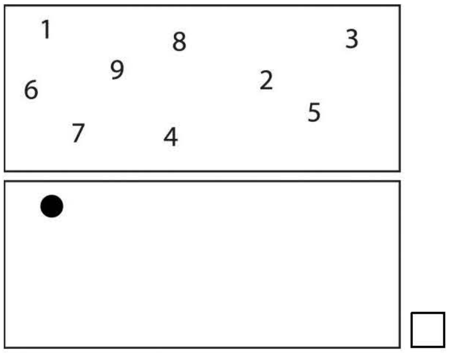

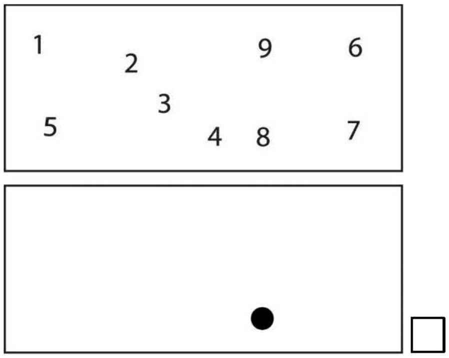

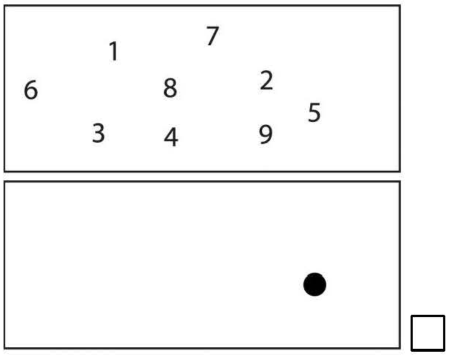

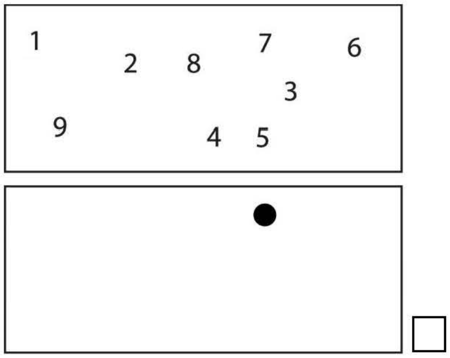

EXÉCUTIF – Complétion de phrase

➲ Dites: 'Écoutez attentivement ces phrases. Aussitôt que j'aurai fini de les lire, veuillez me dire, ou écrire, un mot qui finit la phrase aussi vite que possible. Par exemple: '*elle était si fatiguée qu'elle est allée directement au…lit*'. Ne pas donner de score.

1. Il a appelé le restaurant pour réserver une ....
2. Lorsqu'elle s'est réveillée le matin, le soleil était........................

➲ Dites: 'Maintenant j'aimerais que l'on recommence, mais cette fois-ci j'aimerais que le mot que vous donnerez ne fasse aucun sens dans le contexte de la phrase. Il ne doit pas être relié au mot qui complète correctement la phrase. Par exemple, `*John s'est coupé la main avec…une orange*'. Si la personne ne répond pas dans les 20 secondes, passez à la question suivante.

1. Lisa est allée à la bibliothèque pour rendre quelques ……………………..................................
2. Après sa douche, elle s’est séché avec une ………………………........................
3. Il a mis un sachet de thé dans sa tasse et il a fait bouillir de ……………….................................
4. Il a étudié en médecine pour devenir un ……………………………...…..…..
5. La musique a débuté et tout le monde s’est levé pour ……………………………………………..
6. John a ramassé la laisse et a pris son chien pour une ..………………………………..……..…

Donnez un score de 2 pour un mot différent, 1 pour un mot différent mais relié (associé ou sens opposé) ou 0 pour le mot exacte.

Score
0-12

COGNITION SOCIALE – Partie A

➲ Dites: ‘Vous allez voir quelques photos, une dans chaque coin d’une boîte. Vous devez choisir quelle photo vous préférez. Vous pouvez pointer ou dire quelle image vous préfèrez. Veuillez répondre aussi vite que possible.’ Encerclez le choix du participant.

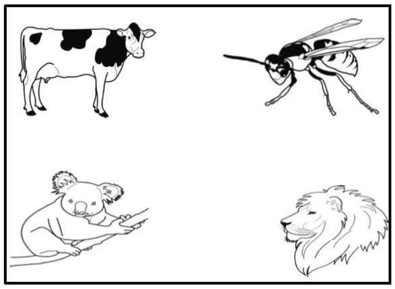

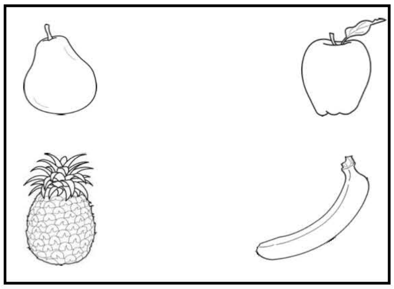

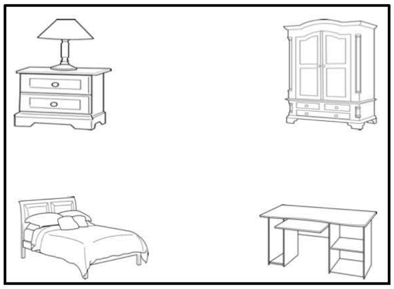

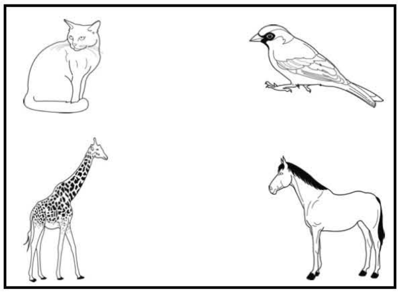

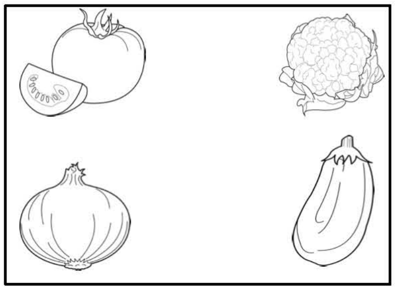

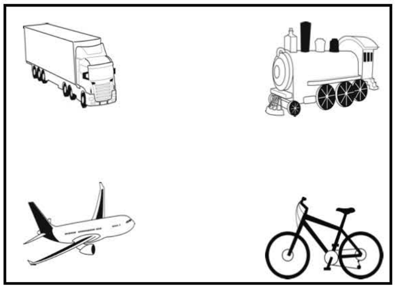

COGNITION SOCIALE – Partie B

➲ Dites: 'Vous allez voir quelques photos, une dans chaque coin d'une boîte. Vous devez choisir quelle photo le visage préfère. Vous pouvez pointer ou dire quelle image il préfère. Veuillez répondre aussi vite que possible.' Encercler le choix du participant. Items corrects = 2 points, erreur = 1 point, erreur égocentrique = 0 points.

Score 0-12

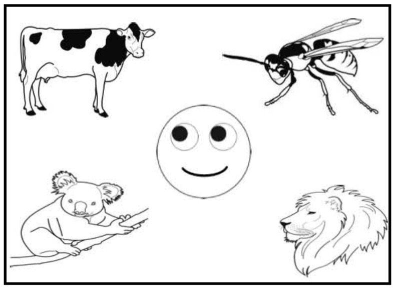

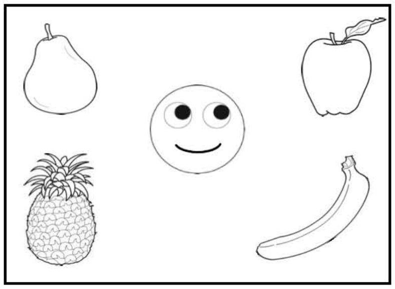

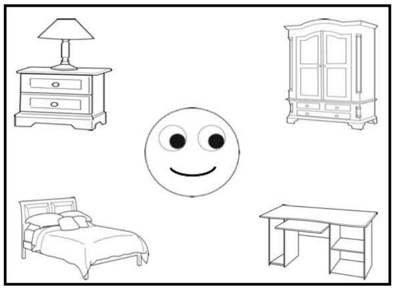

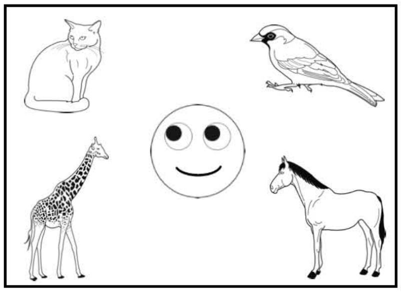

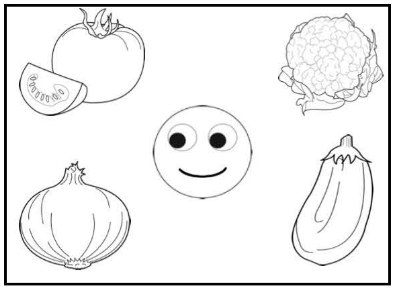

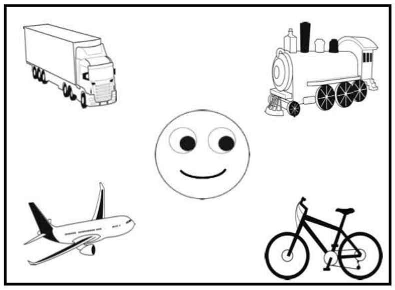

MÉMOIRE – Rappel retardé

Procédure de notation pour la rétention: obtenir un score de rappel différé (page suivante) et, avec le score de rappel immédiat, déterminer le pourcentage retenu. Convertir le pourcentage retenu en score de rétention. Si score = 0, score converti = 0.

Calcul du pourcentage de rappel retardé retenu

(Score de rappel retardé) / (Score de rappel immédiat) *x 100 = % retenu*

(..................................) / (..................................) *x 100 = ...% retenu*

Tableau du pourcentage retenu et du score de rétention

Pourcentage retenu	Score converti	Pourcentage retenu	Score converti
1-10%	**1**	51-60%	**6**
11-20%	**2**	61-70%	**7**
21-30%	**3**	71-80%	**8**
31-40%	**4**	81-90%	**9**
41-50%	**5**	91-100+ %	**10**

➲ Dites: 'Au début de cette entrevue, je vous ai lu une histoire courte. Dites-moi tout ce que vous pouvez vous rappeler sur cette histoire'. Accordez 1 point pour chaque section soulignée (entière ou partielle) rappelée.

Hélène Blake, *de* ***Boston***, *a reçu le* ***Prix*** *d'art du* ***Nord*** *en* ***photographie***. *La dame de* ***47*** *ans a commencé à prendre des photographies en faisant de la* ***randonnée***. *Hélène a battu* ***700*** *compétiteurs avec sa photo d'un* ***chêne*** *aux* ***couleurs d'automne.***

Score brut de rappel retardé (0-10) ☐

Score retenu converti (0-10) ☐

MÉMOIRE – Reconnaissance retardée

Si tous les éléments rappelés, passez et notez 4. Sinon, posez des questions ci-dessous.

Dites: 'Voyons voir si vous pouvez vous rappeler de plus de détails sur cette histoire. Je vais vous poser quelques questions, répondez-moi par oui ou par non.'

Encerclez les réponses (vrai ou faux) et notez 1 point pour chaque item reconnu dans cette section. Utilisez le tableau ci-dessous pour calculer le score.

Score 0-4 ☐

La femme de l'histoire s'appelait-elle Hélène?	**V**	F	1
Son nom de famille était-il Smith?	V	**F**	1
Était-elle de Boston?	**V**	F	1
La femme de l'histoire a-t-elle gagné le Prix d'art du Nord?	**V**	F	1
Son prix était-il pour la peinture?	V	**F**	1
Y avait-il 900 compétiteurs?	V	**F**	1
L'image de la femme était-elle un chêne?	**V**	F	1
L'image de la femme était-elle aux couleurs de l'hiver?	V	**F**	1

Oui = Vrai (V)
Non = Faux (F)

Tableau du score de reconnaissance de la reconnaissance	
No. De bonne réponses	**Score Converti**
0-4	**0**
5	**1**
6	**2**
7	**3**
8	**4**

SCORES

**Langage**	Appellation, Compréhension, Orthographe	/28
**Fluence verbale**	Aisance de langage Lettre P, Aisance de langage Lettre M	/24
**Executif**	Empan de chiffres inversé, Alternance, Complétion de phrase, Cognition sociale	/48
	**SLA-SPECIFIQUE:**	**/100**
Mémoire	*Rappel immédiat, Rappel retardé, Reconnaissance retardée*	*/24*
Visuospatial	*Compter les points, Comptage de cubes, Localisation de chiffres*	*/12*
	SLA NON-SPECIFIQUE:	***/36***
	**SCORE ECAS TOTAL:**	**/136**

S. Abrahams & T.H. Bak
Dot counting is reproduced with kind permission from J. R. Hodges

ECHELLE COGNITIVE ET COMPORTEMENTALE D'EDIMBOURG DE DÉPISTAGE DE LA SLA
ECAS (Version Français Canada 2018)

Dépistage de comportement – Entretien avec aidant

COMPORTEMENT

➲ Veuillez interroger l'aidant au sujet des comportements possibles suivants. Les symptômes devraient avoir eu lieu de façon répétitive et non une fois seulement, et peuvent avoir eu lieu avant le développement d'un symptôme moteur quelconque. Cochez oui, non, ou ne sait pas. Si oui, décrivez brièvement. Donnez 1 point pour chaque réponse oui (maximum = 10).

**A**	**Désinhibition comportementale**			
1	Comportement socialement inapproprié, e.g.<br>*comportement inapproprié avec des étrangers*<br>*comportement criminel*	O	N	NSP
2	Perte de manières ou de décorum, e.g.<br>*remarques crues ou sexuellement explicites, blagues ou opinions pouvant être offensantes pour d'autres*<br>*manque de réponse face aux signaux sociaux*	O	N	NSP
3	Actions impulsives, imprudentes ou négligentes, e.g.<br>*Commence à faire des jeux de hasard, achète ou vend des propriétés sans considérer les conséquences, partage des informations personnelles de façon inappropriée (numéro de carte de crédit, etc.)*	O	N	NSP
**B**	**Apathie, Inertie**			
4	Perte d'intérêt ou de motivation , e.g.<br>*passivité, manque de spontanéité*<br>*besoin d'être poussé pour initier ou continuer des activités routinières*	O	N	NSP
**C**	**Perte de sympathie ou d'empathie**			
5	Diminution de la réponse aux besoins et sentiments des autres<br>*Un score positif à cette section devrait être basé sur des exemples spécifiques montrant un manque de compréhension ou une indifférence aux sentiments des autres.*<br>*Commentaires blessants*<br>*Ne tiens pas compte de la douleur ou la détresse des autres*	O	N	NSP
6	Diminution de l'intérêt social, de l'interrelation, de la chaleur ou de la proximité dans les interactions sociales, e.g.<br>*froideur*<br>*pas de contact visuel*	O	N	NSP
**D**	**Comportement de persévération, stéréotype, compulsif ou de rituel**			
7	Mouvements simples et répétés, e.g.<br>*tapotements, applaudissements*<br>*grattage, arrachage de peau ou de vêtements*<br>*répétition de mots*	O	N	NSP
8	Comportements complexes, compulsifs ou de rituel, e.g.<br>*Comptage, rituel de nettoyage, vérification*<br>*Collectionner, accumuler des objets*	O	N	NSP
**E**	**Frénésies alimentaires ou hyperoralité et changement de préférences alimentaires**			

9	Changement de préférences alimentaires, e.g.<br>*manies alimentaires (habitudes changeantes)*<br>*envies de glucides (particulièrement ceux sucrés)*	O	N	NSP	
10	Grignotage compulsif ou hyperoralité, e.g.,<br>*Boulimie ou continuer à manger même après satiété*<br>*exploration orale ou consommation d'objets non-comestibles*	O	N	NSP	
**SCORE**					
			**TOTAL**		*/10*

**SYMPTÔMES**	
➲ Veuillez cochez la case si au moins un des symptômes était présent dans chacune des catégories suivantes.	
**A. Désinhibition comportementale**	
**B. Apathie, Inertie**	
**C. Perte de sympathie ou d'empathie**	
**D. Comportement de persévération, stéréotype, compulsif ou de rituel**	
**E. Hyperoralité et changement de préférences alimentaires**	

Dépistages de psychose SLA

➲ Veuillez interroger l'aidant au sujet des symptômes possibles suivants. Cochez oui, non, ou ne sait pas. Si oui, décrivez brièvement. Donnez 1 point pour chaque réponse oui (maximum de 3).

1	A des comportements ou croyances bizarres	O	N	NSP
2	Entend ou voit des choses qui ne sont pas là, et/ou ressent la présence de quelqu'un qui n'est pas là	O	N	NSP
3	Est excessivement suspicieux, et/ou se sent persécuté	O	N	NSP
**SCORE**				
	**TOTAL**			*/3*

**DÉBUT ET DURÉE DES SYMPTÔMES**		
➲ Veuillez cocher ou compléter la case pour indiquer la réponse.		
**1. Est-ce que ces symptômes représentent un changement par rapport au comportement passé du patient?**	O	NSP
Si oui, les changements ont-ils eu lieu:		
a. AVANT le début de la maladie?	O	NSP
b. Au même moment?	O	NSP
c. APRÈS le début de la maladie?	O	NSP
**2. Ces symptômes persistent-ils?**	O	NSP
**3. Si non, combien de temps ont-ils duré?**		

Appendix 9.2.7: ECAS Guidance A, B, C [ENGLISH]

Found in [ECAS Guidance A, B, C (EN, V1.1, 2020) - CAPTURE ALS]

EDINBURGH COGNITIVE AND BEHAVIORAL ALS SCREEN – UNIVERSITY OF PENNSYLVANIA (ECAS-PENN)
Administration and Guidance Notes for American English Forms A, B, & C
Version 1.1 Revised (2020)

Developed by Sharon Abrahams and Thomas H. Bak, University of Edinburgh

Adapted for use in North America by Katya Rascovsky, Corey McMillan, and Murray Grossman, University of Pennsylvania, in collaboration with Michael Benatar on behalf of the Clinical Research in ALS and Related Disorders for Therapeutic Development (CReATe) Consortium.

The ECAS is a practical screening tool that incorporates a range of short cognitive tests shown to be sensitive to cognitive impairment in ALS. The ECAS has been designed to differentiate between the different profiles common with aging including depression, Alzheimer's disease and Frontotemporal Dementia. Executive Functions, Memory, Language, Visuospatial skills and Social cognition are specifically assessed while a Behavioral and Psychosis brief interview can be carried out with caregivers or relatives. The ECAS is designed for ALS patients and answers can be given verbally, or by a combination of writing or pointing. It is suitable for patients who are anarthric or patients who have no hand motor function. The total score is 136 points and should take no longer than 15 minutes to administer. Specific guidance on the administration of ECAS-PENN can be obtained by contacting katyaras@mail.med.upenn.edu.

Equipment required:

To carry out the ECAS, you will need a clock or a watch with a second hand (though a stopwatch would be preferable). A calculator is recommended for calculations (though these can also be carried out by hand). Answers can be written or spoken, though should be spoken where possible. If answers are to be written, extra sheets of paper and a selection of pens that will suit the person's writing ability will be required.

Informant:

Please note that an informant is required to complete the Behavior Screen and Psychosis Screen. This could include the caregiver of a patient, a relative of a participant, or close friend. In circumstances where an informant is not available please use the Alternative Behavior Checklist and Alternative Psychosis Checklist to collect data about behavior and psychosis. However, these alternative forms *may not* be used in the generation of ECAS scores and are for data collection purposes only.

Demographics:

Occupation: Indicate highest level of occupation achieved in lifetime (i.e. not necessarily most current occupation or retired)

Handedness: Indicate right, left, or non-dominant.

Years of Education: please enter both total years of education completed (include all forms, including trade school, for example).

Highest Completed Degree: Indicate the highest-level of completed degree (i.e., high school, two-year degree, college/university, master's degree, professional degree (e.g., PhD, MD, JD)).

Language Difficulties: Please ask the participant "Did you have any difficulties reading or learning to write in school?" and write "yes" or "no". If the participant answers with a specific type of language impairment (e.g., dyslexia) please also document this.

Language – Naming: Score 0-8

Administration: There are eight pictures displayed. Ask the person to name the objects shown. No time limit is enforced for the task. Incorrect answers are recorded. A correct score will only be given if the exact name is said or spelled correctly. Self-corrections are allowed; only the final answer is taken for scoring. Do not prompt for an alternative name once a final response is provided.

Scoring: One point is given for every correct name given. **Correct answers (and acceptable alternatives)** are (left to right, top to bottom):

Form A: **scorpion, bow (ribbon), helicopter, fox (wolf/coyote), axe (hatchet), squirrel, swan, and accordion (squeeze box).**

Form B: **peacock, vest (waistcoat), tractor, zebra, wrench, hedgehog (porcupine), owl, and tambourine.**

Form C: **ladybug, bracelet, trailer (camper/RV), butterfly, saw, octopus, parrot, and saxophone.**

Language – Comprehension: Score 0-8

Administration: Using the pictures from the Language - Naming task, ask the person to indicate (e.g. point or say) the correct answer to the questions. Questions are either read by the person or read to the person, depending on preference. Write down the participant's response, whether correct or incorrect. Some questions will require the repetition of an earlier item to serve as an answer; participants are not warned about this in advance but this can be clarified if queried. If no answer is provided, answer space is to be left blank. Self-corrections are allowed; only the final answer is taken for scoring.

Scoring: One point is given for every correct answer given. **Correct** answers are (left to right, top to bottom):

Form A: **helicopter, swan, squirrel, axe (hatchet), helicopter, axe (hatchet), scorpion, and squirrel.**

Form B: **vest (waistcoat), peacock, tambourine, tractor, wrench, hedgehog (porcupine), tractor, and zebra**

Form C: **butterfly, saw, parrot, trailer (camper/RV), bracelet, saxophone, saw, and octopus.**

Note: If the participant incorrectly *names* an item in the Language - Naming Section, but *identifies* the correct item with the wrong name in the Language - Comprehension Section, score as correct. For example, if the participant incorrectly names item one, 'Scorpion', as 'Lobster' in the Language – Naming Section, but answers 'Lobster' when asked *"Something with a sting",* score as correct. In this instance, the person does not know the name of the item but does comprehend what it is. If, however, someone incorrectly names an item in the Language – Naming Section (e.g., 'Scorpion' named as 'Lobster') and then correctly names it in this Language – Comprehension Section do not retrospectively correct the Language – Naming response but correctly score as correct in this section.

Memory – Immediate Recall: Score 0-10

Administration: Say: *"I am going to read you a short story. Please listen carefully. When I am finished, say or write as much as you can remember."*

The story should be read at a steady pace of 2 words per second/the story should take around 20 seconds to read out. When finished reading story aloud, say to participant ***"Now that's the end of the story, what can you remember?"*** Time for recall is unlimited, until participants say that they can remember no more. Self-corrections are allowed; only the final answer is taken for scoring.

Scoring: Score 1 point for each acceptable response in the tables below. Note that number information must always be recalled accurately, for example "Forty two people" recalled as '*forty something*' would score 0 points. This immediate recall score will also be used later on to calculate the percentage of memory retained over time. **No prompts should be given for specific information, only "is that everything you can remember?" should be asked to confirm the participant is finished with recall.**

Form A: Score 1 point for each acceptable response.

Item	Acceptable responses
Sunday	Only 'Sunday' is accepted.

Annual Park Cleanup	'annual park cleanup', 'annual cleanup', 'garbage cleanup', 'park cleanup', or 'annual trash cleanup' are accepted.
Marigold Woods	Score correct if any part of 'Marigold Woods' is recalled verbatim. Additional items may be semantically similar e.g., 'forest', 'park'.
Forty two	Only the exact number 'Forty-two' is accepted.
Bicycles and Shopping Carts	A semantically similar response is required for each item like replacing 'shopping carts' with 'carts' or 'trolley'. A score of zero if no mention of something related to each of bicycles and no mention of something related to shopping carts.
Robert Webber	Either 'Robert' and/or 'Webber' is accepted.
Woodland Project	'Woodland' is required but semantically similar alternative are acceptable for 'project' such as 'plan'.
Impressed and Especially Proud	Any positive emotional response like 'pleased' is accepted.
17	Only the exact number 'Seventeen' is accepted.
Children	'Children' or responses with similar meaning are accepted e.g., 'kids'.

Form B: Score 1 point for each acceptable response.

Item	Accepted responses
Three	Only the exact number 'Three' is accepted.
Fishing boats	'Fishing' and/or 'boats'. If the participant correctly identifies one meaning of this item, score as correct e.g., 'fishermen' is accepted, as is 'sailing boats'.
Helped rescue	'Helped', 'rescue', or 'saved' are accepted
Whale	Only 'whale' or 'whales' are accepted
Shore	'Shore' or responses with similar meaning are accepted e.g., 'near the beach', or 'just off the coast' are accepted
Circles	Close synonyms or responses with similar meaning should be scored as correct e.g., 'the whales were going round and round'.
Alan Williams	Either 'Alan' and/or 'Williams' is accepted.
Marine conservation trust	Score correct if any part of 'marine conservation trust' is recalled verbatim. Additional items may be semantically similar e.g., animal, wildlife, nature, board, trust, group, agency, council.
Thirty-two	Only the exact number 'thirty-two' is accepted.
Last winter	'Last winter', 'this winter', 'previous year', or 'last year' are accepted. 'Last Summer' or 'this year' should not be accepted.

Form C: Score 1 point for each acceptable response.

Item	Acceptable responses
Helen Blake	Either ‘Helen’ and/or ‘Blake’ is accepted.
Boston	Only ‘Boston’ is accepted.
Northern	Only ‘Northern’ or ‘north’ are accepted.
Prize	‘Prize’ or a semantically-related alternative like ‘award’, or ‘competition’ are required. ‘Art’ or a semantic associate on its own is not accepted.
Photography	‘Photography’, ‘photographs’, ‘photographer’ are accepted.
Forty-seven	Only the exact number ‘Forty-seven’ is accepted.
Hiking	‘Walking in the country’ is accepted, whereas just ‘walking’ is not.
Seven hundred	Only the exact number ‘Seven hundred’ is accepted.
Oak tree	Must mention ‘Oak’, or ‘oak tree’ is accepted. ‘Tree’ or ‘forest’ alone is not accepted.
Autumn colors	‘Autumns colors’, ‘Autumn leaves’, ‘autumnal’ or just ‘autumn’ are accepted. ‘Fall’ is an accepted alternative to Autumn. ‘Golden leaves’ is not accepted.

Language – Spelling: Score 0-12

Administration: Say *“Spell, either by writing or speaking, the following words.”*

If the person is using assistive technology, ask them to turn off any predictive text facility. Unlimited time is given for spelling of each word. All words are assessed even if early words in the list are incorrect. Move on to the next word if participant is unable or unwilling to attempt spelling of one word. The word may be clearly stated by the interviewer several times if necessary. For example, if the participant spells ‘constructing’ as ‘construction’, restate the word clearly. Similarly, if the plural of the word is spelled e.g., ‘biscuits’ instead of ‘biscuit’, restate the target word clearly.

Special Instructions:

Form A: it may be necessary to clarify item 12, ‘brought’. If so, say: “I brought the gift to the party”.

Form B: it may be necessary to clarify item 12, ‘thought’. If so, say: “I thought dinner was at eight o’clock”.

Form C: It may be necessary to clarify item 12, ‘wrong’. If so, say: “You called the wrong number”. Please ensure that ‘schedule’ is pronounced ‘sked-ule’ for North America, and not ‘shed-ule’. If participant incorrectly spells ‘wheelbarrow’ as ‘wheel barrel’, please say ‘I would like for you to spell wheelbarrow’ ensuring to pronounce the word clearly.

Scoring: Score 1 point for each correct spelling. Self-corrections are allowed; only the final answer is taken for scoring. Do not penalize if the participant inserts a space in the spelling of the words (e.g., coathanger, toothpick, or babysitter). Caution should be taken in interpretation where low premorbid IQ or a history of reading or spelling difficulties is reported. Please remember to ask the participant and/or informant about premorbid history of language difficulties, as instructed at the top of each ECAS form.

Fluency - Letter: Score 0-12

Administration: The person can perform this task either by speaking or writing. Say: *"I am going to give you a letter of the alphabet and I would like you to say or write as many words as you can beginning with that letter, but not names of people or places, or numbers."*

Form A:

- Speaking: "You will have one minute and the letter is S."
- Writing: "You will have two minutes and the letter is S."

Form B:

- Speaking: "You will have one minute and the letter is F."
- Writing: "You will have two minutes and the letter is F."

Form C:

- Speaking: "You will have one minute and the letter is P."
- Writing: "You will have two minutes and the letter is P."

Next the person copies/repeats these words.

- All answers provided are recorded and copied/repeated (i.e., do not remove repetitions, intrusions, errors).
- If speaking, ensure the participant can read the words you have written, then say: *"Read aloud these words as fast as possible. Before you do this, check that you can read them. I will time you. Ready? Begin."*
- If writing, say: *"Copy these words as fast as possible. I will time you. Ready? Begin."*

Scoring (all forms): Although all answers are recorded in the administration, the following rules apply for scoring items as correct:

- Words must be varied for example: *sugar, salt, slipper, snow, scream, shoot, scale, scissors…*
- Do not include repetitions, nonsense words (i.e., cannot be found in an English dictionary), or proper names in scoring items correct.
  - When a second meaning is provided or indicated each word is scored as an independent item. For example:
    - Apparent repetition of a word, but a second meaning is provided (e.g. '*Fine*' – indicating something is high quality, or '*Fine*' – a monetary penalty).
    - Items that are spoken and a different spelling/meaning is indicated (e.g. *paced* and *paste*).
    - Items in which a meaning change is indicated (e.g. *savor* and *savory*)
  - When a word is related but a different grammatical part of speech (e.g., *final* is a verb and *finally* is an adverb), each word is scored as an independent item.
  - Plural words will be accepted, only if they have not already been provided in singular form (e.g., *paper, papers* = score 1). If both a singular and plural are provided only the first word is scored.
  - If conjugations of verbs are provided (e.g., sit, sat) only the first word is scored.

In order to account for differences in motor speed and speaking time variations, a Verbal Fluency Index (VFI) is calculated using the equation below.

$$VFI = \frac{Test\ time - time\ taken\ to\ repeat\ words}{Number\ of\ correct\ words\ generated}$$

For example, a participant given 60 seconds to complete the task who generates 5 words and then takes 15 seconds to read these words aloud would have a VFI of 9:

$$VFI = \frac{60 - 15}{5} = 9$$

A participant's VFI is converted to a Fluency score using the conversion table provided in ECAS test, page 2.

Executive – Reverse Digit Span: Score 0-12

Administration: Numbers should be read out at a pace of 1 number per second. Say: *"I am going to say some numbers and I would like you to say them back to me in reverse order. For example, if I say '2 3 4', you should say '4 3 2'. Let's practice. If I say '7 1 9', what would you say?"*

If the participant makes an error on the practice trial, they are corrected, and the first trial of the test begins. If the participant fails the first two items then the test is scored at 0 and no further items are attempted. Advance warning that the number sequences will increase in length is provided at the start of each line of a trial. In order to score a trial of a line as correct, participant must accurately recall *all* items in reverse order. No score is given for individual numbers correctly recalled in an incorrectly recalled sequence. If person gets at least one trial of a line correct, move on to the next line. Self-corrections are allowed; only the final answer is taken for scoring. Stop when person gets both trials of a line wrong.

Scoring: Score is total number of trials achieved correctly (out of 12).

Executive – Alternation: Score 0-12

Administration: Say: *"I want you to alternate between numbers and letters, starting with 1-A, then 2-B, 3-C, and so on. Please continue from there, alternating between numbers and letters, in order, without skipping any until I tell you to stop. Let's begin together: 1-A, 2-B, 3-C…".* Having started with the participant, let them continue the sequence alone. If the participant alternates with the letter first and number second (e.g., D-4, E-5) continue the administration since the sequential ordering is preserved. Stop administration after the participant makes an error.

Scoring: One point is given for every correct trial. Score as correct whether the participants first response initiates with a letter then number (e.g., *D-4*, *E-5*, …) or a number then a letter (e.g., *4-D*, *5-E*).

Fluency – 4-Letter Words: Score 0-12

Administration: The person can perform this task either by speaking or writing. Say: *"I am going to give you a letter of the alphabet and I would like you to say or write as many words as you can beginning with that letter.* ***But not names of people or places, or numbers***. ***This time the word must only be four letters long. No more or less than four letters***.*"*

Form A:

- Speaking: "You will have one minute and the letter is T."
- Writing: "You will have two minutes and the letter is T."

Form B:

- Speaking: "You will have one minute and the letter is D."
- Writing: "You will have two minutes and the letter is D."

Form C:

- Speaking: "You will have one minute and the letter is M."
- Writing: "You will have two minutes and the letter is M."

Next the person copies/repeats these words. If writing, say: *"Copy these words as fast as possible. I will time you. Ready? Begin."* If speaking, say: *"Read aloud these words as fast as possible. Before you do this, check that you can read them. I will time you. Ready? Begin."*

Scoring: Plurals are accepted in order to create four letters – for example, *Toes*. Contractions are also accepted – for example, *don't*. See scoring criteria from previous Fluency task to produce VFI, and conversion table provided in ECAS test packet, page 3.

Visuospatial – Dot counting: Score 0-4

Administration: Say *"I would like you to count how many dots are in each box, but without pointing to them"*. Progress from left to right and top to bottom to move through the squares. All squares should be attempted. If the participant begins pointing directly at the dots, remind them of the instructions. If participants begin pointing away from the dots (e.g., in the air, or on their lap), do not correct them.

Scoring: One point for each correct box. **Correct** answers on *all forms* are**:**

Top left **10** Top right **8**

Bottom left **7** Bottom right **9**

Visuospatial – Cube counting: Score 0-4

Administration: Ask the person *"How many cubes are in each structure, including the ones you may not be able to see?"* Progress from left to right and top to bottom to move through the cube structures. All structures should be attempted. If they do not understand the instructions or ask for clarification, please only repeat the instructions verbatim.

Scoring: one point for each correct answer. **Correct** answers are:

Form A: top left **5**, top right **6**, bottom left **10** and bottom right **7**

Form B: top left **5**, top right **6**, bottom left **10** and bottom right **7**

Form C: top left **5**, top right **6**, bottom left **8** and bottom right **7** (note bottom left differs from prior Forms)

Visuospatial – Number location: Score 0-4

Administration: Ask the person *"Which number corresponds to the position of the dot?'* Progress from left to right and top to bottom to move through the squares. All squares should be attempted. If participants fail to understand the instructions, you may clarify the instructions by saying *"if you were to lift the bottom box and place it over the top box, which number would the dot cover?"*

Scoring: One point for each correct answer. **Correct** answers are**:**

Form A: top left **6**, top right **5**, bottom left **2**, bottom right **3**.

Form B: top left **7**, top right **8**, bottom left **4**, bottom right **3**.

Form C: top left **1**, top right **8**, bottom left **5**, bottom right **7**.

Executive – Sentence completion: Score 0-12

Administration: Say *"Listen carefully to these sentences and as soon as I have finished reading them, please tell me, or write, a word that finishes the sentence as quickly as possible."* For example, *'She was so tired that she went straight to…bed'*. Do not score the first two questions. Now say: *"I'd like you to do that again, but this time the word you give should not make sense whatsoever in the context of the sentence. It must not be related to the word that actually completes the sentence.* For example, *'John cut his hand with the sharp…orange'*. If the participant answers with a word which completes the sentence in context then remind them that the requirement is to provide an answer that bears no significance to the context of the sentence. If the person does not respond within 20 seconds, move onto the next question. If an incorrect response is provided, only re-prompt the participant with instructions once (after the first error) during administration. If the participant immediately responds to the same prompt with another word, score only the initial response before the reminder and proceed to the next sentence. If a participant repeats a word from a prior trial score as incorrect and ask them not to repeat a word for the other sentences.

Scoring: Give 2 points for completely unconnected word, 1 for related word (e.g. associated or opposite meaning) and 0 for exact word. See table below for scoring examples. Note: sentences can be non-grammatical.

Form A:

	**Item**	**2 points**	**1 point**	**0 points**
1	The mailman knocked on the…	Car, potato…	Window, gate, mailbag…	Door
2	He brought his umbrella with him in case of…	Rubber, parachute…	Sunshine, wind, ice…	Rain
3	Sally spread her toast with butter and…	Earth, sand…	Cereal, egg, oranges…	Jam, Marmalade, Honey, Cheese, jelly
4	John went to the barber and got his hair…	Moon, table…	Washed, lengthened, singed…	Cut
5	She dived into the swimming…	Garden, swing…	Pond, bath, rock…	Pool
6	They all went to the local café for something to…	Jump, dance…	Do, play, buy…	Eat, drink.

Form B:

	**Item**	**2 points**	**1 point**	**0 points**

1	She answered the phone because it was...	Purple, dangerous, sunny...	Silent, talking...	Ringing, buzzing...
2	The joke was so funny, he started to ...	Undress, walk, disappear...	Cry, snore, fall asleep...	Laugh, giggle, chuckle...
3	Daniel unlocked the door with a ...	Balloon, melon, spanner...	Hammer, penknife...	Key
4	The child cut paper with a pair of...	Shoes, glasses, bananas...	Hands, pliers...	Scissors, shears..
5	After months of practice, Lisa passed her driving...	Force, show, night...	Trial...	Test.
6	Simon ate his dinner with a knife and ...	Nose, paper, pencil...	Spade, shovel, toothpick...	Fork, spoon.

Form C:

	**Item**	**2 points**	**1 point**	**0 points**
1	Lisa went to the library to return some ...	Rice, worm, umbrella...	Videos, DVDs...	Books, magazines...
2	After her shower, she dried herself with a ...	Sandpaper, box, lipstick...	Water, mop...	Towel, sheet.
3	He put a teabag in his mug and boiled the ...	Petrol, spaghetti, orange juice...	Milk...	Kettle, water...
4	He studied medicine to become a ...	Plumber, engineer, carrot...	Researcher, nurse...	Doctor, psychiatrist, GP
5	The music started and everyone got up to...	Brush teeth, fly, cry...	Sit, sleep, shout, talk, yawn...	Dance, drink, go home...
6	John picked up the leash and took his dog for a...	Step class, cremation, flight...	Bath, swim, drive, game of football...	Walk, run...

Social Cognition – Part A

Administration: The first page contains six boxes each with four pictures in each corner.

Say: *"You are going to see some pictures, one in each corner of a box. You have to choose which picture you like best. Either point to or say which picture you like best. Please respond as quickly as possible."* When guiding participants through the boxes, avoid pointing to any particular corner/picture.

Scoring: **Answers are recorded but not scored;** responses are used as information to support scoring in the next section.

Social Cognition – Part B: Score 0-12

Administration: Say: *"You are going to see some pictures, one in each corner of a box. You have to choose which picture* ***does the face like best.*** *Either point to or say which* ***the face likes best****. Please respond as quickly as possible."* Progress through all questions even when incorrect answers are provided. Test instructions can be repeated exactly as written, but **DO NOT amend or add to the instructions for this test, for example, do not say 'which is the face looking at'.** When guiding participants through the boxes, avoid pointing to any particular corner/picture.

Scoring: Two points for each correct response. Of the items not correctly identified, score 1 point if answer was NOT the item that participant picked as their own favorite in the previous section, score 0 points if the item WAS picked as their own favorite.

Memory – Delayed recall: Score 0-10

Administration: Say: *"At the beginning of this interview, I read you a story. Tell me as much as you can remember from that story."* Time for recall is unlimited, until participants say that they can remember no more. Self-corrections are allowed; only the final answer is taken for scoring.

Scoring: Score 1 point for each acceptable response described in Intermediate Recall, see above tables. The percentage of memory retained is now calculated. Take the total score for **Delayed Memory and divide it by the Immediate Memory score, before multiplying this number by 100.** For example:

- With a Delayed Memory recall of 8 items, and an Immediate Memory Score of 9/10
- The percentage of retained memory is 89%.

Some participants may recall more at delay than immediate and the percentage will be displayed as being over 100%. **Use the conversion table provided in ECAS to derive scoring.**

Memory – Delayed recognition: Score 0-4

Administration: This test should only be done if the person failed to recall one or more items. If all the items were recalled, skip the test and score 4.

Otherwise, say: *"Let's see if you can remember anything more about that story. I will ask you some questions, please tell me if they are true or false".*

Scoring: Score 1 point for each correct answer; correct answers are marked in bold in this section. If the person gives a "**don't know**" answer ask them to make a guess on True or False and score accordingly. **Use the conversion table provided in ECAS to derive final scoring for recognition section.**

SCORES

**Language**	Naming, Comprehension, Spelling	/28
**Verbal Fluency**	Fluency Letter S, Fluency Letter T	/24
**Executive**	Reverse Digit Span, Alternation, Sentence Completion, Social Cognition	/48
	**ALS-SPECIFIC:**	**/100**
Memory	*Immediate recall, Delayed retention, Delayed recognition*	*/24*
Visuospatial	*Dot Counting, Cube Counting, Number Location*	*/12*
	ALS NON-SPECIFIC:	***/36***
	**ECAS TOTAL SCORE**	**/136**

CUT OFF SCORES FOR ABNORMALITY (BASED ON EDINBURGH NORMS):	
**ECAS TOTAL SCORE**	**105**
**ALS-SPECIFIC**	**77**
**ALS NON-SPECIFIC**	**24**
**Language**	**26**
**Verbal Fluency**	**14**
**Executive**	**33**
**Memory**	**13**
**Visuospatial**	**10**

ALS Caregiver Behavior Screen
Guidance and Administration
American English Version

Guidance:

Please complete this interview with the carer or relative in private from the participant, ideally in a separate room. There are five components to this screen. Some people may have noticed a change in all areas, some in a few, and others may note there are no changes with the participant. Please ask the carer or relative to give any examples if possible, which should be recorded on the form. The ECAS Behavior Screen is interview based and should therefore be complete with the carer or relative. Please remind the informant that *symptoms may have been present before* or after the onset of ALS.

As indicated above, **under no circumstances should this form be given to the carer or relative to complete on their own.** In circumstances where an informant is not available please use the Alternative Behavior Checklist and Alternative Psychosis Checklist to collect data about behavior and psychosis.

Please note, however, that these alternative forms *may* ***not*** be used in the generation of ECAS scores and are for data collection purposes only.

Administration – Score 0-10

Please ask the informant about the listed possible behaviors. Symptoms should have occurred **repeatedly** and not just on one instance, and may have occurred prior to the development of any motor signs. Check 'Yes', 'No' or 'Don't Know'. If 'Yes', please provide a brief written description. Give one point for every 'Yes' response (maximum = 10).

ALS – Psychosis Screen
Guidance and Administration
American English Version

Guidance: Please ask the informant to complete this section away from the participant, ideally in another room. Please remind the informant that the questions asked are *only relevant since the onset of ALS*. If the answer is Yes to any question, please note any relevant examples or comments.

Administration- Score 0-3

Please ask the informant about the following possible symptoms. Check 'Yes', 'No' or 'Don't Know'. If 'Yes', please provide a brief written description. Give one point for every 'Yes' response (maximum = 3).

Appendix 9.2.8: ECAS Guidance A, B, C [FRENCH]
Found in [ECAS Guidance A, B, C (FR, 16May2020) - CAPTURE ALS]

ECHELLE COGNITIVE ET COMPORTEMENTALE D'EDIMBOURG DE DÉPISTAGE DE LA SLA
ECAS Formulaire A (Version Français Canada 2018)

Procédures de passation

Developed by S. Abrahams and T.H. Bak, University of Edinburgh
Adapted for use in North America by K. Rascovsky and M. Grossman, University of Pennsylvania

L'échelle ECAS est une échelle comportant un certain nombre des tests cognitifs qui se sont révélés sensibles pour la détection de troubles cognitifs dans la SLA. Cette échelle permet de contribuer à différencier différents profils cognitifs du sujet âgé, lors de la dépression, de la maladie d'Alzheimer ou de la démence fronto-temporale. Les fonctions exécutives, la mémoire, le langage, les capacités visuo-spatiales, la cognition sociale sont évalués ainsi que le comportement à l'aide d'un entretien avec l'aidant ou le conjoint. L'échelle ECAS a été spécialement élaborée pour les patients SLA. Les réponses pouvant être données oralement, ou par écrit et désignation. Cela est adapté au patient anarthrique ou présentant un déficit moteur. Le score total est de 136 points et cette évaluation est réalisée en 25 min environ (compter plus de temps si le test est réalisé par écrit).

Matériel nécessaire:
Pour faire passer l'ECAS, vous devez vous munir d'un chronomètre. Une calculatrice est recommandée (mais les calculs peuvent se faire sans). Les réponses peuvent être écrites ou données oralement, **la réponse orale est à privilégier** (si elle est possible). Les réponses données par écrit seront récupérées sur une feuille séparée. Il importe de se munir de stylos permettant de s'adapter au mieux aux possibilités du patient.

Informateur :

Un informateur externe est requis pour compléter le dépistage de Comportement et de Psychose. Ce peut être le proche aidant du patient, un proche ou un ami. Si un informateur n'est pas disponible merci d'utiliser la liste alternative du Comportement et de Psychose pour collecter des données sur le comportement et la psychose. Ces formulaires alternatifs ne peuvent pas être utilisés pour la génération de scores ECAS et ont pour unique but la collecte de données.

Démographie :

Occupation : indiquer le plus haut niveau occupé dans la vie – ne reflète pas nécessairement la position occupée la plus récente ou bien la retraite.

Main utilisée : indiquer Droit, Gauche, ou non dominant.

Années d'éducation : merci d'indiquer le total des années d'éducation complétées (en incluant toutes les formes d'éducation).

Diplôme complété le plus élevé : merci d'indiquer le niveau le plus élevé de diplôme complété. (ex : CEGEP, BSc, MSc, PhD…)

Difficulté de langue : merci de demander au participant « Avez-vous eu des difficultés de lectures ou d'apprentissage de l'écriture à l'école? » et répondre par Oui ou Non. Si le participant donne une réponse spécifique comme par exemple la dyslexie, merci de documenter la réponse.

LANGAGE - Appellation: SCORE 0-8

Consigne: Il y a huit dessins proposés. Demander au patient de nommer ces dessins. Aucune limite de temps n'est imposée pour cette tâche. Les réponses incorrectes sont notées et aucune proposition de correction n'est proposée. La réponse est correcte que si le mot exact est prononcé ou écrit. Des auto-corrections sont acceptées et seule la réponse finale sera prise en compte pour la cotation. Si la réponse donnée est correcte mais ne correspond pas à la réponse attendue comme par exemple 'ruban' pour 'noeud', relancer en disant « connaissez-vous un autre nom pour cette image ? ». Si les réponses correspondent

à un mot moins fréquent mais juste, comme 'hachette' pour 'hache', coter la réponse comme correcte. Ne proposez pas au participant d'autre nom alternatif une fois qu'une réponse finale est donnée.

Cotation: Un point par réponse correcte donnée. Si les réponses sont produites par écrit, ne pas pénaliser les fautes d'orthographe tant que le bon mot est produit. **Les réponses correctes (et les alternatives acceptables) sont (de gauche à droite et du haut vers le bas) : scorpion, noeud, hélicoptère, renard, hache, écureuil, cygne, accordéon.**

LANGAGE - Compréhension: SCORE 0-8

Consigne: Avec la planche de dessins utilisée pour le test précédent, demander au patient d'indiquer en pointant ou bien en disant à l'oral la réponse correcte aux questions posées. Les phrases peuvent être lues par le patient ou par l'examinateur. Il y a plusieurs fois les mêmes réponses, le patient n'en est pas informé à l'avance mais cela peut lui être précisé s'il pose la question. En cas de non réponse, ne rien écrire sur l'espace dédié. Des autocorrections sont acceptées, et seule la réponse finale sera prise pour la cotation.

Cotation: Un point par réponse correcte donnée. **Les réponses correctes sont : hélicoptère, cygne, écureuil, hache, hélicoptère, hache, scorpion, écureuil.**

Note : Si le participant ne nomme pas correctement un item dans la section « Appellation » mais qu'il identifie le bon item avec le mauvais nom dans la section « Compréhension », coter comme bon. Exemple : si le participant nomme incorrectement l'item 1, « Scorpion », comme « Homard » dans la section Appellation, mais qu'il répond « Homard » quand on lui demande « Quelque chose avec un dard? », coter comme bon. Dans cet exemple, le participant ne connait pas le nom de l'item mais comprend de quoi il s'agit.

Cependant, si le participant nomme incorrectement un item dans la partie Appellation (ex : Scorpion nommé Homard) puis ensuite le nomme correctement dans la partie Compréhension : ne pas corriger rétrospectivement la réponse d'Appellation mais coter bon la section Compréhension.

MEMOIRE - Rappel immédiat: SCORE 0-10

Consigne: Dites: '*Je vais vous lire une histoire courte. Veuillez l'écouter attentivement. Lorsque j'ai fini, dites ou écrivez autant que vous le pouvez sur l'histoire.*'

Le rythme de lecture est de 2 mots par seconde. L'histoire doit être lue en 20 secondes en moyenne. A la fin de la lecture, dire au patient : '*l'histoire est terminée, de quoi vous rappelez-vous ?*'.

Il n'y a pas de limite de temps. Le test est arrêté quand le patient dit ne pas se rappeler d'autres éléments. Des autocorrections sont acceptées et seule la réponse finale sera prise pour la cotation.

Cotation: un point pour chaque mot ou groupe de mots soulignés rappelés (en entier ou une partie).. Voir ci-dessous les réponses alternatives acceptables. Les nombres doivent être rappelés correctement, par ex : *42 personnes* rappelé comme *quarante et quelque chose*, est incorrect,et sera cotée 0. Cf. tableau ci-dessous. Le score de rappel immédiat obtenu servira aussi plus tard pour le calcul du pourcentage de ***mémoire différée.*** Ne pas donner d'indices lors du rappel, mais s'assurer que le patient a fini de restituer l'histoire en lui demandant « est-ce tout ce dont vous pouvez vous souvenir ? ».

ECAS 1 : coter 1 point pour chaque section soulignée retenue (entière ou partielle)

Section	Réponses acceptables
Dimanche	Seulement « dimanche » est accepté
Nettoyage annuel du parc	« nettoyage annuel du parc », « nettoyage annuel », « nettoyage des déchets », « nettoyage du parc » ou bien « nettoyage annuel des déchets » sont acceptés
Bois Marigold	Coter bon si n'importe quelle partie de « bois Marigolg » est retenue. D'autres items sémantiquement similaires sont acceptables comme « forêt », « parc ».
Quarante-deux	Seulement le chiffre exact « quarante-deux » est accepté.
Vélos et paniers d'épicerie	Une réponse sémantiquement similaire est nécessaire pour chaque item comme remplacer « paniers d'épicerie » par « chariot » ou « carrosse ». Coter 0 si aucune mention de quelque chose relié à Vélo et aucune mention de quelque chose relié à Paniers d'épicerie.
M.Robert Webber	« Robert » et/ou « Webber » sont acceptables.
projet Woodland	« Woodland » est requis mais des alternatives sémantiquement correctes sont acceptables pour « Projet » comme par exemple « plan ».
Impressionné et particulièrement fier	N'importe quelle réponse émotionnelle positive est acceptée, comme « content », « heureux », etc…
17	Seulement le chiffre exact « 17 » est accepté.
enfants	Sont acceptés « enfants » mais aussi des réponses au sens similaire comme « gamins ».

LANGAGE – Orthographe: SCORE 0-12

Consigne: Dites: 'Veuillez épeler à haute voix sur papier les mots suivants.' Si la personne utilise une technologie d'assistance, demandez-lui d'éteindre toute fonction prédictive.

Il n'y a pas de limite de temps. Tous les mots doivent être *évalués*, même en cas d'erreurs sur les premiers mots. Passer au mot suivant si le patient est incapable ou refuse de répondre. En cas de difficulté lors de l'épellation orale, autoriser le patient à lire en même temps le mot écrit par l'examinateur. Si nécessaire, le mot peut être énoncé clairement plusieurs fois par l'évaluateur. Par exemple, si le participant épelle « Construisant » au lieu de « Construction », répéter clairement le mot. De la même manière, si le pluriel d'un mot est épelé, répéter clairement le mot.

Instructions particulières : Il peut être nécessaire de préciser pour les items 8 et 12 les mots dans un contexte de phrase pour différencier « enregistré » de « enregistrer » et « apporté » de « apporter ».
Exemple : J'ai enregistré un album / J'ai apporté du pain.

Cotation: Un point pour chaque mot correctement épelé ou écrit. Des autocorrections sont acceptées, et seule la réponse finale sera prise pour la cotation. Ne pas pénaliser si le participant insert un espace dans l'épellation des mots (ex : Tourne Vis au lieu de Tournevis). L'existence d'un QI bas prémorbide ou d'antécédent de difficultés de lecture ou d'orthographe doivent être recherchés, pris en compte dans l'interprétation des résultats et notifiés. Se rappeler de demander au participant et/ou à l'informateur s' il y a existence de difficultés de langage ou d'histoire prémorbide, comme indiqué en haut de chaque formulaire ECAS.

FLUENCE VERBALE - Lettre S: Score 0-12

Consigne: Le patient peut réaliser ce test soit oralement soit par écrit. Dites: 'Je vais vous donner une lettre de l'alphabet et j'aimerais que vous disiez ou écriviez autant de mots que vous le pouvez qui commencent par cette lettre. **Les noms propres ou les chiffres ne sont pas acceptés.'**

- Si parlé, dites: 'Vous avez une minute. La lettre est S.'
- Si écrit, dites: 'Vous avez deux minutes. La lettre est S.'

Ensuite, la personne copie ou lit les mots à haute voix.

Les mots seront écrits (par le patient ou l'évaluateur) en colonne sur une feuille préparée avec deux colonnes pour que la copie (à droite) s'appuie sur la production (à gauche). Ne pas enlever les répétitions, les intrusions et les erreurs. Il est recommandé à l'évaluateur de copier les mots en majuscules pour que la lecture par le patient soit possible.

Les réponses incorrectes seront éliminées et ne devront pas être ni lues (épreuve orale) ni recopiées (épreuve écrite) par le patient.

- Si parlé, dites: 'Lisez ces mots à haute voix aussi vite que possible. Avant de faire cela, vérifiez que vous pouvez les lire. Je vais vous chronométrer. Prêt? Commencez.'
- Si écrit, dites: 'Copiez ces mots aussi vite que possible. Je vais vous chronométrer. Prêt? Commencez.'

Cotation: Toutes les réponses fournies sont enregistrées mais les règles suivantes seront appliquées pour déterminer une réponse correcte :

- les mots doivent être diversifiés

- sont cotés comme incorrect:

- les mots absurdes (sans signification), les répétitions, les noms propres
- les mots dérivés (ex : moustache/ moustachu), les persévérations sur un mot comme par exemple un verbe conjugué (à l'infinitif, au participe passé…) alors que le sens n'est pas modifié

- sont cotés comme correct:

- les homographes si un sens différent est précisé par le patient (ex : un *moule* l'objet et une *moule* l'animal)
- les homophones en cas de test réalisé oralement si la différence d'orthographe et/ ou de sens est indiquée par le patient
- un changement de sens quand celui-ci est indiqué (exemple : *montrer* le verbe et *montre* l'objet, si cela est spécifié)
- les différences grammaticales commes entre adverbes et verbes/noms – exemple : « final » et « finalement », côter comme deux items différents
- les mots au pluriel s'ils n'ont pas été donnés précédemment au singulier (ex : « papiers » est accepté si « papier » n'a pas été donné avant. Si le participant dit « papier », puis « papiers » : ne côter comme bon que le premier mot.

Si les réponses sont produites par écrit, ne pas pénaliser les fautes d'orthographe tant que le bon mot est produit.
Exemple de cotation : Merise, menuisier, meuble, *(menuiserie),* maçon, *(maçonnerie),* matériel, manger, *(mangé),* magistrat, *(magistrature),* magique, maison, myrtille, *(magicien),* mûre, mayonnaise, *(maisonnette*)." score = 12
Afin de tenir compte des différences entre les deux formes de test, un Index de Fluence Verbale (**i FV : verbal fluency index)** est calculé selon l'équation suivante :

Calcul de l'index de fluence verbale **i FV** :

$$\textbf{iFV} = \frac{\text{temps du test (en secondes)} - \text{temps de répétition du test (en secondes)}}{\text{Nombre de mots corrects}}$$

Par exemple : un patient qui donne 5 mots corrects en 60 secondes et les relit en 15 secondes, aura un iFV de 9 (60-15/5=9).

Puis cet index (iFV) sera converti en score de fluence en suivant la table de conversion présentée dans l'échelle.

EXÉCUTIF – Empan de chiffres inverse : Score 0-12

<u>Consigne</u>: Les chiffres doivent être lus avec un rythme de 1 par seconde. Dites: 'Je vais vous donner une liste de chiffres et j'aimerais que vous me les répétiez dans l'ordre inversé. Par exemple, si je dis 2-3-4, vous devriez dire 4-3-2. Essayons avec 7-1-9, que me diriez-vous?'
Si le patient ne réussit pas cet essai, un autre essai sera effectué avec seulement deux chiffres, avant de démarrer le test. Si le patient ne réussit pas cet essai à 2 chiffres, le test est coté à 0/12 et aucune autre partie du test n'est tentée. Le patient sera informé à chaque fois qu'il y aura augmentation du nombre de chiffres (à chaque nouvelle ligne). Pour qu'un essai soit coté comme correct, le patient doit répéter *tous* les chiffres en sens inverse. Sera cotée comme incorrecte la répétition de tous les chiffres mais selon une mauvaise séquence. Si le patient réussit au moins un essai sur les deux présentés par ligne, passer à la ligne suivante. Des autocorrections sont acceptées, et seule la réponse finale sera prise pour la cotation. Arrêter après deux échecs consécutifs sur une même ligne. Le patient peut réaliser ce test soit oralement soit par écrit, mais il est recommandé d'essayer de le réaliser oralement. A l'écrit, ne pas autoriser à écrire d'abord les chiffres dans l'ordre ni de les noter de droite à gauche.
<u>Cotation</u>: Le score correspond au nombre total d'essais correctement effectués.

EXÉCUTIF – Alternance: Score 0-12

<u>Consigne:</u> Dites: 'J'aimerais que vous alterniez entre chiffres et lettres, en commençant par 1A, puis 2B, 3C, et ainsi de suite. Veuillez alterner entre chiffres et lettres, dans l'ordre, sans en sauter, jusqu'à ce que je vous dise d'arrêter. Commençons ensemble : 1A, 2B, 3C…' Après avoir commencé avec le participant, laissez-le continuer la séquence tout seul. Si le participant alterne avec la lettre en premier et le chiffre en deuxième (ex : D4, E5,…), poursuivre l'administration du test car l'ordre séquentiel est préservé. Arrêter l'administration après que le patient ai fait une erreur.
<u>Cotation:</u> Un point par essai correct. Côter comme bon même si le participant initie ses réponses par la lettre et non par le chiffre. Pour la version écrite, Ne pas autoriser les stratégies (écrire tous les chiffres puis ensuite les lettres, si le sujet le fait lui dire d'arrêter).

FLUENCE VERBALE - lettre **T**: SCORE 0-12

Consigne: le patient peut réaliser ce test soit oralement soit par écrit. Dites: 'Je vais vous donner une lettre de l'alphabet et j'aimerais que vous disiez ou écriviez autant de mots que vous le pouvez qui commencent par cette lettre. **Les noms propres ou les chiffres ne sont pas acceptés**. **Maintenant, le mot doit être long de quatre lettres. Ni plus, ni moins, que quatre lettres.** A noter que le pluriel du mot est accepté pour faire un mot de 4 lettres.

- Si oral, dites: 'Vous avez une minute. La lettre est T.'
- Si écrit, dites: 'Vous avez deux minutes. La lettre est T.'

Ensuite la personne copie ou lit les mots à haute voix.

- Si parlé, dites: 'Lisez ces mots à haute voix aussi vite que possible. Avant de faire cela, vérifiez que vous pouvez les lire. Je vais vous chronométrer. Prêt? Commencez.'
- Si écrit, dites: 'Copiez ces mots aussi vite que possible. Je vais vous chronométrer. Prêt? Commencez..'

Cotation: suivre les mêmes consignes que celles précédemment appliquées pour le précédent test de fluence (lettre S) pour calculer l'iVF et se référer à la table de conversion présenté dans l'échelle. Les pluriels sont acceptés pour créer des mots de 4 lettres. Exemple : « rats » si la lettre était « R ». Les contractions peuvent également être acceptées.

VISUOSPATIAL – Comptage de points: SCORE 0-4

Consigne: Dites: 'J'aimerais que vous comptiez le nombre de points dans chaque boîte, mais sans les pointer.' Aller de la gauche vers la droite et du haut en bas. Tous les carrés doivent être analysés.

Cotation: un point par réponse correcte.

Les réponses correctes sont : en haut à gauche : 10 ; en haut à droite : 8 ; en bas à gauche : 7 ; en bas à droite : 9.

VISUOSPATIAL – Comptage de cubes: SCORE 0-4

Consigne: Dites: 'Combien de cubes y a-t-il dans chaque structure, incluant ceux que vous n'êtes pas capable de voir?' Aller de la gauche vers la droite et de haut en bas. Tous les dessins doivent être analysés. Si le participant ne comprend pas, répéter seulement les instructions et ne prodiguez pas de clarifications.

Cotation: un point par réponse correcte.

Les réponses correctes sont : en haut à gauche : 5 ; en haut à droite : 6; en bas à gauche : 10 ; en bas à droite : 7.

VISUOSPATIAL – Localisation de chiffres: SCORE 0-4

Consigne: Dites: 'Quel chiffre correspond à la position du point?' Aller de la gauche vers la droite et de haut en bas. Tous les dessins doivent être analysés. Si le patient ne comprend pas les instructions, expliquer « *Imaginez que cette case (montrer la case du bas) soit placée par-dessus celle du haut (montrer la case du haut), sur quel nombre sera positionné le point ?* »

Cotation: Un point par réponse correcte. **Les réponses correctes sont : en haut à gauche : 6 ; en haut à droite : 5; en bas à gauche : 2 ; en bas à droite : 3.**

EXÉCUTIF – Complétion de phrase: SCORE 0-12

Consigne: Dites: 'Écoutez attentivement ces phrases. Aussitôt que j'aurai fini de les lire, veuillez me dire, ou écrire, un mot qui finit la phrase aussi vite que possible. Par exemple: '*elle était si fatiguée qu'elle est allée directement au…lit*'. Ne pas donner de score.

Puis dire: 'Maintenant j'aimerais que l'on recommence, mais cette fois-ci j'aimerais que le mot que vous donnerez ne fasse aucun sens dans le contexte de la phrase. Il ne doit pas être relié au mot qui complète correctement la phrase. Par exemple, `*John s'est coupé la main avec…une orange*'. Si le patient donne un mot lié au contexte ou qui termine exactement la phrase, rappelez-lui la consigne. S'il donne un mot déjà cité précédemment, lui demander d'en trouver un autre. Poser toutes les questions, même si les réponses sont incorrectes. Si le patient ne répond pas dans les 20 secondes, passez à la phrase suivante. Si une réponse incorrecte est donnée, ne répéter les instructions que une fois (après la première erreur) durant l'administration du test. Si le participant répond immédiatement après la répétition des instructions avec un mot différent, ne côter que la réponse initiale avant le rappel et passer à la prochaine phrase. Si le participant répète un mot d'un essai précédent, côter comme incorrect et demander lui de ne pas répéter un mot pour plusieurs phrases.

Cotation: 2 en cas de mots différents, sans aucun rapport, 1 si le mot peut être lié (relié ou opposé) et 0 pour un mot correct (c'est à dire qui complète normalement la phrase). À noter : les phrases peuvent ne pas être correctes sur le plan grammatical. Ne pas pénaliser si le genre ou le nombre ne sont pas respectés.

Voir le tableau ci-dessous pour les exemples de cotation:

	**Question**	**2 points**	**1 point**	**0 points**
**1**	Le facteur a frappé à la	Voiture, pomme de terre, …	Fenêtre, portail, sac de courrier, …	porte
**2**	Il a apporté son parapluie avec lui en cas de	Parachute, caoutchouc, …	Soleil, vent, verglas, …	pluie
**3**	Sally prépare son toast avec du beurre et	Terre, sable, …	Céréales, oeuf, oranges, …	Confiture, marmelade, miel, fromage
**4**	John est allé chez le barbier pour se faire .... les cheveux	Lune, table, …	Laver, rallonger,	couper
**5**	Elle a plongé dans la	Jardin, balançoire, …	Marre, bain, …	piscine
**6**	Ils sont tous allés au café local pour .... quelque chose	Sauter, danser, …	Faire, jouer, acheter, ..	Manger, boire.

COGNITION SOCIALE – Partie A

Consigne: Sur la première page se trouvent six rectangles contenant chacun quatre dessins, un à chaque coin. Dites : 'Vous allez voir quelques photos, une dans chaque coin d'une boîte. Vous devez choisir quelle photo vous préférez. Vous pouvez pointer ou **dire quelle image vous préférez**. Veuillez répondre aussi vite que possible.'

Cotation: Les réponses sont notées mais non cotées. Les réponses seront utilisées pour la cotation du test suivant.

COGNITION SOCIALE – Partie B: SCORE 0-12

Consigne: Dites: 'Vous allez voir quelques photos, une dans chaque coin d'une boîte. Vous devez choisir quelle photo le visage préfère. Vous pouvez pointer ou dire quelle image il préfère. Veuillez répondre aussi vite que possible.' Proposer toutes les cases même en cas de réponses incorrectes. Si le patient ne comprend pas la consigne, ne pas l'indicer mais répéter les instructions.

Cotation: 2 points par réponse correcte, 1 point en cas de réponse incorrecte (dessin incorrectement identifié mais différent du choix donné lors du test précédent), 0 point si la réponse est celle donnée lors du test précédent.

SCORES

MÉMOIRE – Rappel retardé: SCORE 0-10

Consigne: Dites: 'Au début de cette entrevue, je vous ai lu une histoire courte. Dites-moi tout ce que vous pouvez vous rappeler sur cette histoire' Il n'y a pas de limite de temps. Le test est arrêté quand le patient dit ne pas se rappeler d'autres éléments. Des autocorrections sont acceptées, et seule la réponse finale sera prise pour la cotation.

Cotation: 1 point pour chaque section soulignée rappelée (en entier ou pour partie). Utiliser le même tableau de cotation que précédemment (Cf. rappel immédiat).

Le pourcentage de rétention peut être calculé :

Diviser le score obtenu au rappel différé par le score obtenu au rappel immédiat et multiplier par 100.

Par exemple : pour un score de rappel différé de 8/10 et un score de rappel immédiat de 9/10, le pourcentage de mémorisation est de 8/9*100 =89%.

Certains patients peuvent avoir un rappel différé supérieur au rappel immédiat avec un % de mémorisation >100%.

Utiliser la table de conversion insérée dans l'échelle ECAS pour obtenir ensuite le score de rétention. C'est le score de rétention qui est comptabilisé dans le score de mémoire final.

MÉMOIRE – Reconnaissance retardée: SCORE 0-4

Consigne: Ce test **ne sera pas** effectué si le patient a rappelé tous les items de l'histoire (score 10). Dans ce cas il obtiendra 4 points. Dans le cas contraire, dites: 'Voyons voir si vous pouvez vous rappeler de plus de détails sur cette histoire. Je vais vous poser quelques questions, répondez-moi par **oui** ou par **non**.'

Cotation: 1 point par réponse correcte (les réponses correctes sont indiquées en gras). Si le patient répond par **« je ne sais pas »**, demandez-lui de faire un choix entre vrai ou faux et coter en fonction de cette réponse. **Utiliser la table de conversion insérée dans l'échelle ECAS pour déterminer le score final.**

Echelle Cognitive et Comportementale D'Edimbourg – ECAS
Canadian French Version (2018)

Dépistage de comportement – Entretien avec aidant
Procédures de passation

Developed by S. Abrahams and T.H. Bak, University of Edinburgh
Adapted for use in North America by K. Rascovsky and M. Grossman, University of Pennsylvania

**Langage**	Appellation, Compréhension, Orthographe	/28
**Fluence verbale**	Aisance de langage Lettre S, Aisance de langage Lettre T	/24
**Executif**	Empan de chiffres inversé, Alternance, Complétion de phrase, Cognition sociale	/48
	**SLA-SPECIFIQUE:**	**/100**
Mémoire	*Rappel immédiat, Rappel retardé, Reconnaissance retardée*	/24
Visuospatial	*Compter les points, Comptage de cubes, Localisation de chiffres*	/12
	SLA NON-SPECIFIQUE:	***/36***
	**SCORE ECAS TOTAL:**	**/136**

<u>Procédure</u>:

Demander au soignant ou au conjoint de répondre au questionnaire, hors de la présence du patient, idéalement dans une pièce séparée. Il peut avoir noté des modifications dans toutes les dimensions proposées, dans seulement quelques-unes ou

aucun changement n'est rapporté. Demander au soignant, conjoint, de donner des exemples si possible. Ce questionnaire ne doit PAS être donné au soignant ou au conjoint directement, il fait forcément l'objet d'un entretien avec l'examinateur, qui pose les questions et rempli lui-même le questionnaire. Si un informateur n'est pas disponible merci d'utiliser une liste alternative du Comportement et de la psychose pour la collecte de données. Notez que cependant, que ces formulaires alternatifs ne peuvent pas générer de score ECAS et ont pour seul but la collecte de données.
Il y a cinq parties à cette entrevue.

Administration: SCORE 0-10
Interroger le soignant, le conjoint sur l'existence de comportements listés dans l'échelle. Ces comportements doivent être permanents ou répétés et pas seulement notés qu'une seule fois. Ils peuvent avoir précédé les signes moteurs. Cocher oui, non, ou ne sait pas. Si "oui" décrire brièvement le comportement. Donner un point pour chaque "oui" coché (maximum 10)

ECHELLE COGNITIVE ET COMPORTEMENTALE D'EDIMBOURG
ECAS Canadian French Version (2018)
Dépistages de psychose SLA

Questionnaire de l'aidant (entrevue de l'aidant)

Procédure:
Demander au soignant ou au conjoint de compléter cette évaluation, hors de la présence du patient, idéalement dans une pièce séparée. Rappeler au soignant ou au conjoint que ces comportements ne sont pertinents que depuis le début de la maladie. Ce questionnaire ne doit PAS être donné au soignant ou au conjoint directement, il fait forcément l'objet d'un entretien avec l'examinateur, qui pose les questions et rempli lui-même le questionnaire.

Administration: **SCORE 0-3**
Interroger le soignant, le conjoint sur l'existence de comportements listés dans l'échelle. Cocher oui, non, ou ne sait pas. Si "oui" décrire brièvement le comportement. Donner un point pour chaque "oui" coché (maximum 3)

ECHELLE COGNITIVE ET COMPORTEMENTALE D'EDIMBOURG DE DÉPISTAGE DE LA SLA
ECAS Formulaire B (Version Français Canada 2020)

Procédures de passation

Developed by S. Abrahams and T.H. Bak, University of Edinburgh
Adapted for use in North America by K. Rascovsky and M. Grossman, University of Pennsylvania

L'échelle ECAS est une échelle comportant un certain nombre des tests cognitifs qui se sont révélés sensibles pour la détection de troubles cognitifs dans la SLA. Cette échelle permet de contribuer à différencier différents profils cognitifs du sujet âgé, lors de la dépression, de la maladie d'Alzheimer ou de la démence fronto-temporale. Les fonctions exécutives, la mémoire, le langage, les capacités visuo-spatiales, la cognition sociale sont évalués ainsi que le comportement à l'aide d'un entretien avec l'aidant ou le conjoint. L'échelle ECAS a été spécialement élaborée pour les patients SLA. Les réponses pouvant être données oralement, ou par écrit et désignation. Cela est adapté au patient anarthrique ou présentant un déficit moteur. Le score total est de 136 points et cette évaluation est réalisée en 25 min environ (compter plus de temps si le test est réalisé par écrit).

Matériel nécessaire:
Pour faire passer l'ECAS, vous devez vous munir d'un chronomètre. Une calculatrice est recommandée (mais les calculs peuvent se faire sans). Les réponses peuvent être écrites ou données oralement, **la réponse orale est à privilégier** (si elle est possible). Les réponses données par écrit seront récupérées sur une feuille séparée. Il importe de se munir de stylos permettant de s'adapter au mieux aux possibilités du patient.

Informateur :
Un informateur externe est requis pour compléter le dépistage de Comportement et de Psychose. Ce peut être le proche aidant du patient, un proche ou un ami. Si un informateur n'est pas disponible merci d'utiliser la liste alternative du Comportement et de Psychose pour collecter des données sur le comportement et la psychose. Ces formulaires alternatifs ne peuvent pas être utilisés pour la génération de scores ECAS et ont pour unique but la collecte de données.

Démographie :
Occupation : indiquer le plus haut niveau occupé dans la vie – ne reflète pas nécessairement la position occupée la plus récente ou bien la retraite.
Main utilisée : indiquer Droit, Gauche, ou non dominant.
Années d'éducation : merci d'indiquer le total des années d'éducation complétées (en incluant toutes les formes d'éducation).
Diplôme complété le plus élevé : merci d'indiquer le niveau le plus élevé de diplôme complété. (ex : CEGEP, BSc, MSc, PhD…)
Difficulté de langue : merci de demander au participant « Avez-vous eu des difficultés de lectures ou d'apprentissage de l'écriture à l'école? » et répondre par Oui ou Non. Si le participant donne une réponse spécifique comme par exemple la dyslexie, merci de documenter la réponse.

LANGAGE - Appellation: SCORE 0-8

Consigne: Il y a huit dessins proposés. Demander au patient de nommer ces dessins. Aucune limite de temps n'est imposée pour cette tâche. Les réponses incorrectes sont notées et aucune proposition de correction n'est proposée. La réponse est correcte que si le mot exact est prononcé ou écrit. Des auto-corrections sont acceptées et seule la réponse finale sera prise en compte pour la cotation. Si la réponse donnée est correcte mais ne correspond pas à la réponse attendue comme par exemple 'chouette' pour 'hiboux', relancer en disant « connaissez-vous un autre nom pour cette image ? ». Si les réponses

correspondent à un mot moins fréquent mais juste, comme 'hachette' pour 'hache', coter la réponse comme correcte. Ne proposez pas au participant d'autre nom alternatif une fois qu'une réponse finale est donnée.

Cotation: Un point par réponse correcte donnée. Si les réponses sont produites par écrit, ne pas pénaliser les fautes d'orthographe tant que le bon mot est produit. **Les réponses correctes (et les alternatives acceptables) sont (collone gauche puis colonne droite et du haut vers le bas) :**

paon, tracteur, clef à molette, chouette, veste, zèbre, hérisson, tambourin.

LANGAGE - Compréhension: SCORE 0-8

Consigne: Avec la planche de dessins utilisée pour le test précédent, demander au patient d'indiquer en pointant ou bien en disant à l'oral la réponse correcte aux questions posées. Les phrases peuvent être lues par le patient ou par l'examinateur. Il y a plusieurs fois les mêmes réponses, le patient n'en est pas informé à l'avance mais cela peut lui être précisé s'il pose la question. En cas de non réponse, ne rien écrire sur l'espace dédié. Des autocorrections sont acceptées, et seule la réponse finale sera prise pour la cotation.

Cotation: Un point par réponse correcte donnée. **Les réponses correctes sont : Veste, paon, tambourin, tracteur, clef à molette, hérisson, tracteur, zèbre.**

Note : Si le participant ne nomme pas correctement un item dans la section « Appellation » mais qu'il identifie le bon item avec le mauvais nom dans la section « Compréhension », coter comme bon. Exemple : si le participant nomme incorrectement un item , « Scorpion », comme « Homard » dans la section Appellation, mais qu'il répond « Homard » quand on lui demande « Quelque chose avec un dard? », coter comme bon. Dans cet exemple, le participant ne connait pas le nom de l'item mais comprend de quoi il s'agit.

Cependant, si le participant nomme incorrectement un item dans la partie Appellation (ex : Scorpion nommé Homard) puis ensuite le nomme correctement dans la partie Compréhension : ne pas corriger rétrospectivement la réponse d'Appellation mais coter bon la section Compréhension.

MEMOIRE - Rappel immédiat: SCORE 0-10

Consigne: Dites: '*Je vais vous lire une histoire courte. Veuillez l'écouter attentivement. Lorsque j'ai fini, dites ou écrivez autant que vous le pouvez sur l'histoire*.'

Le rythme de lecture est de 2 mots par seconde. L'histoire doit être lue en 20 secondes en moyenne. A la fin de la lecture, dire au patient : *' l'histoire est terminée, de quoi vous rappelez-vous ?'*.

Il n'y a pas de limite de temps. Le test est arrêté quand le patient dit ne pas se rappeler d'autres éléments. Des autocorrections sont acceptées et seule la réponse finale sera prise pour la cotation.

Cotation: un point pour chaque mot ou groupe de mots soulignés rappelés (en entier ou une partie).. Voir ci-dessous les réponses alternatives acceptables. Les nombres doivent être rappelés correctement, par ex : *32 jeunes baleines* rappelé comme *trente et quelque chose*, est incorrect,et sera cotée 0. Cf. tableau ci-dessous. Le score de rappel immédiat obtenu servira aussi plus tard pour le calcul du pourcentage de ***mémoire différée.*** Ne pas donner d'indices lors du rappel, mais s'assurer que le patient a fini de restituer l'histoire en lui demandant « est-ce tout ce dont vous pouvez vous souvenir ? ».

ECAS 2: coter 1 point pour chaque section soulignée retenue (entière ou partielle)

Section	Réponses acceptables
Trois	Seul le nombre exact 3 est accepté
Bateaux de pêche	« Bateaux » et/ou « de pêche ». Si le participant identifie correctement un des sens de cet item, côter comme bon. Ex : « pêcheurs » et « bateaux de plaisance » sont acceptés
Aidé à sauver	« aidé », « sauvé », « sauver » sont acceptés
Baleine	Seulement « baleines » ou « baleines » sont acceptés
Rivage	« Rivage » ou des réponses avec des sens similaires sont acceptés. Ex : « prêt de la plage », « juste à côté de la côte » sont acceptés
Cercles	Les synonymes proches ou les réponses au sens similaires sont acceptés. Ex : « les baleines faisaient des ronds »
Alan Williams	Sont acceptés « Alan » et/ou « Williams »
Société de conservation marine	Côter comme bon n'importe quelle part de « société de conservation marine ». Accepter les items sémantiquement similaires comme : animal, vie sauvage, nature, goupe, société, agence, conseil…
32	Seul le nombre exact 32 est accepté
L'hiver dernier	Sont seulement acceptés : « l'hiver dernier », « cet hiver », « l'année dernière » ou «l'année précédente ». « L,été dernier » ou « cette année » ne sont pas acceptés

LANGAGE – Orthographe: SCORE 0-12

Consigne: Dites: 'Veuillez épeler à haute voix sur papier les mots suivants.' Si la personne utilise une technologie d'assistance, demandez-lui d'éteindre toute fonction prédictive.

Il n'y a pas de limite de temps. Tous les mots doivent être *évalués*, même en cas d'erreurs sur les premiers mots. Passer au mot suivant si le patient est incapable ou refuse de répondre. En cas de difficulté lors de l'épellation orale, autoriser le patient à lire en même temps le mot écrit par l'examinateur. Si nécessaire, le mot peut être énoncé clairement plusieurs fois par l'évaluateur. Par exemple, si le participant épelle « Construisant » au lieu de « Construction », répéter clairement le mot. De la même manière, si le pluriel d'un mot est épelé, répéter clairement le mot.

Instructions particulières : Il peut être nécessaire de préciser pour les items 8 et 12 les mots dans un contexte de phrase pour différencier « enregistré » de « enregistrer » et « apporté » de « apporter ».
Exemple : J'ai enregistré un album / J'ai apporté du pain.

Cotation: Un point pour chaque mot correctement épelé ou écrit. Des autocorrections sont acceptées, et seule la réponse finale sera prise pour la cotation. Ne pas pénaliser si le participant insert un espace dans l'épellation des mots (ex : Tourne Vis au lieu de Tournevis). L'existence d'un QI bas prémorbide ou d'antécédent de difficultés de lecture ou d'orthographe doivent être recherchés, pris en compte dans l'interprétation des résultats et notifiés. Se rappeler de demander au participant et/ou à l'informateur s' il y a existence de difficultés de langage ou d'histoire prémorbide, comme indiqué en haut de chaque formulaire ECAS.

FLUENCE VERBALE - Lettre F: Score 0-12

Consigne: Le patient peut réaliser ce test soit oralement soit par écrit. Dites: 'Je vais vous donner une lettre de l'alphabet et j'aimerais que vous disiez ou écriviez autant de mots que vous le pouvez qui commencent par cette lettre. **Les noms propres ou les chiffres ne sont pas acceptés.'**

- Si parlé, dites: 'Vous avez une minute. La lettre est F.'
- Si écrit, dites: 'Vous avez deux minutes. La lettre est F.'

Ensuite, la personne copie ou lit les mots à haute voix.

Les mots seront écrits (par le patient ou l'évaluateur) en colonne sur une feuille préparée avec deux colonnes pour que la copie (à droite) s'appuie sur la production (à gauche). Ne pas enlever les répétitions, les intrusions et les erreurs. Il est recommandé à l'évaluateur de copier les mots en majuscules pour que la lecture par le patient soit possible.

Les réponses incorrectes seront éliminées et ne devront pas être ni lues (épreuve orale) ni recopiées (épreuve écrite) par le patient.

- Si parlé, dites: 'Lisez ces mots à haute voix aussi vite que possible. Avant de faire cela, vérifiez que vous pouvez les lire. Je vais vous chronométrer. Prêt? Commencez.'
- Si écrit, dites: 'Copiez ces mots aussi vite que possible. Je vais vous chronométrer. Prêt? Commencez.'

Cotation: Toutes les réponses fournies sont enregistrées mais les règles suivantes seront appliquées pour déterminer une réponse correcte :

- les mots doivent être diversifiés

- sont cotés comme incorrect:

  - les mots absurdes (sans signification), les répétitions, les noms propres
  - les mots dérivés (ex : moustache/ moustachu), les persévérations sur un mot comme par exemple un verbe conjugué (à l'infinitif, au participe passé…) alors que le sens n'est pas modifié

- sont cotés comme correct:

  - les homographes si un sens différent est précisé par le patient (ex : un *moule* l'objet et une *moule* l'animal)
  - les homophones en cas de test réalisé oralement si la différence d'orthographe et/ ou de sens est indiquée par le patient
  - un changement de sens quand celui-ci est indiqué (exemple : *montrer* le verbe et *montre* l'objet, si cela est spécifié)
  - les différences grammaticales commes entre adverbes et verbes/noms – exemple : « final » et « finalement », côter comme deux items différents
  - les mots au pluriel s'ils n'ont pas été donnés précédemment au singulier (ex : « papiers » est accepté si « papier » n'a pas été donné avant. Si le participant dit « papier », puis « papiers » : ne côter comme bon que le premier mot.

Si les réponses sont produites par écrit, ne pas pénaliser les fautes d'orthographe tant que le bon mot est produit.
Exemple de cotation : Merise, menuisier, meuble, *(menuiserie),* maçon, *(maçonnerie),* matériel, manger, *(mangé),* magistrat, *(magistrature),* magique, maison, myrtille, *(magicien),* mûre, mayonnaise, *(maisonnette)*." score = 12
Afin de tenir compte des différences entre les deux formes de test, un Index de Fluence Verbale (**i FV : verbal fluency index)** est calculé selon l'équation suivante :

Calcul de l'index de fluence verbale **i FV** :

$$\text{iFV} = \frac{\text{temps du test (en secondes)} - \text{temps de répétition du test (en secondes)}}{\text{Nombre de mots corrects}}$$

Par exemple : un patient qui donne 5 mots corrects en 60 secondes et les relit en 15 secondes, aura un iFV de 9 (60-15/5=9).

Puis cet index (iFV) sera converti en score de fluence en suivant la table de conversion présentée dans l'échelle.

EXÉCUTIF – Empan de chiffres inverse : Score 0-12

<u>Consigne</u>: Les chiffres doivent être lus avec un rythme de 1 par seconde. Dites: 'Je vais vous donner une liste de chiffres et j'aimerais que vous me les répétiez dans l'ordre inversé. Par exemple, si je dis 2-3-4, vous devriez dire 4-3-2. Essayons avec 7-1-9, que me diriez-vous?'
Si le patient ne réussit pas cet essai, un autre essai sera effectué avec seulement deux chiffres, avant de démarrer le test. Si le patient ne réussit pas cet essai à 2 chiffres, le test est coté à 0/12 et aucune autre partie du test n'est tentée. Le patient sera informé à chaque fois qu'il y aura augmentation du nombre de chiffres (à chaque nouvelle ligne). Pour qu'un essai soit coté comme correct, le patient doit répéter *tous* les chiffres en sens inverse. Sera cotée comme incorrecte la répétition de tous les chiffres mais selon une mauvaise séquence. Si le patient réussit au moins un essai sur les deux présentés par ligne, passer à la ligne suivante. Des autocorrections sont acceptées, et seule la réponse finale sera prise pour la cotation. Arrêter après deux échecs consécutifs sur une même ligne. Le patient peut réaliser ce test soit oralement soit par écrit, mais il est recommandé d'essayer de le réaliser oralement. A l'écrit, ne pas autoriser à écrire d'abord les chiffres dans l'ordre ni de les noter de droite à gauche.
<u>Cotation</u>: Le score correspond au nombre total d'essais correctement effectués.

EXÉCUTIF – Alternance: Score 0-12

<u>Consigne:</u> Dites: 'J'aimerais que vous alterniez entre chiffres et lettres, en commençant par 1A, puis 2B, 3C, et ainsi de suite. Veuillez alterner entre chiffres et lettres, dans l'ordre, sans en sauter, jusqu'à ce que je vous dise d'arrêter. Commençons ensemble : 1A, 2B, 3C…' Après avoir commencé avec le participant, laissez-le continuer la séquence tout seul. Si le participant alterne avec la lettre en premier et le chiffre en deuxième (ex : D4, E5,…), poursuivre l'administration du test car l'ordre séquentiel est préservé. Arrêter l'administration après que le patient ai fait une erreur.
<u>Cotation:</u> Un point par essai correct. Côter comme bon même si le participant initie ses réponses par la lettre et non par le chiffre. Pour la version écrite, Ne pas autoriser les stratégies (écrire tous les chiffres puis ensuite les lettres, si le sujet le fait lui dire d'arrêter).

FLUENCE VERBALE - lettre **D**: SCORE 0-12

Consigne: le patient peut réaliser ce test soit oralement soit par écrit. Dites: 'Je vais vous donner une lettre de l'alphabet et j'aimerais que vous disiez ou écriviez autant de mots que vous le pouvez qui commencent par cette lettre. **Les noms propres ou les chiffres ne sont pas acceptés**. **Maintenant, le mot doit être long de quatre lettres. Ni plus, ni moins, que quatre lettres.** A noter que le pluriel du mot est accepté pour faire un mot de 4 lettres.

- Si oral, dites: 'Vous avez une minute. La lettre est D.'
- Si écrit, dites: 'Vous avez deux minutes. La lettre est D.'

Ensuite la personne copie ou lit les mots à haute voix.

- Si parlé, dites: 'Lisez ces mots à haute voix aussi vite que possible. Avant de faire cela, vérifiez que vous pouvez les lire. Je vais vous chronométrer. Prêt? Commencez.'
- Si écrit, dites: 'Copiez ces mots aussi vite que possible. Je vais vous chronométrer. Prêt? Commencez..'

Cotation: suivre les mêmes consignes que celles précédemment appliquées pour le précédent test de fluence (lettre S) pour calculer l'iVF et se référer à la table de conversion présenté dans l'échelle. Les pluriels sont acceptés pour créer des mots de 4 lettres. Exemple : « rats » si la lettre était « R ». Les contractions peuvent également être acceptées.

VISUOSPATIAL – Comptage de points: SCORE 0-4

Consigne: Dites: 'J'aimerais que vous comptiez le nombre de points dans chaque boîte, mais sans les pointer.' Aller de la gauche vers la droite et du haut en bas. Tous les carrés doivent être analysés.
Cotation: un point par réponse correcte.
Les réponses correctes sont : en haut à gauche : 10 ; en haut à droite : 8 ; en bas à gauche : 7 ; en bas à droite : 9.

VISUOSPATIAL – Comptage de cubes: SCORE 0-4

Consigne: Dites: 'Combien de cubes y a-t-il dans chaque structure, incluant ceux que vous n'êtes pas capable de voir?' Aller de la gauche vers la droite et de haut en bas. Tous les dessins doivent être analysés. Si le participant ne comprend pas, répéter seulement les instructions et ne prodiguez pas de clarifications.
Cotation: un point par réponse correcte.
Les réponses correctes sont : en haut à gauche : 5 ; en haut à droite : 6; en bas à gauche : 10 ; en bas à droite : 7.

VISUOSPATIAL – Localisation de chiffres: SCORE 0-4

Consigne: Dites: 'Quel chiffre correspond à la position du point?' Aller de la gauche vers la droite et de haut en bas. Tous les dessins doivent être analysés. Si le patient ne comprend pas les instructions, expliquer « *Imaginez que cette case (montrer la case du bas) soit placée par-dessus celle du haut (montrer la case du haut), sur quel nombre sera positionné le point ?* »

Cotation: Un point par réponse correcte. **Les réponses correctes sont : en haut à gauche : 7 ; en haut à droite : 8; en bas à gauche : 4 ; en bas à droite : 3.**

EXÉCUTIF – Complétion de phrase: SCORE 0-12

Consigne: Dites: 'Écoutez attentivement ces phrases. Aussitôt que j'aurai fini de les lire, veuillez me dire, ou écrire, un mot qui finit la phrase aussi vite que possible. Par exemple: '*elle était si fatiguée qu'elle est allée directement au…lit*'. Ne pas donner de score.

Puis dire: 'Maintenant j'aimerais que l'on recommence, mais cette fois-ci j'aimerais que le mot que vous donnerez ne fasse aucun sens dans le contexte de la phrase. Il ne doit pas être relié au mot qui complète correctement la phrase. Par exemple, `*John s'est coupé la main avec…une orange*'. Si le patient donne un mot lié au contexte ou qui termine exactement la phrase, rappelez-lui la consigne. S'il donne un mot déjà cité précédemment, lui demander d'en trouver un autre. Poser toutes les questions, même si les réponses sont incorrectes. Si le patient ne répond pas dans les 20 secondes, passez à la phrase suivante. Si une réponse incorrecte est donnée, ne répéter les instructions que une fois (après la première erreur) durant l'administration du test. Si le participant répond immédiatement après la répétition des instructions avec un mot différent, ne côter que la réponse initiale avant le rappel et passer à la prochaine phrase. Si le participant répète un mot d'un essai précédent, côter comme incorrect et demander lui de ne pas répéter un mot pour plusieurs phrases.

Cotation: 2 en cas de mots différents, sans aucun rapport, 1 si le mot peut être lié (relié ou opposé) et 0 pour un mot correct (c'est à dire qui complète normalement la phrase). À noter : les phrases peuvent ne pas être correctes sur le plan grammatical. Ne pas pénaliser si le genre ou le nombre ne sont pas respectés.

Voir le tableau ci-dessous pour les exemples de cotation:

	**Question**	**2 points**	**1 point**	**0 points**
**1**	Elle a répondu au téléphone parce qu'il…	était mauve, dangereux, ensoleillé,.	était silencieux, il parlait,….	sonnait
**2**	La blague était tellement drôle qu'il a commencé à….	se déshabiller, marcher, disparaître	pleurer, renifler, s'endormir,…	rire, glousser, rigoler,….
**3**	Daniel a débarré la porte avec …	un balon, un melon,…	un marteau, un couteau à papier,…	une clef
**4**	L'enfant coupe du papier avec une paire de….	chaussures, de lunettes, de bananes,…	mains,…	ciseaux
**5**	Après des mois de pratique, Lisa à passé son ….. de conduire	spectacle, nuit,…	essai,…	test, permis
**6**	Simon a mangé son diner avec un couteau et…	nez, papier, crayon,….	une salade, une pelle, un cure-dent,…	Une fourchette, une cuillère,….

COGNITION SOCIALE – Partie A

Consigne: Sur la première page se trouvent six rectangles contenant chacun quatre dessins, un à chaque coin. Dites : 'Vous allez voir quelques photos, une dans chaque coin d'une boîte. Vous devez choisir quelle photo vous préférez. Vous pouvez pointer ou **dire quelle image vous préférez**. Veuillez répondre aussi vite que possible.'

Cotation: Les réponses sont notées mais non cotées. Les réponses seront utilisées pour la cotation du test suivant.

COGNITION SOCIALE – Partie B: SCORE 0-12

Consigne: Dites: 'Vous allez voir quelques photos, une dans chaque coin d'une boîte. Vous devez choisir quelle photo le visage préfère. Vous pouvez pointer ou dire quelle image il préfère. Veuillez répondre aussi vite que possible.' Proposer toutes les cases même en cas de réponses incorrectes. Si le patient ne comprend pas la consigne, ne pas l'indicer mais répéter les instructions.

Cotation: 2 points par réponse correcte, 1 point en cas de réponse incorrecte (dessin incorrectement identifié mais différent [illegible]

SCORES

MÉMOIRE – Rappel retardé: SCORE 0-10

Consigne: Dites: 'Au début de cette entrevue, je vous ai lu une histoire courte. Dites-moi tout ce que vous pouvez vous rappeler sur cette histoire' Il n'y a pas de limite de temps. Le test est arrêté quand le patient dit ne pas se rappeler d'autres éléments. Des autocorrections sont acceptées, et seule la réponse finale sera prise pour la cotation.

Cotation: 1 point pour chaque section soulignée rappelée (en entier ou pour partie). Utiliser le même tableau de cotation que précédemment (Cf. rappel immédiat).

Le pourcentage de rétention peut être calculé :

Diviser le score obtenu au rappel différé par le score obtenu au rappel immédiat et multiplier par 100.

Par exemple : pour un score de rappel différé de 8/10 et un score de rappel immédiat de 9/10, le pourcentage de mémorisation est de 8/9*100 =89%.

Certains patients peuvent avoir un rappel différé supérieur au rappel immédiat avec un % de mémorisation >100%.

Utiliser la table de conversion insérée dans l'échelle ECAS pour obtenir ensuite le score de rétention. C'est le score de rétention qui est comptabilisé dans le score de mémoire final.

MÉMOIRE – Reconnaissance retardée: SCORE 0-4

Consigne: Ce test **ne sera pas** effectué si le patient a rappelé tous les items de l'histoire (score 10). Dans ce cas il obtiendra 4 points. Dans le cas contraire, dites: 'Voyons voir si vous pouvez vous rappeler de plus de détails sur cette histoire. Je vais vous poser quelques questions, répondez-moi par **oui** ou par **non**.'

Cotation: 1 point par réponse correcte (les réponses correctes sont indiquées en gras). Si le patient répond par **« je ne sais pas »**, demandez-lui de faire un choix entre vrai ou faux et coter en fonction de cette réponse. **Utiliser la table de conversion insérée dans l'échelle ECAS pour déterminer le score final.**

Echelle Cognitive et Comportementale D'Edimbourg – ECAS
Canadian French Version (2018)

Dépistage de comportement – Entretien avec aidant
Procédures de passation

Developed by S. Abrahams and T.H. Bak, University of Edinburgh
Adapted for use in North America by K. Rascovsky and M. Grossman, University of Pennsylvania

**Langage**	Appellation, Compréhension, Orthographe	/28
**Fluence verbale**	Aisance de langage Lettre S, Aisance de langage Lettre T	/24
**Executif**	Empan de chiffres inversé, Alternance, Complétion de phrase, Cognition sociale	/48
	**SLA-SPECIFIQUE:**	**/100**
Mémoire	*Rappel immédiat, Rappel retardé, Reconnaissance retardée*	/24
Visuospatial	*Compter les points, Comptage de cubes, Localisation de chiffres*	/12
	SLA NON-SPECIFIQUE:	***/36***
	**SCORE ECAS TOTAL:**	**/136**

<u>Procédure</u>:

Demander au soignant ou au conjoint de répondre au questionnaire, hors de la présence du patient, idéalement dans une pièce séparée. Il peut avoir noté des modifications dans toutes les dimensions proposées, dans seulement quelques-unes ou

aucun changement n'est rapporté. Demander au soignant, conjoint, de donner des exemples si possible. Ce questionnaire ne doit PAS être donné au soignant ou au conjoint directement, il fait forcément l'objet d'un entretien avec l'examinateur, qui pose les questions et rempli lui-même le questionnaire. Si un informateur n'est pas disponible merci d'utiliser une liste alternative du Comportement et de la psychose pour la collecte de données. Notez que cependant, que ces formulaires alternatifs ne peuvent pas générer de score ECAS et ont pour seul but la collecte de données.
Il y a cinq parties à cette entrevue.

Administration: SCORE 0-10
Interroger le soignant, le conjoint sur l'existence de comportements listés dans l'échelle. Ces comportements doivent être permanents ou répétés et pas seulement notés qu'une seule fois. Ils peuvent avoir précédé les signes moteurs. Cocher oui, non, ou ne sait pas. Si "oui" décrire brièvement le comportement. Donner un point pour chaque "oui" coché (maximum 10)

ECHELLE COGNITIVE ET COMPORTEMENTALE D'EDIMBOURG
ECAS Canadian French Version (2018)
Dépistages de psychose SLA

Questionnaire de l'aidant (entrevue de l'aidant)

Procédure:
Demander au soignant ou au conjoint de compléter cette évaluation, hors de la présence du patient, idéalement dans une pièce séparée. Rappeler au soignant ou au conjoint que ces comportements ne sont pertinents que depuis le début de la maladie. Ce questionnaire ne doit PAS être donné au soignant ou au conjoint directement, il fait forcément l'objet d'un entretien avec l'examinateur, qui pose les questions et rempli lui-même le questionnaire.

Administration: **SCORE 0-3**
Interroger le soignant, le conjoint sur l'existence de comportements listés dans l'échelle. Cocher oui, non, ou ne sait pas. Si "oui" décrire brièvement le comportement. Donner un point pour chaque "oui" coché (maximum 3)

ECHELLE COGNITIVE ET COMPORTEMENTALE D'EDIMBOURG DE DÉPISTAGE DE LA SLA
ECAS Formulaire C (Version Français Canada 2020)

Procédures de passation

Developed by S. Abrahams and T.H. Bak, University of Edinburgh
Adapted for use in North America by K. Rascovsky and M. Grossman, University of Pennsylvania

L'échelle ECAS est une échelle comportant un certain nombre des tests cognitifs qui se sont révélés sensibles pour la détection de troubles cognitifs dans la SLA. Cette échelle permet de contribuer à différencier différents profils cognitifs du sujet âgé, lors de la dépression, de la maladie d'Alzheimer ou de la démence fronto-temporale. Les fonctions exécutives, la mémoire, le langage, les capacités visuo-spatiales, la cognition sociale sont évalués ainsi que le comportement à l'aide d'un entretien avec l'aidant ou le conjoint. L'échelle ECAS a été spécialement élaborée pour les patients SLA. Les réponses pouvant être données oralement, ou par écrit et désignation. Cela est adapté au patient anarthrique ou présentant un déficit moteur. Le score total est de 136 points et cette évaluation est réalisée en 25 min environ (compter plus de temps si le test est réalisé par écrit).

Matériel nécessaire:
Pour faire passer l'ECAS, vous devez vous munir d'un chronomètre. Une calculatrice est recommandée (mais les calculs peuvent se faire sans). Les réponses peuvent être écrites ou données oralement, **la réponse orale est à privilégier** (si elle est possible). Les réponses données par écrit seront récupérées sur une feuille séparée. Il importe de se munir de stylos permettant de s'adapter au mieux aux possibilités du patient.

Informateur :
Un informateur externe est requis pour compléter le dépistage de Comportement et de Psychose. Ce peut être le proche aidant du patient, un proche ou un ami. Si un informateur n'est pas disponible merci d'utiliser la liste alternative du Comportement et de Psychose pour collecter des données sur le comportement et la psychose. Ces formulaires alternatifs ne peuvent pas être utilisés pour la génération de scores ECAS et ont pour unique but la collecte de données.

Démographie :
Occupation : indiquer le plus haut niveau occupé dans la vie – ne reflète pas nécessairement la position occupée la plus récente ou bien la retraite.
Main utilisée : indiquer Droit, Gauche, ou non dominant.
Années d'éducation : merci d'indiquer le total des années d'éducation complétées (en incluant toutes les formes d'éducation).
Diplôme complété le plus élevé : merci d'indiquer le niveau le plus élevé de diplôme complété. (ex : CEGEP, BSc, MSc, PhD…)
Difficulté de langue : merci de demander au participant « Avez-vous eu des difficultés de lectures ou d'apprentissage de l'écriture à l'école? » et répondre par Oui ou Non. Si le participant donne une réponse spécifique comme par exemple la dyslexie, merci de documenter la réponse.

LANGAGE - Appellation: SCORE 0-8

Consigne: Il y a huit dessins proposés. Demander au patient de nommer ces dessins. Aucune limite de temps n'est imposée pour cette tâche. Les réponses incorrectes sont notées et aucune proposition de correction n'est proposée. La réponse est correcte que si le mot exact est prononcé ou écrit. Des auto-corrections sont acceptées et seule la réponse finale sera prise en compte pour la cotation. Si la réponse donnée est correcte mais ne correspond pas à la réponse attendue comme par exemple 'pieuvre' pour 'poulpe', relancer en disant « connaissez-vous un autre nom pour cette image ? ». Si les réponses correspondent

à un mot moins fréquent mais juste, comme 'hachette' pour 'hache', coter la réponse comme correcte. Ne proposez pas au participant d'autre nom alternatif une fois qu'une réponse finale est donnée.

Cotation: Un point par réponse correcte donnée. Si les réponses sont produites par écrit, ne pas pénaliser les fautes d'orthographe tant que le bon mot est produit. **Les réponses correctes (et les alternatives acceptables) sont (de gauche à droite et du haut vers le bas) : coccinelle, bracelet, caravane, papillon, scie, pieuvre, perroquet, saxophone.**

LANGAGE - Compréhension: SCORE 0-8

Consigne: Avec la planche de dessins utilisée pour le test précédent, demander au patient d'indiquer en pointant ou bien en disant à l'oral la réponse correcte aux questions posées. Les phrases peuvent être lues par le patient ou par l'examinateur. Il y a plusieurs fois les mêmes réponses, le patient n'en est pas informé à l'avance mais cela peut lui être précisé s'il pose la question. En cas de non réponse, ne rien écrire sur l'espace dédié. Des autocorrections sont acceptées, et seule la réponse finale sera prise pour la cotation.

Cotation: Un point par réponse correcte donnée. **Les réponses correctes sont : papillon, scie, perroquet, caravane, bracelet, saxophone, scie, pieuvre.**

Note : Si le participant ne nomme pas correctement un item dans la section « Appellation » mais qu'il identifie le bon item avec le mauvais nom dans la section « Compréhension », coter comme bon. Exemple : si le participant nomme incorrectement un item , « Scorpion », comme « Homard » dans la section Appellation, mais qu'il répond « Homard » quand on lui demande « Quelque chose avec un dard? », coter comme bon. Dans cet exemple, le participant ne connait pas le nom de l'item mais comprend de quoi il s'agit.

Cependant, si le participant nomme incorrectement un item dans la partie Appellation (ex : Scorpion nommé Homard) puis ensuite le nomme correctement dans la partie Compréhension : ne pas corriger rétrospectivement la réponse d'Appellation mais coter bon la section Compréhension.

MEMOIRE - Rappel immédiat: SCORE 0-10

Consigne: Dites: '*Je vais vous lire une histoire courte. Veuillez l'écouter attentivement. Lorsque j'ai fini, dites ou écrivez autant que vous le pouvez sur l'histoire.*'

Le rythme de lecture est de 2 mots par seconde. L'histoire doit être lue en 20 secondes en moyenne. A la fin de la lecture, dire au patient : *' l'histoire est terminée, de quoi vous rappelez-vous ?'*.

Il n'y a pas de limite de temps. Le test est arrêté quand le patient dit ne pas se rappeler d'autres éléments. Des autocorrections sont acceptées et seule la réponse finale sera prise pour la cotation.

Cotation: un point pour chaque mot ou groupe de mots soulignés rappelés (en entier ou une partie).. Voir ci-dessous les réponses alternatives acceptables. Les nombres doivent être rappelés correctement, par ex : *700 compétiteurs* rappelé comme *plusieurs centaines et quelque chose*, est incorrect,et sera cotée 0. Cf. tableau ci-dessous. Le score de rappel immédiat obtenu servira aussi plus tard pour le calcul du pourcentage de ***mémoire différée.*** Ne pas donner d'indices lors du rappel, mais s'assurer que le patient a fini de restituer l'histoire en lui demandant « est-ce tout ce dont vous pouvez vous souvenir ? ».

ECAS 3: coter 1 point pour chaque section soulignée retenue (entière ou partielle)

Section	Réponses acceptables
Helen Blake	« Hélène » et/ou « Blake » sont acceptés
Boston	Seul « Boston est accepté
Nord	Seuls « Nord » ou « du Nord » sont acceptés
Prix	Sont acceptés « Prix » ou des alternative sémantiquement similaire comme « récompense », « compétition ». « Art n'est pas accepté »
Photographie	Sont acceptés « photographie », « photos », « photographe »
47	Seul le chiffre exact 47 est accepté
Randonnée	« marcher dans la campagne » est accepté mais simplement « marcher » ne l'est pas
700	Seul le chiffre exact 700 est accepté
Chêne	Doit mentionner « chêne » pour être accepté. « arbre » ou « forêt » ne sont pas acceptés
Couleurs d'automne	Sont seulement acceptés « couleurs d'automne », « feuilles d'automne », « automnal » ou « automne ».

LANGAGE – Orthographe: SCORE 0-12

Consigne: Dites: 'Veuillez épeler à haute voix sur papier les mots suivants.' Si la personne utilise une technologie d'assistance, demandez-lui d'éteindre toute fonction prédictive.

Il n'y a pas de limite de temps. Tous les mots doivent être *évalués*, même en cas d'erreurs sur les premiers mots. Passer au mot suivant si le patient est incapable ou refuse de répondre. En cas de difficulté lors de l'épellation orale, autoriser le patient à lire en même temps le mot écrit par l'examinateur. Si nécessaire, le mot peut être énoncé clairement plusieurs fois par l'évaluateur. Par exemple, si le participant épelle « Construisant » au lieu de « Construction », répéter clairement le mot. De la même manière, si le pluriel d'un mot est épelé, répéter clairement le mot.

Instructions particulières : Il peut être nécessaire de préciser pour les items 8 et 12 les mots dans un contexte de phrase pour différencier « enregistré » de « enregistrer » et « apporté » de « apporter ».
Exemple : J'ai enregistré un album / J'ai apporté du pain.

Cotation: Un point pour chaque mot correctement épelé ou écrit. Des autocorrections sont acceptées, et seule la réponse finale sera prise pour la cotation. Ne pas pénaliser si le participant insert un espace dans l'épellation des mots (ex : Tourne Vis au lieu de Tournevis). L'existence d'un QI bas prémorbide ou d'antécédent de difficultés de lecture ou d'orthographe doivent être recherchés, pris en compte dans l'interprétation des résultats et notifiés. Se rappeler de demander au participant et/ou à

l'informateur s' il y a existence de difficultés de langage ou d'histoire prémorbide, comme indiqué en haut de chaque formulaire ECAS.

FLUENCE VERBALE - Lettre P: Score 0-12

Consigne: Le patient peut réaliser ce test soit oralement soit par écrit. Dites: 'Je vais vous donner une lettre de l'alphabet et j'aimerais que vous disiez ou écriviez autant de mots que vous le pouvez qui commencent par cette lettre. **Les noms propres ou les chiffres ne sont pas acceptés.'**

- Si parlé, dites: 'Vous avez une minute. La lettre est P.'
- Si écrit, dites: 'Vous avez deux minutes. La lettre est P.'

Ensuite, la personne copie ou lit les mots à haute voix.

Les mots seront écrits (par le patient ou l'évaluateur) en colonne sur une feuille préparée avec deux colonnes pour que la copie (à droite) s'appuie sur la production (à gauche). Ne pas enlever les répétitions, les intrusions et les erreurs. Il est recommandé à l'évaluateur de copier les mots en majuscules pour que la lecture par le patient soit possible.

Les réponses incorrectes seront éliminées et ne devront pas être ni lues (épreuve orale) ni recopiées (épreuve écrite) par le patient.

- Si parlé, dites: 'Lisez ces mots à haute voix aussi vite que possible. Avant de faire cela, vérifiez que vous pouvez les lire. Je vais vous chronométrer. Prêt? Commencez.'
- Si écrit, dites: 'Copiez ces mots aussi vite que possible. Je vais vous chronométrer. Prêt? Commencez.'

Cotation: Toutes les réponses fournies sont enregistrées mais les règles suivantes seront appliquées pour déterminer une réponse correcte :

- les mots doivent être diversifiés

- sont cotés comme incorrect:

- les mots absurdes (sans signification), les répétitions, les noms propres
- les mots dérivés (ex : moustache/ moustachu), les persévérations sur un mot comme par exemple un verbe conjugué (à l'infinitif, au participe passé…) alors que le sens n'est pas modifié

- sont cotés comme correct:

- les homographes si un sens différent est précisé par le patient (ex : un *moule* l'objet et une *moule* l'animal)
- les homophones en cas de test réalisé oralement si la différence d'orthographe et/ ou de sens est indiquée par le patient
- un changement de sens quand celui-ci est indiqué (exemple : *montrer* le verbe et *montre* l'objet, si cela est spécifié)
- les différences grammaticales commes entre adverbes et verbes/noms – exemple : « final » et « finalement », côter comme deux items différents
- les mots au pluriel s'ils n'ont pas été donnés précédemment au singulier (ex : « papiers » est accepté si « papier » n'a pas été donné avant. Si le participant dit « papier », puis « papiers » : ne côter comme bon que le premier mot.

Si les réponses sont produites par écrit, ne pas pénaliser les fautes d'orthographe tant que le bon mot est produit.

Exemple de cotation : Merise, menuisier, meuble, *(menuiserie),* maçon, *(maçonnerie),* matériel, manger, *(mangé),* magistrat, *(magistrature),* magique, maison, myrtille, *(magicien),* mûre, mayonnaise, *(maisonnette)*." score = 12

Afin de tenir compte des différences entre les deux formes de test, un Index de Fluence Verbale (**i FV : verbal fluency index)** est calculé selon l'équation suivante :

Calcul de l'index de fluence verbale **i FV** :

iFV = temps du test (en secondes) – temps de répétition du test (en secondes)
Nombre de mots corrects

Par exemple : un patient qui donne 5 mots corrects en 60 secondes et les relit en 15 secondes, aura un iFV de 9 (60-15/5=9).

Puis cet index (iFV) sera converti en score de fluence en suivant la table de conversion présentée dans l'échelle.

EXÉCUTIF – Empan de chiffres inverse : Score 0-12

Consigne: Les chiffres doivent être lus avec un rythme de 1 par seconde. Dites: 'Je vais vous donner une liste de chiffres et j'aimerais que vous me les répétiez dans l'ordre inversé. Par exemple, si je dis 2-3-4, vous devriez dire 4-3-2. Essayons avec 7-1-9, que me diriez-vous?'

Si le patient ne réussit pas cet essai, un autre essai sera effectué avec seulement deux chiffres, avant de démarrer le test. Si le patient ne réussit pas cet essai à 2 chiffres, le test est coté à 0/12 et aucune autre partie du test n'est tentée. Le patient sera informé à chaque fois qu'il y aura augmentation du nombre de chiffres (à chaque nouvelle ligne). Pour qu'un essai soit coté comme correct, le patient doit répéter *tous* les chiffres en sens inverse. Sera cotée comme incorrecte la répétition de tous les chiffres mais selon une mauvaise séquence. Si le patient réussit au moins un essai sur les deux présentés par ligne, passer à la ligne suivante. Des autocorrections sont acceptées, et seule la réponse finale sera prise pour la cotation. Arrêter après deux échecs consécutifs sur une même ligne. Le patient peut réaliser ce test soit oralement soit par écrit, mais il est recommandé d'essayer de le réaliser oralement. A l'écrit, ne pas autoriser à écrire d'abord les chiffres dans l'ordre ni de les noter de droite à gauche.

Cotation: Le score correspond au nombre total d'essais correctement effectués.

EXÉCUTIF – Alternance: Score 0-12

Consigne: Dites: 'J'aimerais que vous alterniez entre chiffres et lettres, en commençant par 1A, puis 2B, 3C, et ainsi de suite. Veuillez alterner entre chiffres et lettres, dans l'ordre, sans en sauter, jusqu'à ce que je vous dise d'arrêter. Commençons ensemble : 1A, 2B, 3C…' Après avoir commencé avec le participant, laissez-le continuer la séquence tout seul. Si le participant alterne avec la lettre en premier et le chiffre en deuxième (ex : D4, E5,…), poursuivre l'administration du test car l'ordre séquentiel est préservé. Arrêter l'administration après que le patient ai fait une erreur.

Cotation: Un point par essai correct. Côter comme bon même si le participant initie ses réponses par la lettre et non par le chiffre. Pour la version écrite, Ne pas autoriser les stratégies (écrire tous les chiffres puis ensuite les lettres, si le sujet le fait lui dire d'arrêter).

FLUENCE VERBALE - lettre M: SCORE 0-12

Consigne: le patient peut réaliser ce test soit oralement soit par écrit. Dites: 'Je vais vous donner une lettre de l'alphabet et j'aimerais que vous disiez ou écriviez autant de mots que vous le pouvez qui commencent par cette lettre. **Les noms propres ou les chiffres ne sont pas acceptés**. **Maintenant, le mot doit être long de quatre lettres. Ni plus, ni moins, que quatre lettres.** A noter que le pluriel du mot est accepté pour faire un mot de 4 lettres.

- Si oral, dites: 'Vous avez une minute. La lettre est M.'
- Si écrit, dites: 'Vous avez deux minutes. La lettre est M.'

Ensuite la personne copie ou lit les mots à haute voix.

- Si parlé, dites: 'Lisez ces mots à haute voix aussi vite que possible. Avant de faire cela, vérifiez que vous pouvez les lire. Je vais vous chronométrer. Prêt? Commencez.'
- Si écrit, dites: 'Copiez ces mots aussi vite que possible. Je vais vous chronométrer. Prêt? Commencez..'

Cotation: suivre les mêmes consignes que celles précédemment appliquées pour le précédent test de fluence (lettre S) pour calculer l'iVF et se référer à la table de conversion présenté dans l'échelle. Les pluriels sont acceptés pour créer des mots de 4 lettres. Exemple : « rats » si la lettre était « R ». Les contractions peuvent également être acceptées.

VISUOSPATIAL – Comptage de points: SCORE 0-4

Consigne: Dites: 'J'aimerais que vous comptiez le nombre de points dans chaque boîte, mais sans les pointer.' Aller de la gauche vers la droite et du haut en bas. Tous les carrés doivent être analysés.

Cotation: un point par réponse correcte.

Les réponses correctes sont : en haut à gauche : 10 ; en haut à droite : 8 ; en bas à gauche : 7 ; en bas à droite : 9.

VISUOSPATIAL – Comptage de cubes: SCORE 0-4

Consigne: Dites: 'Combien de cubes y a-t-il dans chaque structure, incluant ceux que vous n'êtes pas capable de voir?' Aller de la gauche vers la droite et de haut en bas. Tous les dessins doivent être analysés. Si le participant ne comprend pas, répéter seulement les instructions et ne prodiguez pas de clarifications.

Cotation: un point par réponse correcte.

Les réponses correctes sont : en haut à gauche : 5 ; en haut à droite : 6; en bas à gauche : 8 ; en bas à droite : 7.

VISUOSPATIAL – Localisation de chiffres: SCORE 0-4

Consigne: Dites: 'Quel chiffre correspond à la position du point?' Aller de la gauche vers la droite et de haut en bas. Tous les dessins doivent être analysés. Si le patient ne comprend pas les instructions, expliquer « *Imaginez que cette case (montrer la case du bas) soit placée par-dessus celle du haut (montrer la case du haut), sur quel nombre sera positionné le point ?* »

Cotation: Un point par réponse correcte. **Les réponses correctes sont : en haut à gauche : 1 ; en haut à droite : 8; en bas à gauche : 5 ; en bas à droite : 7.**

EXÉCUTIF – Complétion de phrase: SCORE 0-12

Consigne: Dites: 'Écoutez attentivement ces phrases. Aussitôt que j'aurai fini de les lire, veuillez me dire, ou écrire, un mot qui finit la phrase aussi vite que possible. Par exemple: *'elle était si fatiguée qu'elle est allée directement au…lit'*. Ne pas donner de score.

Puis dire: 'Maintenant j'aimerais que l'on recommence, mais cette fois-ci j'aimerais que le mot que vous donnerez ne fasse aucun sens dans le contexte de la phrase. Il ne doit pas être relié au mot qui complète correctement la phrase. Par exemple, *`John s'est coupé la main avec…une orange'*. Si le patient donne un mot lié au contexte ou qui termine exactement la phrase, rappelez-lui la consigne. S'il donne un mot déjà cité précédemment, lui demander d'en trouver un autre. Poser toutes les questions, même si les réponses sont incorrectes. Si le patient ne répond pas dans les 20 secondes, passez à la phrase suivante. Si une réponse incorrecte est donnée, ne répéter les instructions que une fois (après la première erreur) durant l'administration du test. Si le participant répond immédiatement après la répétition des instructions avec un mot différent, ne côter que la réponse initiale avant le rappel et passer à la prochaine phrase. Si le participant répète un mot d'un essai précédent, côter comme incorrect et demander lui de ne pas répéter un mot pour plusieurs phrases.

Cotation: 2 en cas de mots différents, sans aucun rapport, 1 si le mot peut être lié (relié ou opposé) et 0 pour un mot correct (c'est à dire qui complète normalement la phrase). À noter : les phrases peuvent ne pas être correctes sur le plan grammatical. Ne pas pénaliser si le genre ou le nombre ne sont pas respectés.

Voir le tableau ci-dessous pour les exemples de cotation:

	**Question**	**2 points**	**1 point**	**0 points**
**1**	Lisa est allé à la bibliothèque rendre quelques…	vers, pommes, parapluies,….	vidéos, DVD,…	livres, magazines, ouvrages,….
**2**	Après sa douche, elle s'est séchée avec une …	du rouge à lèvres, une banane,…	de l'eau, une serpillère,…	une serviette, un drap,….
**3**	Il a mis un sachet de thé dans sa tasse et à fait bouillir….	du pétrol, des spaghetti, du jus d'orange,…	du lait,….	de l'eau, sa bouilloire,…
**4**	Il a étudié en médecine pour devenir un…	plombier, ingénieur, une carotte,…	chercheur, infimirmier,…	médecin, docteur, psychiatre, ….
**5**	La musique a débuté et tout le monde s'est levé pour…	se brosser les dents, voler, pleurer,…	s'asseoir, dormir, crier, parler, bailler…	dancer, boire, rentrer à la maison,….
**6**	John a ramassé la laisse et a pris son chien pour une…	une classe de step, une incinération/crémation, un vol…	baignade, une partie de football,…	marche, une balade, une promenade,…

COGNITION SOCIALE – Partie A

Consigne: Sur la première page se trouvent six rectangles contenant chacun quatre dessins, un à chaque coin. Dites : 'Vous allez voir quelques photos, une dans chaque coin d'une boîte. Vous devez choisir quelle photo vous préférez. Vous pouvez pointer ou **dire quelle image vous préférez**. Veuillez répondre aussi vite que possible.'

Cotation: Les réponses sont notées mais non cotées. Les réponses seront utilisées pour la cotation du test suivant.

COGNITION SOCIALE – Partie B: SCORE 0-12

Consigne: Dites: 'Vous allez voir quelques photos, une dans chaque coin d'une boîte. Vous devez choisir quelle photo le visage préfère. Vous pouvez pointer ou dire quelle image il préfère. Veuillez répondre aussi vite que possible.' Proposer toutes les cases même en cas de réponses incorrectes. Si le patient ne comprend pas la consigne, ne pas l'indicer mais répéter les instructions.

Cotation: 2 points par réponse correcte, 1 point en cas de réponse incorrecte (dessin incorrectement identifié mais différent du choix donné lors du test précédent), 0 point si la réponse est celle donnée lors du test précédent.

SCORES

MÉMOIRE – Rappel retardé: SCORE 0-10

Consigne: Dites: 'Au début de cette entrevue, je vous ai lu une histoire courte. Dites-moi tout ce que vous pouvez vous rappeler sur cette histoire' Il n'y a pas de limite de temps. Le test est arrêté quand le patient dit ne pas se rappeler d'autres éléments. Des autocorrections sont acceptées, et seule la réponse finale sera prise pour la cotation.

Cotation: 1 point pour chaque section soulignée rappelée (en entier ou pour partie). Utiliser le même tableau de cotation que précédemment (Cf. rappel immédiat).

Le pourcentage de rétention peut être calculé :

Diviser le score obtenu au rappel différé par le score obtenu au rappel immédiat et multiplier par 100.

Par exemple : pour un score de rappel différé de 8/10 et un score de rappel immédiat de 9/10, le pourcentage de mémorisation est de 8/9*100 =89%.

Certains patients peuvent avoir un rappel différé supérieur au rappel immédiat avec un % de mémorisation >100%.

Utiliser la table de conversion insérée dans l'échelle ECAS pour obtenir ensuite le score de rétention. C'est le score de rétention qui est comptabilisé dans le score de mémoire final.

MÉMOIRE – Reconnaissance retardée: SCORE 0-4

Consigne: Ce test **ne sera pas** effectué si le patient a rappelé tous les items de l'histoire (score 10). Dans ce cas il obtiendra 4 points. Dans le cas contraire, dites: 'Voyons voir si vous pouvez vous rappeler de plus de détails sur cette histoire. Je vais vous poser quelques questions, répondez-moi par **oui** ou par **non**.'

Cotation: 1 point par réponse correcte (les réponses correctes sont indiquées en gras). Si le patient répond par **« je ne sais pas »**, demandez-lui de faire un choix entre vrai ou faux et coter en fonction de cette réponse. **Utiliser la table de conversion insérée dans l'échelle ECAS pour déterminer le score final.**

Echelle Cognitive et Comportementale D'Edimbourg – ECAS
Canadian French Version (2018)

Dépistage de comportement – Entretien avec aidant
Procédures de passation

Developed by S. Abrahams and T.H. Bak, University of Edinburgh
Adapted for use in North America by K. Rascovsky and M. Grossman, University of Pennsylvania

**Langage**	Appellation, Compréhension, Orthographe	/28
**Fluence verbale**	Aisance de langage Lettre S, Aisance de langage Lettre T	/24
**Executif**	Empan de chiffres inversé, Alternance, Complétion de phrase, Cognition sociale	/48
	**SLA-SPECIFIQUE:**	**/100**
Mémoire	*Rappel immédiat, Rappel retardé, Reconnaissance retardée*	/24
Visuospatial	*Compter les points, Comptage de cubes, Localisation de chiffres*	/12
	SLA NON-SPECIFIQUE:	**/36**
	**SCORE ECAS TOTAL:**	**/136**

Procédure:

Demander au soignant ou au conjoint de répondre au questionnaire, hors de la présence du patient, idéalement dans une pièce séparée. Il peut avoir noté des modifications dans toutes les dimensions proposées, dans seulement quelques-unes ou

aucun changement n’est rapporté. Demander au soignant, conjoint, de donner des exemples si possible. Ce questionnaire ne doit PAS être donné au soignant ou au conjoint directement, il fait forcément l’objet d’un entretien avec l’examinateur, qui pose les questions et rempli lui-même le questionnaire. Si un informateur n’est pas disponible merci d’utiliser une liste alternative du Comportement et de la psychose pour la collecte de données. Notez que cependant, que ces formulaires alternatifs ne peuvent pas générer de score ECAS et ont pour seul but la collecte de données.
Il y a cinq parties à cette entrevue.

Administration: SCORE 0-10
Interroger le soignant, le conjoint sur l'existence de comportements listés dans l’échelle. Ces comportements doivent être permanents ou répétés et pas seulement notés qu’une seule fois. Ils peuvent avoir précédé les signes moteurs. Cocher oui, non, ou ne sait pas. Si "oui" décrire brièvement le comportement. Donner un point pour chaque "oui" coché (maximum 10)

ECHELLE COGNITIVE ET COMPORTEMENTALE D’EDIMBOURG
ECAS Canadian French Version (2018)
Dépistages de psychose SLA

Questionnaire de l'aidant (entrevue de l’aidant)

Procédure:
Demander au soignant ou au conjoint de compléter cette évaluation, hors de la présence du patient, idéalement dans une pièce séparée. Rappeler au soignant ou au conjoint que ces comportements ne sont pertinents que depuis le début de la maladie. Ce questionnaire ne doit PAS être donné au soignant ou au conjoint directement, il fait forcément l’objet d’un entretien avec l’examinateur, qui pose les questions et rempli lui-même le questionnaire.

Administration: **SCORE 0-3**
Interroger le soignant, le conjoint sur l'existence de comportements listés dans l’échelle. Cocher oui, non, ou ne sait pas. Si "oui" décrire brièvement le comportement. Donner un point pour chaque "oui" coché (maximum 3)

Appendix 9.3: Sematic Fluency and Abrahams Correction

Appendix 9.3.1: Semantic Fluency [ENGLISH]
Appendix 9.3.2: Semantic Fluency [FRENCH]
Appendix 9.3.3: Abrahams Correction [ENGLISH]
Appendix 9.3.4: Abrahams Correction [FRENCH]

Appendix 9.3.1: Semantic Fluency [ENGLISH]
Found in [Semantic Fluency (EN, 28Oct2013) - CAPTURE ALS]

SEMANTIC FLUENCY TEST

Participant ID:______________________________ Age:_________

Date:_______/_______/_______ Examiner:___________________________

Instructions to the subject following the Verbal Fluency Test: *"Tell me all the ANIMALS / FRUIT / VEGETABLES you can think of as quickly as you can, and it doesn't matter what letter of the alphabet the name begins with. Go."*

Say "Fine" or "Good" after each 1-minute performance. If subjects discontinue before the end of the minute, encourage them to try to think of more words. If there is a silence for 15 seconds, repeat the basic instructions, and the test category. Mark an "R" in space if instructions were repeated, and "---"every 15 seconds if possible.

	**ANIMALS. (60)**		**FRUIT. (60)**		**VEGETABLES. (60)**
1.		1.		1.	
2		2		2	
3.		3.		3.	
4.		4.		4.	
5.		5.		5.	
6.		6.		6.	
7.		7.		7.	
8.		8.		8.	
9.		9.		9.	
10.		10.		10.	
11.		11.		11.	
12.		12.		12.	
13.		13.		13.	
14.		14.		14.	
15.		15.		15.	
16.		16.		16.	
17.		17.		17.	
18.		18.		18.	
19.		19.		19.	
20.		20.		20.	
21.		21.		21.	
22.		22.		22.	
23.		23.		23.	
24.		24.		24.	
25.		25.		25.	
	Total Correct:________		Total Correct:________		Total Correct:________

Appendix 9.3.2: Semantic Fluency [FRENCH]
Found in [Semantic Fluency (FR, 09Sep2013) - CAPTURE ALS]

TEST DE FLUENCE SÉMANTIQUE

ID participant:______________________________ Âge:_________

Date:_______/_______/_______ Examinateur (-trice): ___________________________

Instructions pour le sujet, à suivre le test de fluence verbale*: « Dis-moi tous les ANIMAUX / FRUITS / LÉGUMES dont vous pouvez penser, aussi rapidement que possible. Les mots ce que vous dites peuvent commencer avec n'importe lettre de l'alphabet. Allez. »*

Dis "d'accord" ou "bon" après chaque test qui durera 1 minute. Si les sujets arrêtent avant la fin de la minute, encouragez-les de penser d'autres mots. S'il y a un silence pour 15 secondes, répétez les instructions, et la catégorie du test. Marquez un « R » au-dessus du tableau si les instructions ont du être répété, et « --- » chaque 15 secondes si possible.

	**ANIMAUX. (60)**		**FRUITS. (60)**		**LÉGUMES. (60)**
1.		1.		1.	
2		2		2	
3.		3.		3.	
4.		4.		4.	
5.		5.		5.	
6.		6.		6.	
7.		7.		7.	
8.		8.		8.	
9.		9.		9.	
10.		10.		10.	
11.		11.		11.	
12.		12.		12.	
13.		13.		13.	
14.		14.		14.	
15.		15.		15.	
16.		16.		16.	
17.		17.		17.	
18.		18.		18.	
19.		19.		19.	
20.		20.		20.	
21.		21.		21.	
22.		22.		22.	
23.		23.		23.	
24.		24.		24.	
25.		25.		25.	
	Total Correct:________		Total Correct:________		Total Correct:________

Appendix 9.3.3: Abrahams Correction [ENGLISH]

Found in [Abrahams Correction (EN, 13Oct2016) - CAPTURE ALS]

CAPTURE-ALS<br>Abrahams Correction<br>*Following Verbal or Semantic Fluency*	PARTICIPANT ID:	
	DATE:	
	VISIT:	

1. Record whether Fluency was done by writing or speaking. Spoken is timed for 1 minute; Written is timed for 2 minutes.
2. Record number of *correct* words generated.
3. If the subject SPOKE the words, copy their responses legibly. Ensure they are able to read it before proceeding. Say: ***'read aloud these words as fast as possible. Before you do this, check that you can read them. I will time you. Ready? Begin.'*** Time how long it takes to read this list.

   If the subject WROTE the words, they will read their words out loud. ***Say: 'copy these words as fast as possible. I will time you. Ready? Begin.'*** Time how long it takes to write out the list.
4. Record:

   TIME to read/ copy aloud: _____________ ☐ spoken ☐ written

   NUMBER of correct words: _____________

1.	21.
2.	22.
3.	23.
4.	24.
5.	25.
6.	26.
7.	27.
8.	28.
9.	29.
10.	30.
11.	31.
12.	32.
13.	33.
14.	34.
15.	35.
16.	36.
17.	37.
18.	38.
19.	39.
20.	40.

Appendix 9.3.4: Abrahams Correction [FRENCH]

Found in [Abrahams Correction (FR, 13Oct2016) - CAPTURE ALS]

CAPTURE-ALS

Abrahams Correction

Following Verbal or Semantic Fluency

PARTICIPANT ID:

DATE:

VISIT:

1. Indiquez ci-dessous si le Test de Fluence a été conduit oralement ou par écrit. Chronométrez le test oral pour 1 minute; chronométrez le test écrit pour 2 minutes.

2. Inscrivez combien de mots ont été *correctement* généré.

3. Pour le test ORALE, copiez les réponses du sujet de façon lisible, ou fournissez-les avec une rédaction écrite. Dis : « **À haute voix, lisez ces mots aussi rapidement que possible. Avant de commencer, vérifiez si vous êtes capable de lire tous les mots. Je vais vous chronométrer. À vos marques, prêts, allez !** » Chronométrez combien de temps il faut le sujet pour lire la liste.

   Pour le test ÉCRIT, demande le sujet de lire ses réponses à haute voix. Dis : « **À haute voix, répétez ces mots aussi rapidement que possible. Je vais vous chronométrer. À vos marques, prêts, allez !** » Chronométrez combien de temps il faut le sujet pour lire la liste.

4. Notez:

   -durée de lire à haute voix ou copier des mots: __________ ☐ parlée ☐ écrit

   -nombres de mots corrects: __________

1.	21.
2.	22.
3.	23.
4.	24.
5.	25.
6.	26.
7.	27.
8.	28.
9.	29.
10.	30.
11.	31.
12.	32.
13.	33.
14.	34.
15.	35.
16.	36.
17.	37.
18.	38.
19.	39.
20.	40.

Appendix 9.4: Boston Naming Test-II (BNT-II)

Appendix 9.4.1: BNT-II Short & Standard Forms [ENGLISH]
Appendix 9.4.2: BNT-II Short & Standard Form [FRENCH]
Appendix 9.4.3: BNT-II Stimulus picture example

Appendix 9.4.1: BNT-II Short & Standard Forms [ENGLISH]
Found in [BNT-II Short & Standard Forms (EN, 28Jun2021) - CAPTURE ALS]

Boston Naming Test

RECORD BOOKLET

SECOND EDITION

Edith Kaplan, PhD
Harold Goodglass, PhD
Sandra Weintraub, PhD

ERROR CODES

The following error codes are used to categorize incorrect responses. The error code is to be entered in the designated column below.

**ph**	Nonword phonemically based paraphasia
**ph/v**	Real word phonemically based paraphasia (A phonemic paraphasia is scored if more than half of the target word phonology is preserved).
**v**	Verbal paraphasia, semantically related to the target word
**v/u**	Unrelated verbal paraphasia
**n**	Neologism (less than 50% overlap with the phonology of the target word)
**mw**	Multi-word paraphasic/paragrammatic error
**otu**	Other off-target utterance (not considered paraphasia)
**cl**	Circumlocution utterance (not considered paraphasia)
**p**	Perseveration
**perc**	Perceptual

SHORT FORM

Item	Response	Correct Response	Latency Seconds	Stimulus Cue	Phonemic Cue	Error Code(s)	Multiple Choice
1.	house *(home)* . . . . (a kind of building)						
2.	comb . . . . (used for fixing hair)						
3.	toothbrush. . . . . (used in the mouth)						
4.	octopus. . . . . (an ocean animal)						
5.	bench. . . . . (used for sitting)						
6.	volcano. . . . . (a kind of mountain)						
7.	canoe . . . . (used in the water)						
8.	beaver . . . . (an animal)						

Item	Response	Correct Response	Latency Seconds	Stimulus Cue	Phonemic Cue	Error Code(s)	Multiple Choice
9.	cactus *(saguara)* (something that grows)	____	____	____	____	____	____
10.	hammock (you lie on it)	____	____	____	____	____	____
11.	stethoscope (used by doctors and nurses)	____	____	____	____	____	____
12.	unicorn (mythical animal)	____	____	____	____	____	____
13.	tripod (photographers or surveyors use it)	____	____	____	____	____	____
14.	sphinx (it's found in Egypt)	____	____	____	____	____	____
15.	palette (artists use it)	____	____	____	____	____	____

Turn to page 9 for scoring.

STANDARD FORM

Item	Response	Correct Response	Latency Seconds	Stimulus Cue	Phonemic Cue	Error Code(s)	Multiple Choice
1.	bed . . . . (a piece of furniture)	____	____	____	____	____	____
2.	tree . . . . (something that grows outdoors)	____	____	____	____	____	____
3.	pencil . . . . (used for writing)	____	____	____	____	____	____
4.	house *(home)* . . . . (a kind of building)	____	____	____	____	____	____
5.	whistle . . . . (used for blowing)	____	____	____	____	____	____
6.	scissors *(shears)* . . . . . . (used for cutting)	____	____	____	____	____	____
7.	comb . . . . (used for fixing hair)	____	____	____	____	____	____
8.	flower. . . . . (grows in a garden)	____	____	____	____	____	____
9.	saw . . . . (used by a carpenter)	____	____	____	____	____	____
10.	toothbrush . . . . (used in the mouth)	____	____	____	____	____	____
11.	helicopter. . . . . (used for air travel)	____	____	____	____	____	____
12.	broom. . . . . (used for cleaning)	____	____	____	____	____	____

Item	Response	Correct Response	Latency Seconds	Stimulus Cue	Phonemic Cue	Error Code(s)	Multiple Choice
13.	octopus (an ocean animal)						
14.	mushroom *(toadstool)* (something to eat)						
15.	hanger (found in a closet)						
16.	wheelchair (found in a hospital)						
17.	camel *(dromedary)* (an animal)						
18.	mask *(false face)* (part of a costume)						
19.	pretzel (something to eat)						
20.	bench (used for sitting)						
21.	racquet (used for sports)						
22.	snail (an animal)						
23.	volcano (a kind of mountain)						
24.	seahorse *(horsefish)* (an ocean animal)						
25.	dart (you throw it)						

Item	Response	Correct Response	Latency Seconds	Stimulus Cue	Phonemic Cue	Error Code(s)	Multiple Choice
26.	canoe (used in the water)	___	___	___	___	___	___
27.	globe (a kind of map)	___	___	___	___	___	___
28.	wreath (a Christmas decoration)	___	___	___	___	___	___
29.	beaver (an animal)	___	___	___	___	___	___
30.	harmonica *(harp; mouth organ)* (musical instrument)	___	___	___	___	___	___
31.	rhinoceros (an animal)	___	___	___	___	___	___
32.	acorn (it comes from a tree)	___	___	___	___	___	___
33.	igloo (type of house)	___	___	___	___	___	___
34.	stilts (used to make you taller)	___	___	___	___	___	___
35.	dominoes (a game)	___	___	___	___	___	___
36.	cactus *(saguaro)* (something that grows)	___	___	___	___	___	___
37.	escalator (you go up on it)	___	___	___	___	___	___
38.	harp (a musical instrument)	___	___	___	___	___	___

Item	Response	Correct Response	Latency Seconds	Stimulus Cue	Phonemic Cue	Error Code(s)	Multiple Choice
39.	hammock (you lie on it)	____	____	____	____	____	____
40.	knocker (it's on a door)	____	____	____	____	____	____
41.	pelican (a bird)	____	____	____	____	____	____
42.	stethoscope (used by doctors and nurses)	____	____	____	____	____	____
43.	pyramid (found in Egypt)	____	____	____	____	____	____
44.	muzzle (used on dogs)	____	____	____	____	____	____
45.	unicorn (mythical animal)	____	____	____	____	____	____
46.	funnel (used for pouring)	____	____	____	____	____	____
47.	accordion (a musical instrument)	____	____	____	____	____	____
48.	noose (used for hanging)	____	____	____	____	____	____
49.	asparagus (something to eat)	____	____	____	____	____	____
50.	compass (for drawing)	____	____	____	____	____	____
51.	latch *(bolt)* (part of a door)	____	____	____	____	____	____

Item	Response	Correct Response	Latency Seconds	Stimulus Cue	Phonemic Cue	Error Code(s)	Multiple Choice
52.	tripod (photographers or surveyors use it)						
53.	scroll (a document)						
54.	tongs (a utensil)						
55.	sphinx (it's found in Egypt)						
56.	yoke *(oxbow)* (used on farm animals)						
57.	trellis (used in a garden)						
58.	palette (artists use it)						
59.	protractor (measures angles)						
60.	abacus (it's used for counting)						

Appendix 9.4.2: BNT-II Short & Standard Form [FRENCH]

Found in [BNT-II Short Form (FR, 01Aug2014) - CAPTURE ALS] and [BNT-II Standard Form (FR, 01Aug2014) - CAPTURE ALS]

CAPTURE-ALS
BOSTON NAMING TEST – second edition (BNT-II)
(French version)

PARTICIPANT ID:	
DATE:	
VISIT:	

Short Form

Item #	Word (English)	Word (French) (synonym)	Correct Response	Incorrect Response
1.	house	maison	☐ **1**	☐ **0**
2.	comb	peigne	☐ **1**	☐ **0**
3.	toothbrush	brosse à dents	☐ **1**	☐ **0**
4.	octopus	pieuvre	☐ **1**	☐ **0**
5.	bench	banc	☐ **1**	☐ **0**
6.	volcano	volcan	☐ **1**	☐ **0**
7.	canoe	canot (canoe)	☐ **1**	☐ **0**
8.	beaver	castor	☐ **1**	☐ **0**
9.	cactus	cactus	☐ **1**	☐ **0**
10.	hammock	hamac	☐ **1**	☐ **0**
11.	stethoscope	stéthoscope	☐ **1**	☐ **0**
12.	unicorn	licorne (unicorne)	☐ **1**	☐ **0**
13.	tripod	trépied	☐ **1**	☐ **0**
14.	sphinx	sphinx	☐ **1**	☐ **0**
15.	palette	palette (planche à peintre; planche pour peintre; planche à peinture; planche à toile)	☐ **1**	☐ **0**

TOTAL score = ☐

CAPTURE-ALS
BOSTON NAMING TEST – second edition (BNT-II)
(French version)

PARTICIPANT ID:

DATE:

VISIT:

Standard Form

Item #	Word (English)	Word (French) (synonym)	Correct Response	Incorrect Response
1.	bed	lit	☐ **1**	☐ **0**
2.	tree	arbre	☐ **1**	☐ **0**
3.	pencil	crayon	☐ **1**	☐ **0**
4.	house	maison	☐ **1**	☐ **0**
5.	whistle	sifflet	☐ **1**	☐ **0**
6.	scissors	ciseaux (paire de ciseaux)	☐ **1**	☐ **0**
7.	comb	peigne	☐ **1**	☐ **0**
8.	flower	fleur	☐ **1**	☐ **0**
9.	saw	scie	☐ **1**	☐ **0**
10.	toothbrush	brose à dents	☐ **1**	☐ **0**
11.	helicopter	hélicoptère	☐ **1**	☐ **0**
12.	broom	balai	☐ **1**	☐ **0**
13.	octopus	pieuvre	☐ **1**	☐ **0**
14.	mushroom	champignon	☐ **1**	☐ **0**
15.	hanger	cintre (support)	☐ **1**	☐ **0**
16.	wheelchair	chaise roulante (fauteuil roulant)	☐ **1**	☐ **0**
17.	camel	chameau	☐ **1**	☐ **0**
18.	mask	masque	☐ **1**	☐ **0**
19.	pretzel	bretzel (pretzel)	☐ **1**	☐ **0**
20.	bench	banc	☐ **1**	☐ **0**
21.	racquet	raquette	☐ **1**	☐ **0**
22.	snail	escargot (calimaçon)	☐ **1**	☐ **0**
23.	volcano	volcan	☐ **1**	☐ **0**
24.	seahorse	hippocampe (cheval de mer)	☐ **1**	☐ **0**
25.	dart	fléchette (dard)	☐ **1**	☐ **0**
26.	canoe	canot (canoe)	☐ **1**	☐ **0**
27.	globe	globe terrestre	☐ **1**	☐ **0**
28.	wreath	couronne	☐ **1**	☐ **0**
29.	beaver	castor	☐ **1**	☐ **0**

Item #	Word (English)	Word (French) (synonym)	Correct Response	Incorrect Response
30.	harmonica	harmonica	☐ **1**	☐ **0**
31.	rhinoceros	rhinocéros	☐ **1**	☐ **0**
32.	acorn	gland	☐ **1**	☐ **0**
33.	igloo	igloo	☐ **1**	☐ **0**
34.	stilts	échasses	☐ **1**	☐ **0**
35.	dominoes	dominoes	☐ **1**	☐ **0**
36.	cactus	catus	☐ **1**	☐ **0**
37.	escalator	escalier roulant	☐ **1**	☐ **0**
38.	harp	harpe	☐ **1**	☐ **0**
39.	hammock	hamac	☐ **1**	☐ **0**
40.	knocker	marteau (heurtoir)	☐ **1**	☐ **0**
41.	pelican	pélican	☐ **1**	☐ **0**
42.	stethoscope	stethoscope	☐ **1**	☐ **0**
43.	pyramid	pyramide	☐ **1**	☐ **0**
44.	muzzle	muselière	☐ **1**	☐ **0**
45.	unicorn	licorne (unicorne)	☐ **1**	☐ **0**
46.	funnel	entonnoir	☐ **1**	☐ **0**
47.	accordion	accordéon	☐ **1**	☐ **0**
48.	noose	corde de portence (nœud coolant)	☐ **1**	☐ **0**
49.	asparagus	asperge	☐ **1**	☐ **0**
50.	compass	compas	☐ **1**	☐ **0**
51.	latch	loquet	☐ **1**	☐ **0**
52.	tripod	trépied	☐ **1**	☐ **0**
53.	scroll	manuscript	☐ **1**	☐ **0**
54.	tongs	pincettes (pinces)	☐ **1**	☐ **0**
55.	sphinx	sphynx	☐ **1**	☐ **0**
56.	yoke	joug	☐ **1**	☐ **0**
57.	trellis	trellis	☐ **1**	☐ **0**
58.	palette	palette	☐ **1**	☐ **0**
59.	protractor	rapporteur d'angles	☐ **1**	☐ **0**
60.	abacus	boulier (boulier compteur)	☐ **1**	☐ **0**

TOTAL score = ☐

Appendix 9.4.3: BNT-II Stimulus picture example

Found in [BNT-II Stimulus picture example (28Jun2021) - CAPTURE ALS]

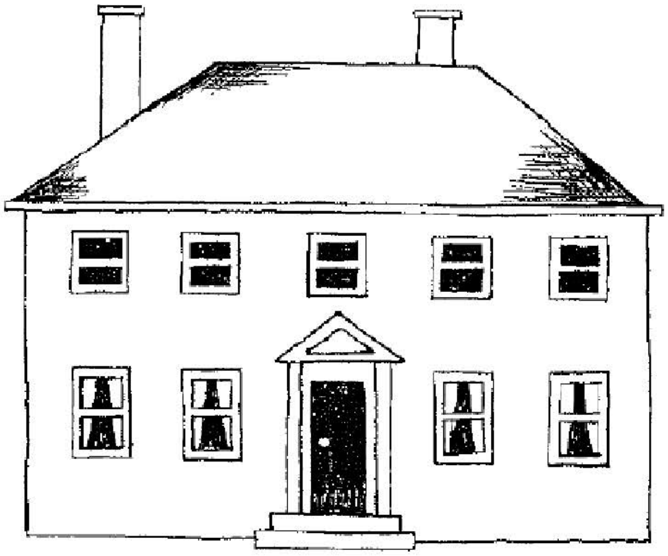

Appendix 9.5: Hopkins Verbal Learning Test – Revised (HVLT-R)

Appendix 9.5.1: HVLT-R (Form 1) [ENGLISH]
Appendix 9.5.2: HVLT-R (Form 2) [ENGLISH]
Appendix 9.5.3: HVLT-R (Form 1) [FRENCH]
Appendix 9.5.4: HVLT-R (Form 2) [FRENCH]

Appendix 9.5.1: HVLT-R (Form 1) [ENGLISH]
Found in [HVLT-R (Form 1) (EN, 09Sep2013) - CAPTURE ALS]

CAPTURE-ALS
Hopkins Verbal Learning Test – Revised (HVLT-R)
FORM 1

PARTICIPANT ID:
DATE:
VISIT:

Word List	Learning Trials			Delayed Recall Trial (20-25 mins)
	Trial 1	Trial 2	Trial 3	Trial 4
**LION**				
**EMERALD**				
**HORSE**				
**TENT**				
**SAPPHIRE**				
**HOTEL**				
**CAVE**				
**OPAL**				
**TIGER**				
**PEARL**				
**COW**				
**HUT**				
**Total correct responses =**				

Completion Time Trial 3 _________ **Start Time Trial 4** __________

Adapted and reproduced by special permission of the Publisher, Psychological Assessment Resources, Inc., 16204 North Florida Avenue, Lutz, Florida 33549, from the Hopkins Verbal Learning Test – Revised by Jason Brandt, PhD and Ralph H.B. Benedict, PhD, Copyright 1991, 1998, 2001 by PAR, Inc. Further reproduction is prohibited without permission from PAR, Inc.
Version 09-Sep-13

CAPTURE-ALS
Hopkins Verbal Learning Test – Revised (HVLT-R)
FORM 1

PARTICIPANT ID:	
DATE:	
VISIT:	

Delayed Recognition Instructions

The Delayed Recognition (Forced Choice) trial is administered immediately after the Delayed Recall trial. Say the following:

> **Now I am going to read a longer list of words to you. some of them are words from the original list, and some are not. After I read each word, I'd like you to say "Yes" if it was on the original list, or "No" if it was not.**

Read the words or the Delayed Recognition trial list in numerical order. Allow the individual as much time as needed to respond. You may use the prompt, **"Was hail on the list? Yes or no?"** The individual must give you a response for every word. If the individual is not sure, ask for a guess.

**Delayed Recognition (Forced Choice)**			
1. HORSE Y N	7. house Y N	13. HUT Y N	19. TENT Y N
2. ruby Y N	8. OPAL Y N	14. EMERALD Y N	20. mountain Y N
3. CAVE Y N	9. TIGER Y N	15. SAPPHIRE Y N	21. cat Y N
4. balloon Y N	10. boat Y N	16. dog Y N	22. HOTEL Y N
5. coffee Y N	11. scarf Y N	17. apartment Y N	23. COW Y N
6. LION Y N	12. PEARL Y N	18. penny Y N	24. diamond Y N

Total number of true-positive responses ("hits"): _________ **/ 12** (no shading)

Semantically-related false-positive errors: _________ /6 (light shading)

Semantically-unrelated false-positive errors: _________ /6 (darker shading)

Total number of false-positive errors: _________ **/12**

	**Raw Score**
**Total Recall** (sum of total correct responses for Trials 1, 2, & 3)	
**Delayed Recall** (Trial 4)	
**Retention (%)** [(Trial 4 ÷ Higher score of Trials 2 and 3) x 100]	
**Recognition Discrimination Index** (Total no. of true-positives) – (Total no. of false-positives)	

Adapted and reproduced by special permission of the Publisher, Psychological Assessment Resources, Inc., 16204 North Florida Avenue, Lutz, Florida 33549, from the Hopkins Verbal Learning Test – Revised by Jason Brandt, PhD and Ralph H.B. Benedict, PhD, Copyright 1991, 1998, 2001 by PAR, Inc. Further reproduction is prohibited without permission from PAR, Inc.
Version 09-Sep-13

Appendix 9.5.2: HVLT-R (Form 2) [ENGLISH]

Found in [HVLT-R (Form 2) (EN, 09Sep2013) - CAPTURE ALS]

CAPTURE-ASL
Hopkins Verbal Learning Test-Revised (HVLT-R)

FORM 2

PARTICIPANT ID:	
DATE:	
VISIT:	

Word List
**FORK**
**RUM**
**PAN**
**PISTOL**
**SWORD**
**SPATULA**
**BOURBON**
**VODKA**
**POT**
**BOMB**
**RIFLE**
**WINE**

	**Learning Trials**			**Delayed Recall Trial (20-25 mins)**
	Trial 1	Trial 2	Trial 3	Trial 4
**Total correct responses =**				

Completion Time Trial 3 _________ **Start Time Trial 4** __________

Adapted and reproduced by special permission of the Publisher, Psychological Assessment Resources, Inc., 16204 North Florida Avenue, Lutz, Florida 33549, from the Hopkins Verbal Learning Test – Revised by Jason Brandt, PhD and Ralph H.B. Benedict, PhD, Copyright 1991, 1998, 2001 by PAR, Inc. Further reproduction is prohibited without permission from PAR, Inc.
Version 09-Sep-13

CAPTURE-ALS
Hopkins Verbal Learning Test-Revised (HVLT-R)
FORM 2

PARTICIPANT ID:	
DATE:	
VISIT:	

Delayed Recognition Instructions

The Delayed Recognition (Forced Choice) trial is administered immediately after the Delayed Recall trial. Say the following:

> **Now I am going to read a longer list of words to you. some of them are words from the original list, and some are not. After I read each word, I'd like you to say "Yes" if it was on the original list, or "No" if it was not.**

Read the words or the Delayed Recognition trial list in numerical order. Allow the individual as much time as needed to respond. You may use the prompt, **"Was hail on the list? Yes or no?"** The individual must give you a response for every word. If the individual is not sure, ask for a guess.

Delayed Recognition (Forced Choice)			
1. spoon Y N	7. harmonica Y N	13. knife Y N	19. WINE Y N
2. PISTOL Y N	8. can opener Y N	14. RUM Y N	20. lemon Y N
3. doll Y N	9. SWORD Y N	15. trout Y N	21. SPATULA Y N
4. whiskey Y N	10. pencil Y N	16. BOMB Y N	22. BOURBON Y N
5. FORK Y N	11. gun Y N	17. PAN Y N	23. beer Y N
6. POT Y N	12. VODKA Y N	18. gold Y N	24. RIFLE Y N

Total number of true-positive responses ("hits"): _________ **/ 12** (no shading)

Semantically-related false-positive errors: _________ /6 (light shading)

Semantically-unrelated false-positive errors: _________ /6 (darker shading)

Total number of false-positive errors: _________ **/12**

	Raw Score
**Total Recall** (sum of total correct responses for Trials 1, 2, & 3)	
**Delayed Recall** (Trial 4)	
**Retention (%)** [(Trial 4 ÷ Higher score of Trials 2 and 3) x 100]	
**Recognition Discrimination Index** (Total no. of true-positives) – (Total no. of false-positives)	

Adapted and reproduced by special permission of the Publisher, Psychological Assessment Resources, Inc., 16204 North Florida Avenue, Lutz, Florida 33549, from the Hopkins Verbal Learning Test – Revised by Jason Brandt, PhD and Ralph H.B. Benedict, PhD, Copyright 1991, 1998, 2001 by PAR, Inc. Further reproduction is prohibited without permission from PAR, Inc.
Version 09-Sep-13

Appendix 9.5.3: HVLT-R (Form 1) [FRENCH]
Found in [HVLT-R (Form 1) (FR) - CAPTURE ALS]

HVLT-R^{MC}

<u>Formulaire 1</u>

Cahier de l'examinateur

Jason Brandt, Ph. D.
Ralph H. B. Benedict, Ph. D.

<u>PAR</u> Psychological Assessment Resources, Inc. · 16204 N. Florida Ave., Lutz, FL 33549 · Numéro sans frais : 1 800 331-TEST · <u>www.parinc.com</u>

© 1991, 1998, 2001 Psychological Assessment Resources, Inc. Tous droits réservés. Ne peut être reproduit en totalité ou en partie sous quelque forme ou de quelque manière que ce soit sans l'autorisation écrite de Psychological Assessment Resources, Inc. Ce formulaire est imprimé à l'encre bleu sarcelle sur papier blanc. Aucune autre version n'est autorisée.

9 8 7 6 5 Réapprovisionnement RO-4753 Imprimé aux États-Unis

Instructions concernant les essais d'apprentissage

Essai 1

Dites ce qui suit :

> **Je vais vous lire une liste de mots. Écoutez attentivement, car lorsque j'aurai terminé, j'aimerais que vous me nommiez autant de mots que vous pourrez vous rappeler. Vous pouvez me les dire dans n'importe quel ordre. Êtes-vous prêt?**

- Répétez ou reformulez les instructions au besoin.
- Lisez les mots au rythme d'environ un mot toutes les deux secondes.
- Si la personne ne commence pas spontanément à énumérer des mots après que vous aurez lu le dernier mot de la liste, dites ce qui suit :

> **D'accord. Maintenant, nommez-moi autant de mots de cette liste que vous pouvez vous rappeler.**

Inscrivez textuellement les réponses données (en indiquant les répétitions et les intrus) dans la colonne « Essai 1 ». Lorsque la personne interrogée indique qu'elle ne peut plus se rappeler d'autres mots, passez à l'Essai 2.

Essai 2

Dites ce qui suit :

> **Nous allons faire un nouvel essai. Je vais vous lire la même liste de mots. Écoutez attentivement, puis nommez-moi autant de mots que vous pouvez vous rappeler, dans n'importe quel ordre, y compris tous les mots que vous m'avez mentionnés la première fois.**

Inscrivez les réponses dans la colonne « Essai 2 », en procédant de la même manière que pour l'Essai 1. Passez ensuite à l'Essai 3.

Essai 3

Dites ce qui suit :

> **Je vais lire la liste encore une fois. Comme plus tôt, j'aimerais que vous me nommiez autant de mots que vous pouvez vous rappeler, dans n'importe quel ordre, y compris tous les mots que vous m'avez déjà mentionnés.**

Inscrivez les réponses dans la colonne « Essai 3 », en procédant de la même manière que pour les autres essais.

REMARQUE : *Ne dites pas à la personne interrogée que la mémorisation des mots sera de nouveau vérifiée plus tard.*

Instructions concernant l'essai de rappel différé

Après un délai de 20 à 25 minutes, dites ce qui suit :

> **Vous rappelez-vous la liste de mots que vous avez essayé d'apprendre plus tôt?**

Dans la négative, rappelez à la personne que vous lui avez lu la liste trois fois et que vous lui avez demandé à chaque fois les mots dont elle se souvenait. Dites ce qui suit :

> **Nommez-moi autant de mots de la liste que vous pouvez vous rappeler.**

Formulaire 1

Catégories sémantiques : Quadrupèdes, pierres précieuses, habitations humaines

Nom : ______________________________ Sexe : _____ Âge : ____ années ____ mois
Examinateur : __________________________________ Date : _________________

**Liste de mots**	Essais d'apprentissage			**Rappel différé** (20 à 25 min)
	Essai 1	Essai 2	Essai 3	Essai 4
LION				
ÉMERAUDE				
CHEVAL				
TENTE				
SAPHIR				
HÔTEL				
CAVERNE				
OPALE				
TIGRE				
PERLE				
VACHE				
CABANE				
Total – réponses correctes =				

Heure de fin – Essai 3 _____

Heure de début – Essai 4 _____

Instructions concernant l'essai de reconnaissance différée

L'essai de reconnaissance différée (choix forcé) a lieu immédiatement après l'essai de rappel différé. Dites ce qui suit :

> **Je vais maintenant vous lire une liste de mots plus longue. Certains de ces mots font partie de la liste originale, tandis que d'autres n'en font pas partie. Après que j'aurai lu chaque mot, j'aimerais que vous répondiez par « oui » ou « non » à la question suivante : ce mot figurait-il dans la liste originale?**

Lisez les mots de l'essai de reconnaissance différée en suivant l'ordre numérique. Donnez à la personne interrogée tout le temps dont elle a besoin pour répondre. Vous pouvez utiliser la question : **« Le mot cheval était-il dans la liste, oui ou non? »** La personne doit donner une réponse pour chaque mot. Si elle n'est pas certaine, dites-lui de deviner.

Essai de reconnaissance différée (choix forcé)			
1. CHEVAL O N	7. maison O N	13. CABANE O N	19. TENTE O N
2. rubis O N	8. OPALE O N	14. ÉMERAUDE O N	20. montagne O N
3. CAVERNE O N	9. TIGRE O N	15. SAPHIR O N	21. chat O N
4. ballon O N	10. bateau O N	16. chien O N	22. HÔTEL O N
5. café O N	11. écharpe O N	17. appartement O N	23. VACHE O N
6. LION O N	12. PERLE O N	18. sou O N	24. diamant O N

Nombre total de réponses correctes positives : _____**/12** (cases non ombragées)

Réponses incorrectes positives avec lien sémantique : _____/6 (cases gris pâle)
Réponses incorrectes positives sans lien sémantique : _____/6 (cases gris foncé)

Nombre total de réponses incorrectes positives : _____**/12**

	Note brute	Note T
**Rappel – total** (somme des réponses correctes aux essais 1, 2 et 3)		
**Rappel différé** (Essai 4)		
**Rétention (%)** [(Essai 4 ÷ note la plus élevée parmi les essais 2 et 3) x 100]		
**Indice de discrimination – reconnaissance** (nombre total de réponses correctes positives) – (nombre total de réponses incorrectes positives)		

Tableau normatif (annexe A) : ____________________

Appendix 9.5.4: HVLT-R (Form 2) [FRENCH]
Found in [HVLT-R (Form 2) (FR) - CAPTURE ALS]

HVLT-R^MC

Formulaire 2

Cahier de l'examinateur

Jason Brandt, Ph. D.
Ralph H. B. Benedict, Ph. D.

PAR Psychological Assessment Resources, Inc. · 16204 N. Florida Ave., Lutz, FL 33549 · Numéro sans frais : 1 800 331-TEST · www.parinc.com

© 1991, 1998, 2001 Psychological Assessment Resources, Inc. Tous droits réservés. Ne peut être reproduit en totalité ou en partie sous quelque forme ou de quelque manière que ce soit sans l'autorisation écrite de Psychological Assessment Resources, Inc. Ce formulaire est imprimé à l'encre bourgogne sur papier blanc. Aucune autre version n'est autorisée.

9 8 7 6 5 4 Réapprovisionnement RO-4754 Imprimé aux États-Unis

Instructions concernant les essais d'apprentissage

Essai 1

Dites ce qui suit :

> **Je vais vous lire une liste de mots. Écoutez attentivement, car lorsque j'aurai terminé, j'aimerais que vous me nommiez autant de mots que vous pourrez vous rappeler. Vous pouvez me les dire dans n'importe quel ordre. Êtes-vous prêt?**

- Répétez ou reformulez les instructions au besoin.
- Lisez les mots au rythme d'environ un mot toutes les deux secondes.
- Si la personne ne commence pas spontanément à énumérer des mots après que vous aurez lu le dernier mot de la liste, dites ce qui suit :

> **D'accord. Maintenant, nommez-moi autant de mots de cette liste que vous pouvez vous rappeler.**

Inscrivez textuellement les réponses données (en indiquant les répétitions et les intrus) dans la colonne « Essai 1 ». Lorsque la personne interrogée indique qu'elle ne peut plus se rappeler d'autres mots, passez à l'Essai 2.

Essai 2

Dites ce qui suit :

> **Nous allons faire un nouvel essai. Je vais vous lire la même liste de mots. Écoutez attentivement, puis nommez-moi autant de mots que vous pouvez vous rappeler, dans n'importe quel ordre, y compris tous les mots que vous m'avez mentionnés la première fois.**

Inscrivez les réponses dans la colonne « Essai 2 », en procédant de la même manière que pour l'Essai 1. Passez ensuite à l'Essai 3.

Essai 3

Dites ce qui suit :

> **Je vais lire la liste encore une fois. Comme plus tôt, j'aimerais que vous me nommiez autant de mots que vous pouvez vous rappeler, dans n'importe quel ordre, y compris tous les mots que vous m'avez déjà mentionnés.**

Inscrivez les réponses dans la colonne « Essai 3 », en procédant de la même manière que pour les autres essais.

REMARQUE : *Ne dites pas à la personne interrogée que la mémorisation des mots sera de nouveau vérifiée plus tard.*

Instructions concernant l'essai de rappel différé

Après un délai de 20 à 25 minutes, dites ce qui suit :

> **Vous rappelez-vous la liste de mots que vous avez essayé d'apprendre plus tôt?**

Dans la négative, rappelez à la personne que vous lui avez lu la liste trois fois et que vous lui avez demandé à chaque fois les mots dont elle se souvenait. Dites ce qui suit :

> **Nommez-moi autant de mots de la liste que vous pouvez vous rappeler.**

Formulaire 2

Catégories sémantiques : Ustensiles de cuisine, boissons alcoolisées, armes

Nom : ______________________________ Sexe : _____ Âge : ____ années ____ mois
Examinateur : ___________________________________ Date : _________________

**Liste de mots**	Essais d'apprentissage			**Rappel différé** (20 à 25 min)
FOURCHETTE	Essai 1	Essai 2	Essai 3	Essai 4
RHUM				
CASSEROLE				
PISTOLET				
ÉPÉE				
SPATULE				
BOURBON				
VODKA				
MARMITE				
BOMBE				
CARABINE				
VIN				
Total – réponses correctes =				

Durée de l'essai – Essai 3 _____

Heure de début – Essai 4 _____

Instructions concernant l'essai de reconnaissance différée

L'essai de reconnaissance différée (choix forcé) a lieu immédiatement après l'essai de rappel différé. Dites ce qui suit :

> **Je vais maintenant vous lire une liste de mots plus longue. Certains de ces mots font partie de la liste originale, tandis que d'autres n'en font pas partie. Après que j'aurai lu chaque mot, j'aimerais que vous répondiez par « oui » ou « non » à la question suivante : ce mot figurait-il dans la liste originale?**

Lisez les mots de l'essai de reconnaissance différée en suivant l'ordre numérique. Donnez à la personne interrogée tout le temps dont elle a besoin pour répondre. Vous pouvez utiliser la question : **« Le mot cuillère était-il dans la liste, oui ou non? »** La personne doit donner une réponse pour chaque mot. Si elle n'est pas certaine, dites-lui de deviner.

**Essai de reconnaissance différée (choix forcé)**			
1. cuillère O N	7. harmonica O N	13. couteau O N	19. VIN O N
2. PISTOLET O N	8. ouvre-boîte O N	14. RHUM O N	20. citron O N
3. poupée O N	9. ÉPÉE O N	15. truite O N	21. SPATULE O N
4. whiskey O N	10. crayon O N	16. BOMBE O N	22. BOURBON O N
5. FOURCHETTE O N	11. fusil O N	17. CASSEROLE O N	23. bière O N
6. MARMITE O N	12. VODKA O N	18. or O N	24. CARABINE O N

Nombre total de réponses correctes positives : _____**/12** (cases non ombragées)

Réponses incorrectes positives avec lien sémantique : _____/6 (cases gris pâle)
Réponses incorrectes positives sans lien sémantique : _____/6 (cases gris foncé)

Nombre total de réponses incorrectes positives : _____**/12**

	Note brute	Note T
**Rappel – total** (somme des réponses correctes aux essais 1, 2 et 3)		
**Rappel différé** (Essai 4)		
**Rétention (%)** [(Essai 4 ÷ note la plus élevée parmi les essais 2 et 3) x 100]		
**Indice de discrimination – reconnaissance** (nombre total de réponses correctes positives) – (nombre total de réponses incorrectes positives)		

Tableau normatif (annexe A) : ____________________

Appendix 9.6: Social Norms Questionnaire

Appendix 9.6.1: Social Norms Questionnaire [ENGLISH]
Appendix 9.6.2: Social Norms Questionnaire [FRENCH]
Appendix 9.6.3: Social Norms Questionnaire Scoring Instructions [ENGLISH]
Appendix 9.6.4: Social Norms Questionnaire Scoring Instructions [FRENCH]

Appendix 9.6.1: Social Norms Questionnaire [ENGLISH]
Found in [Social Norms Questionnaire (EN, 09Sep2016) - CAPTURE ALS]

CAPTURE-ALS<br>**Social Norms Questionnaire**	
PARTICIPANT ID:	
DATE:	
VISIT:	

Instructions
The following is a list of behaviours that a person might engage in. Please decide whether or not it would be socially acceptable and appropriate to do these things in the mainstream culture of Canada and answer yes or no to each. Think about these questions as if they were occurring in front of or with a stranger or acquaintance, NOT a close friend or family member.

Would it be socially acceptable to:

1.	Tell a stranger you don't like their hairstyle?	☐ NO	☐ YES
2.	Spit on the floor?	☐ NO	☐ YES
3.	Blow your nose in public?	☐ NO	☐ YES
4.	As a coworker their age?	☐ NO	☐ YES
5.	Cry during a movie at the theatre?	☐ NO	☐ YES
6.	Cut in line if you are in a hurry?	☐ NO	☐ YES
7.	Laugh when you yourself trip and fall?	☐ NO	☐ YES
8.	Eat pasta with your fingers?	☐ NO	☐ YES
9.	Tell a coworker your age?	☐ NO	☐ YES
10.	Tell someone your opinion of a movie they haven't seen?	☐ NO	☐ YES
11.	Laugh when someone else trips and falls?	☐ NO	☐ YES
12.	Wear the same shirt every day?	☐ NO	☐ YES
13.	Keep money you find on the side walk?	☐ NO	☐ YES
14.	Pick your nose in public?	☐ NO	☐ YES
15.	Tell a coworker you think they are overweight?	☐ NO	☐ YES
16.	Eat ribs with your fingers?	☐ NO	☐ YES
17.	Tell a stranger you like their hairstyle?	☐ NO	☐ YES
18.	Wear the same shirt twice in two weeks?	☐ NO	☐ YES
19.	Tell someone the ending of a movie they haven't seen?	☐ NO	☐ YES
20.	Hug a stranger without asking first?	☐ NO	☐ YES
21.	Talk out loud during a movie at a theatre?	☐ NO	☐ YES
22.	Tell a coworker you think they have lost weight?	☐ NO	☐ YES

Reproduced with permission of the author, Katherine Rankin, PhD, do not copy or distribute without author's permission.

Appendix 9.6.2: Social Norms Questionnaire [FRENCH]

Found in [Social Norms Questionnaire (FR, 15May2017) - CAPTURE ALS]

PARTICIPANT ID:	
DATE:	
VISIT:	

CAPTURE-ALS
Questionnaire de normes sociales

Instructions

Voici une liste de différents comportements qu'une personne pourrait avoir. Pour chacun des comportements décrits ci-dessous, veuillez déterminer si OUI ou NON, il serait socialement acceptable et approprié dans la culture populaire canadienne de se comporter de cette façon. Répondez par OUI ou par NON à chaque question. Réfléchissez à ces situations comme si elles se produisaient en présence d'un étranger ou d'une connaissance et non pas en présence d'un ami proche ou d'un membre de votre famille.

Serait-il socialement acceptable de:

1.	Dire à un(e) étranger(ère) que vous n'aimez pas sa coiffure?	☐ NON	☐ OUI
2.	Cracher pas terre?	☐ NON	☐ OUI
3.	Se moucher en public?	☐ NON	☐ OUI
4.	Demander son âge à un/une collègue de travail?	☐ NON	☐ OUI
5.	Pleurer en regardant un film au cinéma?	☐ NON	☐ OUI
6.	Dépasser les personnes en file d'attente parce que vous êtes pressé(e)?	☐ NON	☐ OUI
7.	Rire lorsque vous trébuchez et tombez?	☐ NON	☐ OUI
8.	Manger des pâtes avec vos doigts?	☐ NON	☐ OUI
9.	Dire votre âge à un/une collègue de travail?	☐ NON	☐ OUI
10.	Donnez votre avis à quelqu'un au sujet d'un film qu'il/elle n'a pas vu?	☐ NON	☐ OUI
11.	Rire lorsque quelqu'un trébuche et tombe?	☐ NON	☐ OUI
12.	Porter la même chemise tous les jours?	☐ NON	☐ OUI
13.	Garder l'argent que vous avez trouvé sur le trottoir?	☐ NON	☐ OUI
14.	Se mettre les doigts dans le nez en public?	☐ NON	☐ OUI
15.	Dire à un/une collègue de travail qu'il/elle a un surplus de poids?	☐ NON	☐ OUI
16.	Manger des côtes levées avec vos doigts?	☐ NON	☐ OUI
17.	Dire à un(e) étranger(ère) que vous aimez sa coiffure?	☐ NON	☐ OUI
18.	Porter la même chemise deux fois en deux semaines?	☐ NON	☐ OUI
19.	Raconter à quelqu'un la fin d'un film qu'il/elle n'a pas encore vu?	☐ NON	☐ OUI
20.	Serrer un étranger dans ses bras sans lui demander la permission?	☐ NON	☐ OUI
21.	Parler à voix haute durant un film au cinéma?	☐ NON	☐ OUI
22.	Dire à votre collègue que vous croyez qu'il/elle a perdu du poids?	☐ NON	☐ OUI

Reproduit avec la permission de l'auteur: Katherine Rankin, PhD. Traduction française mise au point par une collaboration entre CIMA-Q et CCNV.

Appendix 9.6.3: Social Norms Questionnaire Scoring Instructions [ENGLISH]

Found in [Social Norms Questionnaire Scoring Instruction (EN, 18Mar2022) - CAPTURE ALS]

CAPTURE - ALS<br><br>Social Norms Questionnaire Scoring	PARTICIPANT ID:	
	DATE:	
	VISIT:	

	Summary Score	Description	Score
1.	Sum of items 1 to 22	Use scoring key for correct responses;<br>**Correct = 0; Incorrect = 1 point**	Range: 0 - 22
2.	SNQ22 Total Score	Calculated as: 22 – Sum of items 1 to 22	Range: 0 - 22
3.	Break Score	Calculated as: Sum of items with grey background in the Scoring Key (i.e. 1, 2, 4, 5, 8, 11, 12, 14, 15, 19, 20, 21)	Range: 0 - 12
4.	Overadhere Score	Calculated as: Sum of items with white background in the Scoring Key (i.e. 3, 6, 7, 9, 10, 13, 16, 17, 18, 22	Range: 0 - 10
5.	Yes/No Ratio Score	Calculated as:<br>(Number of "Yes" responses) ÷ (Number of "No" responses)	

Note: If Yes/No Ratio score is higher than 5 or below 0.3; please consider whether the subject was too impaired to fill out the form or answered the questions in a meaningless way.

Scoring Key – Correct Responses

1.	Tell a stranger you don't like their hairstyle?	NO
2.	Spit on the floor?	NO
3.	Blow your nose in public?	YES
4.	Ask a coworker their age?	NO
5.	Cry during a movie at the theatre?	YES
6.	Cut in line if you are in a hurry?	NO
7.	Laugh when you yourself trip and fall?	YES
8.	Eat pasta with your fingers?	NO
9.	Tell a coworker your age?	YES
10.	Tell someone your opinion of a movie they haven't seen?	YES
11.	Laugh when someone else trips and falls?	NO
12.	Wear the same shirt every day?	NO
13.	Keep money you find on the side walk?	YES
14.	Pick your nose in public?	NO
15.	Tell a coworker you think they are overweight?	NO
16.	Eat ribs with your fingers?	YES
17.	Tell a stranger you like their hairstyle?	YES
18.	Wear the same shirt twice in two weeks?	YES
19.	Tell someone the ending of a movie they haven't seen?	NO
20.	Hug a stranger without asking first?	NO
21.	Talk out loud during a movie at the theatre?	NO
22.	Tell a coworker you think they have lost weight?	YES

Appendix 9.6.4: Social Norms Questionnaire Scoring Instructions [FRENCH]
Found in [Social Norms Questionnaire Scoring Instruction (FR, 18Mar2022) - CAPTURE ALS]

CAPTURE - ALS		
Social Norms Questionnaire Scoring	PARTICIPANT ID:	
	DATE:	
	VISIT:	

	Summary Score	Description	Score
1.	Sum of items 1 to 22	Use scoring key for correct responses; **Correct = 0; Incorrect = 1 point**	Range: 0 - 22
2.	SNQ22 Total Score	Calculated as: 22 – Sum of items 1 to 22	Range: 0 - 22
3.	Break Score	Calculated as: Sum of items with grey background in the Scoring Key (i.e. 1, 2, 4, 5, 8, 11, 12, 14, 15, 19, 20, 21)	Range: 0 - 12
4.	Overadhere Score	Calculated as: Sum of items with white background in the Scoring Key (i.e. 3, 6, 7, 9, 10, 13, 16, 17, 18, 22	Range: 0 - 10
5.	Yes/No Ratio Score	Calculated as: (Number of "Yes" responses) ÷ (Number of "No" responses)	

Note: If Yes/No Ratio score is higher than 5 or below 0.3; please consider whether the subject was too impaired to fill out the form or answered the questions in a meaningless way.

Scoring Key – Correct Responses

1.	Dire à un(e) étranger(ère) que vous n'aimez pas sa coiffure?	NO
2.	Cracher pas terre?	NO
3.	Se moucher en public? your nose in public?	YES
4.	Demander son âge à un/une collègue de travail?	NO
5.	Pleurer en regardant un film au cinéma?	YES
6.	Dépasser les personnes en file d'attente parce que vous êtes pressé(e)?	NO
7.	Rire lorsque vous trébuchez et tombez?	YES
8.	Manger des pâtes avec vos doigts?	NO
9.	Dire votre âge à un/une collègue de travail?	YES
10.	Donnez votre avis à quelqu'un au sujet d'un film qu'il/elle n'a pas vu?	YES
11.	Rire lorsque quelqu'un trébuche et tombe?	NO
12.	Porter la même chemise tous les jours?	NO
13.	Garder l'argent que vous avez trouvé sur le trottoir?	YES
14.	Se mettre les doigts dans le nez en public?	NO
15.	Dire à un/une collègue de travail qu'il/elle a un surplus de poids?	NO
16.	Manger des côtes levées avec vos doigts?	YES
17.	Dire à un(e) étranger(ère) que vous aimez sa coiffure?	YES
18.	Porter la même chemise deux fois en deux semaines?	YES
19.	Raconter à quelqu'un la fin d'un film qu'il/elle n'a pas encore vu?	NO
20.	Serrer un étranger dans ses bras sans lui demander la permission?	NO
21.	Parler à voix haute durant un film au cinéma?	NO
22.	Dire à votre collègue que vous croyez qu'il/elle a perdu du poids?	YES

Appendix 9.7: Hospital Anxiety and Depression Scale (HADS)

Appendix 9.7.1: HADS [ENGLISH]
Appendix 9.7.2: HADS [FRENCH]

Appendix 9.7.1: HADS [ENGLISH]

Found in [HADS (EN, V3, 16FEB2009) CAPTURE ALS]

CAPTURE ALS

PROTOCOL: CAPTURE ALS-1 CONFIDENTAL

Hospital Anxiety and Depression Scale (HADS)

GL assessment the measure of potential

Participant ID: ______________________________ Date: ______________

FOLD HERE

Clinicians are aware that emotions play an important part in most illnesses. If your clinician knows about these feelings, he/she will be able to help you more.

This questionnaire is designed to help your clinician know how you feel. Read each item below and **underline the reply** which comes closest to how you have been feeling in the past 7 days. Ignore the numbers printed at the edge of the questionnaire.

Don't take too long over your replies: your immediate reaction to each item will probably be more accurate than a long thought-out response.

FOLD HERE

A	D	
		**I feel tense or 'wound up'**
3		Most of the time
2		A lot of the time
1		From time to time, occasionally
0		Not at all
		**I still enjoy the things I used to enjoy**
	0	Definitely as much
	1	Not quite so much
	2	Only a little
	3	Hardly at all
		**I get a sort of frightened feeling as if something awful is about to happen**
3		Very definitely and quite badly
2		Yes, but not too badly
1		A little, but it doesn't worry me
0		Not at all
		**I can laugh and see the funny side of things**
	0	As much as I always could
	1	Not quite so much now
	2	Definitely not so much now
	3	Not at all
		**Worrying thoughts go through my mind**
3		A great deal of the time
2		A lot of the time
1		Not too often
0		Very little
		**I feel cheerful**
	3	Never
	2	Not often
	1	Sometimes
	0	Most of the time
		**I can sit down feeling relaxed and at ease**
0		Definitely
1		Usually
2		Not often
3		Not at all

	A	D
**I feel as if I am slowed down**		
Nearly all the time		3
Very often		2
Sometimes		1
Not at all		0
**I get a sort of frightened feeling like 'butterflies' in the stomach**		
Not at all	0	
Occasionally	1	
Quite often	2	
Very often	3	
**I have lost interest in my appearance**		
Definitely		3
I don't take as much care as I should		2
I may not take quite as much care		1
I take just as much care as ever		0
**I feel restless as if I have to be on the move**		
Very much	3	
Quite a lot	2	
Not very much	1	
Not at all	0	
**I look forward with enjoyment to things**		
As much as I ever did		0
Rather less than I used to		1
Definitely less than I used to		2
Hardly at all		3
**I get sudden feelings of panic**		
Very often	3	
Quite often	2	
Not very often	1	
Not at all	0	
**I can enjoy a good book or radio or television program**		
Often		0
Sometimes		1
Not often		2
Very seldom		3

Please make sure you have answered all the questions

	A	D
**TOTAL**		

HADS copyright © R.P. Snaith and A.S. Zigmond, 1983, 1992, 1994. Record form items originally published in *Acta Psychiatrica Scandinavica*, 67, 361–70, copyright © Munksgaard International Publishers Ltd, Copenhagen, 1983.
This edition first published in 1994 by nferNelson Publishing Company Ltd,
389 Chiswick High Road, 9th Floor East, London, W4 4AL
GL Assessment is part of the Granada Group
This form may not be reproduced by any means without first obtaining permission from the publisher.
Email: permissions@gl-assessment.co.uk

Appendix 9.7.2: HADS [FRENCH]

Found in [HADS (FR, V3, 16FEB2009) CAPTURE ALS]

PROTOCOL: CAPTURE ALS-1 CONFIDENTAL

CAPTURE ALS

Questionnaire sur l'anxiété et la dépression en milieu hospitalier (HADS)

GL assessment — the measure of potential

Participant ID : ______________________________ Date : ______________

PLIEZ ICI

Les médecins savent que les émotions jouent un rôle important dans la plupart des maladies. Si vous informez votre médecin des émotions que vous éprouvez, il sera en mesure de mieux vous aider.

Ce questionnaire est conçu pour aider votre médecin à savoir comment vous vous sentez. Lisez chaque énoncé et **soulignez la réponse** qui exprime le mieux ce que vous avez ressenti au cours des 7 derniers jours. Ne tenez pas compte des numéros qui se trouvent de chaque côté du questionnaire.

Ne mettez pas trop de temps à répondre, votre réaction immédiate à chaque énoncé sera probablement plus précise qu'une réponse longuement réfléchie.

PLIEZ ICI

A	D	
		**Je me sens tendu(e) ou nerveux(se).**
3		La plupart du temps
2		Souvent
1		De temps en temps, occasionnellement
0		Jamais
		**Je continue de prendre plaisir aux choses qui me plaisaient auparavant.**
	0	Toujours autant
	1	Pas autant
	2	Seulement un peu
	3	Plus du tout
		**Je suis pris(e) d'un sentiment de peur, comme si quelque chose de terrible allait arriver.**
3		Oui et assez intensément
2		Oui, mais pas trop intensément
1		Un peu, mais cela ne m'inquiète pas
0		Jamais
		**Je peux rire et voir le côté amusant des choses.**
	0	Autant qu'avant
	1	Pas autant qu'avant
	2	Nettement moins qu'avant
	3	Pas du tout
		**Des pensées inquiétantes me passent par la tête.**
3		La plupart du temps
2		Souvent
1		Pas très souvent
0		Rarement
		**Je suis joyeux(se).**
	3	Jamais
	2	Pas souvent
	1	Parfois
	0	La plupart du temps
		**Je peux m'asseoir l'esprit tranquille et me sentir détendu(e).**
0		Toujours
1		Souvent
2		Rarement
3		Jamais

	A	D
**J'ai l'impression de fonctionner au ralenti.**		
Presque tout le temps		3
Très souvent		2
Parfois		1
Jamais		0
**Je suis pris(e) d'un sentiment de peur, j'ai comme un nœud dans l'estomac.**		
Jamais	0	
Occasionnellement	1	
Assez souvent	2	
Très souvent	3	
**Je ne m'intéresse plus à mon apparence.**		
Plus du tout		3
Souvent je n'y accorde pas d'attention		2
Parfois je n'y accorde pas d'attention		1
J'y accorde autant d'attention qu'avant		0
**J'ai la bougeotte, c'est comme si je n'arrivais pas à tenir en place.**		
Beaucoup	3	
Assez	2	
Pas beaucoup	1	
Pas du tout	0	
**Je me réjouis d'avance à l'idée de faire certaines choses.**		
Autant qu'avant		0
Un peu moins qu'avant		1
Nettement moins qu'avant		2
Plus du tout		3
**J'ai des sentiments soudains de panique.**		
Très souvent	3	
Assez souvent	2	
Pas très souvent	1	
Jamais	0	
**Je peux prendre plaisir à un bon livre ou à une bonne émission de radio ou de télévision.**		
Souvent		0
Parfois		1
Rarement		2
Très rarement		3

Vérifiez maintenant que vous avez répondu à toutes les questions.

	A	D
**TOTAL**		

HADS © R.P. Snaith et A.S. Zigmond, 1983, 1992, 1994.
Les items de ce questionnaire ont été publiés à l'origine dans *Acta Psychiatrica Scandinavica*, 67, 361–70,
© Munksgaard International Publishers Ltd, Copenhagen, 1983.
Cette édition a été publiée pour la première fois en 1994 par nferNelson Publishing Company Ltd,
389 Chiswick High Road, 9th Floor East, London, W4 4AL
GL Assessment fait partie de Granada Group
Ce questionnaire ne peut être reproduit d'aucune façon sans la permission préalable de l'éditeur.
Courriel : permissions@gl-assessment.co.uk

Appendix 9.8: Center for Neurological Study-Lability Scale (CNS-LS)

Appendix 9.8.1: CNS-LS [ENGLISH]
Appendix 9.8.2: CNS-LS (FR, APR2016) - CAPTURE ALS

CAPTURE ALS THE SOLUTION IS IN YOU

Appendix 9.8.1: CNS-LS [ENGLISH]

Found in [CNS-LS (EN, 2020) - CAPTURE ALS]

CENTER FOR NEUROLOGIC STUDY-LABILITY SCALE (CNS-LS) FOR PSEUDOBULBAR AFFECT (PBA)

The CNS-LS is a short (seven-item), self-administered questionnaire, designed to be completed by the patient, that provides a quantitative measure of the perceived frequency of PBA episodes. The CNS-LS can assist in evaluating patients who may be experiencing symptoms of PBA. A CNS-LS score of 13 or higher may suggest PBA.[1] This score does not diagnose PBA, and lower or higher scores can occur in persons with and without PBA.

NAME ______________________________ *DATE OF ASSESSMENT* _____/_____/_____

Using the scale below, please write the number that describes the degree to which each item applies to you **DURING THE PAST WEEK**.[1] Write only 1 number for each item.[1]

1	2	3	4	5
Applies never	Applies rarely	Applies occasionally	Applies frequently	Applies most of the time

ASSESSMENT QUESTIONS		*ANSWER (1-5)*
1	There are times when I feel fine one minute, and then I'll become tearful the next over something small or for no reason at all.	
2	Others have told me that I seem to become amused very easily or that I seem to become amused about things that really aren't funny.	
3	I find myself crying very easily.	
4	I find that even when I try to control my laughter, I am often unable to do so.	
5	There are times when I won't be thinking of anything happy or funny at all, but then I'll suddenly be overcome by funny or happy thoughts.	
6	I find that even when I try to control my crying, I am often unable to do so.	
7	I find that I am easily overcome by laughter.	
	TOTAL SCORE :	

PLEASE PROVIDE THE COMPLETED QUESTIONNAIRE TO YOUR HEALTH CARE PROVIDER.

The CNS-LS has been validated in amyotrophic lateral sclerosis (ALS) and multiple sclerosis (MS) patient populations.[1,2]

This questionnaire is not intended to substitute for professional medical assessment and/or advice.

References: 1. Moore SR, Gresham LS, Bromberg MB, Kasarkis EJ, Smith RA. A self report measure of affective lability. *J Neurol Neurosurg Psychiatry.* 1997;63(1):89-93. **2.** Smith RA, Berg JE, Pope LE, Callahan JD, Wynn D, Thisted RA. Validation of the CNS emotional lability scale for pseudobulbar affect (pathological laughing and crying) in multiple sclerosis patients. *Mult Scler.* 2004;10(6):679-685.

©2020 Avanir Pharmaceuticals, Inc. All rights reserved. MLR-PBA-US-0453-1220

Appendix 9.8.2: CNS-LS [FRENCH]
Found in [CNS-LS (FR, APR2016) - CAPTURE ALS]

Centre d'Étude Neurologique - Échelle de labilité (CNS-LS) pour le syndrome pseudo-bulbaire (SPB)

Le CNS-LS est un questionnaire court (sept items), auto-administré, conçu pour être rempli par le patient, qui donne une mesure quantitative de la fréquence perçue des épisodes de SPB. Le CNS-LS peut aider les médecins à diagnostiquer précisément le SPB. Un score supérieur ou égal à 13 peut suggérer un SPB.

Nom du patient : ____________________

Date de l'évaluation : ____________________

En vous référant à l'échelle ci-dessous, veuillez noter le chiffre qui correspond au degré qui vous décrit le mieux pour chacun des items, AU COURS DE LA SEMAINE ÉCOULÉE. Indiquer un seul chiffre pour chaque item.

Ne s'applique jamais	S'applique rarement	S'applique occasionnellement	S'applique fréquemment	S'applique la plupart du temps
1	2	3	4	5

	Questions d'évaluation	Réponses
1	Parfois, je me sens bien pendant une minute, puis j'ai envie de pleurer pour une raison insignifiante ou sans aucune raison.	
2	D'autres personnes m'ont dit que je pouvais m'amuser très facilement ou sembler être amusé(e) par des choses qui ne sont en réalité pas amusantes.	
3	Je trouve que je pleure très facilement.	
4	Je trouve que même lorsque je tente de contrôler mon rire, j'en suis souvent incapable.	
5	Par moments, je ne pense à rien de joyeux ou de comique, mais je suis ensuite subitement submergé(e) par des pensées comiques ou joyeuses.	
6	Je trouve que même lorsque je tente de contrôler mes pleurs, j'en suis souvent incapable.	
7	Je me trouve facilement pris(e) de fous rires.	

Score total : ____________

Le CNS-LS a été validé dans des populations de patients atteints de SLA et de SEP.

Ce questionnaire n'est pas destiné à remplacer une évaluation et/ou un avis médical professionnel.

Référence : Moore SR, Gresham LS, Bromberg MB, Kasarkis EJ, Smith RA. A self report measure of affective lability. *J Neurol Neurosurg Psychiatry.* 1997;63(1):89-93.

AVANIR™ pharmaceuticals

© 2015 Avanir Pharmaceuticals, Inc. Tous droits réservés. CNS-LS French Version (v0.1) PBA-0047-OTH-1215

Appendix 9.9: Frontal Systems Behaviour Scale (FrSBe)

Appendix 9.9.1: FrSBe (Self-Rating form) [ENGLISH]
Appendix 9.9.2: FrSBe (Self-Rating form) [FRENCH]
Appendix 9.9.3: FrSBe (Family-Rating form) [ENGLISH]
Appendix 9.9.4: FrSBe (Family-Rating form) [FRENCH]

Appendix 9.9.1: FrSBe (Self-Rating form) [ENGLISH]
Found in [FrSBe (Self-Rating form) (EN, 27Sep2016) - CAPTURE ALS]

CAPTURE-ALS
FRONTAL SYSTEMS BEHAVIOUR SCALE (FrSBe)
SELF-RATING FORM

PARTICIPANT ID:
DATE:
VISIT:

Instructions:
Inside this form is a list of phrases that can be used to describe a person's behaviour. Please read each phrase carefully. Using the rating scale below, circle the box under each column that corresponds to how often you have engaged in the behaviour described. Rate your behaviour for each point in time - **Before the illness or injury** and **At the present time**. Please try to provide a rating for all of the statements.

1	2	3	4	5
Almost never	Seldom	Sometimes	Frequently	Almost always

Examples:
If during the time *before* your illness or injury you *almost never* felt confused, then you would tick the box under the 1 column labeled **Before illness or injury**. If at the *present* time you *frequently* feel confused, then you would tick the box under the 4 column labeled **At the present time**. If you need to change an answer after you have already circled it, just mark an X through the incorrect number and then circle the correct number.

	Before illness or injury	At the present time
1. I speak only when spoken to.	1 2 3 4 5	1 2 3 4 5
**2. I am easily angered or irritated; I have emotional outbursts without good reason.**	1 2 3 4 5	1 2 3 4 5
3. Repeat certain actions or get stuck on certain ideas.	1 2 3 4 5	1 2 3 4 5
**4. I do things impulsively.**	1 2 3 4 5	1 2 3 4 5
5. Mix up a sequence, get confused when doing several things in a row.	1 2 3 4 5	1 2 3 4 5
**6. Laugh or cry too easily.**	1 2 3 4 5	1 2 3 4 5
7. Make the same mistakes over and over, do not learn from past experience.	1 2 3 4 5	1 2 3 4 5
**8. Have difficulty starting an activity, lack initiative, motivation.**	1 2 3 4 5	1 2 3 4 5
9. Make inappropriate sexual comments and advances, am too flirtatious.	1 2 3 4 5	1 2 3 4 5
**10. Do or say embarrassing things.**	1 2 3 4 5	1 2 3 4 5
11. Neglect my personal hygiene.	1 2 3 4 5	1 2 3 4 5
**12. Can't sit still, am hyperactive.**	1 2 3 4 5	1 2 3 4 5
13. Am unaware of my problems or when I make mistakes.	1 2 3 4 5	1 2 3 4 5
**14. Sit around doing nothing.**	1 2 3 4 5	1 2 3 4 5
15. Am disorganized.	1 2 3 4 5	1 2 3 4 5
**16. Lose control of my urine or bowels and it doesn't seem to bother me.**	1 2 3 4 5	1 2 3 4 5
17. Cannot do two things at once (for example, talk and prepare a meal).	1 2 3 4 5	1 2 3 4 5
**18. Talk out of turn, interrupt others in conversations.**	1 2 3 4 5	1 2 3 4 5
19. Show poor judgment, poor problem solver.	1 2 3 4 5	1 2 3 4 5
**20. Make up fantastic stories when unable to remember something.**	1 2 3 4 5	1 2 3 4 5

Adapted and reproduced by special permission of the Publisher, Psychological Assessment Resources, Inc., 16204 North Florida Avenue, Lutz, Florida 33549, from the Frontal Systems Behavior Scale by Janet Grace, PhD and Paul F. Malloy, PhD, Copyright 1992, 2000, 2001 by PAR, Inc. Further reproduction is prohibited without permission of PAR, Inc.
Version 09-Sep-13

CAPTURE-ALS
FRONTAL SYSTEMS BEHAVIOUR SCALE (FrSBe)
SELF-RATING FORM

	Before illness or injury	At the present time
21. Have lost interest in things that used to be fun or important to me.	1 2 3 4 5	1 2 3 4 5
**22. Say one thing, then do another thing.**	**1 2 3 4 5**	**1 2 3 4 5**
23. Start things but fail to finish them, "peter out."	1 2 3 4 5	1 2 3 4 5
**24. Show little emotion, am unconcerned and unresponsive.**	**1 2 3 4 5**	**1 2 3 4 5**
25. Forget to do things but then remember when prompted or when it is too late.	1 2 3 4 5	1 2 3 4 5
**26. Am inflexible, unable to change routines.**	**1 2 3 4 5**	**1 2 3 4 5**
27. Get in trouble with the law or authorities.	1 2 3 4 5	1 2 3 4 5
**28. Do risky things just for the heck of it.**	**1 2 3 4 5**	**1 2 3 4 5**
29. Am slow moving, lack energy, inactive.	1 2 3 4 5	1 2 3 4 5
**30. Am overly silly, have a childish sense of humor.**	**1 2 3 4 5**	**1 2 3 4 5**
31. Find that food has no taste or smell.	1 2 3 4 5	1 2 3 4 5
**32. Swear.**	**1 2 3 4 5**	**1 2 3 4 5**

Read each of the following items carefully before responding

1	2	3	4	5
Almost always	Frequently	Sometimes	Seldom	Almost never

	Before illness or injury	At the present time
33. Apologize for misbehaviour (for example, apologize for swearing).	1 2 3 4 5	1 2 3 4 5
34. **Pay attention, concentrate even when there are distractions.**	**1 2 3 4 5**	**1 2 3 4 5**
35. Think things through before acting (for example, consider finances before spending money).	1 2 3 4 5	1 2 3 4 5
**36. Use strategies to remember important things (for example, write notes to myself).**	**1 2 3 4 5**	**1 2 3 4 5**
37. Am able to plan ahead.	1 2 3 4 5	1 2 3 4 5
**38. Am interested in sex.**	**1 2 3 4 5**	**1 2 3 4 5**
39. Care about my appearance (for example, daily grooming).	1 2 3 4 5	1 2 3 4 5
**40. Benefit from feedback, accept constructive criticism from others.**	**1 2 3 4 5**	**1 2 3 4 5**
41. Get involved with activities spontaneously (such as hobbies).	1 2 3 4 5	1 2 3 4 5
**42. Do things without being requested to do so.**	**1 2 3 4 5**	**1 2 3 4 5**
43. Am sensitive to the needs of other people.	1 2 3 4 5	1 2 3 4 5
**44. Get along well with others.**	**1 2 3 4 5**	**1 2 3 4 5**
45. Act appropriately for my age.	1 2 3 4 5	1 2 3 4 5
**46. Can start conversations easily.**	**1 2 3 4 5**	**1 2 3 4 5**

Adapted and reproduced by special permission of the Publisher, Psychological Assessment Resources, Inc., 16204 North Florida Avenue, Lutz, Florida 33549, from the Frontal Systems Behavior Scale by Janet Grace, PhD and Paul F. Malloy, PhD, Copyright 1992, 2000, 2001 by PAR, Inc. Further reproduction is prohibited without permission of PAR, Inc.
Version 09-Sep-13

CAPTURE-ALS
FRONTAL SYSTEMS BEHAVIOUR SCALE (FrSBe)
SCORING SHEET

PARTICIPANT ID:

DATE:

VISIT:

SCORING: Transfer each circled item score from the **BEFORE illness or injury** column to the corresponding shaded box in the **BEFORE** column below. Transfer each circled item score from the **At the PRESENT time** column to the corresponding shaded box in the AFTER column below. Sum the scores for each subscale (A, D, and E) and enter the total in the space provided at the bottom of each column. Sum the 2 subtotals on each page to obtain total subscale BEFORE and AFTER scores. Total all BEFORE and all AFTER scores.

	**BEFORE**			**AFTER**		
	Apathy	Disinhibition	Executive Dysfunction	Apathy	Disinhibition	Executive Dysfunction
1.						
2.						
3.						
4.						
5.						
6.						
7.						
8.						
9.						
10.						
11.						
12.						
13.						
14.						
15.						
16.						
17.						
18.						
19.						
20.						
21.						
22.						
23.						
24.						
	**Subtotal:**	**Subtotal:**	**Subtotal:**	**Subtotal:**	**Subtotal:**	**Subtotal:**

Adapted and reproduced by special permission of the Publisher, Psychological Assessment Resources, Inc., 16204 North Florida Avenue, Lutz, Florida 33549, from the Frontal Systems Behavior Scale by Janet Grace, PhD and Paul F. Malloy, PhD, Copyright 1992, 2000, 2001 by PAR, Inc. Further reproduction is prohibited without permission of PAR, Inc.
Version 09-Sep-13

	BEFORE Apathy	BEFORE Disinhibition	BEFORE Executive Dysfunction	AFTER Apathy	AFTER Disinhibition	AFTER Executive Dysfunction
25.						
26.						
27.						
28.						
29.						
30.						
31.						
32.						
33.						
34.						
35.						
36.						
37.						
38.						
39.						
40.						
41.						
42.						
43.						
44.						
45.						
46.						

Subtotals (page 4)						
Subtotals (page 3)						
**Subscale Totals**	*Apathy:*	*Disinhibition:*	*Executive Dys.:*	*Apathy:*	*Disinhibition:*	*Executive Dys.:*
	**Total BEFORE:**			**Total AFTER:**		

Adapted and reproduced by special permission of the Publisher, Psychological Assessment Resources, Inc., 16204 North Florida Avenue, Lutz, Florida 33549, from the Frontal Systems Behavior Scale by Janet Grace, PhD and Paul F. Malloy, PhD, Copyright 1992, 2000, 2001 by PAR, Inc. Further reproduction is prohibited without permission of PAR, Inc.
Version 09-Sep-13

Appendix 9.9.2: FrSBe (Self-Rating form) [FRENCH]

Found in [FrSBe (Self-Rating form) (FR, 08Jan2015) - CAPTURE ALS]

CAPTURE-ALS

FrSBe
Formulaire d'auto-évaluation

PARTICIPANT ID:

DATE:

VISIT:

Instructions:
Vous trouverez dans ce questionnaire une série de phrases qui décrivent le comportement d'une personne. Lisez chaque phrase avec attention. En vous référant à la signification des chiffres ci-dessous, entourez le chiffre qui vous semble correspondre à la fréquence avec laquelle votre proche manifeste ce comportement. Essayez si possible de répondre à toutes les questions. Si vous voulez changer votre réponse, faites un X sur le nombre entouré par erreur et entourez le bon nombre.

*Noter le comportement à chaque point en temps - avant la maladie ou blessure et aujourd'hui.

1	2	3	4	5
Presque jamais	Rarement	Parfois	Souvent	Presque tout le temps

	Avant blessure ou maladie	Aujourd'hui
1. Je ne parle que quand on m'adresse la parole	1 2 3 4 5	1 2 3 4 5
2. Je m'agace ou me fâche facilement. Je suis si facilement irritable que je m'emporte sans raison.	1 2 3 4 5	1 2 3 4 5
3. Je répète certaines actions ou me braque sur certaines idées.	1 2 3 4 5	1 2 3 4 5
4. J'agis de façon impulsive.	1 2 3 4 5	1 2 3 4 5
5. Je mélange l'ordre des choses, je me sens désorienté(e) quand il faut faire plusieurs choses à la suite.	1 2 3 4 5	1 2 3 4 5
6. Je ris ou je pleure trop facilement.	1 2 3 4 5	1 2 3 4 5
7. Je fais et refais toujours la même erreur, je n'arrive pas à retenir les expériences passées.	1 2 3 4 5	1 2 3 4 5
8. J'ai du mal à commencer une activité, je manque d'initiative, de motivation.	1 2 3 4 5	1 2 3 4 5
9. Je fais des commentaires et des propositions d'ordre sexuel hors de propos, je suis trop dragueur.	1 2 3 4 5	1 2 3 4 5
10. Je dis ou je fais des choses gênantes.	1 2 3 4 5	1 2 3 4 5
11. Je néglige mon hygiène personnelle.	1 2 3 4 5	1 2 3 4 5
12. Je n'arrive pas à rester tranquille, je suis hyperactif (ve).	1 2 3 4 5	1 2 3 4 5
13. Je ne me rends pas compte de mes problèmes ou de quand je fais une erreur.	1 2 3 4 5	1 2 3 4 5
14. Je reste à ne rien faire.	1 2 3 4 5	1 2 3 4 5
15. Je suis désorganisé(e).	1 2 3 4 5	1 2 3 4 5
16. Je n'arrive pas à contrôler mon urine ou mes selles, mais ça ne me gêne pas.	1 2 3 4 5	1 2 3 4 5
17. Je ne peux pas faire deux choses à la fois (par exemple parler et préparer le repas).	1 2 3 4 5	1 2 3 4 5
18. Je parle quand ce n'est pas à moi de le faire, j'interromps les gens dans les conversations.	1 2 3 4 5	1 2 3 4 5
19. Je n'ai pas beaucoup de discernement, je ne sais pas bien résoudre un problème.	1 2 3 4 5	1 2 3 4 5
20. Je peux inventer des histoires extraordinaires, alors que je suis incapable de me souvenir de quelque chose.	1 2 3 4 5	1 2 3 4 5
21. J'ai perdu l'intérêt pour des choses qui étaient auparavant amusantes ou importantes pour moi.	1 2 3 4 5	1 2 3 4 5

Adapté et reproduit par autorisation spéciale de l'éditeur, Psychological Assessment Resources, Inc., 16204 North Florida Avenue, Lutz, Florida 33549, de l'Echelle comportementale des systèmes frontaux, de Janet Grace, PhD, et Paul F. Malloy, PhD, Copyright 1992, 2000, 2001 by PAR, Inc. Toute autre reproduction est interdite sans la permission de PAR, Inc.

1	2	3	4	5
Presque jamais	Rarement	Parfois	Souvent	Presque tout le temps

	Avant blessure ou maladie					Aujourd'hui				
22. Je dis quelque chose, puis je fais autre chose.	1	2	3	4	5	1	2	3	4	5
23. Je commence des choses, mais je n'arrive pas à les finir, ça tombe à l'eau.	1	2	3	4	5	1	2	3	4	5
24. Je ne manifeste pas beaucoup d'émotions, je ne suis pas concerné(e) ou insensible.	1	2	3	4	5	1	2	3	4	5
25. J'oublie de faire des choses, et puis je m'en souviens quand on me les rappelle ou quand il est trop tard.	1	2	3	4	5	1	2	3	4	5
26. Je suis rigide, je suis incapable de changer mes habitudes.	1	2	3	4	5	1	2	3	4	5
27. J'ai des ennuis avec la loi ou les autorités.	1	2	3	4	5	1	2	3	4	5
28. Je fais des choses dangereuses juste pour m'amuser.	1	2	3	4	5	1	2	3	4	5
29. Je me déplace lentement, je manque d'énergie, je suis inactif (ve).	1	2	3	4	5	1	2	3	4	5
30. Je suis trop bête, j'ai un humour puéril.	1	2	3	4	5	1	2	3	4	5
31. Je trouve que la nourriture n'a pas de goût ni d'odeur.	1	2	3	4	5	1	2	3	4	5
32. Je dis des gros mots.	1	2	3	4	5	1	2	3	4	5

Les propositions suivantes utilisent une nouvelle échelle de cotation. Lisez bien cette échelle, puis chacune des différentes propositions qui suivent avant de répondre.

1	2	3	4	5
Presque tout le temps	Souvent	Parfois	Rarement	Presque jamais

	Avant blessure ou maladie					Aujourd'hui				
33. Je m'excuse de mes écarts de comportement (par exemple, je m'excuse de dire des gros mots).	1	2	3	4	5	1	2	3	4	5
34. Je fais attention, je me concentre, même quand il y a des distractions.	1	2	3	4	5	1	2	3	4	5
35. Je réfléchis avant d'agir (par exemple, je pense au budget avant de dépenser de l'argent).	1	2	3	4	5	1	2	3	4	5
36. J'utilise des stratégies pour me souvenir des choses importantes (par exemple, j'écris des notes pour moi-même).	1	2	3	4	5	1	2	3	4	5
37. Je suis capable de faire des projets.	1	2	3	4	5	1	2	3	4	5
38. Je suis attiré(e) par le sexe.	1	2	3	4	5	1	2	3	4	5
39. Je fais attention à mon aspect extérieur (par exemple, je soigne ma présentation tous les jours)	1	2	3	4	5	1	2	3	4	5
40. Je tire bénéfice des réactions que je provoque, j'accepte les critiques et les réactions des autres.	1	2	3	4	5	1	2	3	4	5
41. Je me lance spontanément dans des activités ou des passe-temps.	1	2	3	4	5	1	2	3	4	5
42. Je fais des choses sans qu'on me le demande.	1	2	3	4	5	1	2	3	4	5
43. Je suis sensible aux besoins d'autrui.	1	2	3	4	5	1	2	3	4	5
44. Je m'entends bien avec les autres gens.	1	2	3	4	5	1	2	3	4	5
45. J'agis conformément à mon âge.	1	2	3	4	5	1	2	3	4	5
46. Je peux facilement engager une conversation.	1	2	3	4	5	1	2	3	4	5

Adapté et reproduit par autorisation spéciale de l'éditeur, Psychological Assessment Resources, Inc., 16204 North Florida Avenue, Lutz, Florida 33549, de l'Echelle comportementale des systèmes frontaux, de Janet Grace, PhD, et Paul F. Malloy, PhD, Copyright 1992, 2000, 2001 by PAR, Inc. Toute autre reproduction est interdite sans la permission de PAR, Inc.

Appendix 9.9.3: FrSBe (Family-Rating form) [ENGLISH]

Found in [FrSBe (Family-Rating form) (EN, 27Sep2016) - CAPTURE ALS]

CAPTURE-ALS
FRONTAL SYSTEMS BEHAVIOUR SCALE (FrSBe)
FAMILY-RATING FORM

PARTICIPANT ID:
DATE:
VISIT:

Instructions:
Inside this form is a list of phrases that can be used to describe a person's behaviour. Please read each phrase carefully. Using the rating scale below, circle the box under each column that corresponds to how often you have engaged in the behaviour described. Rate your family member's behaviour for each point in time - **Before the illness or injury** and **At the present time**. Please try to provide a rating for all of the statements.

1	2	3	4	5
Almost never	Seldom	Sometimes	Frequently	Almost always

Examples:
If during the time *before* your family member's illness or injury he or she *almost never* felt confused, then you would tick the box under the 1 column labeled **Before illness or injury**. If at the *present* time your family member *frequently* feels confused, then you would tick the box under the 4 column labeled **At the present time**. If you need to change an answer after you have already circled it, just mark an X through the incorrect number and then circle the correct number.

	Before illness or injury	At the present time
1. Speaks only when spoken to.	1 2 3 4 5	1 2 3 4 5
**2. Is easily angered or irritated; has emotional outbursts without good reason.**	**1 2 3 4 5**	**1 2 3 4 5**
3. Repeats certain actions or get stuck on certain ideas.	1 2 3 4 5	1 2 3 4 5
**4. Does things impulsively.**	**1 2 3 4 5**	**1 2 3 4 5**
5. Mixes up a sequence, get confused when doing several things in a row.	1 2 3 4 5	1 2 3 4 5
**6. Laughs or cries too easily.**	**1 2 3 4 5**	**1 2 3 4 5**
7. Makes the same mistakes over and over, does not learn from past experience.	1 2 3 4 5	1 2 3 4 5
**8. Has difficulty starting an activity, lack initiatives, motivation.**	**1 2 3 4 5**	**1 2 3 4 5**
9. Makes inappropriate sexual comments and advances, is too flirtatious.	1 2 3 4 5	1 2 3 4 5
**10. Does or says embarrassing things.**	**1 2 3 4 5**	**1 2 3 4 5**
11. Neglects personal hygiene.	1 2 3 4 5	1 2 3 4 5
**12. Can't sit still, is hyperactive.**	**1 2 3 4 5**	**1 2 3 4 5**
13. Denies having problems or is unaware of problems or mistakes.	1 2 3 4 5	1 2 3 4 5
**14. Sits around doing nothing.**	**1 2 3 4 5**	**1 2 3 4 5**
15. Is disorganized.	1 2 3 4 5	1 2 3 4 5
**16. Loses control of urine or bowels and seems unconcerned**	**1 2 3 4 5**	**1 2 3 4 5**
17. Cannot do two things at once (for example, talk and prepare a meal).	1 2 3 4 5	1 2 3 4 5
**18. Talks out of turn, interrupts others in conversations.**	**1 2 3 4 5**	**1 2 3 4 5**
19. Shows poor judgment, is a poor problem solver.	1 2 3 4 5	1 2 3 4 5
**20. Makes up fantastic stories when unable to remember something.**	**1 2 3 4 5**	**1 2 3 4 5**

Adapted and reproduced by special permission of the Publisher, Psychological Assessment Resources, Inc., 16204 North Florida Avenue, Lutz, Florida 33549, from the Frontal Systems Behavior Scale by Janet Grace, PhD and Paul F. Malloy, PhD, Copyright 1992, 2000, 2001 by PAR, Inc. Further reproduction is prohibited without permission of PAR, Inc.

CAPTURE-ALS
FRONTAL SYSTEMS BEHAVIOUR SCALE (FrSBe)
FAMILY-RATING FORM

	Before illness or injury	At the present time
21. Has lost interest in things that used to be fun or important to him/her.	1 2 3 4 5	1 2 3 4 5
**22. Says one thing, then does another thing.**	1 2 3 4 5	1 2 3 4 5
23. Starts things but fails to finish them, "peters out."	1 2 3 4 5	1 2 3 4 5
**24. Shows little emotion, is unconcerned and unresponsive.**	1 2 3 4 5	1 2 3 4 5
25. Forgets to do things but then remembers when prompted or when it is too late.	1 2 3 4 5	1 2 3 4 5
**26. Is inflexible, unable to change routines.**	1 2 3 4 5	1 2 3 4 5
27. Gets in trouble with the law or authorities.	1 2 3 4 5	1 2 3 4 5
**28. Does risky things just for the heck of it.**	1 2 3 4 5	1 2 3 4 5
29. Is slow moving, lacks energy, inactive.	1 2 3 4 5	1 2 3 4 5
**30. Is overly silly, has childish sense of humour.**	1 2 3 4 5	1 2 3 4 5
31. Complains that food has no taste or smell.	1 2 3 4 5	1 2 3 4 5
**32. Swears.**	1 2 3 4 5	1 2 3 4 5

Read each of the following items carefully before responding

1	2	3	4	5
Almost always	Frequently	Sometimes	Seldom	Almost never

	Before illness or injury	At the present time
33. Apologizes for misbehaviour (for example, apologize for swearing).	1 2 3 4 5	1 2 3 4 5
34. **Pays attention, concentrates even when there are distractions.**	1 2 3 4 5	1 2 3 4 5
35. Thinks things through before acting (for example, considers finances before spending money).	1 2 3 4 5	1 2 3 4 5
**36. Uses strategies to remember important things (for example, write notes to self).**	1 2 3 4 5	1 2 3 4 5
37. Is able to plan ahead.	1 2 3 4 5	1 2 3 4 5
**38. Is interested in sex.**	1 2 3 4 5	1 2 3 4 5
39. Cares about his/her appearance (for example, daily grooming).	1 2 3 4 5	1 2 3 4 5
**40. Benefits from feedback, accepts constructive criticism from others.**	1 2 3 4 5	1 2 3 4 5
41. Gets involved with activities spontaneously (such as hobbies).	1 2 3 4 5	1 2 3 4 5
**42. Does things without being requested to do so.**	1 2 3 4 5	1 2 3 4 5
43. Is sensitive to the needs of other people.	1 2 3 4 5	1 2 3 4 5
**44. Gets along well with others.**	1 2 3 4 5	1 2 3 4 5
45. Acts appropriately for his/her age.	1 2 3 4 5	1 2 3 4 5
**46. Can start conversations spontaneously.**	1 2 3 4 5	1 2 3 4 5

Adapted and reproduced by special permission of the Publisher, Psychological Assessment Resources, Inc., 16204 North Florida Avenue, Lutz, Florida 33549, from the Frontal Systems Behavior Scale by Janet Grace, PhD and Paul F. Malloy, PhD, Copyright 1992, 2000, 2001 by PAR, Inc. Further reproduction is prohibited without permission of PAR, Inc.
Version 09-Sep-13

CAPTURE-ALS
FRONTAL SYSTEMS BEHAVIOUR SCALE (FrSBe)
SCORING SHEET

PARTICIPANT ID:
DATE:
VISIT:

SCORING: Transfer each circled item score from the **BEFORE illness or injury** column to the corresponding shaded box in the **BEFORE** column below. Transfer each circled item score from the **At the PRESENT time** column to the corresponding shaded box in the AFTER column below. Sum the scores for each subscale (A, D, and E) and enter the total in the space provided at the bottom of each column. Sum the 2 subtotals on each page to obtain total subscale BEFORE and AFTER scores. Total all BEFORE and all AFTER scores.

	BEFORE			AFTER		
	Apathy	Disinhibition	Executive Dysfunction	Apathy	Disinhibition	Executive Dysfunction
1.						
2.						
3.						
4.						
5.						
6.						
7.						
8.						
9.						
10.						
11.						
12.						
13.						
14.						
15.						
16.						
17.						
18.						
19.						
20.						
21.						
22.						
23.						
24.						
	**Subtotal:**	**Subtotal:**	**Subtotal:**	**Subtotal:**	**Subtotal:**	**Subtotal:**

Adapted and reproduced by special permission of the Publisher, Psychological Assessment Resources, Inc., 16204 North Florida Avenue, Lutz, Florida 33549, from the Frontal Systems Behavior Scale by Janet Grace, PhD and Paul F. Malloy, PhD, Copyright 1992, 2000, 2001 by PAR, Inc. Further reproduction is prohibited without permission of PAR, Inc.
Version 09-Sep-13

	BEFORE			AFTER		
	Apathy	Disinhibition	Executive Dysfunction	Apathy	Disinhibition	Executive Dysfunction
25.						
26.						
27.						
28.						
29.						
30.						
31.						
32.						
33.						
34.						
35.						
36.						
37.						
38.						
39.						
40.						
41.						
42.						
43.						
44.						
45.						
46.						

Subtotals (page 4)						
Subtotals (page 3)						
**Subscale Totals**	*Apathy:*	*Disinhibition:*	*Executive Dys.:*	*Apathy:*	*Disinhibition:*	*Executive Dys:.*
	**Total BEFORE:**			**Total AFTER:**		

Adapted and reproduced by special permission of the Publisher, Psychological Assessment Resources, Inc., 16204 North Florida Avenue, Lutz, Florida 33549, from the Frontal Systems Behavior Scale by Janet Grace, PhD and Paul F. Malloy, PhD, Copyright 1992, 2000, 2001 by PAR, Inc. Further reproduction is prohibited without permission of PAR, Inc.

Appendix 9.9.4: FrSBe (Family-Rating form) [FRENCH]

Found in [FrSBe (Family-Rating form) ((FR, 08Jan2015) - CAPTURE ALS]

CAPTURE-ALS

FrSBe

Formulaire d'évaluation des proches

PARTICIPANT ID:

DATE:

VISIT:

Instructions:
Vous trouverez dans ce questionnaire une série de phrases qui décrivent le comportement d'une personne. Lisez chaque phrase avec attention. En vous référant à la signification des chiffres ci-dessous, entourez le chiffre qui vous semble correspondre à la fréquence avec laquelle votre proche manifeste ce comportement. Essayez si possible de répondre à toutes les questions. Si vous voulez changer votre réponse, faites un X sur le nombre entouré par erreur et entourez le bon nombre.

*Noter le comportement de votre membre de la famille à chaque point en temps - avant la maladie ou blessure et aujourd'hui.

1	2	3	4	5
Presque jamais	Rarement	Parfois	Souvent	Presque tout le temps

	Avant blessure ou maladie					Aujourd'hui				
1. Il parle seulement quand on lui adresse la parole.	1	2	3	4	5	1	2	3	4	5
2. Il est si facilement irritable qu'il s'emporte sans raison.	1	2	3	4	5	1	2	3	4	5
3. Il répète certaines actions ou se braque sur certaines idées.	1	2	3	4	5	1	2	3	4	5
4. Il agit de façon impulsive.	1	2	3	4	5	1	2	3	4	5
5. Il mélange l'ordre des choses, il se sent désorienté quand il faut faire plusieurs choses à la suite.	1	2	3	4	5	1	2	3	4	5
6. Il rit ou il pleure trop facilement.	1	2	3	4	5	1	2	3	4	5
7. Il fait et refait toujours la même erreur, il n'arrive pas à retenir les expériences passées.	1	2	3	4	5	1	2	3	4	5
8. Il a du mal à commencer une activité, il manque d'initiative, de motivation.	1	2	3	4	5	1	2	3	4	5
9. Il fait des commentaires et des propositions d'ordre sexuel hors de propos, il est trop dragueur.	1	2	3	4	5	1	2	3	4	5
10. Il dit ou il fait des choses gênantes.	1	2	3	4	5	1	2	3	4	5
11. Il néglige son hygiène personnelle.	1	2	3	4	5	1	2	3	4	5
12. Il n'arrive pas à rester tranquille, il est hyperactif.	1	2	3	4	5	1	2	3	4	5
13. Il ne se rend pas compte de ses problèmes ou de quand il fait une erreur.	1	2	3	4	5	1	2	3	4	5
14. Il reste à ne rien faire.	1	2	3	4	5	1	2	3	4	5
15. Il est désorganisé.	1	2	3	4	5	1	2	3	4	5
16. Il n'arrive pas à contrôler son urine ou ses selles, et ça n'a pas l'air de le gêner.	1	2	3	4	5	1	2	3	4	5
17. Il ne peut pas faire deux choses à la fois (par exemple parler et préparer le repas).	1	2	3	4	5	1	2	3	4	5
18. Il parle quand ce n'est pas à lui de le faire, il interrompt les gens dans les conversations.	1	2	3	4	5	1	2	3	4	5
19. Il n'a pas beaucoup de discernement, il ne sait pas bien résoudre un problème.	1	2	3	4	5	1	2	3	4	5
20. Il peut inventer des histoires extraordinaires, alors qu'il est incapable de se souvenir de quelque chose.	1	2	3	4	5	1	2	3	4	5

Adapté et reproduit par autorisation spéciale de l'éditeur, Psychological Assessment Resources, Inc., 16204 North Florida Avenue, Lutz, Florida 33549, de l'Echelle comportementale des systèmes frontaux, de Janet Grace, PhD, et Paul F. Malloy, PhD, Copyright 1992, 2000, 2001 by PAR, Inc. Toute autre reproduction est interdite sans la permission de PAR, Inc.

1 Presque jamais	2 Rarement	3 Parfois	4 Souvent	5 Presque tout le temps

21. Il a perdu l'intérêt pour des choses qui étaient auparavant amusantes ou importantes pour lui.	1	2	3	4	5	1	2	3	4	5
22. Il dit quelque chose, puis il fait autre chose.	1	2	3	4	5	1	2	3	4	5
23. Il commence des choses, mais n'arrive pas à les finir, ça tombe à l'eau.	1	2	3	4	5	1	2	3	4	5
24. Il ne manifeste pas beaucoup d'émotions, il n'est pas concerné ou insensible.	1	2	3	4	5	1	2	3	4	5
25. Il oublie de faire des choses, et puis s'en souvient quand on les lui rappelle ou quand il est trop tard.	1	2	3	4	5	1	2	3	4	5
26. Il est rigide, il est incapable de changer ses habitudes.	1	2	3	4	5	1	2	3	4	5
27. Il a des ennuis avec la loi ou les autorités.	1	2	3	4	5	1	2	3	4	5
28. Il fait des choses dangereuses juste pour s'amuser.	1	2	3	4	5	1	2	3	4	5
29. Il se déplace lentement, il manque d'énergie, il est inactif.	1	2	3	4	5	1	2	3	4	5
30. Il est trop bête, il a un humour puéril.	1	2	3	4	5	1	2	3	4	5
31. Il trouve que la nourriture n'a pas de goût ni d'odeur.	1	2	3	4	5	1	2	3	4	5
32. Il dit des gros mots.	1	2	3	4	5	1	2	3	4	5

Les propositions suivantes utilisent une nouvelle échelle de cotation. Lisez bien cette échelle, puis chacune des différentes propositions qui suivent avant de répondre.

1 Presque tout le temps	2 Souvent	3 Parfois	4 Rarement	5 Presque jamais

	Avant blessure ou maladie					Aujourd'hui				
33. Il s'excuse de ses écarts de comportement (par exemple, il s'excuse quand il dit des gros mots).	1	2	3	4	5	1	2	3	4	5
34. Il fait attention, il se concentre, même quand il y a des distractions.	1	2	3	4	5	1	2	3	4	5
35. Il réfléchit avant d'agir (par exemple, il pense au budget avant de dépenser de l'argent).	1	2	3	4	5	1	2	3	4	5
36. Il utilise des stratégies pour se souvenir des choses importantes (par exemple, il écris des notes pour lui-même).	1	2	3	4	5	1	2	3	4	5
37. Il est capable de faire des projets.	1	2	3	4	5	1	2	3	4	5
38. Il est attiré par le sexe.	1	2	3	4	5	1	2	3	4	5
39. Il fait attention à son apparence (par exemple, il soigne sa présentation tous les jours).	1	2	3	4	5	1	2	3	4	5
40. Il tire bénéfice des réactions qu'il provoque, il accepte les critiques et les réactions des autres.	1	2	3	4	5	1	2	3	4	5
41. Il se lance spontanément dans des activités ou des passe-temps.	1	2	3	4	5	1	2	3	4	5
42. Il fait des choses sans qu'on le lui demande.	1	2	3	4	5	1	2	3	4	5
43. Il est sensible aux besoins d'autrui.	1	2	3	4	5	1	2	3	4	5
44. Il s'entend bien avec les autres gens.	1	2	3	4	5	1	2	3	4	5
45. Il agit conformément à son âge.	1	2	3	4	5	1	2	3	4	5
46. Il peut facilement engager une conversation.	1	2	3	4	5	1	2	3	4	5

Adapté et reproduit par autorisation spéciale de l'éditeur, Psychological Assessment Resources, Inc., 16204 North Florida Avenue, Lutz, Florida 33549, de l'Echelle comportementale des systèmes frontaux, de Janet Grace, PhD, et Paul F. Malloy, PhD, Copyright 1992, 2000, 2001 by PAR, Inc. Toute autre reproduction est interdite sans la permission de PAR, Inc.

Appendix 9.10: Stroop Test (Victoria version)

Appendix 9.10.1: Stroop Test (Victoria version) [ENGLISH]
Appendix 9.10.2: Stroop Test (Victoria version) [FRENCH]
Appendix 9.10.3: Stroop Test (Victoria version) Manual [ENGLISH]
Appendix 9.10.4: Stroop Test (Victoria version) stimulus cards [ENGLISH]
Appendix 9.10.5: Stroop Test (Victoria version) stimulus cards [FRENCH]
Appendix 9.10.6: Stroop Test (Victoria version) pointing modification

Appendix 9.10.1: Stroop Test (Victoria version) [ENGLISH]
Found in [Stroop Test (Victoria version) (EN) - CAPTURE ALS]

STROOP TEST:
VICTORIA VERSION

Name: ____________________ Date Tested: _____________ Tested By: ______________

**DOTS**	G	B	Y	R
	Y	R	G	B
	B	G	Y	R
	B	Y	R	G
	R	G	B	Y
	Y	G	B	R
**WORDS**	G	B	Y	R
	Y	R	G	B
	B	G	Y	R
	B	Y	R	G
	R	G	B	Y
	Y	G	B	R
**COLORS**	G	B	Y	R
	Y	R	G	B
	B	G	Y	R
	B	Y	R	G
	R	G	B	Y
	Y	G	B	R

	Time	z	%ile
Dots			
Words			
Colors			

	Errors	z	%ile
Dots			
Words			
Colors			

Unauthorized reproduction forbidden.

Appendix 9.10.2: Stroop Test (Victoria version) [FRENCH]
Found in [Stroop Test (Victoria version) (FR) - CAPTURE ALS]

Stroop Test: Victoria Version

(French Translation)

Name: ____________________ Date Tested: ________________ Tested By: ____________________

DOTS	V	B	J	R
	J	R	V	B
	B	V	J	R
	B	J	R	V
	R	V	B	J
	J	V	B	R
WORDS	V	B	J	R
	J	R	V	B
	B	V	J	R
	B	J	R	V
	R	V	B	J
	J	V	B	R
COLORS	V	B	J	R
	J	R	V	B
	B	V	J	R
	B	J	R	V
	R	V	B	J
	J	V	B	R

	Time	Errors
Dots		
Words		
Colors		

Appendix 9.10.3: Stroop Test (Victoria version) Manual [ENGLISH]
Found in [Stroop Test (Victoria version) Manual (EN) - CAPTURE ALS]

STROOP TEST:
VICTORIA VERSION

Manual of Instructions and Norms

Regard, M. (1981)

Printed and Distributed by
The Test Material Sales Office
Psychology Clinic
Department of Psychology
University of Victoria
British Columbia, Canada

UNAUTHORIZED REPRODUCTION FORBIDDEN

STROOP TEST

Purpose

This test measures the ease with which a person can shift his or her perceptual set to conform to changing demands and suppress a habitual response in favor of an unusual one.

Description

This measure of cognitive flexibility was originally developed by Stroop (1935). His version consists of three white cards, each containing 10 rows of five items. There are four parts to the test. In Part 1, the subject reads randomized color names (blue, green, red, yellow) printed in black type. In Part 2, the subject reads the color names (blue, green, red, yellow) printed in colored ink (blue, green, red, yellow), ignoring the color of the print (the print color never corresponds to the color name). In Part 3, the subject has to name the color of dots (blue, green, red, yellow). In Part 4, the subject is given the card used in Part 2. This time, however, he or she must name the color in which the color names are printed and disregard their verbal content. Of major interest is the subject's behavior when presented with colored words printed in nonmatching colored inks. Stroop reported that normal people can read colored words printed in colored ink as fast as when the words are presented in black ink. However, the time to complete the task increases significantly when the subject is asked to name the color of the ink rather than read the word. This decrease in color-naming speed is called the "color-word interference effect."

Other versions of the Stroop Test have been developed (e.g., Golden, 1976, 1978). The Victoria Version (Regard, 1981) is similar to that devised by Perret (1974). We use this version because of its short administration time and sensitivity to frontal lobe disorders. It consists of three 21.5 x 14 cm cards, each containing six rows of four items (Helvetica, 28 point). The rows are spaced 1 cm apart. In Part D, the subject has to name as quickly as possible the color of 24 dots printed in blue, green, red, or yellow. Each color is used six times, and the four are arranged in a pseudorandom order within the array, each color appearing once in each row. Unlike the original Stroop Test, Part W is similar to Part D, except that the dots are replaced by common words (when, hard, and over), printed in lower case. The subject is required to name the colors in which the stimuli are printed, and to disregard their verbal content. Part C is similar to Parts D and W, but here the colored stimuli are the color names "blue, green, red, and yellow" printed in lower case so that the print color never corresponds to the color name.

Administration

In the Victoria version, the three cards are always presented in the same sequence: D, W, C. Instruct the subject to read or call out the color name as quickly as possible. Start the timer immediately after providing instruction. Instruct as follows:

PART D
"Name the colors of the dots as quickly as you can. Begin here [point], and go across the rows from left to right." Direct the patient's eyes across the rows from left to right.

PART W
"This time, name the colors of the words as quickly as you can. Begin here, and go across the rows from left to right." Clarify if necessary: "**Name the colors in which the words are printed.**"

PART C
"Again, name the colors in which the words are printed, as quickly as you can." Clarify if necessary: **"Don't *read* the word, tell me the color in which the word is printed."**

The errors in color naming are corrected immediately, if not spontaneously corrected by the patient. Then instruct the subject to go on as rapidly as possible.

Approximate Time for Administration

The approximate time required is five minutes.

Scoring

For each part, record both the time to complete and the number of errors. Score spontaneous corrections as correct.

Comment

We have looked at test-retest reliability, using a one-month interval between test sessions. We found reliability estimates of .90, .83, and .91 for the three parts of the test. However, experience with the test does affect performance. Normal college students showed significant practice effects ($p<.001$). On the second administration, performance improved by 2 points on Parts D and W, and by about five seconds on Part C.

The Stroop Test has been studied in psychiatric and brain-damaged patients. The test is fairly effective in distinguishing between normal controls and brain-damaged patients and between psychiatric and brain-damaged samples (Golden, 1976). It also appears to be sensitive to the severity of dementia (Koss et al., 1984). There is evidence that impairment on our version of the Stroop is related to the location of the cerebral lesion. Both Perret (1974) and Regard (1981) reported that the interference effect on Part C (relative to Part W) was greater for patients with left frontal lobe damage than for other patient or control groups.

Normative Data

Both age and intellectual level may contribute to performance on the Stroop Test (Comalli, 1965; Comalli et al., 1962; Das, 1970; Regard, 1981). In adults, aging appears to be linked to slowing in color naming and an increase in the Stroop interference effect.

Regard (1981) presented normative information for the Victoria version (see Table 3-16). The values are derived from a sample of young adults with average intellectual abilities. Recently, we compiled norms for use with older adults. The data are based on a relatively well-educated sample (n = 86; mean education = 13.2 years; SD = 3.1) of healthy elderly people, aged 50-94 (mean = 68.5; SD = 10.78). These data are also given in Table 3-16. Unfortunately data for children are not yet available.

Table 3-16. Modified Stroop Test: Mean Reading Time (in Seconds) and Errors for the 24-Item/Card Form

		Age (years)				
		20-35	50-59	60-69	70-79	80+
N		40	19	28	24	15
Name color of Dots ("D")						
Seconds	M	10.10	13.74	12.71	15.00	18.87
	SD	2.01	2.58	1.90	5.07	4.67
Errors	M	0.03	--	--	0.08	0.20
	SD	0.16	--	--	0.28	0.56
Name color print of noncolor words ("W")						
Seconds	M	12.00	16.58	16.32	19.04	24.13
	SD	2.49	3.34	3.33	5.10	5.13
Errors	M	0.03	--	0.04	--	0.13
	SD	0.16	--	0.19	--	0.35
Name color print of color words ("C)"						
Seconds	M	19.25	28.90	31.82	38.83	61.13
	SD	5.18	7.62	9.86	13.29	30.94
Errors	M	0.23	0.42	0.36	0.71	2.73
	SD	0.53	0.77	0.68	1.16	2.46

Note: Regard (1981) tested 40 right-handed normal people, mean age 26.7 years (range 20-35 years). We gathered data in 1989 from 86 healthy older adults, aged 50-94 years (mean - 68.5).

Sources: Regard (1981); Spreen & Strauss (unpublished data).

References

Comalli, P.E. (1965). Cognitive functioning in a group of 80-90 year-old men. *Journal of Gerontology, 20,* 14-17.

Comalli, P.E., Jr., Wapner, S. & Werner, H. (1962). Interference effects of Stroop Color-Word Test in childhood, adulthood and aging. *Journal of Genetic Psychology, 100,* 47-53.

Das, J.P. (1970). Changes in Stroop-Test responses as a function of mental age. *British Journal of Clinical and Social Psychology, 9,* 68-73.

Golden, J.C. (1976). Identification of brain disorders by the Stroop Color and Word Test. *Journal of Clinical Psychology, 32,* 654-658.

Golden, J.C. (1978). *Stroop Color and Word Test.* Chicago, IL: Stoelting.

Koss, E., Ober, B.A., Delis, D.C. & Friedland, R.P. (1984). The Stroop Color-Word Test: Indicator of dementia severity. *International Journal of Neuroscience, 24,* 53-61.

Perret, E. (1974). The left frontal lobe of man and the suppression of habitual responses in verbal categorical behavior. *Neuropsychologia, 12,* 323-330.

Regard, M. (1981). Cognitive rigidity and flexibility: A neuropsychological study. Unpublished Ph.D. dissertation. University of Victoria, British Columbia.

Stroop, J.R. (1935). Studies of interference in serial verbal reaction. Journal of *Experimental Psychology, 18,* 643-662.

Appendix 9.10.4: Stroop Test (Victoria version) stimulus cards [ENGLISH]

Found in [Stroop Test (Victoria version) stimulus cards (EN) - CAPTURE ALS]

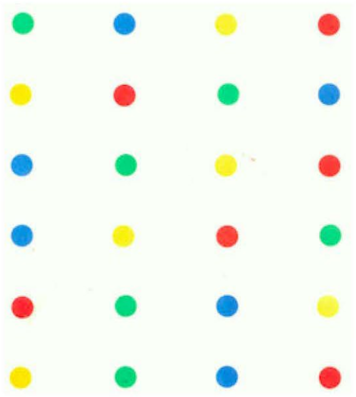

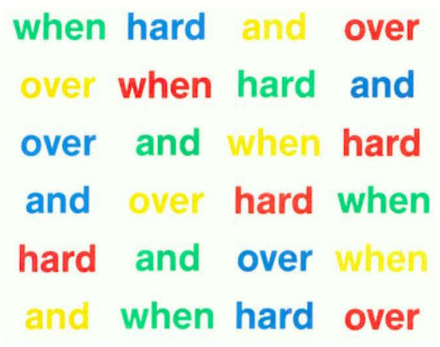

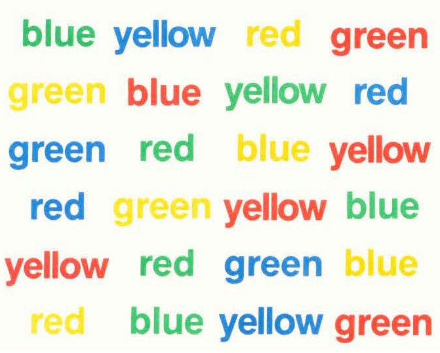

Appendix 9.10.5: Stroop Test (Victoria version) stimulus cards [FRENCH]

Found in [Stroop Test (Victoria version) stimulus cards (FR) - CAPTURE ALS]

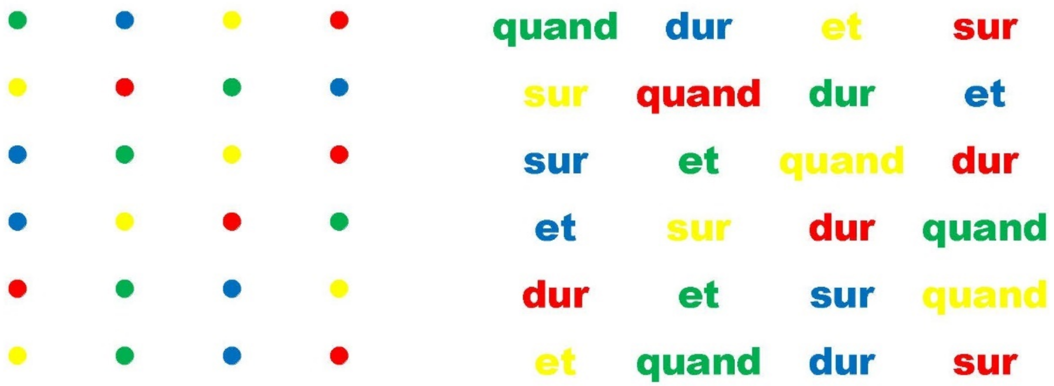

bleu	jaune	rouge	vert
vert	bleu	jaune	rouge
vert	rouge	bleu	jaune
rouge	vert	jaune	bleu
jaune	rouge	vert	bleu
rouge	bleu	jaune	vert

Appendix 9.10.6: Stroop Test (Victoria version) pointing modification

Found in [Stroop Test (Victoria version) pointing modification - CAPTURE ALS]

Victoria Stroop test (pointing modification)

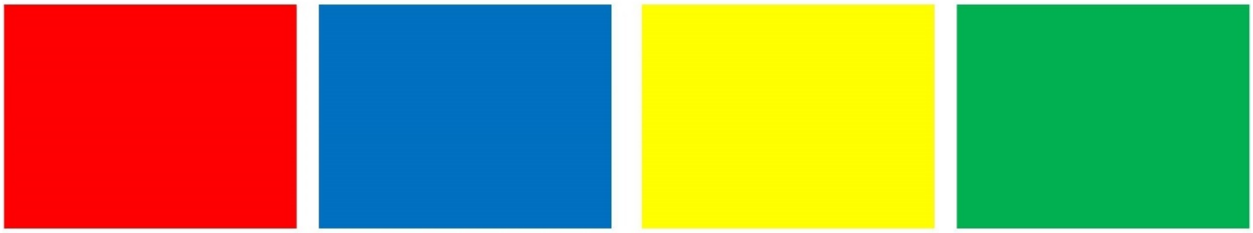

Appendix 9.11: Judgment of Line Orientation Form (JLO)

Appendix 9.11.1: JLO example [ENGLISH]
Appendix 9.11.2: JLO Form V&H [ENGLISH]
Appendix 9.11.3: JLO instructions [ENGLISH]
Appendix 9.11.4: JLO instructions [FRENCH]

Appendix 9.11.1: JLO example [ENGLISH]

Found in [JLO example (EN) – CAPTURE ALS]

An example of an item from the Judgement of Line Orientation Test (Benton, Hamsher, Varney, & Spreen, 1983)

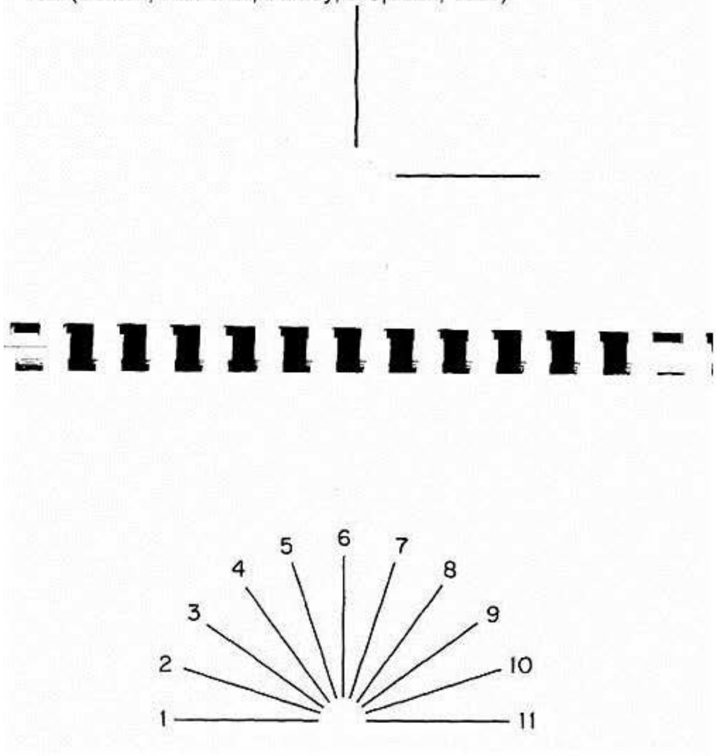

Appendix 9.11.2: JLO Form V&H [ENGLISH]

Found in [JLO Form V&H (EN, 04Sep2013) – CAPTURE ALS]

CAPTURE-ALS
Benton Judgement of Line Orientation (JLO)

only administered if ECAS Visuospatial score ≤10

PARTICIPANT ID: ______
DATE: ______
VISIT: ______

FORM V (see separate instruction sheet)

Practice Items

A__________ 1-6 B__________ 4-8 C __________ 4-10 D __________ 7-8 E __________2-4

Test Items

1.__________	5-10	11.__________	1-7	21.__________	6-10
2.__________	2-11	12.__________	2-6	22.__________	2-9
3.__________	1-2	13.__________	7-9	23.__________	3-8
4.__________	1-7	14.__________	2-5	24.__________	9-11
5.__________	6-7	15.__________	1-9	25.__________	3-4
6.__________	5-6	16.__________	7-8	26.__________	8-9
7.__________	4-5	17.__________	3-5	27.__________	8-11
8.__________	1-3	18.__________	10-11	28.__________	7-10
9.__________	5-11	19.__________	1-4	29.__________	3-10
10.__________	1-10	20.__________	3-11	30.__________	5-8

Correct ______

FORM H (see separate instruction sheet)

DATE: ______
VISIT: ______

Practice Items

A__________ 1-6 B__________ 4-8 C __________ 4-10 D __________ 7-8 E __________2-4

Test Items

1.__________	5-10	11.__________	5-11	21.__________	9-11
2.__________	5-6	12.__________	4-5	22.__________	6-10
3.__________	6-7	13.__________	7-8	23.__________	3-11
4.__________	1-2	14.__________	2-6	24.__________	8-9
5.__________	2-11	15.__________	3-5	25.__________	3-8
6.__________	1-7	16.__________	10-11	26.__________	7-10
7.__________	1-10	17.__________	2-5	27.__________	3-4
8.__________	1-7	18.__________	1-4	28.__________	3-10
9.__________	7-9	19.__________	1-9	29.__________	5-8
10.__________	1-3	20.__________	2-9	30.__________	8-11

Correct ______

Adapted and reproduced by special permission of the Publisher, Psychological Assessment Resources, Inc., 16204 North Florida Avenue, Lutz, Florida 33549, from the Judgment of Line Orientation by Arthur L. Benton, Ph.D., Copyright 1983 by PAR, Inc. Further reproduction is prohibited without permission of PAR, Inc.
Version 04-Sep-13

Appendix 9.11.3: JLO instructions [ENGLISH]
Found in [JLO instructions (EN, 04Sep2013) – CAPTURE ALS]

CAPTURE-ALS
BENTON JUDGEMENT OF LINE ORIENTATION (JLO)

****ADMINISTRATION AND SCORE SHEET****

ADMINISTRATION

- Place the test booklet on a table in front of the patient with the booklet opened so that the stimulus items in the upper ½ are positioned at an angle of about 45° with respect to the surface of the table
- The subject is allowed to hold and position the test booklet to their best advantage.
- Begin with Practice Item A, point to the lines on the upper stimulus page, and say:

  "See these 2 lines? Which 2 lines down here [*pointing to the response card*] **are in exactly the same position and point in the same direction as the 2 lines up here? Tell me the number of the lines."**
  *If incorrect say: **"It's not #--, what would you say it is?"** If correct answers are given, say: **"That's right,"** and proceed with next Practice Item.

- After the 5 Practice Items have been presented, and with the test booklet opened to the page labeled "Test Items," say to the patient:

  "Now we are going to do more of these, except now the lines which you see up here [pointing to the upper page] **will be shorter, because part of the line has been erased. Tell me (or show me) which 2 lines her are pointing in the same direction as the lines up here."**

- Do not tell the subject how they are doing on any test items. However, general statements can be made to provide encouragement and sustain motivation.
- If, after 30 seconds a patient hasn't given a response, they should be encouraged to make their best guess regardless of how uncertain they are about it.
- There is no time limit for responding. Record the actual response choice on the scoring form.

ADMINISTRATION – SUBJECT DIFFICULTIES

- If the patient does not understand the task on Practice Item A, continue by using your hand to cover the line in position 6 and point to the other line (in position 1) and say:

  "Let us just look at this line. Which line down here [pointing to the response-choice display] points in the same direction as this one [pointing to the stimulus line] and is also in the same position?"

- Record the response on the record sheet where it is labeled A'. Correct the patient if they still give the wrong answer. Then proceed using these extended instructions, by covering the other line (in position 1) and pointing to the line in position 6. After demonstrating the line in position 6, again supply the correct answer if it's not given on the 2nd trial (A'). Continue with Practice Item B. If the patient again gives the wrong answers for this practice item, follow up with the extended instructions using single lines (trial B').

- Continue with this cycle of instructions until the patient gives 2 correct responses on the Practice Items on the 1st trial. A correct response means identifying both response choices for the pair of stimulus lines. If this criterion is not met with at least 2 Practice Items, discontinue the test.

SCORING

- Circle errors.
- Tally the number of completely correct items and record it (max score = 30).

Adapted and reproduced by special permission of the Publisher, Psychological Assessment Resources, Inc., 16204 North Florida Avenue, Lutz, Florida 33549, from the Judgment of Line Orientation by Arthur L. Benton, Ph.D., Copyright 1983 by PAR, Inc. Further reproduction is prohibited without permission of PAR, Inc.
Version 04-Sep-13

Appendix 9.11.4: JLO instructions [FRENCH]
Found in [JLO instructions (FR, 06Jan2022) – CAPTURE ALS]

CAPTURE-ALS
Benton Judgment of Line Orientation (JLO)
French Instructions

- Le carnet du test demeure dans le bureau de MJG.

ADMINISTRATION

- Placer le carnet à plat sur la table devant le patient, avec la demie du haut positionnée dans un angle d'environ 45° par rapport à la table.

- Les patients peuvent tenir et positionner le carnet s'il le désire.

- Commencer avec l'item de pratique A : indiquer les deux lignes de la page du haut et dire :

« Vous voyez ces 2 lignes ? Lesquelles des 2 lignes ici (montrer) **sont exactement dans la même position et indiquent la même direction que les 2 lignes ici** (montrer) **? Dites-moi le numéro des 2 lignes ».**

*Si incorrect, dire : **« Ce n'est pas le numéro ___, voyez-vous une autre réponse ? »** Corriger le patient avec l'illustration (voir les plus amples consignes dans le manuel de JOL) si les exemples ne sont pas réussis.
Si la réponse est correcte, dire : **« C'est exact »** et continuer avec l'item suivant.

Une bonne réponse signifie que le patient a identifié les 2 orientations des lignes correctement. Si ce critère n'est pas rencontré avec au moins 2 items de pratique, discontinuer le test.

- Après les items de pratique, avec le livre ouvert à la page où est indiqué « Test items », dites :

« Maintenant, nous allons en faire encore plusieurs sauf que les lignes que vous voyez en haut (indiquer la page du haut) **vont être plus courtes, parce qu'une partie des lignes a été effacée. Dites-moi** (ou montrez-moi) **quelles 2 lignes indiquent la même direction que les lignes du haut ».**

- Ne dites pas au sujet comment il s'en sort dans les épreuves du test. Cependant, des déclarations générales peuvent être faites pour l'encourager et soutenir sa motivation.

- Si, au bout de 30 secondes, le patient n'a pas donné de réponse, il faut l'encourager à faire sa meilleure supposition, même s'il n'est pas certain de la réponse.

- Il n'y a pas de limite de temps pour répondre. Inscrivez le choix de réponse réel sur le formulaire de notation.

Appendix 9.12: Amyotrophic Lateral Sclerosis Assessment Questionnaire Short Form (ALSAQ-5)

Appendix 9.12.1: ALSAQ-5 [ENGLISH]
Appendix 9.12.2: ALSAQ-5 [FRENCH]

Appendix 9.12.1: ALSAQ-5 [ENGLISH]
Found in [ALSAQ-5 (EN, V1.0, 2000) – CAPTURE ALS]

ALSAQ-5

Please complete this questionnaire as soon as possible. If you have any difficulties filling in the questionnaire by yourself, please get someone else to help you with it. However, it is **your** responses that we are interested in.

The questionnaire consists of a number of statements about difficulties that you may have experienced **during the last 2 weeks**. There are no right or wrong answers; your first response is likely to be the most accurate for you. **Please check the box that best describes your own experience or feelings.**

Please try to answer every question, even though some may seem similar to others or may not seem relevant to you.

All the information you give will be kept **strictly confidential**.

The following statements all refer to difficulties that you may have had **during the last 2 weeks**.
Please indicate, by checking the appropriate box, how often the following statements have been true for you.

ALSAQ-5 © Oxford University Innovation Limited, 2000. All rights reserved. Translated to English (Canada) by PharmaQuest Ltd.

The following statements all refer to certain difficulties that you may have had <u>during the last 2 weeks</u>. Please indicate, by checking the appropriate box, how often the following statements have been true for you.

If you cannot do the activity at all, please check **Always or cannot do at all**.

How often <u>during the last 2 weeks</u> have the following been true?

Please check ***one box*** *for each question.*

	Never	Rarely	Some-times	Often	Always or cannot do at all
**1. I have found it difficult to stand up.**	☐	☐	☐	☐	☐
**2. I have had difficulty using my arms and hands.**	☐	☐	☐	☐	☐
**3. I have had difficulty eating solid food.**	☐	☐	☐	☐	☐
**4. I have felt that my speech was not easy to understand.**	☐	☐	☐	☐	☐
**5. I have felt hopeless about the future.**	☐	☐	☐	☐	☐

Please make sure that you have checked ***one box for each question.***

Thank you for completing this questionnaire.

ALSAQ-5 Version 1.00 © 2000 Isis Innovation Ltd

ALSAQ-5 © Oxford University Innovation Limited, 2000. All rights reserved. Translated to English (Canada) by PharmaQuest Ltd.

Appendix 9.12.2: ALSAQ-5 [FRENCH]
Found in [ALSAQ-5 (FR, V1.0, 2000) – CAPTURE ALS]

ALSAQ-5

Veuillez remplir ce questionnaire aussitôt que possible. Si vous avez de la difficulté à répondre seul(e) à ce questionnaire, veuillez demander à quelqu'un de vous aider. Cependant, ce sont **vos** réponses qui nous intéressent.

Le questionnaire comporte un certain nombre de déclarations sur les difficultés que vous avez pu éprouver **au cours des deux dernières semaines**. Il n'y a pas de bonnes ou de mauvaises réponses : la réponse qui vous vient d'abord à l'esprit sera probablement la meilleure pour vous. **Veuillez cocher la case qui correspond le mieux à votre vécu ou à vos sensations et vos émotions.**

Essayez de répondre à chaque question même si certaines d'entre elles ont l'air de se ressembler ou bien ne semblent pas correspondre à votre cas.

Toutes les informations que vous fournirez seront traitées de façon **strictement confidentielle**.

Les déclarations suivantes font toutes référence aux difficultés que vous avez pu rencontrer **au cours des deux dernières semaines**. Veuillez indiquer la fréquence à laquelle chacune des déclarations suivantes a été vraie dans votre cas en cochant la case appropriée.

ALSAQ-5 © Oxford University Innovation Limited, 2000. All rights reserved. Translated to French (Canada) by PharmaQuest Ltd.

Toutes les déclarations suivantes font référence à certaines difficultés que vous avez pu rencontrer au cours des deux dernières semaines. Veuillez indiquer la fréquence à laquelle chacune des déclarations suivantes a été vraie dans votre cas en cochant la case appropriée.

Si vous ne pouvez pas marcher du tout, veuillez cocher ***Toujours ou ne peux faire du tout l'activité.***

Au cours des deux dernières semaines, à quelle fréquence les déclarations suivantes ont-elles été vraies?

Veuillez cocher ***une case*** *par déclaration.*

	**Jamais**	**Rarement**	**Parfois**	**Souvent**	**Toujours ou ne peux faire du tout l'activité**
**1. J'ai eu de la difficulté à me mettre debout.**	☐	☐	☐	☐	☐
**2. J'ai eu de la difficulté à utiliser mes bras et mes mains.**	☐	☐	☐	☐	☐
**3. J'ai eu de la difficulté à manger de la nourriture solide.**	☐	☐	☐	☐	☐
**4. J'ai senti que mes paroles n'étaient pas comprises facilement.**	☐	☐	☐	☐	☐
**5. Je me suis senti(e) sans espoir quant à l'avenir.**	☐	☐	☐	☐	☐

Veuillez vous assurer d'avoir coché ***une case pour chaque question.***

Merci d'avoir répondu à ce questionnaire.

ALSAQ-5 Version 1.00 © 2000 Isis Innovation Ltd

ALSAQ-5 © Oxford University Innovation Limited, 2000. All rights reserved. Translated to French (Canada) by PharmaQuest Ltd.

Appendix 9.13: World Health Organization Quality of Life-BREF Scale (WHOQOL-BREF)

Appendix 9.13.1: WHOQOL-BREF [ENGLISH]
Appendix 9.13.2: WHOQOL-BREF [FRENCH]

Appendix 9.13.1: WHOQOL-BREF [ENGLISH]
Found in [WHOQOL-BREF (EN, Dec1996) – CAPTURE ALS]

WHO/MSA/MNH/PSF/97.4
English only
Distr.: Limited

WHOQOL-BREF

PROGRAMME ON MENTAL HEALTH
WORLD HEALTH ORGANIZATION
GENEVA

For office use only

	Equations for computing domain scores	Raw score	Transformed scores*	
**Domain 1**	(6-Q3) + (6-Q4) + Q10 + Q15 + Q16 + Q17 + Q18<br>☐ + ☐ + ☐ + ☐ + ☐ + ☐ + ☐	=	**4-20**	**0-100**
**Domain 2**	Q5 + Q6 + Q7 + Q11 + Q19 + (6-Q26)<br>☐ + ☐ + ☐ + ☐ + ☐ + ☐	=		
**Domain 3**	Q20 + Q21 + Q22<br>☐ + ☐ + ☐	=		
**Domain 4**	Q8 + Q9 + Q12 + Q13 + Q14 + Q23 + Q24 + Q25<br>☐ + ☐ + ☐ + ☐ + ☐ + ☐ + ☐ + ☐	=		

* Please see Table 4 on page 10 of the manual, for converting raw scores to transformed scores.

This document is not issued to the general public, and all rights are reserved by the World Health Organization (WHO). The document may not be reviewed, abstracted, quoted, reproduced or translated, in part or in whole, without the prior written permission of WHO. No part of this document may be stored in a retrieval system or transmitted in any form or by any means - electronic, mechanical or other - without the prior written permission of WHO.

MSA/MNH/PSF/97.6
Page 16

I.D. number

ABOUT YOU

Before you begin we would like to ask you to answer a few general questions about yourself: by circling the correct answer or by filling in the space provided.

What is your **gender**? Male Female

What is you **date of birth**? ________ / ________ / ________
Day / Month / Year

What is the highest **education** you received?
None at all
Primary school
Secondary school
Tertiary

What is your **marital status**?
Single
Married
Living as married
Separated
Divorced
Widowed

Are you currently **ill**? Yes No

If something is wrong with your health what do you think it is?___________________________illness/ problem

Instructions

This assessment asks how you feel about your quality of life, health, or other areas of your life. **Please answer all the questions.** If you are unsure about which response to give to a question, **please choose the one** that appears most appropriate. This can often be your first response.

Please keep in mind your standards, hopes, pleasures and concerns. We ask that you think about your life **in the last two weeks.** For example, thinking about the last two weeks, a question might ask:

		Not at all	Not much	Moderately	A great deal	Completely
	Do you get the kind of support from others that you need?	1	2	3	4	5

You should circle the number that best fits how much support you got from others over the last two weeks. So you would circle the number 4 if you got a great deal of support from others as follows.

		Not at all	Not much	Moderately	A great deal	Completely
	Do you get the kind of support from others that you need?	1	2	3	4	5

You would circle number 1 if you did not get any of the support that you needed from others in the last two weeks.

Please read each question, assess your feelings, and circle the number on the scale for each question that gives the best answer for you.

		Very poor	Poor	Neither poor nor good	Good	Very good
1(G1)	How would you rate your quality of life?	1	2	3	4	5

		Very dissatisfied	Dissatisfied	Neither satisfied nor dissatisfied	Satisfied	Very satisfied
2 (G4)	How satisfied are you with your health?	1	2	3	4	5

The following questions ask about **how much** you have experienced certain things in the last two weeks.

		Not at all	A little	A moderate amount	Very much	An extreme amount
3 (F1.4)	To what extent do you feel that physical pain prevents you from doing what you need to do?	1	2	3	4	5
4(F11.3)	How much do you need any medical treatment to function in your daily life?	1	2	3	4	5
5(F4.1)	How much do you enjoy life?	1	2	3	4	5
6(F24.2)	To what extent do you feel your life to be meaningful?	1	2	3	4	5

		Not at all	A little	A moderate amount	Very much	Extremely
7(F5.3)	How well are you able to concentrate?	1	2	3	4	5
8 (F16.1)	How safe do you feel in your daily life?	1	2	3	4	5
9 (F22.1)	How healthy is your physical environment?	1	2	3	4	5

The following questions ask about **how completely** you experience or were able to do certain things in the last two weeks.

		Not at all	A little	Moderately	Mostly	Completely
10 (F2.1)	Do you have enough energy for everyday life?	1	2	3	4	5
11 (F7.1)	Are you able to accept your bodily appearance?	1	2	3	4	5
12 (F18.1)	Have you enough money to meet your needs?	1	2	3	4	5
13 (F20.1)	How available to you is the information that you need in your day-to-day life?	1	2	3	4	5
14 (F21.1)	To what extent do you have the opportunity for leisure activities?	1	2	3	4	5

		Very poor	Poor	Neither	Good	Very good

				poor nor good		
15 (F9.1)	How well are you able to get around?	1	2	3	4	5

The following questions ask you to say how **good or satisfied** you have felt about various aspects of your life over the last two weeks.

		Very dissatisfied	Dissatisfied	Neither satisfied nor dissatisfied	Satisfied	Very satisfied
16 (F3.3)	How satisfied are you with your sleep?	1	2	3	4	5
17 (F10.3)	How satisfied are you with your ability to perform your daily living activities?	1	2	3	4	5
18(F12.4)	How satisfied are you with your capacity for work?	1	2	3	4	5
19 (F6.3)	How satisfied are you with yourself?	1	2	3	4	5
20(F13.3)	How satisfied are you with your personal relationships?	1	2	3	4	5
21(F15.3)	How satisfied are you with your sex life?	1	2	3	4	5
22(F14.4)	How satisfied are you with the support you get from your friends?	1	2	3	4	5
23(F17.3)	How satisfied are you with the conditions of your living place?	1	2	3	4	5
24(F19.3)	How satisfied are you with your access to health services?	1	2	3	4	5
25(F23.3)	How satisfied are you with your transport?	1	2	3	4	5

The following question refers to **how often** you have felt or experienced certain things in the last two weeks.

		Never	Seldom	Quite often	Very often	Always
26 (F8.1)	How often do you have negative feelings such as blue mood, despair, anxiety, depression?	1	2	3	4	5

Did someone help you to fill out this form?..

How long did it take to fill this form out?..

Do you have any comments about the assessment?

....

....

THANK YOU FOR YOUR HELP

Appendix 9.13.2: WHOQOL-BREF [FRENCH]
Found in [WHOQOL-BREF (FR, Dec1996) – CAPTURE ALS]

WHOQOL-Bref (vf. O.2)
Page 1

WHO QOL - Bref

Version test
Avril 1997

PROGRAMME SUR LA SANTE MENTALE
ORGANISATION MONDIALE DE LA SANTE
GENEVE

Information sur le calcul des scores

	Equations pour calculer les scores par domaine	score bruts	scores de 4 à 20	scores de 0 à 100
Domaine 1	(6-Q3) + (6-Q4) + Q10 + Q15 + Q16 + Q17 +Q18<br>☐ + ☐ + ☐ + ☐ + ☐ +☐ + ☐			
Domaine 2	Q5 + Q6 + Q7 + Q11 + Q19 + (6 - Q26)<br>☐ + ☐ + ☐ + ☐ + ☐ + ☐			
Domaine 3	Q20 + Q21 + Q22<br>☐ + ☐ + ☐			
Domaine 4	Q8 + Q9 + Q12 + Q13 + Q14 + Q23 + Q24 + Q25<br>☐ +☐ + ☐ + ☐ + ☐ +☐ + ☐ + ☐			

WHOQOL - 26 (vf. O.2)
Page 2

		Très mauvaise	Mauvaise	Ni bonne, ni mauvaise	Bonne	Très bonne
1 (G1)	Comment trouvez-vous votre qualité de vie ?	1	2	3	4	5
		Pas du tout satisfait	Pas satisfait	Ni satisfait ni insatisfait	Satisfait	Très satisfait
2 (G4)	Etes-vous satisfait de votre santé ?	1	2	3	4	5
		Pas du tout	Un peu	Modérément	Beaucoup	Complètement
3 (F1.4)	La douleur (physique) vous empêche-t-elle de faire ce que vous avez à faire ?	1	2	3	4	5
4 (F11.3)	Un traitement médical vous est-il nécessaire pour faire face à la vie de tous les jours ?	1	2	3	4	5
5 (F4.1)	Trouvez-vous la vie agréable ?	1	2	3	4	5
6 (F24.2)	Vos croyances personnelles donnent-elles un sens à votre vie ?	1	2	3	4	5
		Pas du tout	Un peu	Modérément	Beaucoup	Tout à fait
7 (5.3)	Etes-vous capable de vous concentrer ?	1	2	3	4	5
8 (F16.1)	Vous sentez vous en sécurité dans votre vie de tous les jours ?	1	2	3	4	5
9 (F22.1)	Votre environnement est-il sain (pollution, bruit, salubrité, etc.) ?	1	2	3	4	5
		Pas du tout	Un peu	Modérément	Suffisamment	Tout à fait
10 (F2.1)	Avez-vous assez d'énergie dans la vie de tous les jours ?	1	2	3	4	5
11 (F7.1)	Acceptez-vous votre apparence physique ?	1	2	3	4	5
12 (F18.1)	Avez-vous assez d'argent pour satisfaire vos besoins ?	1	2	3	4	5
13 (F20.1)	Avez vous le sentiment d'être assez informé pour faire face à la vie de tous les jours ?	1	2	3	4	5
14 (F21.1)	Avez-vous la possibilité d'avoir des activités de loisirs ?	1	2	3	4	5

WHOQOL - 26 (vf. O.2)
Page 3

		Très mauvaise	Mauvaise	Ni bonne, ni mauvaise	Bonne	Très bonne
15 (F9.1)	Comment trouvez-vous votre capacité à vous déplacer seul ?	1	2	3	4	5
		Très insatisfait	Insatisfait	Ni satisfait ni insatisfait	Satisfait	Très satisfait
16 (F3.3)	Etes-vous satisfait de votre sommeil ?	1	2	3	4	5
17 (F10.3)	Etes-vous satisfait de votre capacité à accomplir vos activités quotidiennes ?	1	2	3	4	5
18 (F12.4)	Etes-vous satisfait de votre capacité à travailler ?	1	2	3	4	5
		Pas du tout	Un peu	Modérément	Beaucoup	Extrêmement
19 (F6.3)	Avez-vous une bonne opinion de vous-même ?	1	2	3	4	5
		Pas du tout satisfait	Pas satisfait	Ni satisfait ni insatisfait	Satisfait	Très satisfait
20 (F13.3)	Etes-vous satisfait de vos relations personnelles ?	1	2	3	4	5
21 (F15.3)	Etes-vous satisfait de votre vie sexuelle ?	1	2	3	4	5
22 (F14.4)	Etes-vous satisfait du soutien que vous recevez de vos amis ?	1	2	3	4	5
23 (F17.3)	Etes-vous satisfait de l'endroit où vous vivez ?	1	2	3	4	5
		Pas du tout	Un peu	Modérément	Beaucoup	Tout à fait
24 (F19.3)	Avez vous facilement accès aux soins dont vous avez besoin ?	1	2	3	4	5
		Pas du tout satisfait	Pas satisfait	Ni satisfait ni insatisfait	Satisfait	Très satisfait
25 (F23.3)	Etes-vous satisfait de vos moyens de transport ?	1	2	3	4	5
		Jamais	Parfois	Souvent	Très souvent	Toujours
26 (F8.1)	Eprouvez-vous souvent des sentiments négatifs comme le cafard, le désespoir, l'anxiété ou la dépression ?	1	2	3	4	5

This translation was not created by the World Health Organization (WHO). WHO is not responsible for the content or accuracy of this translation. In the event of any inconsistency between the English and the translated version, the original English version shall be the binding and authentic version.

Appendix 9.14: Neurocognitive Evaluations Form

Appendix 9.14.1: Neurocognitive Evaluations Form (SCREENING) [ENGLISH]
Appendix 9.14.2: Neurocognitive Evaluations Form (Month 0 and 8) [ENGLISH]
Appendix 9.14.3: Neurocognitive Evaluations Form (Month 4 and 12) [ENGLISH]
Appendix 9.14.4: Neurocognitive Evaluations Form (Caregiver) (Month 0 and 8) [ENGLISH]
Appendix 9.14.5: Neurocognitive Evaluations Form (Caregiver) (Month 4 and 12) [ENGLISH]

Appendix 9.14.1: Neurocognitive Evaluations Form (SCREENING) [ENGLISH]
Found in [Neurocognitive Evaluations Form (SCREENING) (EN, V1, 03-JAN-2022) CAPTURE ALS]

Neurocognitive Evaluations Form
(Screening Visit)

Montreal Cognitive Assessment (MoCA)			NOT DONE ☐
Spoken ☐ OR Written ☐			
**Visuospatial/Executive score (/5):**	**Modification drawings used?**	Yes ☐ No ☐	
**Naming score (/3):**			
**Attention score (/6):**			
**Language score (/3):**			
**Abstraction score (/2):**			
**Delayed recall score (/5):**			
**Orientation score (/6):**			
**Total score (/30) (+1 point if years of education is ≤12):**			

Appendix 9.14.2: Neurocognitive Evaluations Form (Month 0 and 8) [ENGLISH]

Found in [Neurocognitive Evaluations Form (Month 0 and 8) (EN, V1, 03-JAN-2022) CAPTURE ALS]

Neurocognitive Evaluations Form
Visit 2 (month-0) and Visit 4 (month-8)

**Edinburgh Cognitive and Behavioral ALS Screen (ECAS)**	NOT DONE ☐
**Date:**	Spoken ☐ **OR** Written ☐
**Test version:** A ☐ B ☐ C ☐	
**Language:** Naming (/8): Comprehension (/8): Spelling (/12):	
**Verbal Fluency:** First Letter (S/F/P) (/12): Second Letter (T/D/M) (/12):	
**Executive:** Reverse Digit Span (/12): Alternation (/12): Sentence Completion (/12): Social Cognition (/12):	
**Memory:** Immediate recall (/10): Delayed retention (/10): Delayed recognition (/4):	
**Visuospatial:** Dot Counting (/4): Cube Counting (/4): Number Location (/4):	
**Domain Totals:** Language (/28): Verbal Fluency (/24): Executive (/48): Memory (/24): Visuospatial (/12):	
**ECAS TOTAL score (/136):**	
**Semantic Fluency + Abrahams Correction**	NOT DONE ☐
**Date:**	Spoken ☐ **OR** Written ☐
**Semantic fluency:** Animal ☐ Fruit ☐ Vegetable ☐	
**Time to read aloud/copy (s):** **Number of correct words:**	
**Boston Naming Test – II (BNT)**	NOT DONE ☐
**Date:**	Spoken ☐ **OR** Written ☐
**Test version:** Short form ☐ Standard (Long) form ☐	
**Total score (/15 short, /60 long):**	
**Hopkins Verbal Learning Test – Revised (HVLT-R)**	NOT DONE ☐
**Date:**	Spoken ☐ **OR** Written ☐
**Test version:** Form 1 ☐ Form 2 ☐	
**Total recall:**	
**Delayed recall:**	
**Retention (%):**	
**Recognition Discrimination Index:**	
**Social Norms Questionnaire**	NOT DONE ☐
**Date:**	
**Sum of Items:** **SNQ22 Total Score:** **Break Score:**	
**Overadhere Score:** **Y/N Ratio Score:**	

Hospital Anxiety and Depression Scale (HADS)		NOT DONE ☐
**Date:**		
**Total score Anxiety (A /21):**	**Total score Depression (D /21):**	
**Score (/3) on Question "I feel as if I am slowed down":**		
**Center for Neurological Study – Lability Scale (CNS-LS)**		NOT DONE ☐
**Date:**		
**Total score (/35):**		

Frontal Systems Behavioral Scale (FrSBe) SELF-RATING FORM		NOT DONE ☐
**Date:**		
	**BEFORE ILLNESS (RAW SCORE)**	**PRESENT TIME (RAW SCORE)**
**Apathy:**		
**Disinhibition:**		
**Executive Dysfunction:**		
**Total:**		

Stroop Test (Victoria version)			NOT DONE ☐
**Date:**		Spoken ☐ **OR**	Colour Box Pointing ☐
**Trial 1 (Color Dots):**	Time taken (s):	# of errors:	
**Trial 2 (Color Words):**	Time taken (s):	# of errors:	
**Trial 3 (Color-Word Interference):**	Time taken (s):	# of errors:	

Benton Judgment of Line Orientation (JLO)		NOT DONE ☐
**Date:**	Spoken ☐ **OR**	Written ☐
**Test version:** V ☐ H ☐		
**Total score (/30):**		

Appendix 9.14.3: Neurocognitive Evaluations Form (Month 4 and 12) [ENGLISH]

Found in [Neurocognitive Evaluations Form (Month 4 and 12) (EN, V1, 03-JAN-2022) CAPTURE ALS]

Neurocognitive Evaluations Form
Visit 3 (month-4) and Visit 5 (month-12)

**Edinburgh Cognitive and Behavioral ALS Screen (ECAS)**					NOT DONE ☐
**Date:**			Spoken ☐	**OR**	Written ☐
**Test version:** A ☐ B ☐ C ☐					
**Language:**	Naming (/8):	Comprehension (/8):	Spelling (/12):		
**Verbal Fluency:**	First Letter (S/F/P) (/12):	Second Letter (T/D/M) (/12):			
**Executive:**	Reverse Digit Span (/12):	Alternation (/12):	Sentence Completion (/12):	Social Cognition (/12):	
**Memory:**	Immediate recall (/10):	Delayed retention (/10):	Delayed recognition (/4):		
**Visuospatial:**	Dot Counting (/4):	Cube Counting (/4):	Number Location (/4):		
**Domain Totals:**	Language (/28):	Verbal Fluency (/24):	Executive (/48):	Memory (/24):	Visuospatial (/12):
**ECAS TOTAL score (/136):**					
**Semantic Fluency + Abrahams Correction**					NOT DONE ☐
**Date:**			Spoken ☐	**OR**	Written ☐
**Semantic fluency:**	Animal ☐	Fruit ☐	Vegetable ☐		
**Number of correct words:**		**Time to read aloud/copy (s):**			

Appendix 9.14.4: Neurocognitive Evaluations Form (Caregiver) (Month 0 and 8) [ENGLISH]

Found in [Neurocognitive Evaluations Form (Caregiver) (Month 0 and 8) (EN, V1, 03-JAN-2022) CAPTURE ALS]

Neurocognitive Evaluations Form – Caregiver
Visit 2 (month-0) and Visit 4 (month-8)

**CAREGIVER QUESTIONNAIRES:**	
**Edinburgh Cognitive and Behavioral ALS Screen (ECAS) Caregiver Behaviour**	**NOT DONE ☐**
**Date:**	
**Behavioral disinhibition**	Score (/3):
**Apathy or Inertia**	Score (/1):
**Loss of sympathy or empathy**	Score (/2):
**Perseverative, stereotyped, compulsive, or ritualistic behaviour**	Score (/2):
**Hyperorality and altered food preferences**	Score (/2):
**Behavioral Total (/10):**	
**ALS Psychosis Screen**	
**Has strange or bizarre beliefs or behaviors:**	Y/N/DK
**Hears or sees things that are not there, and/or feels the presence of someone who is not there:**	Y/N/DK
**Is overly suspicious, and/or feels persecuted:**	Y/N/DK
**Score (/3):**	
**Onset and Duration of Symptoms**	
**1. Do these symptoms represent a CHANGE from the patient's previous behaviour?**	Y/N
If yes, did the changes occur:	
a. BEFORE the onset of the disease?	Y/N
b. At the same time as other symptoms?	Y/N
c. AFTER the onset of the disease?	Y/N
**2. Do they still persist?**	Y/N
**3. If not, how long did they last?**	
**Frontal Systems Behavioral Scale (FrSBe) FAMILY-RATING FORM**	**NOT DONE ☐**
**Date:**	

	**BEFORE ILLNESS (RAW SCORE)**	**PRESENT TIME (RAW SCORE)**
**Apathy:**		
**Disinhibition:**		
**Executive Dysfunction:**		
**Total:**		

Appendix 9.14.5: Neurocognitive Evaluations Form (Caregiver) (Month 4 and 12) [ENGLISH]

Found in [Neurocognitive Evaluations Form (Caregiver) (Month 4 and 12) (EN, V1, 03-JAN-2022) CAPTURE ALS]

**Neurocognitive Evaluations Form – Caregiver<br>Visit 3 (month-4) and Visit 5 (month-12)**	
**CAREGIVER QUESTIONNAIRES:**	
**Edinburgh Cognitive and Behavioral ALS Screen (ECAS) Caregiver Behaviour**	**NOT DONE ☐**
**Date:**	
**Behavioral disinhibition**	Score (/3):
**Apathy or Inertia**	Score (/1):
**Loss of sympathy or empathy**	Score (/2):
**Perseverative, stereotyped, compulsive, or ritualistic behaviour**	Score (/2):
**Hyperorality and altered food preferences**	Score (/2):
**Behavioral Total (/10):**	
**ALS Psychosis Screen**	
**Has strange or bizarre beliefs or behaviors:**	Y/N/DK
**Hears or sees things that are not there, and/or feels the presence of someone who is not there:**	Y/N/DK
**Is overly suspicious, and/or feels persecuted:**	Y/N/DK
**Score (/3):**	
**Onset and Duration of Symptoms**	
**1. Do these symptoms represent a CHANGE from the patient's previous behaviour?**	Y/N
If yes, did the changes occur:	
a. BEFORE the onset of the disease?	Y/N
b. At the same time as other symptoms?	Y/N
c. AFTER the onset of the disease?	Y/N
**2. Do they still persist?**	Y/N
**3. If not, how long did they last?**	
