# Supplementary material for: Comprehensive analysis platform to understand, remedy, and eliminate amyotrophic lateral sclerosis (CAPTURE ALS): Study protocol for a Canadian multicenter, multimodal, longitudinal observational study
Source: PLoS One. 2025 Dec 4;20(12):e0332430. doi: 10.1371/journal.pone.0332430 (PMC12677780; doi:10.1371/journal.pone.0332430)
Supplement: S9 Appendix — (PDF) [file pone.0332430.s009.pdf]

S9 Appendix. Biosample Collection, Processing, Storage, Shipment SOP.

CAPTURE ALS

BIOSAMPLE COLLECTION, PROCESSING, STORAGE, SHIPMENT

SOP01EN02

Issue Date: 26-October-2022
Version: 2.0

Prepared by:

Claire Magnussen
Program Manager

26-October-2022
Date

Approved by:

Sanjay Kalra MD
CAPTURE ALS National PI

26-October-2022
Date

TABLE OF CONTENTS

1. SCOPE AND APPLICABILITY 4
2. SUMMARY OF METHOD 4
3. DEFINITIONS 4
4. PERSONNEL QUALIFICATIONS/RESPONSIBILITIES 4
5. CAUTIONS 5
6. EQUIPMENT AND SUPPLIES 5
6.1. Equipment 5
6.2. Supplies 5
7. PROCEDURES 8
7.1. Recommended Biosample Collection/Visit 8
7.2. Recommended Order of Blood Draw Tubes 9
7.3. Managing Biosamples in the LORIS Database 9
7.4. Generating and Printing Labels Overview 9
7.4.1. Collection Tube and Cryovial Labels 9
7.4.3. Creating Labels 10
7.4.3.1. Biosample Label Excel Template 10
7.4.3.2. Generating labels 10
7.4.3.3. Changing labels location 11
7.4.4. Using Labels 12
7.4.4.1. Label placement 12
7.4.4.2 Scanning labels into LORIS 12
7.5. Whole Blood for Serum Isolation 13
7.6. Whole Blood for PBMC Isolation 14
7.7. Whole Blood for DNA Extraction 15
7.8. Whole Blood for Plasma Isolation 16
7.9. Whole Blood for RNA Extraction 18
7.10. Cerebrospinal Fluid (CSF) Collection (optional procedure) 19
7.11. PACKAGING AND SHIPPING INSTRUCTIONS 20
7.11.1. Shipping to C-BIG Repository 20

7.11.2. Shipping Address 20
7.11.3. C-BIG Hours of Operation and Holidays 20
7.11.4. Frozen Sample Shipments 21
7.11.4.1. Frozen Sample Shipping Supplies 21
7.11.4.2. Frozen Sample Packing Instructions 22
7.11.4.3. Frozen Sample Shipping Instructions 22
7.11.5. Ambient Sample Shipments (Credo) 24
7.11.5.1. Ambient Sample Shipping Supplies 24
7.11.5.2. Ambient Sample Packing Instructions 24
7.11.5.3. Ambient Sample Shipping Instructions 24
7.11.5.4. Credo Return Shipping Instructions 26
8. APPENDICES 27
Appendix 8.1: CAPTURE ALS Biosample Collection Form [Blood] 28
Appendix 8.2: CAPTURE ALS Biosample Collection Form [CSF] 29
Appendix 8.3: Sample Processing Form - Serum 30
Appendix 8.4: Sample Processing Form - PBMC via Leucosep 31
Appendix 8.5: Sample Processing Form - Plasma 33
Appendix 8.6: Sample Processing Form - RNA 34
Appendix 8.7: Sample Processing Form – CSF 35
Appendix 8.8: Biosample Labels Template 36
Appendix 8.9: CAPTURE ALS Packing Slip 37

1. SCOPE AND APPLICABILITY

This SOP describes the processes for the labelling, collection, processing, shipment, and storage of CAPTURE ALS biosamples including blood and cerebrospinal fluid (CSF).

2. SUMMARY OF METHOD

Following participant consent, blood, and optional CSF will be collected by qualified medical professionals according to the schedule outlined in the CAPTURE ALS protocol and following local site SOPs.

Blood: Blood will be processed at the site of collection to isolate serum and plasma, which will be aliquoted, and batch shipped to the C-BIG Repository for long-term storage. In general, blood will be shipped to and processed by the C-BIG Repository to isolate DNA, and PBMCs and for long-term storage of RNA.

CSF: 1-2mL of CSF will be sent to the site's local laboratory for analysis of cell count, protein, and glucose. The remaining CSF will be processed and aliquoted at the site of collection and batch shipped to the C-BIG Repository for long-term storage.

3. DEFINITIONS

C-BIG	Clinical Biological Imaging and Genetic Repository
CSF	Cerebrospinal Fluid
DNA	Deoxyribonucleic acid
DOT	Department of Transportation
EDTA	Ethylenediaminetetraacetic acid
g	Gravity
GTT	Green Top Blood Collection Tube (Sodium Heparin)
IATA	International Air Transport Association
LIMS	Laboratory Information Management System
LORIS	Longitudinal Online Research and Imaging System
MD	Medical Doctor
NCCLS	National Committee for Clinical Laboratory Standards
OSHA	Occupational Safety and Health Administration
PBMCs	Peripheral blood mononuclear cells
PTT	Purple Top Blood Collection Tube
RBC	Red blood cells
RNA	Ribonucleic acid
RTT	Red Top Blood Collection Tube

4. PERSONNEL QUALIFICATIONS/RESPONSIBILITIES

This SOP concerns all personnel who will be delegated to process human biosamples for CAPTURE ALS. All personnel processing specimens must be currently certified in the transportation of dangerous goods.

5. CAUTIONS

- All biosamples derived from a human source are considered to be biohazardous. Use appropriate precautions when working with such samples (i.e. personal protection equipment such as gloves, lab coat and safety glasses). All waste (samples and related contact materials) must be placed in marked biohazardous waste containers and disposed of under hospital or institutional guidelines.
- Ensure that informed consent is obtained prior to participant biosample collection.
- Follow your facility's SOPs for biosample collection.
- Blood collection at each visit must not exceed 12 tubes of blood which is equivalent to 8 tablespoons or 120mL.
- CSF collection at each visit must not exceed 16mL.
- Best results are obtained when samples are processed promptly.

6. EQUIPMENT AND SUPPLIES

The equipment and supplies listed below are recommendations only and may be substituted by alternative/equivalent product more suitable for site-specific tasks or procedures.

6.1. Equipment

- Room temperature centrifuge with swinging bucket (2000-2500g)
- Refrigerated centrifuge with swinging bucket (250g)
- -20˚C freezer
- -80˚C freezer
- Micropipettes (100-1000µL) and tips
  - **Note:** if a pipette is not available, sterile transfer pipettes may be used
- Laser printer
- Windows computer with Microsoft Office and working internet
- Barcode scanner
  - Suggested: Eyoyo Wired USB 2D Barcode Scanner, Model EY-H2
    https://www.amazon.ca/Eyoyo-Handheld-Convenience-Supermarket-Warehouse/dp/B088QV215Y/ref=pd_lpo_1?pd_rd_i=B088QV215Y&psc=1

6.2. Supplies

CAPTURE ALS *suggests* the following supplies for the local collection, processing, storage, and shipment of all CAPTURE ALS biosamples.

**Supply Name**	**Supply Description**	**Suggested Vendor**	**Item/ Catalog #**	**Comments**
Blood Collection Materials	N/A	At site's discretion	N/A	alcohol wipes, needles, gauze, bandaids etc

Cryovials, 1mL, conical bottom	Nunc Biobanking and cell culture cryogenic tube 1mL; polypropylene; screw cap; self-standing	ThermoFisher	366656	10 cryovials = plasma<br>15 cryovials = serum<br>45 cryovials = CSF
Sterile Transfer Pipettes	Corning™ Falcon™ Plastic DisposableTransfer Pipet; Individually Wrapped; Sterile 3mL; 1,2mL graduations	Fisher Scientific	13-669-12	To aliquot samples
15mL Tube	15mL polypropylene tube; graduation; screw cap	Sarstedt	62.554.205	1 for processing of each, serum, plasma, CSF
Cryogenic labels	Fisherbrand Micryo Label; Laser Printer Sheets; 1.2 x 2.5 cm, -196°C to 121°C	Fisher Scientific	15-930-A	1 label per vacutainer and cryovial<br>Use FISHC6 label template
**SERUM COLLECTION**				
BD Vacutainer™ venous blood collection tube, red top (RTT)	10.0mL; Glass; Conventional Closure; Red Cap; Silicone Coated	Fisher Scientific	02-685-A (BD 366430)	2 tubes per serum sample
**PMBC COLLECTION**				
BD Vacutainer™ blood collection tube with Sodium Heparin, green top (GTT)	10mL; Glass; Conventional Closure; Green cap; Sodium Heparin	Fisher Scientific	02-685-3B (BD 366480)	5 tubes per PBMC sample
**PLASMA AND DNA COLLECTION**				
BD Vacutainer™ blood collection tube with K_2EDTA, purple top (PTT)	10mL; Plastic; Hemogard Closure; Lavender Cap; K_2EDTA	Fisher Scientific	02-657-32 (BD 366643)	2 tubes for DNA<br>1 tube for plasma sample
**RNA COLLECTION**				
PAXgene blood RNA tube	2.5mL; Plastic; Hemogard Closure; Red Cap; RNA stabilization additive	BD	BD 762165	2 tubes per RNA sample
**CSF COLLECTION**				
Lumbar Puncture Tray, preferred needle, additional supplies	N/A	At site's discretion		1 kit per LP
15mL Tube	15mL polypropylene tube; graduation; screw cap	Sarstedt	62.554.205	2 per CSF collection
**SHIPPING MATERIALS**				
Parafilm, Elastics, Packing Tape	N/A	At site's discretion	N/A	
Thermo Scientific Cryoboxes	Cryoboxes, Chipboard, White, holds 81 vials, 132 x 132 x 51mm	Fisher Scientific	12-565-182	Holds 81 cryovials split participant samples across 2 boxes
Absorbent Materials	Absorbent Sheet; 3 x 6 in.; Absorbs up to 90mL	Fisher Scientific	22-130-040	Utilize per IATA standards

Dry Ice Label	Labels, Dry ice; Therapak; 5.5 x 5.5 in.; Complies with DOT (49 CFR 173.217) and IATA	Fisher Scientific	22-130-065	1 per shipping box
UN 3373 Biological Substance Category B Labels	8 x 4 in.; for shipping biological substances by air; complies with IATA regulations	Fisher Scientific	22-130-069	1 per shipping box
Biohazard Specimen Transport Bags	Transport Bag, Specimen; Fisherbrand; Zippered; Polyethylene; Three-wall; With attached document compartment; Comply with OSHA and NCCLS guidelines; Large printed biohazard graphic; Size: 8 x 10 in.	Fisher Scientific	01-800-08	1 per cryovial box
Absorbent Pouch	6 Bay Aqui-Pak, Absorbent Pak, Therapak	VWR	89170-936 (Therapak 10316)	1 per PBMC collection
Saf T Pak Leak Proof Bag and Tyvek Outer Envelope	Disposable 2-Part Secondary Pressure Vessel (95KPA), Medium; Inner Leak Proof Polybag 9.25 X 12in, Tyvek outer envelope, 7.5 X 9.5in	ESBE Scientific	STP-710	1 per PBMC collection
**FROZEN SHIPMENTS**				
Insulated Shipper	Shipper, Insulated; ThermoSafe; For Pharamceutical Reagents; Economical; Lightweight; Reusable; Recyclable; Two-piece shippers with extra-thick insulating walls of expanded polystyrene; Dimensions (outside): 14-7/8 x 13-1/8 x 12-1/8 in.; Dimensions (inside): 11-3/4 x 10 x 9-1/8 in	Fisher Scientific	03-529-1	About two (2) 81 cell boxes fit per shipper with dry ice
**AMBIENT SHIPMENTS**				
Credo Cube Series 22 -248	Credo Cube^{TM} Series 22, 2L, +15˚C -+25˚C, PCM Coolant, 53h Duration, 127.5m^3; Dimensions (outside, mm): 276 X 250 X 241; 4.4kg	Fischer Scientific	NC1578810 (Pelican GH02APLAT CM22C-WT)	These boxes are re-usable and will be shipped back to the site

7. PROCEDURES

7.1. Recommended Biosample Collection/Visit

Biosample Collection Form [blood] (Appendix 8.1) and Biosample Collection Form [CSF] (Appendix 8.2) must be completed by the person collecting the biosample.

**Patient Participant**		
Visit	Timepoint	Suggested Collection Protocol
2	0<br>Month	2 RTT (10mL) → Serum<br>5 GTT (Heparin, 10mL) → PBMC<br>3 PTT (K_2EDTA, 10mL) → Plasma (1X), DNA (2X)<br>2 PAXgene RNA (2.5mL) → RNA<br>**Total Blood Tubes: 12**<br>**Total Blood Volume: 105mL**<br>CSF (up to 16mL) → optional procedure for patients only 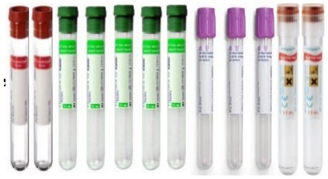
3	4<br>Month	2 RTT (10mL) → Serum<br>1 PTT (K_2EDTA,10mL) → Plasma<br>**Total Blood Tubes: 3**<br>**Total Blood Volume: 30mL**<br>CSF (up to 16mL) → optional procedure for patients only 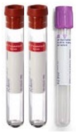
4	8<br>Month	2 RTT (10mL) → Serum<br>1 PTT (K_2EDTA,10mL) → Plasma<br>**Total Blood Tubes: 3**<br>**Total Blood Volume: 30mL**<br>CSF (up to 16mL) → optional procedure for patients only 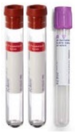
5	12<br>Month	2 RTT (10mL) → Serum<br>1 PTT (K_2EDTA,10mL) → Plasma<br>**Total Blood Tubes: 3**<br>**Total Blood Volume: 30mL**<br>CSF (up to 16mL) → optional procedure for patients only 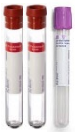

**Control Participant**		
Visit	Timepoint	Suggested Collection Protocol
2	0<br>Months	2 RTT (10mL) → Serum<br>1 PTT (K_2EDTA,10mL) → Plasma<br>2 PAXgene RNA (2.5mL) → RNA<br>**Total Blood Tubes: 5**<br>**Total Blood Volume: 35mL** 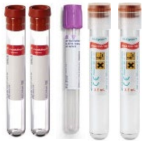
3	8<br>Months	2 RTT (10mL) → Serum<br>1 PTT (K_2EDTA, 10mL) → Plasma<br>**Total Blood Tubes: 3**<br>**Total Blood Volume: 30mL** 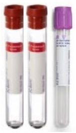

7.2. Recommended Order of Blood Draw Tubes

1. Red Top Tubes (Serum)
2. Green Top Heparin Tubes (PBMC)
3. Purple Top Tubes (Plasma, DNA)
4. PAXgene Tubes (RNA)

7.3. Managing Biosamples in the LORIS Database

The LORIS database (https://cbigr.loris.ca/ , login using unique username and password) will be used to manage all samples collected through CAPTURE ALS. Personnel managing biosamples will need to request access to the Laboratory Information Management System (LIMS) module of LORIS (see LORIS manual). Samples will be collected, processed, stored, and shipped using the guidelines described below in sections 7.5 to 7.11. Processing of each sample type must be documented in LORIS. Detailed instructions and LORIS screenshots can be found in the LORIS manual. Paper processing forms may be used as an intermediary if data entry is not done directly in LORIS. The processing forms, specific to each sample type, can be found in the Appendix: Sample processing form - Serum (Appendix 8.3), Sample Processing Form - Plasma (Appendix 8.5), Sample Processing Form – CSF (Appendix 8.7). Sample Processing Form - PBMC (Appendix 8.4) and RNA processing form (Appendix 8.6) are included but may not be required by sites, as these samples will, in most cases, be processed by the C-BIG Repository. Any deviations to the sample processing protocols should be documented in the *comment section* of LORIS. Each sample will be identified with a label containing a QR code which will allow it to be scanned into LORIS (see section 7.4.4.2).

7.4. Generating and Printing Labels Overview

This section describes the processes to create labels for CAPTURE ALS biosamples (vacutainers and cryovials) at local sites, only. Labels generated at C-BIG will follow the existing LORIS formatting.

7.4.1. Collection Tube and Cryovial Labels

Each collection tube (vacutainer or 15mL tube) and cryovial label should contain the following information:

- **CAPTURE ALS Participant ID:** CAPTXXXXXXX
- **Vial #:** A unique number that should never be repeated within a participant
- **Sample type:** serum, plasma, CSF, DNA, RNA, PBMC
- **Visit Label:** 00M, 04M, 08M, 12M
- **Collection Date:** DD/MM/YYYY
- **QR code:** generated automatically from CAPT ID and Vial #

7.4.3. Creating Labels

7.4.3.1. Biosample Label Excel Template

- Use the provided biosample label template (Appendix 8.8) to generate labels for collection and local processing of biosamples.
    - This file contains four sheets, one for each visit (00M, 04M, 08M, 12M).
    - Each sheet contains all possible labels for a visit, including labels for vacutainers to collect whole blood or CSF, and labels for cryovials containing processed samples. Note: not all labels may be required for a particular visit. Consult the protocol and section 7.1 to determine what samples are collected at each visit.
    - Rows 2-15 contain vacutainer labels. Labels contain the PSCID or CAPTURE ID (CAPTXXXXXX), unique vial #, sample type, visit label (XXM), collection date (DD/MM/YYYY) and barcode. The barcode is automatically generated from the PSCID + vial #.
    - Rows 16-85 contain cryovial labels. The template will generate 15 serum labels, 10 plasma labels and 45 CSF labels. Labels contain the PSCID or CAPTURE ID (CAPTXXXXXX), unique vial #, sample type (serum, plasma, CSF), visit label (XXM), collection date (DD/MM/YYYY) and barcode. The barcode is automatically generated from the PSCID + vial #.
- Prior to each participant visit, complete the required fields (Xs, DD/MM/YYYY) in the label template. Ensure that the sheet that corresponds to the correct visit (00M, 04M, 08M, 12M) is used. Never modify the unique vial #, as this number is used to generate a unique barcode.

7.4.3.2. Generating labels

- Once the biosample label template has been completed with the participant information, Open Word.
- Click “Open” and open the label layout template provided: “*FISH-C6 Label Template CAPTURE ALS”.*
- Go to “Mailings” tab. Under the “Start Mail Merge”, select “Labels” and click “Cancel” in the Label Options pop up to activate the label template specified in the current Word file.
- Select “Select Recipients” and select “Use an Existing List” option.
- Select the excel file containing biosample label data.
- Select the correct visit point (00M, 04M etc.).
- Select “Edit Recipient List” and select only the rows that you wish to print. E.g. Do not select PBMC rows if you are not collecting PBMCs at this visit.
- Select “Insert Merge Field” and select all the options (PSCID, Vial #, Type, Visit Label, Collection Date, Barcode) one at a time by pressing “insert” after each option is selected.
- Make sure the font of «PSCID» «Vial_» «Type» «VisitLabel» «CollectionDate» is set to “Calibri (Body)” and font size to 4.

- Add a space after each selection (e.g. «PSCID»space«Vial_»).
- Press enter to bring “«Barcode»” to a separate line and right click on “Toggle Field Codes”.
- Replace MERGEFIELD with MERGEBarcode so that it reads MERGEBarcode Barcode Right click on MERGEBarcode Barcode to select the option “Edit Barcode”.
- Under the “Barcode type” select QR Code.
- On the bottom left, select “Advanced” and set scale to 28%.
- Close by pressing OK.
- Center the labels and select “Update Labels”.
- Select “Preview Results”.
  Note: You may see incorrect number of labels or 1 label only as Word will shift to the last label hiding others.
- Press on the icon shown in the image below underlined in red to shift back to the first label.

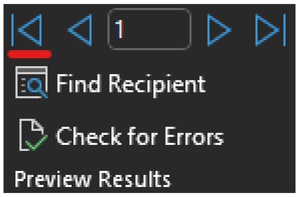

- Select “Finish & Merge”, “Print Documents” and then select “All” to print out the labels.
- Print to a laser printer
  Note: Only print labels from a word file. Never print from a PDF as it will make QR code unreadable by the scanner. Load the label paper into the printer tray, do not use the manual feed as this may cause the labels to print out of the margins.
- Test the QR codes using a barcode scanner to ensure that they can be read.

7.4.3.3. Changing labels location

A label sheet may have already been partially used. To start printing labels from a location other than the top left cell follow these steps:

- Finish creating labels as normal
- Cut the labels by highlighting the labels, right clicking the mouse and selecting the option “cut”
- Select the desired cell to paste the label selected above
- Right click the mouse and select “Paste” to move the label in selected cell

7.4.4. Using Labels

7.4.4.1. Label placement

Ensure that the label is placed **vertically** on the cryovial tube or vacutainer with the longest side of the label going up the length of the cryovial. DO NOT wrap the label around the tube, the QR code may not be readable.

7.4.4.2 Scanning labels into LORIS

Each sample will be identified with a label containing a unique QR code. Following processing, the barcode scanner will be used to scan each QR code, entering the biosample into LORIS. This process links each biosample to a participant, visit and processing details. For full details see the LORIS manual. This step requires access to a computer.

7.5. Whole Blood for Serum Isolation

Whole blood samples are collected at each visit for patient (MONTH 0, 4, 8, 12) and control participants (MONTH 0, 8). Samples are processed ***locally*** at each site for serum isolation. Two (2) tubes are suggested for this type of collection/visit.

WHOLE BLOOD COLLECTION FOR SERUM

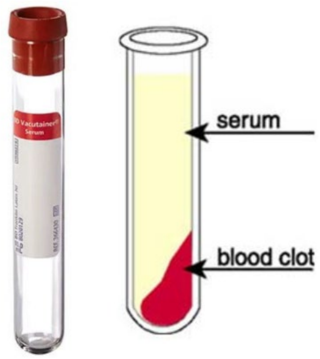

(BD Diagnostic, Cat# 366430*)*

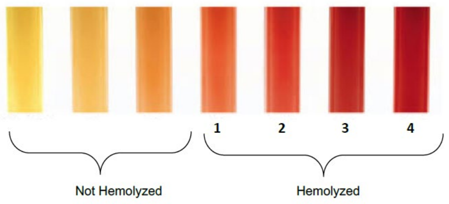

Hemolysis Index

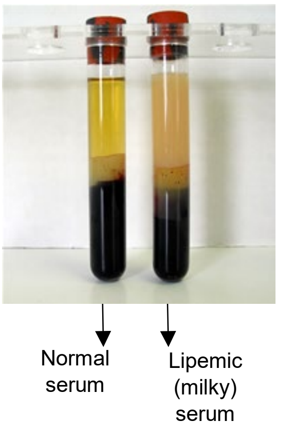

PREPARATION

Prepare two (2) 10mL red top tubes (RTT) by affixing labels.
Prepare up to fifteen (15) 1.0mL cryovials by affixing labels.

COLLECTION

- Completely fill two (2) 10mL RTT with blood.
- Complete the *Biosample Collection Form [blood]* (Appendix 8.1).
- Keep tube upright at room temperature for a minimum of 30min and maximum of 120 min to allow blood to clot.

PROCESSING

- Serum isolation will be performed at the local site following SOP *CBIG-02-003 titled "Serum Isolation from Whole Blood" (01May2020)* as summarized below. Document serum processing on LORIS or by first completing the *serum processing form* (Appendix 8.3)
- Centrifuge at room temperature for 10 min at 2500Xg using a centrifuge with a swinging bucket.
- Indicate the degree of hemolysis (0-4) and whether the sample is lipemic (milky).
- Using a sterile transfer pipette, transfer supernatant (serum) to a new 15mL conical tube without disturbing the pellet.
- Using a micropipette or sterile transfer pipette, transfer 0.5mL aliquots into the 1.0mL cryovials, filling as many vials as possible.

STORE FROZEN

- Store in a -80ºC freezer until ready for shipment.
- Only if absolutely needed, aliquots can be stored at -20 ºC for up to 1 week before transferring to -80 ºC.

SHIP

- Split the cryovials into two 81-cell freezer storage boxes, i.e., half of the samples in one box, half in the other. Each box should be shipped separately.
- For each participant, group samples by type and time point in the boxes.
- Bulk ship frozen samples on dry ice (see 7.11.4).

7.6. Whole Blood for PBMC Isolation

Whole blood samples are collected from patient participants at a single time-point (MONTH 0 suggested) and shipped at room temperature to C-BIG for PBMC isolation. Five (5) tubes are suggested for this type of collection. No aliquoting is required.

**WHOLE BLOOD COLLECTION FOR PBMC**<br>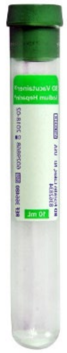<br>*(BD Diagnostic, Cat# 366480)*	**PURPOSE OF COLLECTION**<br>PBMC isolation at C-BIG.<br><br>**PREPARATION**<br>Prepare five (5) 10mL green top sodium heparin tubes (GTT) by affixing labels.<br>Ensure the tubes are at room temperature (15-25˚C). ***Warning:*** *Green vacutainers require careful handling, they are made of glass.*<br><br>**COLLECTION**<br>• Completely fill five (5) 10mL green top sodium heparin tubes with blood.<br>• Gently invert tube 8-10 times immediately after blood draw.<br>• Complete the *Biosample Collection Form [blood*] (Appendix 8.1).<br><br>**SHIP**<br>• Vacutainers should be kept at room temperature (15-25˚C) and shipped immediately.<br>• Ship priority overnight at ambient temperature in Credo Cube to C-BIG (see 7.11.5).<br><br>**PROCESSING**<br>PBMC isolation will be performed by C-BIG following SOP *CBIG-02-002 titled "PMBC Isolation from Whole Blood (Leucosep Method) (30Apr2020).* Only if PBMC processing is done locally, does the *PBMC processing form* (Appendix 8.4) need to be completed on LORIS.

7.7. Whole Blood for DNA Extraction

Whole blood samples are collected from patient participants at MONTH 0 and shipped to C-BIG for DNA extraction. Two (2) tubes are suggested for this type of collection. No aliquoting is required.

WHOLE BLOOD COLLECTION FOR DNA

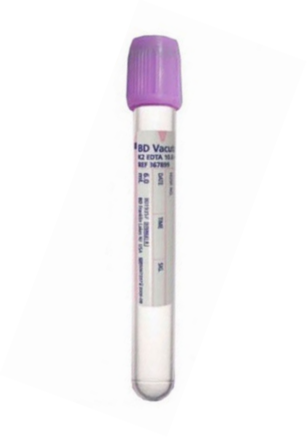

(BD Diagnostic, Cat# 366643*)*

PURPOSE OF COLLECTION

DNA extraction at C-BIG.

PREPARATION

Prepare two (2) 10mL purple top K_2EDTA tubes (PTT) by affixing labels.

COLLECTION

- Completely fill two (2) 10mL purple top K_2EDTA tubes with blood.
- Complete the *Biosample Collection Form [blood]* (Appendix 8.1).

STORE FROZEN

- Store vertically in a -80ºC freezer until ready for shipment.
- Only if absolutely needed, vacutainer can be stored at -20 ºC for 2 days before transferring to -80 ºC.
- ***Warning:*** *Tubes will expand during freezing, avoid storing them in a confined rack (e.g. Styrofoam rack).*

SHIP

- Place the frozen tube(s) in a biohazard bag.
- DNA tubes from a single participant should be shipped separately from one another.
- Bulk ship frozen samples on dry ice (see 7.11.4).

PROCESSING

DNA extraction will be performed by C-BIG following SOP *CBIG-02-004, titled "DNA Extraction from Whole Blood (Accelerated Method)" (01May2020).*

7.8. Whole Blood for Plasma Isolation

Whole blood samples are collected at each visit for patient (MONTH 0, 4, 8, 12) and control participants (MONTH 0, 8). Samples are processed ***locally*** at each site for plasma isolation. One (1) tube is suggested for this type of collection/visit.

PLASMA COLLECTION

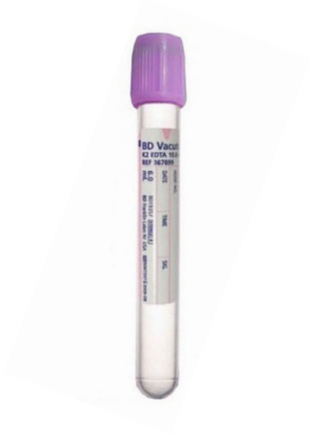

(BD Diagnostic, Cat# 366643*)*

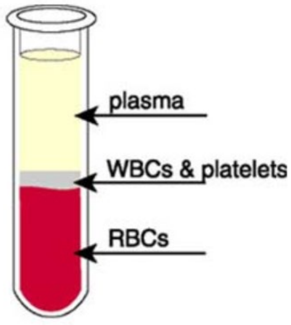

Plasma is the top layer. White blood cells and platelets are in a whitish layer called the "buffy coat", just under the plasma and above the red blood cells.

PREPARATION

Prepare one (1) 10mL purple top K_2EDTA tube (PTT) by affixing a label.

Prepare up to ten (10) 1.0mL cryovials by affixing labels.

COLLECTION

- Completely fill one (1) purple top K_2EDTA tube with blood.
- Store purple top tube upright at room temperature away from direct light for a minimum of 30 min to allow blood to sediment.
- Complete the *Biosample Collection Form [blood]* (Appendix 8.1).

PROCESSING

- Plasma isolation will be performed at the local site following SOP *CBIG-02-011 titled "Plasma Isolation from Whole Blood" (02Oct2020)* as summarized below. Document plasma processing on LORIS or by first completing the *plasma processing form* (Appendix 8.5).
- Centrifuge at 4ºC for 12 min at 250Xg using a centrifuge with a swinging bucket.
- Using a sterile transfer pipette, quickly transfer supernatant (plasma) to a new 15mL polypropylene conical tube.
- ***Warning:*** *Do not disturb the buffy coat.*
- Using a micropipette or sterile transfer pipette, transfer 0.5mL aliquots into the 1.0mL cryovials, filling as many vials as possible.

STORE FROZEN

- Plasma samples can be transferred directly to -80˚C freezer until shipment.
- Only if absolutely needed, aliquots can be stored at -20 ºC for 2 days before transferring to -80 ºC.

	**SHIP**<br>• Split the cryovials into two 81-cell freezer storage boxes, i.e., half of the samples in one box, half in the other. Each box should be shipped separately.<br>• For each participant, group samples by type and time point in the boxes.<br>• Bulk ship frozen samples on dry ice (see 7.11.4).

7.9. Whole Blood for RNA Extraction

Whole blood samples are collected from patient and controls participants at MONTH 0 and shipped to C-BIG for RNA storage. Two (2) tubes are suggested for this type of collection. No aliquoting is required.

<table>
<tr>
<td>

WHOLE BLOOD

COLLECTION FOR

RNA

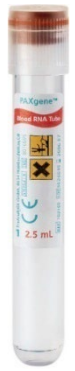

(BD Diagnostic, Cat# 762165*)*

</td>
<td>

PURPOSE OF COLLECTION

RNA storage at C-BIG.

PREPARATION

Prepare two (2) 2.5mL PAXgene RNA tubes by affixing labels.

COLLECTION

- Completely fill two (2) 2.5mL PAXgene RNA tubes with blood.
- Gently invert tube 8-10 times immediately after blood draw.
- Complete the *Biosample Collection Form [blood]* (Appendix 8.1)*.*

STORE FROZEN

- Follow protocol *“CAPTURE ALS RNA Processing (07Mar2022)”* as summarized below and found in Appendix 8.6. This “processing” does not need to be documented in LORIS.
- Keep tubes upright at room temperature for a minimum of 2 hours and a maximum of 72hours to allow blood to clot.
- Transfer tubes to -20ºC for at least 24hours before transferring to -80ºC freezer until ready for shipment.

SHIP

- Place the frozen tube(s) in a biohazard bag.
- RNA tubes from a single participant should be shipped separately from one another.
- Bulk ship frozen samples on dry ice (see 7.11.4).

PROCESSING / LONG-TERM STORAGE

RNA tubes will be stored by C-BIG at -80ºC for future RNA extraction.

</td>
</tr>
</table>

7.10. Cerebrospinal Fluid (CSF) Collection (optional procedure)

CSF COLLECTION

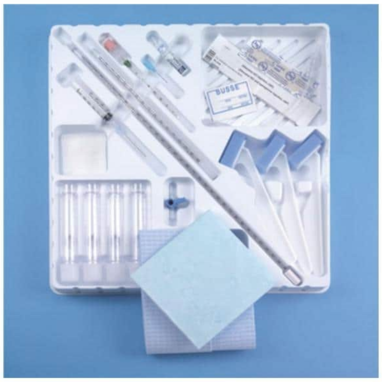

Local site's preferred lumbar puncture tray

PREPARATION

Prepare lumbar puncture tray and additional supplies and two (2) 15mL polypropylene tubes by affixing labels.

Prepare up to forty-five (45) 1.0mL cryovials by affixing labels.

***Warning:** Coagulation and platelets tests must be reviewed by a MD within 4 weeks of performing a lumbar puncture.*

COLLECTION

- A licensed practitioner will collect **up to 16mL** of CSF in accordance with local site SOPs.
- CSF should be collected in polypropylene tubes due to their low protein binding potential, no additives should be used.
- Immediately after collection, keep the CSF at 4ºC or on ice until ready to process.
- Complete the *Biosample Collection Form [CSF]* (Appendix 8.2).

PROCESSING

- Send 1-2ml of collected CSF to local laboratory for cell count, protein and glucose analysis, follow local laboratory guidelines.
- CSF processing will be performed at the local site following SOP *CBIG-02-008 titled "CSF Processing" (01May2020)* as summarized below. Document CSF processing on LORIS or by first completing the *CSF processing form* (Appendix 8.7).
- Process the sample promptly.
- Using a sterile transfer pipette, transfer the CSF to a new 15mL polypropylene tube.
- Centrifuge at room temperature for 10min at 2000Xg using a centrifuge with a swinging bucket.
- Using a sterile transfer pipette, transfer the supernatant (CSF) to a new 15mL polypropylene tube, do not disturb the pellet.
- Using a micropipette or sterile transfer pipette, transfer 0.5mL aliquots into the 1.0mL cryovials, filling as many vials as possible.

	**STORE FROZEN**<br>• Store cryovials directly in a -80ºC freezer until ready for shipment. CSF cannot be stored at -20ºC.<br><br>**SHIP**<br>• Split the cryovials into two 81-cell freezer storage boxes, i.e., half of the samples in one box, half in the other. Each box should be shipped separately.<br>• For each participant, group samples by type and time point in the boxes.<br>• Bulk ship frozen samples on dry ice (see 7.11.4).

7.11. PACKAGING AND SHIPPING INSTRUCTIONS

7.11.1. Shipping to C-BIG Repository

- Samples will be shipped either frozen on dry ice or at ambient temperature in a credo cube.
- Frozen samples will be shipped in two batches to prevent the loss of all samples from a participant in the event a shipment is lost or thawed prior to delivery.
- Samples will be shipped on Monday to Thursday for delivery on Tuesday to Friday. *Avoid shipping on Friday to prevent sample delivery on a weekend.*
- Prior to shipment of samples, confirmation of shipment date, via telephone or email, shall be made with the C-BIG coordinator.
- To schedule a shipment, visit https://www.fedex.com/en-ca/shipping.html
- Sites will receive a sample shipment confirmation from C-BIG after samples arrive with instructions when to ship the second box.

7.11.2. Shipping Address

**Mailing Address:**	Attention: Marie-Noelle Boivin<br>C-BIG Repository<br>Montreal Neurological Institute<br>3801 University Street<br>Room NWB150<br>Montreal, Quebec<br>H3A 2B4
**Phone Number:**	514-398-8439
**Email:**	cbig.mni@mcgill.ca

7.11.3. C-BIG Hours of Operation and Holidays

Monday to Friday 7:30 am to 4:30 pm Eastern Time

C-BIG lab coordinators will be available to receive shipments from 7:45AM to 3:00PM.

If a shipment at C-BIG is anticipated outside of these hours, an arrangement MUST be made with the C-BIG coordinator at the contact information above.

Holiday Observations

Based on previous years, FedEx observes the following holidays. These dates are subject to change by FedEx.

It is ***strongly recommended*** that you verify FedEx and C-BIG schedules prior to any holiday shipments to Montreal.

<u>Avoid collecting specimens for shipment on the day before an observed holiday</u>

New Years Day	January 1
Good Friday	Date varies yearly
Easter Monday	Date varies yearly
Victoria Day	The second to last Monday in May
St. Jean Baptiste Day	June 24
Canada Day	July 1
Labor Day	1st Monday in September
Thanksgiving	2nd Monday in October
Christmas Day	December 25
Boxing Day	December 26

7.11.4. Frozen Sample Shipments

The following samples are shipped frozen on dry ice to C-BIG:

1. Serum, plasma, and CSF cryovials
2. Purple Top DNA Collection Vacutainer(s)
3. PAXgene RNA Collection Vacutainer(s)

7.11.4.1. Frozen Sample Shipping Supplies

- Biohazard Specimen Transport Bags
- Absorbent Sheet(s)
- Dry Ice Label(s)

- UN 3373 Biological Substance Category B Label(s)
- Insulated Shipping Container(s)
- Dry Ice
- Packing Tape

7.11.4.2. Frozen Sample Packing Instructions

- Order dry ice from your preferred supplier. 10lbs of dry ice is required for the suggested insulated Styrofoam shipping container.
- Complete the packing slip (see Appendix 8.9) and place it in the shipping container. Retain a copy for site records. If DNA or RNA tubes are being sent, include a copy of the Biosample Collection Form [Blood] (Appendix 8.1) showing the collection details. This will facilitate processing at C-BIG.
- Cryovials: Place each 81-cell sample cryobox in a biohazard specimen transport bag with an absorbent sheet. **Do not tape the 81-cell sample box.**
- Whole Tubes: Place frozen whole blood tube(s) in a biohazard bag with an absorbent sheet.
- Place up to two (2) biohazard specimen transport bags each containing a single 81-cell box in an insulated Styrofoam shipping container. A third biohazard bag containing whole blood tubes may be added to the same shipping container.
- Surround samples on all sides (bottom, top, sides) with dry ice (10lbs). **Do not tape the insulated Styrofoam shipping container.**
- Seal the cardboard shipping box with standard packing tape.

7.11.4.3. Frozen Sample Shipping Instructions

- Affix the ‘Substance B’ shipping label to the outside of the cardboard shipping box.
- Complete the ‘Dry Ice’ shipping label and affix to the outside of the shipping box.

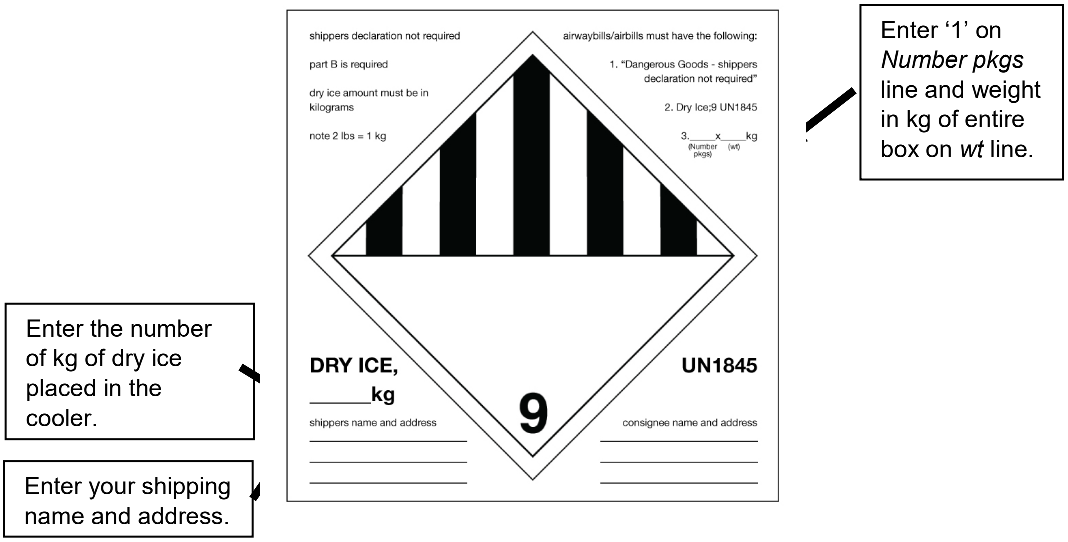

- Complete the FedEx Intra-Canada Air waybill with the CAPTURE ALS account number. CAPTURE ALS will be charged for the shipment.
  - Section 1/From: Complete Date, Sender's Name & Shipping Address
  - Section 2/Internal Reference: CAPTURE ALS, ZAEXK
  - Section 3/To: Enter Recipient's Name & Address
    - Recipient Name: C-BIG (M.N. Boivin)
    - Phone: 514-398-8439
    - Company: CBIG MNI -MCGILL U
    - Address (line 1): Neuro Immunology
    - Address (line 2): 3801 Rue University, Room NWB150
    - City: Montreal
    - Province: QC
    - Postal Code: H3A 2B4
  - Section 4/Shipment Information: Complete number packages, total weight and value.
  - Section 5a/Express Package Services: Check "FedEx Priority Overnight"
  - Section 6/Packaging: Check "Other", write "Insulated Shipper"
  - Section 7/Special Handling:
    - Check "YES - Shippers Declaration Not Required"
    - Check "Dry Ice" and enter the total weight of dry ice (10lbs).
  - Section 8/Payment: Check "Third Party", FedEx Acct No. 111266182

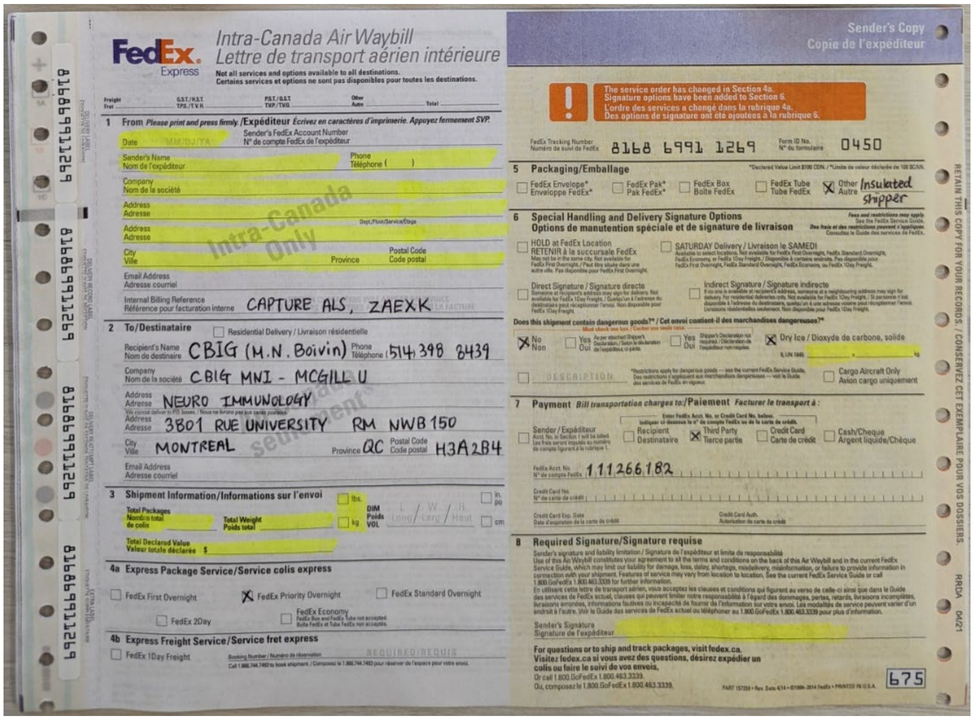
FedEx Express
Intra-Canada Air Waybill
Lettre de transport aérien intérieure
Sender's Copy
Copie de l'expéditeur

FedEx Tracking Number: 8168 6991 1269 — Form ID No.: 0450

1 From / Expéditeur
Internal Billing Reference: CAPTURE ALS, ZAEXK

2 To / Destinataire
Recipient's Name: CBIG (M.N. Boivin) — Phone: 514 398 8439
Company: CBIG MNI - MCGILL U
Address: NEURO IMMUNOLOGY
Address: 3801 RUE UNIVERSITY RM NWB 150
City: MONTREAL — Province: QC — Postal Code: H3A2B4

3 Shipment Information / Informations sur l'envoi

4a Express Package Service / Service colis express
☒ FedEx Priority Overnight

4b Express Freight Service / Service fret express

5 Packaging / Emballage
☒ Other / Autre: Insulated shipper

6 Special Handling and Delivery Signature Options
☒ No / Non
☒ Dry Ice / Dioxyde de carbone, solide

7 Payment / Paiement
☒ Third Party / Tierce partie
FedEx Acct. No.: 111266182

8 Required Signature / Signature requise

675

- Schedule a pick-up time and drop off the box at the pick-up location
- Email the biobank at cbig.mni@mcgill.ca or call the Biobank at 514-398-8439 to let them know which samples are being sent as well as the tracking number.

7.11.5. Ambient Sample Shipments (Credo)

Green Top Blood Collection Tubes (GTTs; for PBMC isolation) are shipped at controlled ambient temperate to C-BIG in a credo cube.

7.11.5.1. Ambient Sample Shipping Supplies

1. GTTs for PBMC Isolation
2. Elastic
3. Parafilm
4. Biohazard specimen transport bag
5. Absorbent Pouch
6. Safe T Pak Leak proof bag and Tvek outer envelope
7. Credo Box
8. Packing Tape

7.11.5.2. Ambient Sample Packing Instructions

- The GTTs should be kept at room temperature and sent on the same day as collection.
- A single credo cube can contain PBMC collections from two (2) participants collected on the same day. The following steps should be followed for each participant.
- Complete the packing slip (Appendix 8.9) and place it in the shipping container. Retain a copy for site records. Include a copy of the Biosample Collection Form [Blood] (Appendix 8.1) to facilitate sample processing at C-BIG.
- Place parafilm on the lids of the GTTs that have been filled.
- Place up to 6 GTTs, from a single participant, inside the absorbent Aqui-Pak pouch.
- Roll the absorbent pouch and attach it with an elastic.
- Place the absorbent protected vacutainers in the biohazard bag.
- Place the biohazard bag containing vacutainers in the Saf T Pak leak-proof poly bag (part #1 of STP-710) and seal.
- Place the leak-proof poly bag in the Tyvek outer envelope and seal (part #2 of STP-710) and seal.
- Repeat steps above as necessary for additional GTTs.
- Place the biohazard envelope in the Credo Cube.
- Seal the cardboard shipping box with standard packing tape.

7.11.5.3. Ambient Sample Shipping Instructions

- Affix the ‘Substance B’ shipping label to the outside of the cardboard shipping box.
- Complete the FedEx Intra-Canada Air waybill with the CAPTURE ALS account number. CAPTURE ALS will be charged for the shipment.
    - Section 1/From: Complete Date, Sender’s Name & Shipping Address

- Section 2/Internal Reference: CAPTURE ALS, ZAEXK
- Section 3/To: Enter Recipient's Name & Address
    - Recipient Name: C-BIG (M.N. Boivin)
    - Phone: 514-398-8439
    - Company: CBIG MNI -MCGILL U
    - Address (line 1): Neuro Immunology
    - Address (line 2): 3801 Rue University, Room NWB150
    - City: Montreal
    - Province: QC
    - Postal Code: H3A 2B4
- Section 4/Shipment Information: Complete number packages, total weight and value.
- Section 5a/Express Package Services: Check "FedEx Priority Overnight"
- Section 6/Packaging: Check "Other", write "Insulated Shipper"
- Section 7/Special Handling:
    - Check "NO"
- Section 8/Payment: Check "Third Party", FedEx Acct No. 111266182

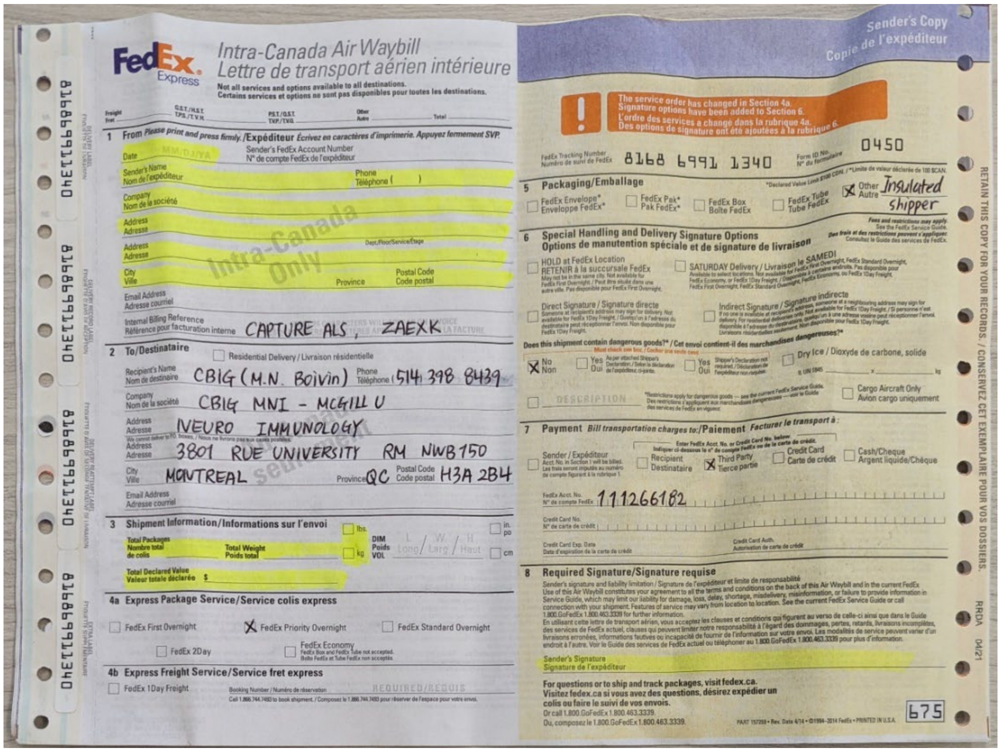

- Schedule a pick-up time and drop off the box at the pick-up location
- Email the biobank at cbig.mni@mcgill.ca or call the Biobank at 514-398-8439 to let them know which samples are being sent as well as the tracking number.

7.11.5.4. Credo Return Shipping Instructions

- C-BIG will return credo boxes to each site using FedEx Ground and Acct No. 111266182.
- Ensure that “Collect” is checked off on the Ground waybill when items are shipped to or from the University of Alberta.

8. APPENDICES

Appendix 8.1: CAPTURE ALS Biosample Collection Form [Blood]

Appendix 8.2: CAPTURE ALS Biosample Collection Form [CSF]

Appendix 8.3: Sample Processing Form - Serum

Appendix 8.4: Sample Processing Form - PBMC via Leucosep

Appendix 8.5: Sample Processing Form - Plasma

Appendix 8.6: Sample Processing Form - RNA

Appendix 8.7: Sample Processing Form - CSF

Appendix 8.8: Biosample Labels Template

Appendix 8.9: CAPTURE ALS Packing Slip

Appendix 8.1: CAPTURE ALS Biosample Collection Form [Blood]

Found in [Biosample Collection Form [Blood] (7Mar2022) CAPTURE ALS]

CAPTURE ALS

Biosample Collection Form [Blood]

**SECTION 1. PARTICIPANT**	
**CONTROL ☐ PATIENT ☐**	**CAPTURE-ALS ID:** ***CAPT*** __ __ __ __ __ __ __

**SECTION 2. BASIC DEMOGRAPHIC INFORMATION**	
**SEX: MALE ☐ FEMALE ☐**	**Date of birth:** MM / YYYY

**SECTION 3. STUDY INFORMATION**				
**Study Name: CAPTURE-ALS**				
**Visit Month:**	**0 ☐**	**4 ☐**	**8 ☐**	**12 ☐**
**Site:**	**Edmonton ☐**	**Montreal ☐**	**Toronto ☐**	**Quebec City ☐**
**Current Diagnosis:**	**ALS ☐**	**ALS-related disease ☐**	**Healthy Control ☐**	**Other ☐** : ____________

SECTION 4. BLOOD DRAW

	**VACCUTAINERS**				
	**Purpose**	**Color**	**Type**	**# Requested**	**# Drawn**
**[Patient Participant]**<br>**0 Month (Total tubes:12)**<br>*2* ***RTT*** *(Serum),* ***5 GTT*** *(PBMC),****3 PTT*** *(Plasma (1), DNA (2)),* ***2 PAXgene*** *(RNA)*<br>**4, 8, 12 Month (Total tubes :3)**<br>**2 RTT** (Serum), **1 PTT** (Plasma)	Serum	Red	No Additive	2	
	PBMC	Green	Heparin	5	
**[Healthy Control Participant]**<br>**0 Month (Total tubes: 5 / visit)**<br>***2 RTT*** *(Serum),* ***1 PTT*** *(Plasma),* ***2 PAXgene*** *(RNA)*<br>**8 Month (Total tubes : 3 / visit)**<br>***2 RTT*** *(Serum),* ***1 PTT*** *(Plasma)*	Plasma	Purple	K_2EDTA	1	
	DNA	Purple	K_2EDTA	2	
	RNA		PAXgene	2	

Recommended Order of Blood Draw: RTT, GTT, PTT, PAXgene

**Drawn by:**	**Draw Date:** DD / MM / YYYY	**Draw Time:** hh / mm

Appendix 8.2: CAPTURE ALS Biosample Collection Form [CSF]

Found in [Biosample Collection Form [CSF] (7Mar2022) CAPTURE ALS]

CAPTURE ALS

Biosample Collection Form [CSF]

SECTION 1. PARTICIPANT	
PATIENT ☐	CAPTURE-ALS ID: *CAPT*__ __ __ __ __ __ __

SECTION 2. BASIC DEMOGRAPHIC INFORMATION	
SEX: MALE ☐ FEMALE ☐	Date of birth: MM / YYYY

SECTION 3. STUDY INFORMATION				
Study Name: CAPTURE-ALS				
Visit Month:	0 ☐	4 ☐	8 ☐	12 ☐
Site:	Edmonton ☐	Montreal ☐	Toronto ☐	Quebec City ☐
Current Diagnosis:	ALS ☐	ALS-related disease ☐	Healthy Control ☐	Other ☐ : ____________

SECTION 4. CSF DRAW				
☐ PT/PTT/INR/CBC levels verified by a physician prior to LP				
A licensed practitioner will collect **up to 16mL** of CSF.<br><br>Must be collected in **polypropylene** tubes<br>No additives used.<br><br>1 tube **(1-2 mL)** goes to local lab for analysis of cell count, protein, glucose.	**VACCUTAINERS**			
	**Purpose**	**Type**	**# Requested**	**# Drawn**
	CSF	Polypropylene tubes	2	
Immediately after collection, keep the CSF at 4ºC				
**LP performed by:**<br>NAME / SIGNATURE	**Draw Date:**<br>DD / MM / YYYY		**Draw Time:**<br>hh / mm	
☐ 1 tube of CSF (1-2 mL) sent to local lab for analysis<br>☐ CSF collected for CAPTURE-ALS (volume of CSF collected: ______________ **mL)**				
**Note** *(did the participant experience any adverse event, etc.)*:				

Appendix 8.3: Sample Processing Form - Serum

Found in [Sample Processing Form – Serum (7Mar2022) CAPTURE ALS]

Serum Isolation from Whole Blood
Sample Processing Form: Serum
Last updated: 7March2022

**SERUM PROCESSING**			**SOP Employed:** CBIG-02-003
Processed by: ________________			CAPT ID: _____
Time start processing: __:__		Date: ___/____/_____	

**VACCUTAINERS/TUBES**			**QUANTITY**		
**RECEIVED**	**TYPE**	**RECEIVED**	**ALIQUOTS**		
__	___	_____**mL**	___ x **500µL**		
**Issues**	☐ Milky serum	☐ Hemolyzed, if checked, score (0-4)		☐ Expired tubes	Date of expiry: __ /
**RECEIVED**	**TYPE**	**RECEIVED**	**ALIQUOTS**		
__	___	____**mL**	___ x **500µL**		
**Issues**	☐ Milky Serum	☐ Hemolyzed, if checked, score (0-4)		☐ Expired tubes	Date of expiry: /

**STEPS:**			
☐	1. Keep tube upright at room temperature for a minimum of 30min and a maximum of 120 min to allow blood to clot		
☐	2. Centrifuge 10 minutes at 2500Xg	Start:__:__	End:__:__
☐	3. Transfer SN to a new 15 mL Falcon tube		
☐	4. Prepare aliquots (500 µL)		
☐	5. Store at -80°C (until ship to C-BIG)	Date: ___/____/_____	

**REAGENTS TRACKING**				
	Lot #	Expiry Date	Opened	Finished
Vacutainers	_____	___/_____	N/App	N/App

Notes:

Adapted from C-BIG Serum Sample Processing Form, Dated: April 22, 2020

Appendix 8.4: Sample Processing Form - PBMC via Leucosep

Found in [Sample Processing Form – PBMC vis Leucosep (7Mar2022) CAPTURE ALS]

PBMC Isolation from Whole Blood (Leucosep Method)
Sample Processing Form: PBMC via Leucosep
Last updated: 7March2022

**PBMC PROCESSING**				**SOP Employed:** CBIG-02-002
Processed by: ________________			CAPT ID: _____	
Time start processing: __:__			Date: __/__/____	
**VACCUTAINERS/TUBES**		**QUANTITY**		
**RECEIVED**	**TYPE**	**RECEIVED**		
___	___	_____mL		
**Issues**	☐ Red Pellet (RP)	☐ Clotted	☐ Expired tubes Date of expiry: ___/_____	
**STEPS:**				
☐	1. Distribute 15 mL of Ficoll into the Leucosep Tube			
☐	2. Centrifuge for 30 seconds at 1000Xg			
☐	3. Distribute the blood from the vacutainers and dilute 1:2 with PBS into the Leucosep Tube (up to 35 mL)			
☐	4. Centrifuge for 10 minutes at 1000Xg, with NO break	Start:__:__	End:__:__	
☐	5. Collect PBMC by pouring into a new 50 mL tube and complete suspension to 50 mL with PBS			
☐	6. Centrifuge for 10 minutes at 250Xg	Start:__:__	End:__:__	
☐	7. Discard supernatant, loosen pellet, add 10 mL of PBS			
☐	8. Count cell			
☐	9. Take cell if needed for trizol and complete suspension to 50 mL with PBS			
☐	10. Centrifuge for 10 minutes at 250Xg	Start:__:__	End:__:__	
☐	11. Discard supernatant and loosen cell pellet			
☐	12. Add solution A (HI HuAB serum)			
☐	13. Add solution B (HI HuAB serum + DMSO)			
☐	Prepare aliquots	Date: __/__/____		
☐	Store at -80°C	Date: __/__/____		
☐	Store in liquid nitrogen	Date: __/__/____		
**PBMC COUNTING**				**SOP Employed: CBIG-03-001**
Dilution factor :**2**		Cell suspension volume:**10.00mL**		
Countess FL II (already take into account the dilution factor)		**Count #1**	_____	**x10⁶ cells/ mL**
		**Count #2**	_____	**x 10⁶ cells/mL**
SUM OF COUNTS: Count #1 + Count #2	_____	CELL COUNTING MEAN: Sum of Count #1 and Count #2 / 2	_____	
TOTAL CELLS ISOLATED : Cell Counting Mean x Cell Suspension Volume (mL)				
Coefficient of Variance (% C.V.) (need to be under 5%)	_____	Live:____ Dead:____	Live:____ Dead:____	**Total PBMC** _____ **x10⁶**
			☐If trizol done take:	_____**x 10⁶ cells**
			**Final PBMC count (after trizol)**	_____**x 10⁶ cells**

Adapted from C-BIG PBMC via Leucosep Sample Processing Form, Dated: April 22, 2020

PBMC FREEZING			
FREEZING SOLUTION B PREPATION (mL)<br>HuAB serum + 20% DMSO	HuAB: _____**mL**<br>DMSO: _____**mL**	NUMBER OF VIALS	__
TOTAL FREEZING SOLUTION VOLUME USED (mL)<br>50% Solution A (HuAB serum) + 50% Solution B<br>Total Volume = 1.00mL x Number of Vials	_____**mL**	CELLS PER VIAL<br>Total Cells Isolated / Number of Vials<br>**SHOULD NOT EXCEED 21.50 x10^6	_____**x10^6**

QUANTITY - ALIQUOTS

___ X vials at _____ x 10^6 cells / mL in each vial

REAGENTS TRACKING

	Lot #	Expiry Date	Opened	Finished
Leucosep	_____	___/_____	N/App	N/App
PBS	_____	___/_____		
Ficoll	_____	___/_____		
HI HuAB serum	_____	___/_____		
DMSO	_____	- / -		
Vacutainers	_____	___/_____	N/App	N/App

Notes:

Appendix 8.5: Sample Processing Form - Plasma

Found in [Sample Processing Form – Plasma (7Mar2022) CAPTURE ALS]

Plasma Isolation from Whole Blood
Sample Processing Form: Plasma
Last updated: 7March2022

**PLASMA PROCESSING**			**SOP Employed:** CBIG-02-011
Processed by: ________________		CAPT ID: _____	
Time start processing: __:__		Date: __/____/_____	
**VACCUTAINERS/TUBES**		**QUANTITY**	
**RECEIVED**	**TYPE**	**RECEIVED**	
___	___	_____**mL**	

**Concerns**	☐ Expired tubes Date of expiry: ___/_____

**STEPS**			
☐	1. Store PTT in the dark and upright for 30 min	Start:__:__	End:__:__
☐	2. Centrifuge for 12 minutes at 250Xg at 4°C	Start:__:__	End:__:__
☐	3. Transfer supernatant into a new conical tube		
☐	4. Prepare aliquots (500 µL)		
☐	Store at -80°C (until ship to C-BIG)	Date: __/____/_____	

**QUANTITY - ALIQUOTS**
___ X vials at 500 µL

**REAGENTS TRACKING**				
	Lot #	Expiry Date	Opened	Finished
Vacutainers	_____	___/_____	N/App	N/App

Notes:

Adapted from C-BIG Plasma Sample Processing Form, Dated: August 25, 2020

Appendix 8.6: Sample Processing Form - RNA

Found in [Sample Processing Form – RNA (7Mar2022) CAPTURE ALS]

RNA Processing
Sample Processing Form: RNA
Last updated: 7March2022

**RNA PROCESSING**			**SOP employed: CAPT-01-012**
Processed by: ________________		CAPT ID: _____	
Time collected: __:__		Date: ___/____/_____	
**VACCUTAINERS/TUBES**		**QUANTITY**	
**RECEIVED**	**TYPE**	**RECEIVED**	
__	___	_____mL	
**Issues** ☐	☐	☐ Expired tubes Date of expiry: ___/_____	

**STEPS:**	
☐	1. Keep tubes upright at room temperature for a minimum of 2hours and a maximum of 72hours to allow blood to clot.
☐	2. Transfer tubes to -20°C for at least 24hours.
☐	3. Transfer tubes to -80°C freezer (until ship to C-BIG) Date: ___/____/_____

**REAGENTS TRACKING**				
		Expiry Date	Opened	Finished
Vacutainers	_____	___/____	N/App	N/App

Notes:

Appendix 8.7: Sample Processing Form – CSF

Found in [Sample Processing Form - CSF (7Mar2022) CAPTURE ALS]

CSF Processing
Sample Processing Form: CSF
Last updated: 7March2022

**CSF PROCESSING (SUPERNATANT)**				**SOP Employed:** CBIG-02-008
Processed by: ________			CAPT ID: ____	
Time start processing: __:__			Date: __/__/__	
**VACCUTAINERS/TUBES**		**QUANTITY**		
**RECEIVED**	**TYPE**	**RECEIVED**		
__	___	____mL		
**Issues**	☐ Blood contamination	☐ Not kept at 4^{0}C immediately after lumbar puncture	☐ Expired tubes Date of expiry: ___/____	
**STEPS:**				
☐	1. Transfer the CSF to a new 15 mL Falcon tube			
☐	2. Centrifuge for 10 minutes at 2000Xg	Start:__:__	End:__:__	
☐	3. Remove supernatant, and put it in a new 15 mL Falcon tube			
☐	4. Remove supernatant and aliquot it.			
☐	5. Prepare aliquots (500 µL)	Date: __/__/__		
☐	6. Store at -80°C (until ship to C-BIG)	Date: __/__/__		
**QUANTITY - ALIQUOTS**				
____ x **500µL**				

Notes:

Adapted from C-BIG CSF Sample Processing Form, Dated: April 22, 2020

Page **1** of **1**

Appendix 8.8: Biosample Labels Template

Found in [Biosample Labels Template (EN, V2, 26Oct2022) CAPTURE ALS]

PSCID	Vial #	Type	VisitLabel	CollectionDate	Barcode
CAPTXXXXXXX	201	Serum	00M	DD/MM/20XX	CAPTXXXXXXX201
CAPTXXXXXXX	202	Serum	00M	DD/MM/20XX	CAPTXXXXXXX202
CAPTXXXXXXX	203	PBMC	00M	DD/MM/20XX	CAPTXXXXXXX203
CAPTXXXXXXX	204	PBMC	00M	DD/MM/20XX	CAPTXXXXXXX204
CAPTXXXXXXX	205	PBMC	00M	DD/MM/20XX	CAPTXXXXXXX205
CAPTXXXXXXX	206	PBMC	00M	DD/MM/20XX	CAPTXXXXXXX206
CAPTXXXXXXX	207	PBMC	00M	DD/MM/20XX	CAPTXXXXXXX207
CAPTXXXXXXX	208	DNA	00M	DD/MM/20XX	CAPTXXXXXXX208
CAPTXXXXXXX	209	DNA	00M	DD/MM/20XX	CAPTXXXXXXX209
CAPTXXXXXXX	210	Plasma	00M	DD/MM/20XX	CAPTXXXXXXX210
CAPTXXXXXXX	211	CSF	00M	DD/MM/20XX	CAPTXXXXXXX211
CAPTXXXXXXX	212	CSF	00M	DD/MM/20XX	CAPTXXXXXXX212
CAPTXXXXXXX	213	RNA	00M	DD/MM/20XX	CAPTXXXXXXX213
CAPTXXXXXXX	214	RNA	00M	DD/MM/20XX	CAPTXXXXXXX214
CAPTXXXXXXX	215	Serum	00M	DD/MM/20XX	CAPTXXXXXXX215
CAPTXXXXXXX	216	Serum	00M	DD/MM/20XX	CAPTXXXXXXX216
CAPTXXXXXXX	217	Serum	00M	DD/MM/20XX	CAPTXXXXXXX217
CAPTXXXXXXX	218	Serum	00M	DD/MM/20XX	CAPTXXXXXXX218
CAPTXXXXXXX	219	Serum	00M	DD/MM/20XX	CAPTXXXXXXX219
CAPTXXXXXXX	220	Serum	00M	DD/MM/20XX	CAPTXXXXXXX220
CAPTXXXXXXX	221	Serum	00M	DD/MM/20XX	CAPTXXXXXXX221
CAPTXXXXXXX	222	Serum	00M	DD/MM/20XX	CAPTXXXXXXX222
CAPTXXXXXXX	223	Serum	00M	DD/MM/20XX	CAPTXXXXXXX223
CAPTXXXXXXX	224	Serum	00M	DD/MM/20XX	CAPTXXXXXXX224
CAPTXXXXXXX	225	Serum	00M	DD/MM/20XX	CAPTXXXXXXX225
CAPTXXXXXXX	226	Serum	00M	DD/MM/20XX	CAPTXXXXXXX226
CAPTXXXXXXX	227	Serum	00M	DD/MM/20XX	CAPTXXXXXXX227
CAPTXXXXXXX	228	Serum	00M	DD/MM/20XX	CAPTXXXXXXX228
CAPTXXXXXXX	229	Serum	00M	DD/MM/20XX	CAPTXXXXXXX229
CAPTXXXXXXX	230	Plasma	00M	DD/MM/20XX	CAPTXXXXXXX230
CAPTXXXXXXX	231	Plasma	00M	DD/MM/20XX	CAPTXXXXXXX231
CAPTXXXXXXX	232	Plasma	00M	DD/MM/20XX	CAPTXXXXXXX232
CAPTXXXXXXX	233	Plasma	00M	DD/MM/20XX	CAPTXXXXXXX233
CAPTXXXXXXX	234	Plasma	00M	DD/MM/20XX	CAPTXXXXXXX234
CAPTXXXXXXX	235	Plasma	00M	DD/MM/20XX	CAPTXXXXXXX235
CAPTXXXXXXX	236	Plasma	00M	DD/MM/20XX	CAPTXXXXXXX236
CAPTXXXXXXX	237	Plasma	00M	DD/MM/20XX	CAPTXXXXXXX237
CAPTXXXXXXX	238	Plasma	00M	DD/MM/20XX	CAPTXXXXXXX238
CAPTXXXXXXX	239	Plasma	00M	DD/MM/20XX	CAPTXXXXXXX239
CAPTXXXXXXX	240	CSF	00M	DD/MM/20XX	CAPTXXXXXXX240
CAPTXXXXXXX	241	CSF	00M	DD/MM/20XX	CAPTXXXXXXX241
CAPTXXXXXXX	242	CSF	00M	DD/MM/20XX	CAPTXXXXXXX242
CAPTXXXXXXX	243	CSF	00M	DD/MM/20XX	CAPTXXXXXXX243
CAPTXXXXXXX	244	CSF	00M	DD/MM/20XX	CAPTXXXXXXX244
CAPTXXXXXXX	245	CSF	00M	DD/MM/20XX	CAPTXXXXXXX245
CAPTXXXXXXX	246	CSF	00M	DD/MM/20XX	CAPTXXXXXXX246
CAPTXXXXXXX	247	CSF	00M	DD/MM/20XX	CAPTXXXXXXX247
CAPTXXXXXXX	248	CSF	00M	DD/MM/20XX	CAPTXXXXXXX248
CAPTXXXXXXX	249	CSF	00M	DD/MM/20XX	CAPTXXXXXXX249
CAPTXXXXXXX	250	CSF	00M	DD/MM/20XX	CAPTXXXXXXX250
CAPTXXXXXXX	251	CSF	00M	DD/MM/20XX	CAPTXXXXXXX251
CAPTXXXXXXX	252	CSF	00M	DD/MM/20XX	CAPTXXXXXXX252
CAPTXXXXXXX	253	CSF	00M	DD/MM/20XX	CAPTXXXXXXX253
CAPTXXXXXXX	254	CSF	00M	DD/MM/20XX	CAPTXXXXXXX254
CAPTXXXXXXX	255	CSF	00M	DD/MM/20XX	CAPTXXXXXXX255
CAPTXXXXXXX	256	CSF	00M	DD/MM/20XX	CAPTXXXXXXX256
CAPTXXXXXXX	257	CSF	00M	DD/MM/20XX	CAPTXXXXXXX257
CAPTXXXXXXX	258	CSF	00M	DD/MM/20XX	CAPTXXXXXXX258
CAPTXXXXXXX	259	CSF	00M	DD/MM/20XX	CAPTXXXXXXX259
CAPTXXXXXXX	260	CSF	00M	DD/MM/20XX	CAPTXXXXXXX260
CAPTXXXXXXX	261	CSF	00M	DD/MM/20XX	CAPTXXXXXXX261
CAPTXXXXXXX	262	CSF	00M	DD/MM/20XX	CAPTXXXXXXX262
CAPTXXXXXXX	263	CSF	00M	DD/MM/20XX	CAPTXXXXXXX263
CAPTXXXXXXX	264	CSF	00M	DD/MM/20XX	CAPTXXXXXXX264
CAPTXXXXXXX	265	CSF	00M	DD/MM/20XX	CAPTXXXXXXX265
CAPTXXXXXXX	266	CSF	00M	DD/MM/20XX	CAPTXXXXXXX266
CAPTXXXXXXX	267	CSF	00M	DD/MM/20XX	CAPTXXXXXXX267
CAPTXXXXXXX	268	CSF	00M	DD/MM/20XX	CAPTXXXXXXX268
CAPTXXXXXXX	269	CSF	00M	DD/MM/20XX	CAPTXXXXXXX269
CAPTXXXXXXX	270	CSF	00M	DD/MM/20XX	CAPTXXXXXXX270
CAPTXXXXXXX	271	CSF	00M	DD/MM/20XX	CAPTXXXXXXX271
CAPTXXXXXXX	272	CSF	00M	DD/MM/20XX	CAPTXXXXXXX272
CAPTXXXXXXX	273	CSF	00M	DD/MM/20XX	CAPTXXXXXXX273
CAPTXXXXXXX	274	CSF	00M	DD/MM/20XX	CAPTXXXXXXX274
CAPTXXXXXXX	275	CSF	00M	DD/MM/20XX	CAPTXXXXXXX275
CAPTXXXXXXX	276	CSF	00M	DD/MM/20XX	CAPTXXXXXXX276
CAPTXXXXXXX	277	CSF	00M	DD/MM/20XX	CAPTXXXXXXX277
CAPTXXXXXXX	278	CSF	00M	DD/MM/20XX	CAPTXXXXXXX278
CAPTXXXXXXX	279	CSF	00M	DD/MM/20XX	CAPTXXXXXXX279
CAPTXXXXXXX	280	CSF	00M	DD/MM/20XX	CAPTXXXXXXX280
CAPTXXXXXXX	281	CSF	00M	DD/MM/20XX	CAPTXXXXXXX281
CAPTXXXXXXX	282	CSF	00M	DD/MM/20XX	CAPTXXXXXXX282
CAPTXXXXXXX	283	CSF	00M	DD/MM/20XX	CAPTXXXXXXX283
CAPTXXXXXXX	284	CSF	00M	DD/MM/20XX	CAPTXXXXXXX284

Appendix 8.9: CAPTURE ALS Packing Slip

Found in [Sample Packing Slip (12July2022) CAPTURE ALS]

CAPTURE ALS

Biosample Packing Slip

Site Name: ____________________________________

Date Shipped: ___ ___/ ___ ___/ ___ ___ ___ ___
D D M M Y Y Y Y

Page ____ of ____

Shipped By (Full Name): ________________________________

Participant CAPTURE ID	Visit (Month)	Date Collected (dd/mm/yyyy)	# plasma vials	# serum vials	# CSF vials	# PTT (DNA)	# GTT (PBMC)	# PAXgene tubes (RNA)

PTT: Purple Top Tube, GTT: Green Top Tube

Retain one copy on site, and provide a second copy in the shipping container for use at C-BIG Repository
