# Supplementary material for: Comprehensive analysis platform to understand, remedy, and eliminate amyotrophic lateral sclerosis (CAPTURE ALS): Study protocol for a Canadian multicenter, multimodal, longitudinal observational study
Source: PLoS One. 2025 Dec 4;20(12):e0332430. doi: 10.1371/journal.pone.0332430 (PMC12677780; doi:10.1371/journal.pone.0332430)
Supplement: S1 Table — (DOCX) [file pone.0332430.s010.docx]

**S1 Table. Full List of Features Assessed in Neurological Examination.**

|  | **Assessment** | **Severity/Scale** |
| --- | --- | --- |
| **Face** | Fasciculations | Yes / No |
|  | Weakness | Yes / No |
| **Tongue** | Fasciculations | Yes / No |
|  | Atrophy | Yes / No |
|  | Weakness | Yes / No |
|  | Movements | Normal / Slow / No movement |
| **Dysarthria** | Dysarthria | 0 = None  1 = Mild  2 = Moderate  3 = Severe (unintelligible or anarthric) |
| **Fasciculations** | Trunk | Yes / No |
|  | Arm | Yes / No |
|  | Leg | Yes / No |
| **Atrophy** | Arm | Yes / No |
|  | Leg | Yes / No |
| **Spasticity** | Upper Extremities  (Forearm Supination / Pronation) | Modified Ashworth Scale  0 = No increase in tone  1 = Slight increase: catch and release OR minimal resistance at end of ROM  1+ = Slight increase: catch and release followed by minimal resistance throughout the remainder (<½) of ROM  2 = Moderate increase in tone, but passive movement easy  3 = Marked increase in tone, passive movement difficult  4 = Rigid and immobile |
|  | Lower Extremities  (Knee Flexion) |  |
| **Power** | Neck Extension | 0 = No contraction  1 = Flicker or trace of contraction  2 = Active movement without gravity  3 = Active movement against gravity  4- = Active movement against minimal resistance  4 = Active movement against moderate resistance  4+ = Active movement against strong resistance  5 = Normal power |
|  | Neck Flexion |  |
|  | Infraspinatus |  |
|  | Deltoids |  |
|  | Biceps |  |
|  | Triceps |  |
|  | Wrist Extensors |  |
|  | Wrist Flexors |  |
|  | Extensor Digitorum Communis (EDC) |  |
|  | First Dorsal Interosseous (FDI) |  |
|  | Abductor Digiti Minimi (ADM) |  |
|  | Abductor Pollicis Brevis (APB) |  |
|  | Iliopsoas |  |
|  | Hip Abductors |  |
|  | Quadriceps |  |
|  | Hamstrings |  |
|  | Ankle Dorsiflexors |  |
|  | Ankle Plantarflexors |  |
|  | Extensor Hallucis Longus (EHL) |  |
| **Muscle Stretch Reflexes** | Jaw Jerk | 0 = Absent  1 = Present  2 = Clonus |
|  | Biceps | 0 = Absent  1 = Decreased, or seen only with reinforcement  2 = Normal  3 = Brisk, may have spread  4 = Brisk with clonus |
|  | Brachioradialis |  |
|  | Triceps |  |
|  | Quadriceps |  |
|  | Triceps Surae |  |
| **Other Reflexes** | Hoffman’s | 0 = Absent  1 = Present  5 = Not done |
|  | Superficial abdominal |  |
|  | Babinski |  |
| **Pseudobulbar Affect** | Pseudobulbar affect | Yes / No |
| **Cerebellar Dysfunction** | Arm (finger – nose ataxia) | Yes / No / Unable (too weak for reliable assessment) |
|  | Leg (heel – shin ataxia) |  |
| **Gait** | Gait | Normal  Abnormal  Abnormal-Spastic  Abnormal-Ataxic  Unable to walk without aids |
