# Supplementary material for: Comprehensive analysis platform to understand, remedy, and eliminate amyotrophic lateral sclerosis (CAPTURE ALS): Study protocol for a Canadian multicenter, multimodal, longitudinal observational study
Source: PLoS One. 2025 Dec 4;20(12):e0332430. doi: 10.1371/journal.pone.0332430 (PMC12677780; doi:10.1371/journal.pone.0332430)
Supplement: S2 Table — (DOCX) [file pone.0332430.s011.docx]

**S2 Table. Details of the Neurocognitive Battery (affected participants).**

| Affected Participants | Visit 1 | Visit 2 | Visit 3 | Visit 4 | Visit 5 |
| --- | --- | --- | --- | --- | --- |
|  | -1 | 0 month | 4 months | 8 months | 12 months |
|  | Screening | Baseline |  |  |  |
| Semantic Fluency + Abrahams Correction |  | X (Animal) | X (Fruit) | X (Vegetable) | X (Animal) |
| Boston Naming Test-II (BNT-II) |  | X (Short) |  | X (Long) |  |
| Hopkins Verbal Learning Test (HVLT-R) |  | X (Form 1) |  | X (Form 2) |  |
| Social Norms Questionnaire |  | X |  | X |  |
| Hospital Anxiety and Depression Scale (HADS) |  | X |  | X |  |
| Center for Neurologic Study-Lability Scale (CNS-LS) |  | X |  | X |  |
| Frontal Systems Behavior Scale (FrSBe) (Self-Rating form) |  | X |  | X |  |
| Stroop test (Victoria version) |  | X |  | X |  |
| Judgment of Line Orientation (JLO) |  | X (Form V) |  | X (Form H) |  |
