# Supplementary material for: Comprehensive analysis platform to understand, remedy, and eliminate amyotrophic lateral sclerosis (CAPTURE ALS): Study protocol for a Canadian multicenter, multimodal, longitudinal observational study
Source: PLoS One. 2025 Dec 4;20(12):e0332430. doi: 10.1371/journal.pone.0332430 (PMC12677780; doi:10.1371/journal.pone.0332430)
Supplement: S4 Table — (DOCX) [file pone.0332430.s013.docx]

**S4 Table. Details of Neurocognitive Testing Battery.**

| **Instrument** | **Domain** |
| --- | --- |
| Montreal Cognitive Assessment (MoCA) | Short‐term memory, visuospatial function, executive function, attention, concentration and working memory, language, and orientation [1]. |
| Edinburgh Cognitive and Behavioural ALS Screen (ECAS) [2] | Executive functions, social cognition, verbal fluency and language (ALS-specific), but also memory and visuospatial abilities (Non ALS-specific) [3]. |
| Semantic Fluency + Abrahams Correction [4] |  |
| Boston Naming Test-II (BNT-II) | Compromised lexical retrieval abilities and aphasia through visual confrontation naming [5]. |
| Hopkins Verbal Learning Test (HVLT-R) | Verbal learning and memory [6]. |
| Social Norms Questionnaire | Degree to which a person understands and can accurately identify implicitly, but widely accepted social boundaries [7]. |
| Hospital Anxiety and Depression Scale (HADS) | Anxiety and depression in a general medical population of patients [8]. |
| Center for Neurologic Study-Lability Scale (CNS-LS) | Perceived frequency of pseudobulbar affect episodes. |
| FrSBe (Self-Rating form) | Behavior associated with damage to the frontal systems of the brain, with subscales measuring Apathy, Disinhibition, and Executive Dysfunction [9]. |
| Stroop test (Victoria version) | Executive functions such as cognitive flexibility, selective attention, cognitive inhibition, and information processing speed. Measures the ability with which an individual can maintain a goal and inhibit an automatic, overlearned response, in favor of a less familiar one [10]. |
| Judgment of Line Orientation (JLO) | Visuospatial judgment. Measures accuracy of angular orientation based on judgments about a pair of angled lines that visually match an identical pair immersed within a semicircular array of 11 lines [11]. |
| World Health Organization Quality of Life-BREF Scale (WHOQOL-BREF) | Self-report questionnaire which assesses 4 domains of quality of life (QOL): physical health, psychological health, social relationships, and environment. In addition, there are 2 items that measure overall QOL and general health [12]. |
| The Amyotrophic Lateral Sclerosis Assessment Questionnaire Short Form (ALSAQ-5) | Quality of life in ALS patients. |

**References:**

1. Davis, D. H.; Creavin, S. T.; Yip, J. L.; Noel-Storr, A. H.; Brayne, C.; Cullum, S. (2015) Montreal Cognitive Assessment for the diagnosis of Alzheimer's disease and other dementias. In : Cochrane Database Syst Rev, vol. 2015, n° 10, Cd010775. DOI: 10.1002/14651858.CD010775.pub2.
2. Abrahams, Sharon; Newton, Judith; Niven, Elaine; Foley, Jennifer; Bak, Thomas H. (2014) Screening for cognition and behaviour changes in ALS. In : Amyotrophic Lateral Sclerosis and Frontotemporal Degeneration, vol. 15, n° 1-2, p. 9–14. DOI: 10.3109/21678421.2013.805784.
3. Siciliano, M.; Trojano, L.; Trojsi, F.; Greco, R.; Santoro, M.; Basile, G. et al. (2017) Edinburgh Cognitive and Behavioural ALS Screen (ECAS)-Italian version: regression based norms and equivalent scores. In : Neurol Sci, vol. 38, n° 6, p. 1059–1068. DOI: 10.1007/s10072-017-2919-4.
4. Shao, Z.; Janse, E.; Visser, K.; Meyer, A. S. (2014) What do verbal fluency tasks measure? Predictors of verbal fluency performance in older adults. In : Front Psychol, vol. 5, p. 772. DOI: 10.3389/fpsyg.2014.00772.
5. Jefferson, A. L.; Wong, S.; Gracer, T. S.; Ozonoff, A.; Green, R. C.; Stern, R. A. (2007) Geriatric performance on an abbreviated version of the Boston naming test. In : Appl Neuropsychol, vol. 14, n° 3, p. 215–223. DOI: 10.1080/09084280701509166.
6. Belkonen, Stacy (2011) Hopkins Verbal Learning Test. In : Jeffrey S. Kreutzer, John DeLuca et Bruce Caplan, coord.: Encyclopedia of Clinical Neuropsychology. New York, NY: Springer New York, p. 1264–1265.
7. van den Berg, E.; Poos, J. M.; Jiskoot, L. C.; Montagne, B.; Kessels, R. P. C.; Franzen, S. et al. (2021) Impaired Knowledge of Social Norms in Dementia and Psychiatric Disorders: Validation of the Social Norms Questionnaire–Dutch Version (SNQ-NL). In : Assessment, vol. 29, n° 6, p. 1236–1247. DOI: 10.1177/10731911211008234.
8. Stern, Anna F. (2014) The Hospital Anxiety and Depression Scale. In : Occupational Medicine, vol. 64, n° 5, p. 393–394. DOI: 10.1093/occmed/kqu024.
9. Malloy, P.; Tremont, G.; Grace, J.; Frakey, L. (2007) The Frontal Systems Behavior Scale discriminates frontotemporal dementia from Alzheimer's disease. In : Alzheimers Dement, vol. 3, n° 3, p. 200–203. DOI: 10.1016/j.jalz.2007.04.374.
10. Tremblay, M. P.; Potvin, O.; Belleville, S.; Bier, N.; Gagnon, L.; Blanchet, S. et al. (2016) The Victoria Stroop Test: Normative Data in Quebec-French Adults and Elderly. In : Arch Clin Neuropsychol, vol. 31, n° 8, p. 926–933. DOI: 10.1093/arclin/acw029.
11. Irani, Farzin (2011) Judgment of Line Orientation. In : Jeffrey S. Kreutzer, John DeLuca et Bruce Caplan, coord.: Encyclopedia of Clinical Neuropsychology. New York, NY: Springer New York, p. 1372–1374.
12. World Health Organization Quality of Life- BREF (WHOQOL-BREF). SCIRE Professional.
