# Supplementary material for: Comprehensive analysis platform to understand, remedy, and eliminate amyotrophic lateral sclerosis (CAPTURE ALS): Study protocol for a Canadian multicenter, multimodal, longitudinal observational study
Source: PLoS One. 2025 Dec 4;20(12):e0332430. doi: 10.1371/journal.pone.0332430 (PMC12677780; doi:10.1371/journal.pone.0332430)
Supplement: S5 Table — (DOCX) [file pone.0332430.s014.docx]

**S5 Table. Summary of Biosample Processing.**

| **Sample Type** | **Processing Location** | **Procedure** | **Storage** |
| --- | --- | --- | --- |
| Whole Blood Serum Isolation | Locally at each site. | Collection is done with two 10 mL RTTs and centrifuged at room temperature for 10 minutes at 2500Xg using a centrifuge with a swinging bucket. Serum is aliquoted into 1.0 mL cryovials (0.5 mL aliquots). | -80°C freezer until they are ready to be shipped. |
| Whole Blood PBMC Isolation | C-BIG | After blood collection using five 10 mL GTTs (Sodium Heparin), they are inverted 8-10 times. | Vacutainers are kept at room temperature and shipped immediately, priority overnight at ambient temperature. |
| Whole Blood DNA Extraction | C-BIG | Blood collection is done using two 10 mL PTT (K_2_EDTA). | Stored vertically in a -80°C freezer until ready for shipment. |
| Whole Blood Plasma Isolation | Locally at each site. | Kept upright at room temperature for a minimum of 30 minutes. Then it will be centrifuged at 4°C for 12 minutes at 250Xg. Plasma is transferred to a new 15 mL polypropylene conical tube then aliquoted by 0.5 mL into the 1.0 mL cryovials using a micropipette. | -80°C freezer until shipment. |
| Whole Blood RNA Extraction | C-BIG | Tubes are inverted 8-10 times immediately. Tubes are kept upright at room temperature for a minimum of 2 hours and a maximum of 72 hours to allow blood to clot. | Moved to -20°C for at least 24 hours before transferring to -80°C freezer until ready for shipment. |
| Cerebrospinal Fluid (CSF) | Locally at each site. | CSF is immediately kept at 4°C or on ice until ready to process. 1-2 mL of CSF will be sent to local laboratory for cell count, protein and glucose analysis following local laboratory guidelines. CSF sample is transferred into a new 15 mL polypropylene tube, then centrifuged at room temperature for 10 minutes. CSF is transferred to a new 15 mL polypropylene tube. Sample is aliquoted by 0.5 mL into the 1.0 mL cryovials. | -80°C freezer. |
