# Supplementary material for: Comprehensive analysis platform to understand, remedy, and eliminate amyotrophic lateral sclerosis (CAPTURE ALS): Study protocol for a Canadian multicenter, multimodal, longitudinal observational study
Source: PLoS One. 2025 Dec 4;20(12):e0332430. doi: 10.1371/journal.pone.0332430 (PMC12677780; doi:10.1371/journal.pone.0332430)
Supplement: Attachment — Submitted filename: Response to Reviewers.pdf [file pone.0332430.s016.pdf]

Response to Reviewers

Manuscript: Comprehensive Analysis Platform to Understand, Remedy, and Eliminate Amyotrophic Lateral Sclerosis (CAPTURE ALS): Study protocol for a Canadian multicenter, multimodal, longitudinal observational study. [PONE-D-24-55157]

Dear PLOS ONE,

Thank you for the opportunity to revise and resubmit our manuscript "Comprehensive Analysis Platform to Understand, Remedy, and Eliminate Amyotrophic Lateral Sclerosis (CAPTURE ALS): Study protocol for a Canadian multicenter, multimodal, longitudinal observational study".

We appreciate the comments from the editors and reviewers. We have addressed each comment carefully and revised the manuscript accordingly.
The reviewers/editor's original comments are in bold/italics, followed by our response in plain text.

Journal Requirements:

1. Please ensure that your manuscript meets PLOS ONE's style requirements, including those for file naming. The PLOS ONE style templates can be found at https://journals.plos.org/plosone/s/file?id=wjVg/PLOSOne_formatting_sample_main_body.pdf and https://journals.plos.org/plosone/s/file?id=ba62/PLOSOne_formatting_sample_title_authors_affiliations.pdf

Thank you for providing the appropriate URLs. Our manuscript was reviewed for style requirements and file naming. It should now meet PLOS ONE's requirements.

2. We note that the grant information you provided in the 'Funding Information' and 'Financial Disclosure' sections do not match. When you resubmit, please ensure that you provide the correct grant numbers for the awards you received for your study in the 'Funding Information' section.

Thank you for letting us know. In the Funding Information section, "Brain Canada" was updated to "Fondation Brain Canada" as searched through the dropdown menu. "CIHR" was also modified to "Canadian Institutes of Health Research" from the dropdown menu.

Please note that the grant numbers for the awards are unavailable. The funding from "Fondation Brain Canada" was through a partnership with Regeneron Pharmaceuticals,

Alnylam Pharmaceuticals and ALS Canada. If these three funding partners should not be included under “Funding Information”, please let us know.

The Chris Snow/Calgary Flames Foundation and ALS Quebec/ALS Action Canada were received through philanthropic means.

The funding statement below is the statement that the CAPTURE ALS Executive Committee includes in their charter and what was agreed with the primary funder, Brain Canada. Please also note that the receipt of the awards was a result of many individuals. The initials included in the statement are those who received the funds to their institution, which were then dispersed as subawards to others as applicable. If the initials need to be adjusted or removed to properly reflect the funding, please do let us know. This is the updated version:

The CAPTURE ALS platform has been made possible with the financial support of Health Canada, through the Canada Brain Research Fund, an innovative partnership between the Government of Canada (through Health Canada) and Brain Canada (https://braincanada.ca/), and of the ALS Society of Canada (https://als.ca/), Alnylam Pharmaceuticals (https://www.alnylam.com/), Regeneron Pharmaceuticals (https://www.regeneron.com/), the Canadian Institutes of Health Research (CIHR) (https://cihr-irsc.gc.ca/e/193.html) (SK, YM), and the Chris Snow/Calgary Flames Foundation (SK). The views expressed herein do not necessarily represent the views of the Minister of Health or the Government of Canada. The onboarding of CHUM (Centre hospitalier de l’université de Montreal) has been made possible with financial support of the ALS Society of Quebec (https://sla-quebec.ca/en/) and ALS Action Canada (https://www.alsactioncanada.org/) (GM). The funders had no role in study design, data collection and analysis, decision to publish, or preparation of the manuscript. There was no additional external funding received for this study.

3. Thank you for stating in your Funding Statement.

Please provide an amended statement that declares *all* the funding or sources of support (whether external or internal to your organization) received during this study, as detailed online in our guide for authors at http://journals.plos.org/plosone/s/submit-now. Please also include the statement “There was no additional external funding received for this study.” in your updated Funding Statement.

Please include your amended Funding Statement within your cover letter. We will change the online submission form on your behalf.

The funding statement has been updated as indicated in #2 above, and is included in our cover letter. The online submission form was updated as well to ensure the correct statement is received.

4. Please note that funding information should not appear in the Acknowledgments section or other areas of your manuscript. We will only publish funding information present in the Funding Statement section of the online submission. Please remove any funding-related text from the manuscript.

Thank you for letting us know. The funding information from the manuscript has been removed.

5. When completing the data availability statement of the submission form, you indicated that you will make your data available on acceptance. We strongly recommend all authors decide on a data sharing plan before acceptance, as the process can be lengthy and hold up publication timelines. Please note that, though access restrictions are acceptable now, your entire data will need to be made freely accessible if your manuscript is accepted for publication. This policy applies to all data except where public deposition would breach compliance with the protocol approved by your research ethics board. If you are unable to adhere to our open data policy, please kindly revise your statement to explain your reasoning and we will seek the editor's input on an exemption. Please be assured that, once you have provided your new statement, the assessment of your exemption will not hold up the peer review process.

Thank you very much for pointing out that this was not clear. As mentioned in the manuscript main text, this is a protocol document and does not include any data from CAPTURE ALS. All data relating to the protocol are included in the manuscript text or in the supplemental materials, and will be available without requiring further contact. Any additional specific information regarding the study procedures and protocols can be directed to the corresponding author from any qualified investigator.

The Data Availability statement was removed from the manuscript, and updated in the submission portal:

Standard operating procedures (SOPs) and selected case report forms are provided in supporting information.

There is no data published in this manuscript, except for recruitment numbers.

The CAPTURE ALS protocol, SOPs, and information on how to access data and biosamples from the CAPTURE ALS database are published on http://captureals.ca. Data and biosamples can be requested through open science access principles through this URL: https://cbigr-open.loris.ca/. Data will start to be accessible at the beginning of Q4 of 2025.

6. Please include a caption for Figure 1.

Any reference to Figure 1 was removed. Therefore, a caption is not included. Apologies for the confusion.

7. Please upload a copy of Figure 1, to which you refer in your text on page 15. If the figure is no longer to be included as part of the submission please remove all reference to it within the text.

Thank you. As noted in #6, there is no reference to Figure 1.

8. Please include captions for your Supporting Information files at the end of your manuscript, and update any in-text citations to match accordingly. Please see our Supporting Information guidelines for more information: http://journals.plos.org/plosone/s/supporting-information.

Thank you for noting this. We appreciate the details. The attached URL was referenced, and we confirm that Supporting Information guidelines have been followed in the resubmission.

Please review your reference list to ensure that it is complete and correct. If you have cited papers that have been retracted, please include the rationale for doing so in the manuscript text, or remove these references and replace them with relevant current references. Any changes to the reference list should be mentioned in the rebuttal letter that accompanies your revised manuscript. If you need to cite a retracted article, indicate the article's retracted status in the References list and also include a citation and full reference for the retraction notice.

Thank you for this important comment and consideration. We conducted a review of the references, and there are no cited papers that have since been retracted. We can add that there are 61 scientific peer-reviewed papers out of a total of 73 references. The other references are websites. References have since been added to the list to respond to the reviewer's comments.

Thank you for sharing the responses to the reviewers' questions.

Reviewers

Reviewer #1: This is a protocol paper of an important study on ALS in Canada.

I have the following concerns/questions.

1. Regarding diagnosis of ALS, what do the authors think about the Gold Coast criteria? The Gold Coast criteria do not necessarily need UMN signs to diagnose ALS; patients with PMA can be diagnosed with ALS. On the other hand, "possible ALS" of the rEE criteria may include PLS but not PMA. Well-defined PMA, which is readily diagnosed as ALS based on the Gold Coast criteria, would be critical to this cohort because it is to address the heterogeneity of ALS. Recruiting ALS patients without UMN signs (i.e., PMA) would also merit the cohort because it follows up patients longitudinally and may reveal the clinical and imaging-based natural course of PMA, e.g., what proporion of patients with PMA later develops UMN signs or imaging abnormalities

Thank you very much for raising this excellent point. There is growing literature regarding the use of the Gold Coast criteria for ALS diagnosis and research studies. At the time of CAPTURE ALS's protocol development, this was prior to the original publication of the Gold Coast criteria, so it was not included. We agree with the Reviewer's excellent points that the use of the Gold Coast criteria may have improved the phenotypic range of ALS cases in our study. We have added a section to the discussion in the section "Limitations" addressing this very important point.

2. The ALSFRS-R SOP has recently been harmonized between ENCALS/TRICALS and NEALS. Do the authors consider using the harmonized SOP?
Thank you for sharing this question with us. One of CAPTURE ALS' Executive Committee members is an author on the referenced paper and we do appreciate the work and collaboration that was put into this harmonized version. CAPTURE ALS' ALSFRS-R SOP is based primarily on the ENCALS SOP, except for the breathing items which are rely on the NEALS SOP. In order to maintain data and rater consistency, the current SOP will remain. However, when CAPTURE ALS is ready to expand and enter another iteration to continue data and biosample collection, this harmonized version will be implemented to ensure consistency across all international studies and platforms.

Reviewer #2: This article describes a long term natural history study of ALS in Canada, as well as a data collection effort from healthy control subjects. This study exists in the context of other, quite similar efforts occuring in the US and in Europe. All of these efforts have more similarities than differences. If, as contended by all groups, the strengths of these effort lie in their large scales and

thus potential to identify potentially different ALS genetic groups, other non genetic risk factors, or potentially disease modification targets unique to specific ALS subtypes, then it would behoove all parties to proactively determine a data analysis plan that merged the data in common. All groups including this one plan on open source data, so that a formal plan for this should not be impossible. I recognize that this is not a unique issue to this project, but one that should be raised within all groups. For the purpose of this manuscript, I'd suggest that the authors consider this and decide whether such an aggregation effort is important and necessary. If so, a discussion on how they would propose to proceed would be warranted. If not, this should be discussed and justified.

Thank you for raising this excellent point, which emphasizes the importance of collaboration in the global effort to better understand ALS. We agree wholeheartedly that it would be ideal for data to be collected in a way that allows it to be easily compared to that from other projects. In the case of CAPTURE ALS, there is a specific emphasis on deep phenotyping, neuroimaging, and biomarkers from multiple biosample types (DNA, DNA methylation data, RNA, plasma NfL), and longitudinal data collection. Based on the goals of the project, the precise data points and timing of data collection will vary. However, despite the obvious challenges, there is excellent potential in aggregation efforts as suggested by the Reviewer, and we have added material to the discussion in the “Limitations” section to address this.

In hopes of future broad harmonization and interoperability, the protocol was set up with reflection on what other groups and platforms were collecting, and we shared our protocol with others. We consulted others operating other initiatives such as Robert Bowser.

Sincerely,

Natalie Saunders and Dr. Sanjay Kalra
